# Comparative proteomic analysis of liver antioxidant mechanisms in *Megalobrama amblycephala* stimulated with dietary emodin

**DOI:** 10.1038/srep40356

**Published:** 2017-01-13

**Authors:** Changyou Song, Bo Liu, Jun Xie, Xianping Ge, Zhenxin Zhao, Yuanyuan Zhang, Huimin Zhang, Mingchun Ren, Qunlan Zhou, Linghong Miao, Pao Xu, Yan Lin

**Affiliations:** 1 Wuxi Fisheries College, Nanjing Agricultural University, Wuxi, 214081, China; 2 Key Laboratory of Freshwater Fisheries and Germplasm Resources Utilization, Ministry of Agriculture, Freshwater Fisheries Research Center, Chinese Academy of Fishery Sciences, Wuxi, 214081, China

## Abstract

Oxidative stress is a toxicological endpoint that correlates with the nutrition status of fish through cellular damage, inflammation, and apoptosis. In order to understand the antioxidant mechanism induced by dietary emodin in *Megalobrama amblycephala* liver, a comparative proteomic analysis was performed to investigate the proteome alteration under emodin administration. 27 altered protein spots were separated under 30 mg kg^−1^ emodin stimulation based on 2-DE, and were all successfully identified using MALDI-TOF/TOF, representing 17 unique proteins. These proteins were functionally classified into antioxidant, metabolism, cytoskeleton, chaperone, signal transduction and cofactor groups. Network interaction and Gene Ontology annotation indicated 10 unique proteins were closely related to antioxidation and directly regulated by each other. Compared with the control group, administration of 30 mg kg^−1^ emodin significantly increased the antioxidant-related mRNA expressions of *GPx1*, *GSTm* and *HSP7*0, but decreased the mRNA expressions of *GAPDH* and *Sord*, which was consistent with the protein expression. Nevertheless, *Pgk1* and *Aldh8a1* were up- and down-regulated, and *ALDOB* was down- and up-regulated at the mRNA and protein levels, respectively. These results revealed that the altered proteins enhanced antioxidation via complex regulatory mechanisms, and 30 mg kg^−1^ emodin was a suitable immunostimulant for *M. amblycephala*.

Fish health and growth depend on a stable internal environment of the body, including a balanced antioxidant defence system. Immunostimulant is an effective nutritional feed additive that typically affects antioxidant and immunity mechanisms^1^, which provide protection against a wide range of adverse stresses. Purivirojkul^2^ reported that 0.18 g kg^−1^ dietary peptidoglycan significantly enhanced the superoxide anion, phenoloxidase, and bactericidal activity in shrimp. Other research documented that oral administration of vitamin C and Chinese herbs effectively improved immune activity and vibriosis resistance capability in the shrimp *Litopenaeus vennamei*^3^. In addition, studies have confirmed that dietary folic acid, probiotics and chitosan can increase the antioxidant activity of *M. amblycephala*^4^, gilthead seabream^5^ and Nile tilapia^6^, respectively. Emodin is an active anthraquinone that is extracted from the rhizomes of rhubarb (*Rheum officinale* Bail)^7^. In mammals, functional research investigating emodin has typically focused on its antitumor, anti-bacterial, anti-inflammatory, antioxidant, liver protective, and immune regulatory capabilities^8^,^9^,^10^. Previous studies have shown that dietary emodin can improve growth^11^, enhance immunity^12^, and provide antioxidant properties^13^ in *M. amblycephala*.

Oxidative stress results from an imbalance between pro-oxidants and anti-oxidant defense systems. Pro-oxidants include reactive oxygen species (ROS) that primarily arise as by-products of normal metabolic activities^14^ and have been reported to be involved in the toxicity of several xenobiotics^15^,^16^. If not scavenged properly, pro-oxidants may cause oxidative damage to proteins, nucleic acids and other macromolecules^17^. Under pro-oxidant conditions, there is a need for a cellular defense system to maintain normal metabolism^18^. Therefore, enhancement of antioxidant activity through nutrition is of much interest in intensive aquaculture strategies.

Proteins play an essential role in a variety of cellular functions, and oxidative damage can reduce the function of critical proteins^19^; traditional physiological and biochemical methods cannot determine the molecular mechanisms underlying this phenomenon. Proteomic approaches are being increasingly applied in aquaculture on the premise that the identification of specific changes in protein expression in response to a particular challenge can elucidate the underlying molecular pathways^20^,^21^,^22^. More recently, nutritional omics has also become a research focus aiming at understanding how diet influences gene transcription, protein expression and metabolite synthesis^23^. In reviews, Kussmann^24^,^25^ considered the application of proteomics in nutritional research to contribute to the identification of bioactive food components, assess their biological efficacy, and to elucidate biomarkers for defining the susceptibility of an individual to diet in nutritional interventions.

*M. amblycephala* is one of the most widely cultivated freshwater fish species in China, and has also been introduced to America, Africa and Euro-Asia. *M. amblycephala* has great consumer demand in China, and its production reached 0.73 million tons in 2014^26^. Nevertheless, in an intensive culture system, cultivated *M. amblycephala* has suffered adverse stressors from their living surroundings, such as overcrowding, high temperature, poor water quality, and pathogen infection. All these adverse stressors could lead to poor growth performance^12^, disease outbreak^27^ and even massive mortalities^28^,^29^. Therefore, to alleviate the effects of these stressors should be an important goal of aquaculture research.

As one of the most important defensive tissues, liver combats oxidative stress caused by excessive ROS and plays an important role in the metabolism of different nutrients and toxins^30^. Recently, we found that 30 mg kg^−1^ emodin was the optimum level of dietary supplementation to enhance the growth performance, non-specific immunity, and disease resistance to *Aeromonas hydrophila*^12^ (shown in Supplementary Material 1). Although research has shown that emodin affects both immunity and antioxidation, the underling molecular mechanisms are still not entirely understood. Thus, the 30 mg kg^−1^ group was selected to examine the altered antioxidant-related proteins of livers of *M. amblycephala* fed with dietary emodin using proteomic approaches. Furthermore, identification and expression analysis of these proteins at mRNA and protein levels elucidated the antioxidant molecular mechanism associated with emodin. To our knowledge, this is the first documented application of a proteomic approach in *M. amblycephala* research stimulated with dietary emodin. Our results provide new insights into the anti-oxidative responses and molecular mechanisms underlying the protective effect of emodin on *M. amblycephala.*

## Results

### 2-DE and classification of altered proteins

**Figure 1:**
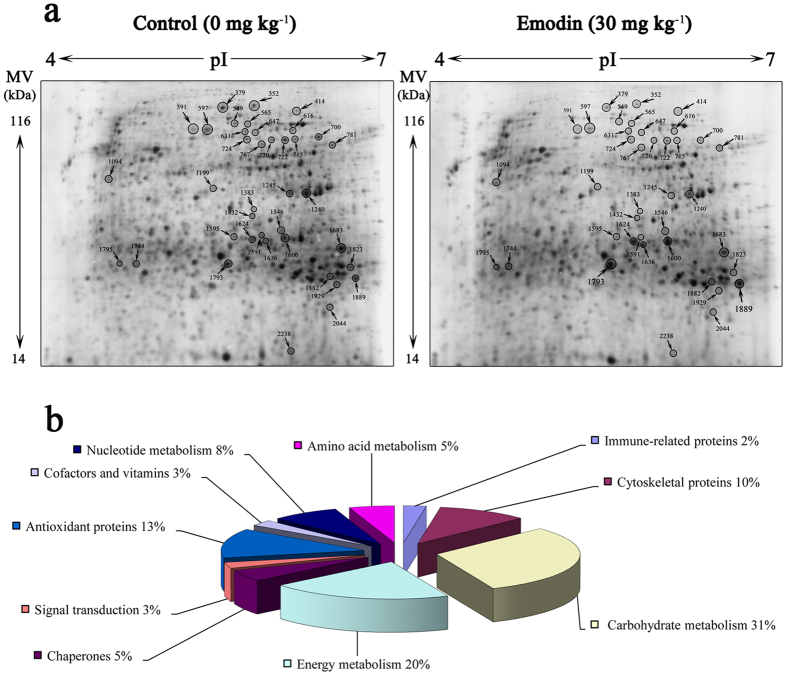
The 2-DE reference maps and functional classification of altered proteins derived from the hepatic cells of the control and 30 mg kg^−1^ emodin groups (pI 4-7). (**a**) Significantly increased or decreased proteins are denoted by a circle, arrow and spot number. Molecular masses of marker proteins and pI are mentioned on the left side and at the top, respectively. (**b**) The identified *M. amblycephala* liver proteins were classified based on their GO annotations for biological processes and molecular functions.

Figure 1a shows the 2-DE maps for the control and 30 mg kg^−1^ emodin groups after an 8-week feeding trial. A total of 27 significantly altered protein spots between the control and emodin groups (up/down-regulated ≥2 fold) were detected, including 6 up-regulated and 21 down-regulated spots. Eventually, all protein spots were identified and were found to represent 17 unique proteins (shown in Table 1). The MALDI-TOF analysis results were shown with Target No. of altered proteins in Supplementary Material 2. The peptides for MS/MS identification were shown in Supplementary Material 3.

The identified *M. amblycephala* liver proteins could be classified into 9 categories based on their GO annotations for biological processes and molecular functions (shown in Table 1 and Fig. 1b). Approximately 38% of the identified protein spots (10 spots containing 6 unique proteins) were functionally determined to participate in carbohydrate metabolism, representing the most abundant category in this study. Proteins related to energy metabolism (19% of the spots, 5 spots, 2 unique proteins) came the second in terms of the amount. Another large set of identified proteins belonged to the cytoskeletal category (15% of the spots, 4 spots, 2 unique proteins). Other categories associated with the identified proteins included antioxidant proteins (7% of the spots, 2 spots, 2 unique proteins), nucleotide metabolism (7% of the spots, 2 spots, 1 unique protein), chaperones (4% of the spots, 1 spot), amino acid metabolism (4% of the spots, 1 spot), cofactors and vitamins (4% of the spots, 1 spot), and signal transduction (4% of the spots, 1 spot). Proteins related to carbohydrate and energy metabolism were predominant and accounted for approximately 57% of the altered proteins.

### Network analysis and functional annotation of altered proteins

**Figure 2:**
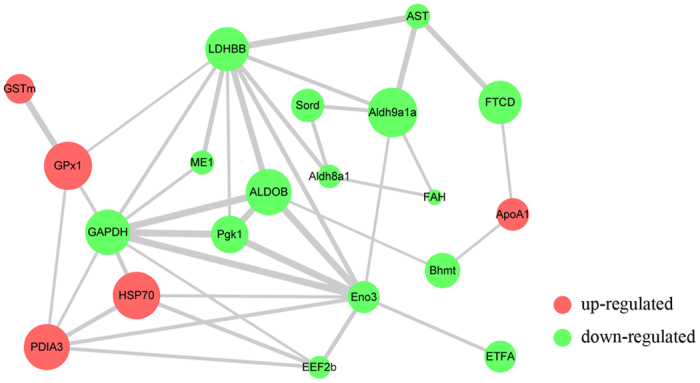
Network interaction of altered proteins identified in the 30 mg kg^−1^ emodin group. In the network, each node represents a protein; nodes in red and green color represent up- and down-regulated proteins, respectively. The size of a node represents an average of the protein abundance. The width of a line connecting proteins represents the intensity of the protein interaction, as extracted from STRING database.

To further explore the potential antioxidant-related proteins in *M. amblycephala*, network interaction and functional annotation analysis were performed. The interaction network was constructed using Cytoscape based on the protein expression database, and the interaction and intensity retrieved from NCBI and STRING. The results indicated that 15 unique proteins had important roles in the interaction network (shown in Fig. 2).

Concomitantly, the annotation results indicated that 10 proteins were functionally involved in antioxidant processes, including fructose-bisphosphate aldolase B (*ALDOB*), glyceraldehyde-3-phosphate dehydrogenase (*GAPDH*), glutathione peroxidase 1 (*GPx1*), glutathione S-transferase mu (*GSTm*), heat shock 70 kDa protein (*HSP70*), malic enzyme 1 (*ME1*), L-lactate dehydrogenase B-B (*LDHBB*), phosphoglycerate kinase 1 (*Pgk1*), aldehyde dehydrogenase family 8 member A1 (*Aldh8a1*) and sorbitol dehydrogenase (*Sord*). According to the interaction network, these 10 antioxidant-related proteins were directly regulated by each other, and 5 of them (*ALDOB*, *GAPDH*, *GPx1*, *GSTm*, and *HSP70*) were up-regulated while the other 5 (*Aldh8a1*, *Sord*, *ME1*, *LDHBB*, and *Pgk1*) were down-regulated at the protein level. Among the antioxidant-related proteins, *ALDOB* and *GAPDH* were functionally annotated to energy metabolism, *GPx1* and *GSTm* were annotated to antioxidants, *HSP70* was chaperones, and the remaining 6 proteins (*ME1*, *LDHBB*, *Pgk1*, *Aldh8a1*, *Eno3* and *Sord*) played a role in carbohydrate metabolism. Considered together, these results suggested that the 10 uniquely expressed proteins could be related to the enhancement of the antioxidant reaction in *M. amblycephala* hepatic cells.

Besides, altered proteins of keratin 8 (*krt8*, up-regulated) and keratin 18 (*krt18*, down-regulated) were not involved in the network interaction. The other 5 proteins of eukaryotic translation elongation factor 2b (*EEF2b*), beta-enolase (*Eno3*), betaine-homocysteine S-methyltransferase 1 (*Bhmt*) and aspartate aminotransferase (*AST*) were down-regulated, whereas apolipoprotein A-I (*ApoA1*) was up-regulated since it was involved in network interaction but not directly related to antioxidation.

### RT-PCR analysis of altered proteins

**Figure 3:**
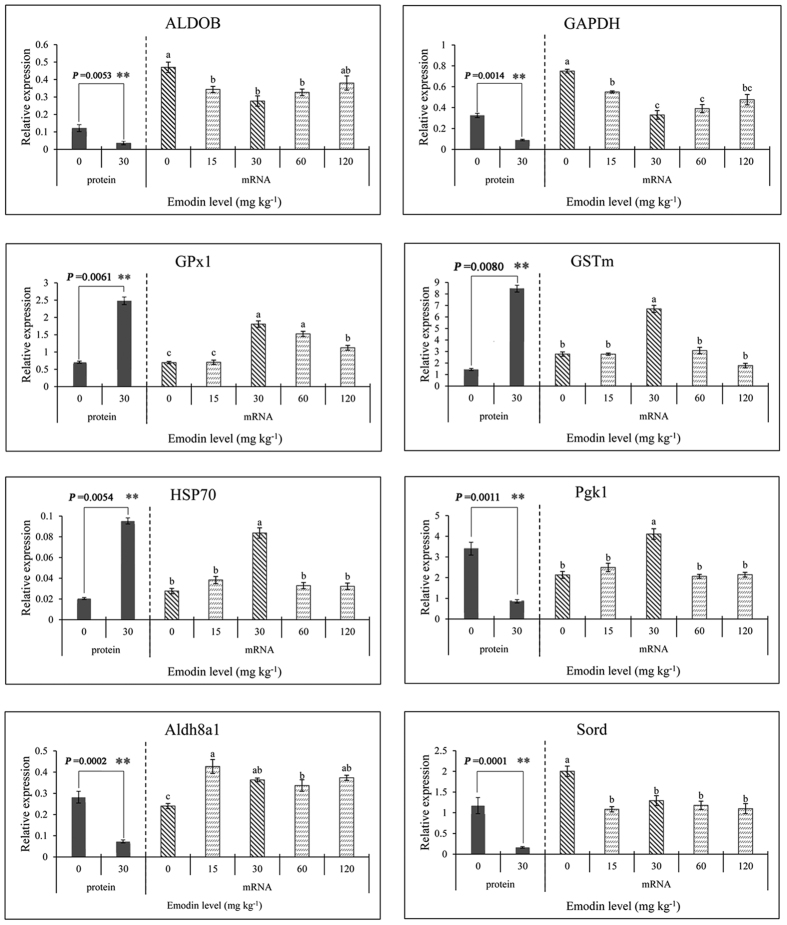
Relative expression of antioxidant-related proteins at protein and mRNA levels. Diverse little letters above histogram bars show significant differences (*P* < 0.05) in different dosage groups in Duncan’s multiple range tests. Significant differences (*P* < 0.05 or *P* < 0.01) between values obtained the control 30 mg kg^−1^ emodin group were marked by asterisks above histogram bars in Independent-Samples t-tests.

Among the identified proteins, 8 key proteins out of 10 involved in antioxidation were selected for further expression pattern analysis at the transcript level. The RT-PCR results (shown in Fig. 3) indicated that 30 mg kg^−1^ emodin significantly improved the mRNA expression of *GPx1, GSTm, HSP70*, *Pgk1* and *Aldh8a1* compared with the control group (*P* < 0.05). In contrast, the mRNA expressions of *ALDOB*, *GAPDH*, and *Sord* were significantly decreased (*P* < 0.05) during dietary emodin stimulation. In addition, the mRNA expressions of *GPx1, GSTm, HSP70* and *Pgk1* first increased and then decreased with the increasing of dietary emodin levels, with the highest expression being observed in the 30 mg kg^−1^ group (*P* < 0.05); *ALDOB* and *GAPDH* showed a reverse pattern, exhibiting the lowest expression in the 30 mg kg^−1^ group (*P* < 0.05).

### Expression analysis at the mRNA and protein levels

**Figure 4:**
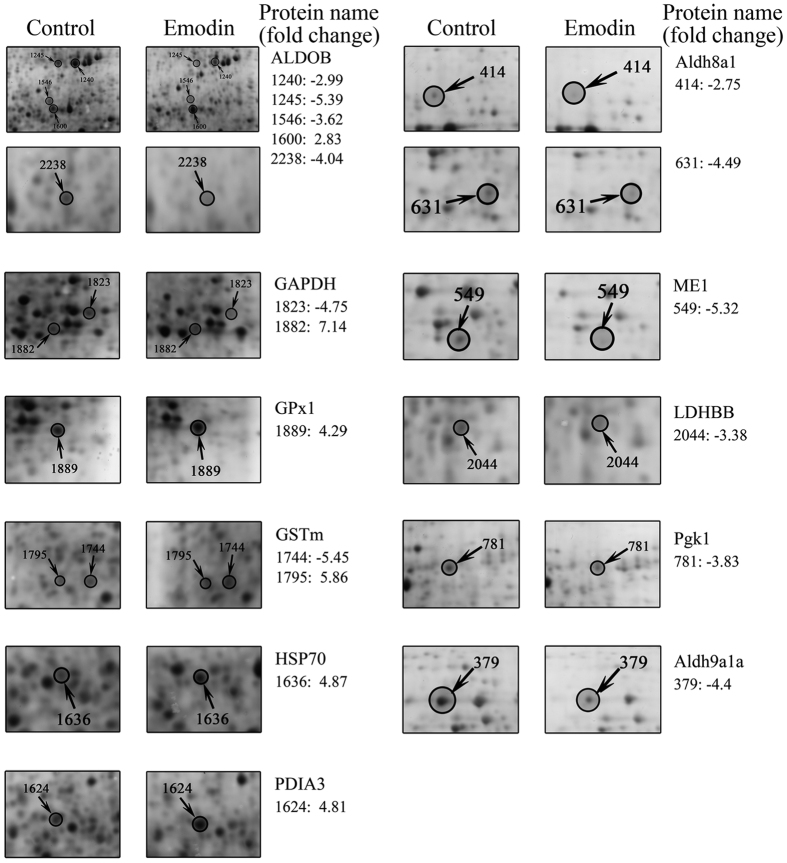
Emodin-responsive changes in the abundance of antioxidant-related proteins. Differentially expressed proteins with increased or decreased abundance are marked in the control and 30 mg kg^−1^ groups. Fold changes of protein spots are mentioned on the right side.

The mRNA and protein expression trends observed for antioxidant-related proteins were generally influenced by dietary emodin (shown in Figs 3 and 4). More precisely, the representative 2D protein profiles of the control and 30 mg kg^−1^ emodin groups are shown in Fig. 3. *GPx1*, *GSTm* and *HSP70* were up-regulated at the mRNA and protein levels in the 30 mg kg^−1^ group compared with the control group, while *GAPDH* and *Sord* were down-regulated at both levels. Conversely, the expression levels of *Pgk1* and *Aldh8a1* were up- and down-regulated at mRNA and protein levels, while *ALDOB* was down-regulated at the mRNA level but up-regulated at the protein level. Briefly, these results suggested that the 10 altered antioxidant-related proteins might be involved in the enhancement of antioxidation in *M. amblycephala* via a complex molecular regulatory mechanism.

### Heatmap analysis of altered proteins at mRNA and protein levels

**Figure 5:**
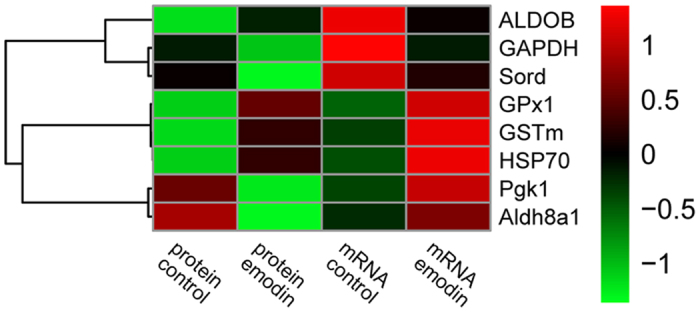
Heatmap of antioxidant-related altered proteins based on mRNA and protein expressions. In this figure, weak and strong correlations between variables are displayed in green and red, respectively. The dendrogram for strain clustering is shown on the left-hand side of the heatmap. The width of the cluster merged from the left side represents the distance of the two clusters.

Figure 5 presents the heatmap based on the Spearman correlation matrix of the antioxidant-related altered proteins. In this figure, the proteins were clustered into 3 groups based on the mRNA and protein expression under 30 mg kg^−1^ emodin stimulation: *ALDOB*, *GAPDH*, and *Sord* representing the first group; *GPx1*, *GSTm*, and *HSP70* the second group; and *Pgk1* and *Aldh8a1* the third group. The heatmap results indicated that the expression patterns of the second clustered group under emodin stimulation were up-regulated at both mRNA and protein levels compared with the control group. Meanwhile, the expressions in the third clustered group were up-regulated and down-regulated at mRNA and protein levels, respectively.

### Emodin induced antioxidant mechanism analysis

**Figure 6:**
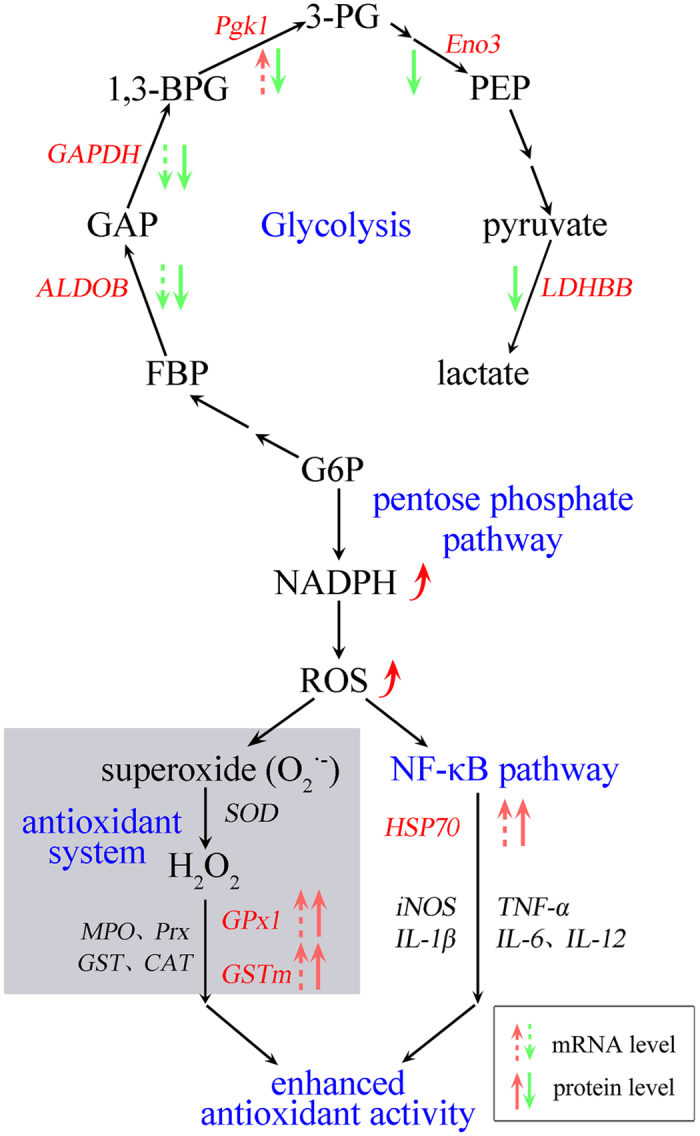
Antioxidant-related mechanism of altered proteins under emodin stimulation in *M. amblycephala*. Abbreviations in red represent the altered proteins in present study. Dashed arrows are used for mRNA expression, and solid arrows are used for protein expression. The up-arrow in red represents up-regulation, and down-arrow in green represents down-regulated. G6P, glucose-6-phosphate; FBP, fructose-1,6-bisphosphate; GAP, glyceraldaldehyde-3-phosphate; 1,3-BPG, 1,3-bisphosphoglycerate; 3-PG, 3-phosphoglycerate; PEP, phosphoenolpyruvate; SOD, superoxide dismutase; MPO; CAT, catalase; NF-κB, nuclear factor κappa Β; iNOS, inducible nitric oxide synthase; TNF-α, tumor necrosis factor-α; IL-1β, Interleukins-1β; IL-6, Interleukins-6; IL-12, Interleukins-12.

Antioxidant mechanism induced by dietary emodin is elucidated in Fig. 6. The down-regulated expression of altered proteins (*ALDOB*, *GAPDH*, *Pgk1*, *Eno3*, and *LDHBB*) inhibited the glycolysis pathway, but inversely activated NADPH through pentose phosphate pathway (PPP). Simultaneously, NADPH stimulated ROS production, then activated the antioxidant system and NF-κB pathway by enhancing the expression of *GPx1*, *GSTm*, and *HSP70*, which synergically enhanced the antioxidant mechanism. These results were complemented with the network interaction and GO annotation analysis.

## Discussion

In the present study, the identified proteins were functionally categorized and annotated according to the gene ontology of biological processes and molecular functions. Accordingly, the altered proteins could be classified into 9 categories, indicating their complex regulatory relationships in response to dietary emodin. To gain deeper insight into the antioxidant mechanism induced by dietary emodin, the uniquely altered proteins were then subjected to the network instruction and GO annotation. Protein-protein interaction networks provided insights into the functional mechanisms of living cells^31^. GO annotation analysis of the differentially expressed proteins revealed more information about protein functions in the antioxidant pathway. Notably, 15 proteins were involved in the interaction network, and interestingly, 10 of them were directly regulated by each other and functionally related to antioxidation. These results revealed that the administration of dietary emodin had significant effects on the regulation of antioxidation in *M. amblycephala*, and the 10 altered proteins might play important roles in the antioxidant response to dietary emodin.

Moreover, 2 altered cytoskeleton proteins of krt8 and krt18 were not involved in the network interaction, while the other 5 network interacted proteins (EEF2b, Eno3, Bhmt, AST, and ApoA1), which were respectively identified as nucleotide metabolism, carbohydrate metabolism, cofactors, amino acid metabolism and signal transduction proteins, were not related to antioxidation.

To clearly elaborate the dynamics underlying the expression of antioxidant-related proteins at the transcript level, 8 proteins that were closely related to antioxidant metabolism were subjected to mRNA expression analysis by RT-PCR. All quantified mRNA levels exhibited highly dynamic mRNA profiles from the control to the 120 mg kg^−1^ emodin group. Some proteins showed different expression patterns at the mRNA compared with those at the protein level. For example, *Pgk1* and *Aldh8a1* were up-regulated at the mRNA level but down-regulated at the protein level, while *ALDOB* was down-regulated at the mRNA level but up-regulated at the protein level, which potentially revealed the evidence that transcription and translation are differentially regulated and timed. In addition, these results will urge us to be careful when interpreting the mechanisms underlying protein expression at the transcriptional or translational level. Briefly, the gene-specific unique mRNA expression patterns supported the importance of antioxidant-related gene regulations during emodin administration.

We also applied a so-called heatmap analysis to the Spearman correlation matrix. The heatmap also re-arranges the rows and columns of the data so that similar rows, and similar columns, are grouped together, with their similarity represented by a dendrogram. The heatmap results indicated that the key variables of altered proteins stimulated by dietary emodin are comparable with the results from the RT-PCR analysis.

Liver is one of the most important organs for obtaining an overall assessment of the immune response and antioxidant activity. In this study, 10 uniquely expressed proteins in liver were functionally related to antioxidation based on a proteomic analysis.

One remarkable change in the proteome in response to emodin administration was the alteration of antioxidant proteins^32^. GPx, an antioxidant enzyme, is an important component of the antioxidant system, together with SOD, CAT, and GST. The level of GPx activity reflects the redox level in the fish body, and is a common physiological index to quantify oxidative stress in fish^33^. GSTm is a membrane-bound protein that has been shown to protect cells against oxidative stress^34^,^35^. Over-expression of GSTm can impact antioxidant stress status via lipid oxidation^36^. In the present study, both enzymes substantially increased at mRNA and protein levels in response to emodin administration, potentially because they had a common substrate of reduced glutathione. These results were consistent with previous reports that emodin could enhance antioxidant levels^37^,^38^. Thus, the two enzymes are the primary defense components and play an important role in cellular protection against adverse effects and in the antioxidant pathway.

In the present study, a chaperone protein (HSP70) was functionally related to antioxidant metabolism. There is growing evidence that over expressed HSP70 can mediate oxidative activity^39^. Another research in our lab also demonstrated that HSP70 could have the relationship with antioxidant system^13^. HSP70 protects cells against oxidative damage either due to necrosis or apoptosis^40^,^41^. For example, HSP70 is a potent regulator of inflammation because of its ability to prevent activation of the NF-κB pathway^42^. Moreover, it has been well documented that oxidative stress, especially hyperoxia, stimulates the expression of HSP70^43^. In addition, ROS are well known to cause protein misfolding^44^. HSP70 is integrally related to antioxidation by regulating the ROS level and modulating the redox status of the cytosol via reactions between their cysteine groups and cytochrome C^41^,^45^. In the present study, HSP70 was up-regulated following emodin administration, which was consistent with our previous studies^7^, and equivalent findings have been reported in other aquatic animals^46^,^47^. In summary, we hypothesize that HSP70 can alleviate antioxidant stress via a mutual regulation of other identified proteins.

Among the altered proteins in the liver of *M. amblycephala*, ALDOB and GAPDH have been associated with energy metabolism. ALDOB and GAPDH are well known for their role in glycolysis^36^,^48^. In addition to the roles in antioxidant regulation, these two proteins have also been implicated in apoptosis and cellular stress^49^,^50^, as well as frequently associated with oxidative stress^51^,^52^. In the present study, ALDOB was down-regulated at the mRNA level but up-regulated at protein level, while GAPDH was down-regulated at both levels. These expression patterns may potentially due to more efficient transport and utilization of nutrition, which results in an increase in the energy available for growth^53^. In addition, it might reflect the differences in energy demands between emodin administration and the control groups. Moreover, apoptosis has been linked to inflammation^54^, and the increased expression of ALDOB and decreased expression of GAPDH at protein level were associated with increased expressions of HSP70, GPx1, and GSTm in the present study, revealing enhanced immunity.

Moreover, proteins that play a role in carbohydrate metabolism (Pgk1 and Aldh8a1) were also altered under emodin administration. Pgk1 was reported to suppress ROS production and coordinate glycolysis and the TCA cycle^55^, which have been shown to decrease oxidative stress^55^,^56^. In the present study, the interaction network also demonstrated that these carbohydrate-associated proteins might modulate antioxidant activity. For example, Pgk1 and Aldh8a1 were all up-regulated at the mRNA level but down-regulated at the protein level in response to emodin administration, which might provide the evidence that transcription and translation are differentially regulated and timed.

Meanwhile, the antioxidant-related mechanisms highlight the modulation of altered proteins involved in the glycolysis, pentose phosphate pathway, NF-κB pathway, and antioxidant system. In the current study, the glycolysis process was suppressed with the regulating of ALDOB, GAPDH, Pgk1, Eno3 and LDHBB under dietary emodin stimulation. Reports also suggested that there was a metabolic balance of glucose-6-phosphate (G6P) consumption between pentose phosphate pathway (PPP) and glycolysis^57^, and increased G6P could activate NADPH^58^. Besides, the NADPH oxidase enzymes (NOXes) are the major sources of ROS^59^. Therefore, ROS was generated with the activation of NADPH. The ROS induced enhancement of antioxidant activity has been well documented^60^. NF-κB is targeted as an integral messenger in the enhancement of the response to environmental perturbation, which activates a series of cellular genes including HSP70, iNOS, IL-1β, IL-6, IL-12 and TNF-α^41^,^61^,^62^ to enhance the antioxidant mechanism. Consequently, dietary emodin enhanced the antioxidant mechanism by glycolysis, pentose phosphate pathway, NF-κB pathway and antioxidant system. A complete understanding of the mechanisms underlying this pattern of expression requires further elucidation of protein functions, modification, regulation, and protein-protein interactions, but we speculate that the regulation at the protein level might reflect a low level of inflammation and oxidative stress in the *M. amblycephala*. Furthermore, these up- or down-regulated proteins play important roles in the oxidative defense of fish.

In conclusion, the present work revealed that 10 altered proteins were involved in antioxidant process by regulating each other, suggesting the beneficial effects of dietary emodin on antioxidant mechanisms in *M. amblycephala*. Our data showed that 27 protein spots responded to 30 mg kg^−1^ dietary emodin, all of which were successfully identified (6 up-regulated and 21 down-regulated), representing 17 unique proteins. The results also explicitly demonstrated that 30 mg kg^−1^ dietary emodin induced different expression patterns of various antioxidant related proteins at the transcript level (*GPx1*, *GSTm*, *HSP70*, *Pgk1*, and *Aldh8a1* were increased, and *ALDOB*, *GAPDH*, and *Sord* were decreased). The altered proteins were directly regulated by each other, and 10 proteins categorized as related to energy metabolism, antioxidants, chaperones, and carbohydrate metabolism were responsible for the reduction of oxidative stress. Although additional work is required to evaluate the precise regulatory mechanisms of the identified proteins, the results of the present study may indicate the antioxidative properties of dietary emodin in *M. amblycephala*.

## Methods

### Ethics statement

This study was approved by the Animal Care and Use Committee of Nanjing Agricultural University (Nanjing, China). All animal procedures were performed according to the Guideline for the Care and Use of Laboratory Animals in China.

### Experimental animals and feeding experiments

Healthy *M. amblycephala* fingerlings with an average weight of 3.94 ± 0.15 g were obtained from the Freshwater Fisheries Research Center, Chinese Academy of Fishery Sciences. Two weeks acclimation was conducted prior to the feeding experiments. After acclimation, 375 fishes were randomly distributed into five groups (15 circular fiberglass tanks, 300 L water/tank, 3 tanks/group, 25 individuals/tank): the control group fed the basal diet (0 mg kg^−1^ emodin) and the treatment groups fed experimental diets (15, 30, 60 and 120 mg kg^−1^ emodin) by hand to apparent satiation three times daily (08:30, 12:00, and 16:00) for 8 weeks.

The emodin used in this study was purchased from Xi’an Feida Bio-Tech Co., Ltd, China (purity >99%). Formulation of the experimental diets and environmental quality preferences were performed according to established protocols^12^.

### Sample collection and soluble protein preparation

After a rearing period of 8 weeks, the fish were fasted for 24 h, and then 9 livers from each group (3 individuals/tank, 3 tanks/group) were dissected individually, frozen in liquid nitrogen, and stored at −80 °C for further analysis. The 3 livers (0.5 g/each) in each tank of the control and 30 mg kg^−1^ group were randomly mixed together and chopped into 3 mm × 3 mm pieces. Protein extraction was performed using a homogenizer in lysis buffer containing 7 M urea, 2 M thiourea, 65 mM Tris, 4% CHAPS and 0.2% IPG buffer; enzyme inhibitor was added simultaneously at a ratio of 50:1. Protein extracts were centrifuged at 12000 rpm for 30 min at 4 °C, washed three times, lyophilized and re-dissolved in lysis buffer. The protein concentration was estimated using a modified Bradford assay (Bio-Rad, USA) with BSA as a standard^63^.

### Two-dimensional gel electrophoresis

Three biological replicates in each group were considered for 2-DE analysis. The preliminary experiment showed that the pI of most proteins ranged from 4 to 7, and the strip of 4–7 exhibited a clearer 2-DE spectrum. In order to achieve a better separation of liver proteins, a narrow strip of 4–7 was employed for further analysis. A total of 500 μg of liver proteins in rehydration buffer (7 M Urea, 2 M thiourea, 4% CHAPS, 65 mM DDT and 3.4 μl of IPG buffer) was used to rehydrate the IPG strip (7 cm, pH 4–7, Bio-Rad, USA) for 16 h. The IEF was performed at a constant temperature of 20 °C with a continuous increase in voltage up to 8000 V with an Ettan IPGphor Isoelectric Focusing System (GE Amersham, Sweden). For second dimension electrophoresis, 14% SDS-PAGE was performed using a Hofer SE 600 (GE Amersham, Sweden) system.

The gel was stained with Coomassie G-250 (10% w/v ammonium sulfate, 10% v/v phosphoric acid, 20% v/v methanol and 0.12% w/v CBB G-250) for at least 2 h until saturation, then destained in water for 30 min until the background is clear.

### Image analysis

The 2-DE images were scanned with a UMAX Power Look 1120 scanner (UMAX, Taiwan) at 400 dpi and analyzed with Image Master 2D Platinum 6.0 (GeneBiology, Sweden). Comparative analysis of the protein spots was conducted by matching corresponding spots across different gels. The spot intensity was quantified by total spot volume normalization, and the statistical comparison was performed using the resulting spot volume percentage. Only proteins with significant changes (*P* < 0.05, up/down-regulation ≥2.0-fold) were considered differentially expressed and subjected to subsequent identification by MALDI-TOF mass spectrometry.

### MALDI-TOF-MS and MS/MS

To identify the proteins of interest, the altered spots were subjected to MALDI-TOF analysis. Before MS analysis, the differentially expressed protein spots were manually excised from the gels for in-gel digestion. The gel pieces were digested in 10 μg/mL trypsin at 37 °C for 24 h. The digested samples were collected and vacuum-dried, dissolved in 50% v/v ACN containing 5% v/v TFA and subjected to MALDI-TOF/TOF.

MS spectra were obtained using ReFlex^TM^ III MALDI-TOF (Bruker, Germany), and peptide mass maps were acquired in reflectron mode (20 kV and 23 kV accelerating voltage) with 100 laser shots per spectrum. For MS/MS spectra, 8 abundant precursor ions per sample were selected for subsequent fragmentation using 2000 laser shots per precursor ion. The selected peptides were submitted to Mascot for an NCBInr database search (database downloaded in February 10, 2012), and the tolerance was set at 1 Da. High confidence identifications with statistically significant search scores (*P* < 0.05) were regarded as a positive identification.

### Network construction and GO annotation

The associated interaction data and score were extracted from STRING (Version 10.0, http://string-db.org/). The protein network was constructed based on data retrieved and protein expression using Cytoscape 3.1.2 for further analysis.

Gene Ontology (GO) annotation of altered proteins was performed by DAVID (Version 6.7, https://david.abcc.ncifcrf.gov/) based on the information obtained for BP (Biological Processes), MF (Molecular Functions) and CC (Cellular Components).

### RNA extraction and RT-PCR analysis

Total RNA was extracted from 9 livers in each group using RNAiso Plus reagent (Dalian Takara Co. Ltd., China). Primers (shown in Table 2) for each gene were designed using primer premier 5.0 based on the mRNA sequences obtained from the *M. amblycephala* liver genome database of aquatic disease and feed laboratory of Freshwater Fisheries Research Center, Chinese Academy of Fishery Sciences (mRNA sequences were shown in Supplementary Material 4). All primers were synthesized by Shanghai Generay Biotechnology, Co., Ltd, China. Real-time quantitative PCR (RT-PCR) was performed according to Habte-Tsion^64^ with the SYBR^®^ Primix Ex TaqTM II (TliRNase Plus) Kit using an ABI 7500 Real-time PCR System. The relative expression levels of the target genes were normalized to the housekeeping *M. amblycephala* gene β-actin, and further calculated using the double-standard curve method^65^.

### Heatmap analysis

The heatmap was conducted in R with the pheatmap package. The input data is a 4 by 8 symmetric Spearman correlation matrix. The rows and columns are reordered by dendrogram, because of the symmetry of the input data, rows and columns are rearranged in the same order.

### Data analysis

Duncan’s multiple range test was used to analyze the RT-PCR results after one-way ANOVA in SPSS (version 19.0), and an independent samples t-test was conducted to analyze the protein expressions. All results are expressed as the mean ± standard error of the mean (X ± SEM).

## Additional Information

**How to cite this article**: Song, C. *et al*. Comparative proteomic analysis of liver antioxidant mechanisms in *Megalobrama amblycephala* stimulated with dietary emodin. *Sci. Rep.* **7**, 40356; doi: 10.1038/srep40356 (2017).

**Publisher's note:** Springer Nature remains neutral with regard to jurisdictional claims in published maps and institutional affiliations.

## Supplementary Material

Supplementary Information

## Figures and Tables

**Table 1:** Altered proteins in hepatic cells from the 30 mg kg^−1^ versus the control emodin group detected by 2-DE and TOF-MS/MS.

Spot No.	Target No.	Protein name	Species	Accession No.	Abbr.	T-test *P*-value	Fold change
**Cytoskeleton proteins**							
597	C6	keratin 18	*Danio rerio*	gi|29335504	Krt18	0.049	−3.71
591	C7	keratin, type I cytoskeletal 18	*Danio rerio*	gi|30410758	Krt18	0.002	−2.28
1094	C9	keratin-like protein	*Ctenopharyngodon idella*	gi|226510657	Krt8	0.017	3.50
616	D4	keratin 18	*Danio rerio*	gi|29335504	Krt18	0.002	−3.13
**Carbohydrate metabolism**							
549	C18	malic enzyme 1, NADP(+)-dependent, cytosolic	*Xenopus tropicalis*	gi|187608085	ME1	0.039	−4.87
631	C21	aldehyde dehydrogenase family 8 member A1	*Danio rerio*	gi|52218932	Aldh8a1	0.001	−3.70
647	C22	beta-enolase	*Danio rerio*	gi|47551317	Eno3	3.252e–4	−3.85
722	D6	Eno3 protein	*Danio rerio*	gi|77567762	Eno3	0.003	−2.47
715	D7	beta-enolase	*Danio rerio*	gi|47551317	Eno3	0.011	−4.99
724	C23	Eno3 protein	*Danio rerio*	gi|77567762	Eno3	1.833e–4	−2.30
720	D1	beta-enolase	*Danio rerio*	gi|47551317	Eno3	0.001	−2.06
781	D9	phosphoglycerate kinase 1	*Danio rerio*	gi|41388972	Pgk1	0.001	−3.37
2044	E3	L-lactate dehydrogenase B-B chain	*Danio rerio*	gi|50540008	LDHBB	0.010	−2.95
1432	F19	Sorbitol dehydrogenase	*Salmo salar*	gi|213514212	Sord	7.320e–5	−18.68
**Energy metabolism**							
1823	F1	glyceraldehyde 3-phosphate dehydrogenase	*Gobiocypris rarus*	gi|298201228	GAPDH	0.001	−4.35
1546	F4	fructose-bisphosphate aldolase B	*Oryzias latipes*	gi|46849373	ALDOB	0.025	−5.06
1600	F5	fructose 1,6-bisphosphate aldolase B	*Cynoscion*	gi|77380115	ALDOB	0.004	2.83
1245	F21	PREDICTED: fructose-bisphosphate aldolase B-like	*Oncorhynchus mykiss*	gi|348514442	ALDOB	0.022	−4.95
1240	F23	PREDICTED: fructose-bisphosphate aldolase B-like	*Oncorhynchus mykiss*	gi|348514442	ALDOB	0.001	−2.19
**Chaperone**							
1636	F9	HSP70 protein	*Poecilia reticulata*	gi|157679184	HSP70	0.005	4.87
**Signal transduction**							
1793	E15	apolipoprotein A-I	*M. amblycephala*	gi|319429949	ApoA1	0.008	3.64
**Antioxidant proteins**							
1795	E11	glutathione S-transferase mu	*Cyprinus carpio*	gi|112901117	GSTm	0.039	5.86
1889	E24	glutathione peroxidase 1	*Danio rerio*	gi|169403976	GPx1	0.006	4.29
**Cofactors and vitamins**							
700	D8	betaine--homocysteine S-methyltransferase 1	*Danio rerio*	gi|60279651	Bhmt1	0.003	−2.40
**Amino acid metabolism**							
1199	C17	aspartate aminotransferase, cytoplasmic	*Danio rerio*	gi|47085773	AST	1.860e–5	−3.58
**Nucleotide metabolism**							
1591	F8	eukaryotic translation elongation factor 2b	*Danio rerio*	gi|41386743	EEF2b	6.77e–4	−2.81
1595	G10	eukaryotic translation elongation factor 2b	*Danio rerio*	gi|41386743	EEF2b	7.19e–4	−4.53

Liver proteins were differentially expressed in the 30 mg kg^−1^ versus the control emodin groups as described in the Methods section and identified by 2-DE and MS/MS, with relative expression ratios.

**Table 2:** Primer sequences for RT-PCR analysis of antioxidant related genes.

Genes	Primer sequences (5′ → 3′)	Product size (bp)	Amplification efficiency (%)
*β-actin*	(F) TCTGCTATGTGGCTCTTGACTTCG	132	94.27
	(R) CCTCTGGGCACCTGAACCTCT		
*ALDOB*	(F) AGGACAAGGGCATCGTAGTTGGTAT	137	103.89
	(R) GTCACAACCGTCCTTTTTGTACTGG		
*GAPDH*	(F) TGGTGCCAGTCAGAACATCA	196	98.97
	(R) TGCAGCCTTGACGACTTTCT		
*GPx1*	(F) GAACGCCCACCCTCTGTTTG	122	97.75
	(R) CGATGTCATTCCGGTTCACG		
*GSTm*	(F) AACCTCTGTGGGGAAACTGAAGAAG	150	92.53
	(R) TAGAGTTCCTGGCAGATTCTCATCG		
*HSP70*	(F) CGACGCCAACGGAATCCTAAAT	92	95.87
	(R) CTTTGCTCAGTCTGCCCTTGT		
*Pgk1*	(F) GCCTTTACCTTCCTCAAGGTTCTCA	150	96.79
	(R) GTCTGCAGTGATGAAGTCAACAGGA		
*Aldh8a1*	(F) ACATTCCACGATCAGCGTACAACTT	177	98.38
	(R) TCTTCCAGGTCAGGAGATACAGTGG		
*Sord*	(F) GAGCAGCATTCAAACAGCCATCTAT	104	98.94
	(R) CTGCTGCATTCAGAAGAGGTATGGT		

Primers for each gene were designed using primer premier 5.0. Amplification efficiency was determined according to RT-PCR analysis.
