# Supplementary material for: Comparative proteomic analysis of liver antioxidant mechanisms in Megalobrama amblycephala stimulated with dietary emodin
Source: Sci Rep. 2017 Jan 13;7:40356. doi: 10.1038/srep40356 (PMC5233964; doi:10.1038/srep40356)
Supplement: Supplementary Information [file srep40356-s1.pdf]

## Supplementary Information

# Comparative proteomic analysis of liver antioxidant mechanisms in *Megalobrama amblycephala* stimulated with dietary emodin

Changyou Song[1], Bo Liu[1,2,*], Jun Xie[1,2], Xianping Ge[1,2***], Zhenxin Zhao[1], Yuanyuan Zhang[1], Huimin Zhang[1], Mingchun Ren[2], Qunlan Zhou[2], Linghong Miao[2], Pao Xu[1,2**], Yan Lin[2]

[1] *Wuxi Fisheries College, Nanjing Agricultural University, Wuxi, 214081, China*

[2] *Key Laboratory of Freshwater Fisheries and Germplasm Resources Utilization, Ministry of Agriculture, Freshwater Fisheries Research Center, Chinese Academy of Fishery Sciences, Wuxi, 214081, China*

Corresponding authors:

* Bo Liu, E-mail: liub@ffrc.cn. Tel: 86-510-85556101

** Pao Xu, E-mail: xup@ffrc.cn. Tel: 86-510-85557959

*** Xianping Ge, E-mail: gexp@ffrc.cn. Tel: 86-510-85557892

Present address of all authors: Wuxi Fisheries College, Nanjing Agricultural University, No.9 Shanshui East Road, Wuxi 214081 FFRC, PR China, CAFS.

## Contents of Supplementary materials

Supplementary material 1·······················Page 1  to Page 3

Supplementary material 2·······················Page 4  to Page 84

Supplementary material 3·······················Page 85 to Page 97

Supplementary material 4·······················Page 98 to Page 113

## Supplementary material 1

## Previous reports demonstrating 30 mg kg$^{-1}$ emodin as the optimum level of dietary supplementation

As reported in our previous study, 30 mg kg$^{-1}$ emodin was the optimum level of dietary supplementation to greatly enhance the growth performance, non-specific immunity activity, and disease resistance to *Aeromonas hydrophila*. Therefore, the 30 mg kg$^{-1}$ group was selected for further analysis of proteome alterations. The concrete evidences are shown in Table S1, Figure S1 and S2.

**Table S1**

| *Dietary emodin (mg /kg)* | *Initial body weight (g)* | *Final body weight (g)* | *WG[2] (%)* | *SGR[3] (%/d)* | *FCR[4]* | *Survival rate[5] (%)* |
|---|---|---|---|---|---|---|
| 0 | 3.5±0.01 | 11.54±1.14 | 229.51±3.38$^{a}$ | 2.13±0.02$^{a}$ | 1.78±0.06$^{a}$ | 98.67±1.33 |
| 15 | 3.51±0.02 | 12.04±0.15 | 245.9±5.1$^{b}$ | 2.21±0.03$^{b}$ | 1.69±0.12$^{a}$ | 100 |
| 30 | 3.44±0.05 | 11.95±0.2 | 245.36±1.11$^{b}$ | 2.22±0.08$^{b}$ | 1.58±0.04$^{a}$ | 94.67±5.33 |
| 60 | 3.49±0.02 | 11.84±1.08 | 240.26±1.08$^{ab}$ | 2.19±0.12$^{ab}$ | 1.84±0.09$^{a}$ | 100 |
| 120 | 3.49±0.01 | 10.95±0.3 | 214.13±4.25$^{c}$ | 2.04±0.05$^{c}$ | 2.18±0.07$^{b}$ | 100 |

**Table S1. Effects of dietary emodin on growth performance in *M. amblycephala***

As reported previously, 30 mg kg$^{-1}$ emodin significantly increased the weight gain (WG), specific growth rate (SGR), while significantly decreased the feed conversion ratio (FCR). In brief, 30 mg kg$^{-1}$ dietary emodin is a suitable supplementation that can greatly enhance the growth performance of *M. amblycephala*.

**Figure S1**

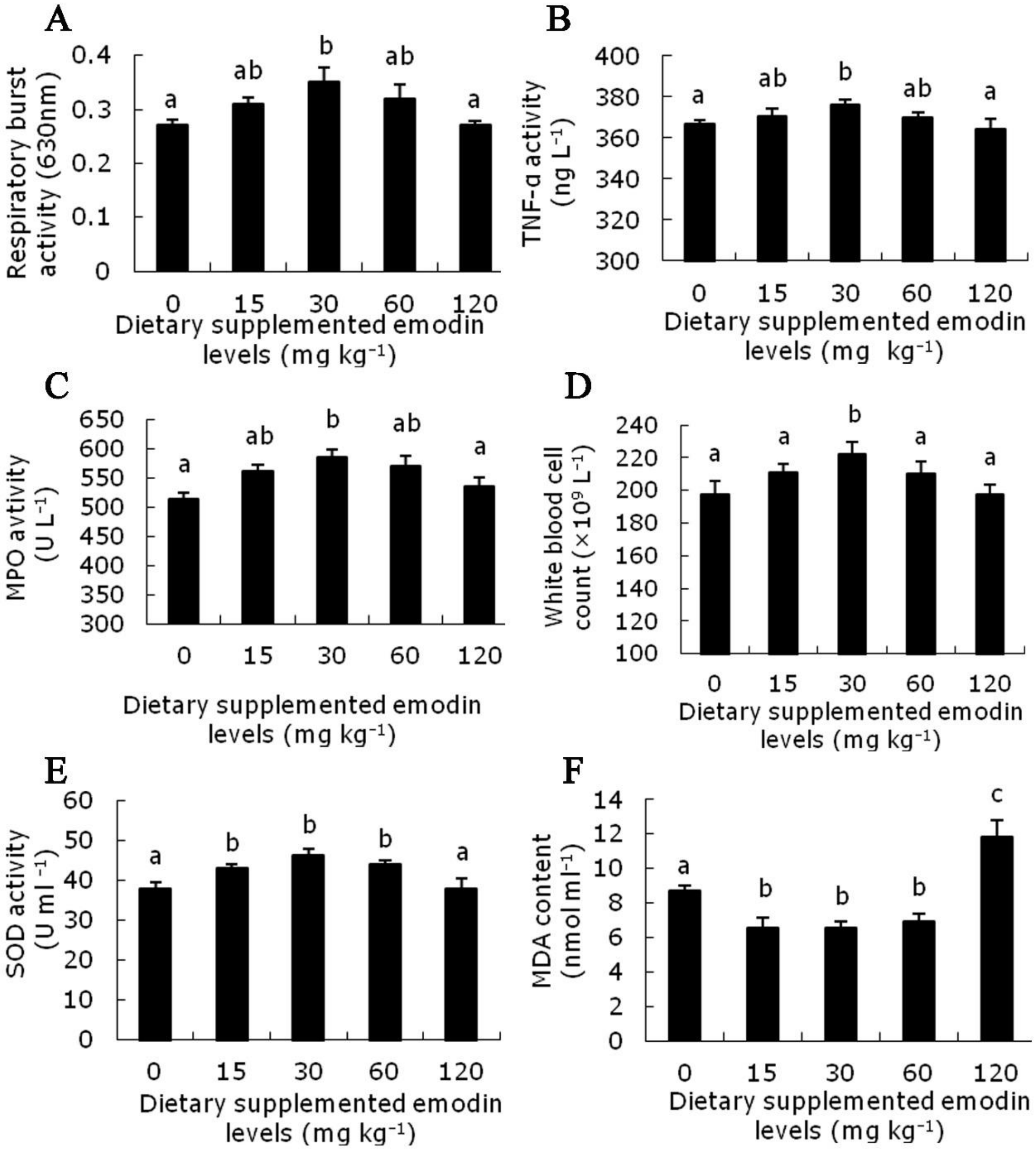


**Figure S1. Effects of dietary emodin on immune response in *M. amblycephala***

30 mg $kg^{-1}$ dietary emodin significantly increased the respiratory burst and TNF-α activity, plasma Leucocyte myeloperoxidase (MPO) activity, white blood cell (WBC), plasma superoxide dismutase (SOD) activity, and plasma malondialdehyde (MDA) content. Results indicate that the non-specific immunity as represented by the parameters tested was improved in *M. amblycephala* juvenile fed diet supplemented with 30 mg $kg^{-1}$ emodin.

**Figure S2**

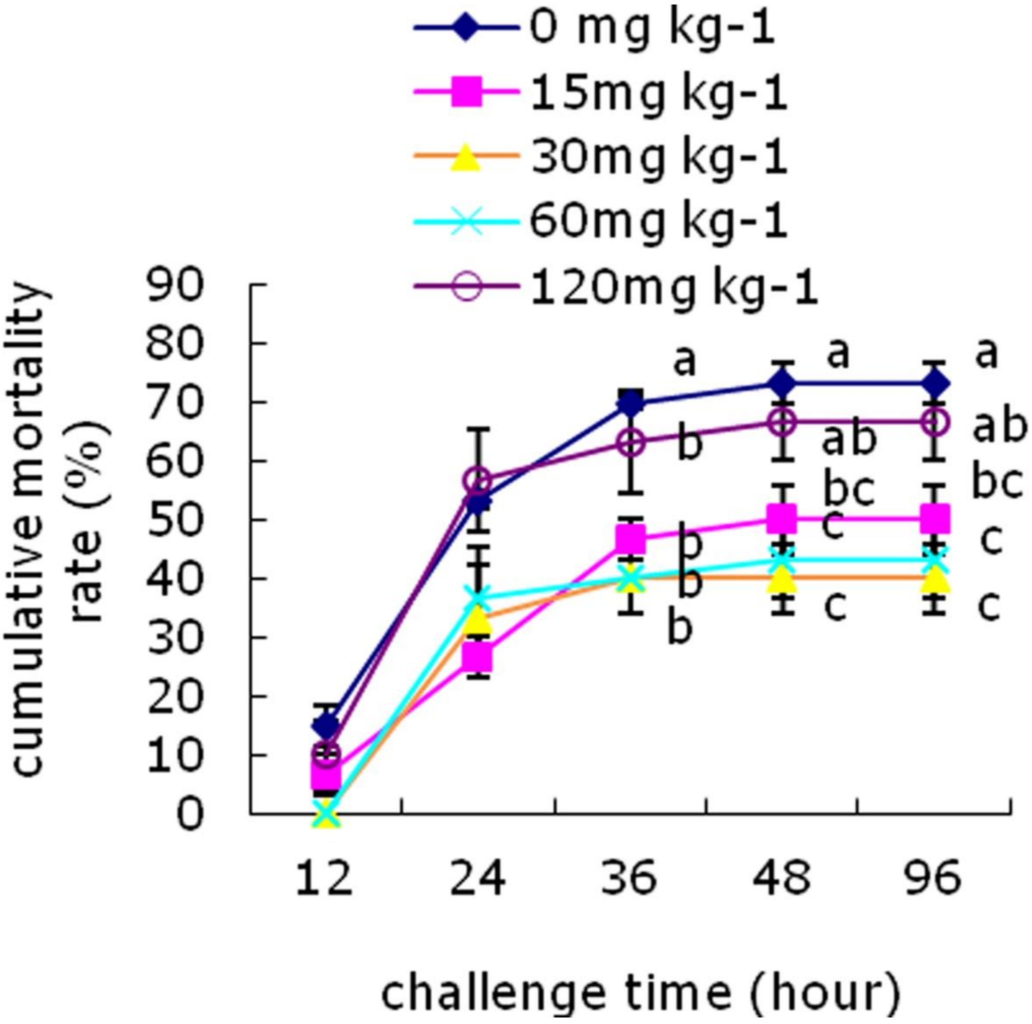


**Figure S2. Effects of dietary emodin on disease resistance to *Aeromonas hydrophila* in *M. amblycephala***

The cumulative mortality rate in 30 mg $kg^{-1}$ dietary emodin group injected with *Aeromonas hydrophila* ($5\times10^{6}$ CFU/mL) was significantly lower than that of the other groups. Results shown that improved non-specific immune response could increase resistance to pathogen infection in *M. amblycephala* fed with optimum emodin diet.

## Supplementary material 2

### The MALDI-TOF analysis results were shown with Target No. of altered proteins

*Applied Biosystems 4700 Proteomics Analyzer 7044*

4700 Reflector Spec #1 MC[BP = 1116.6, 45933]

C6_MS

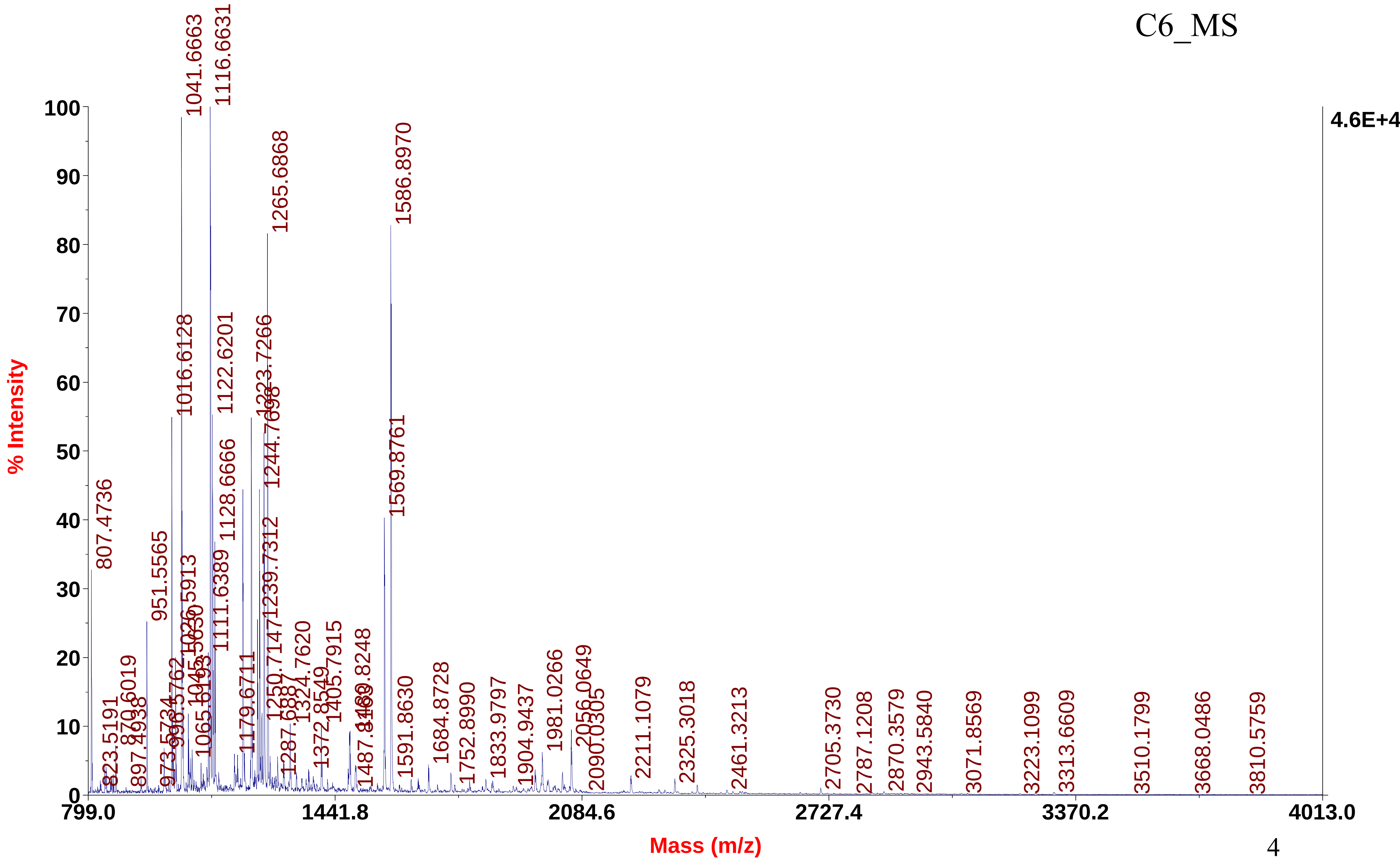

*Applied Biosystems 4700 Proteomics Analyzer 7044*

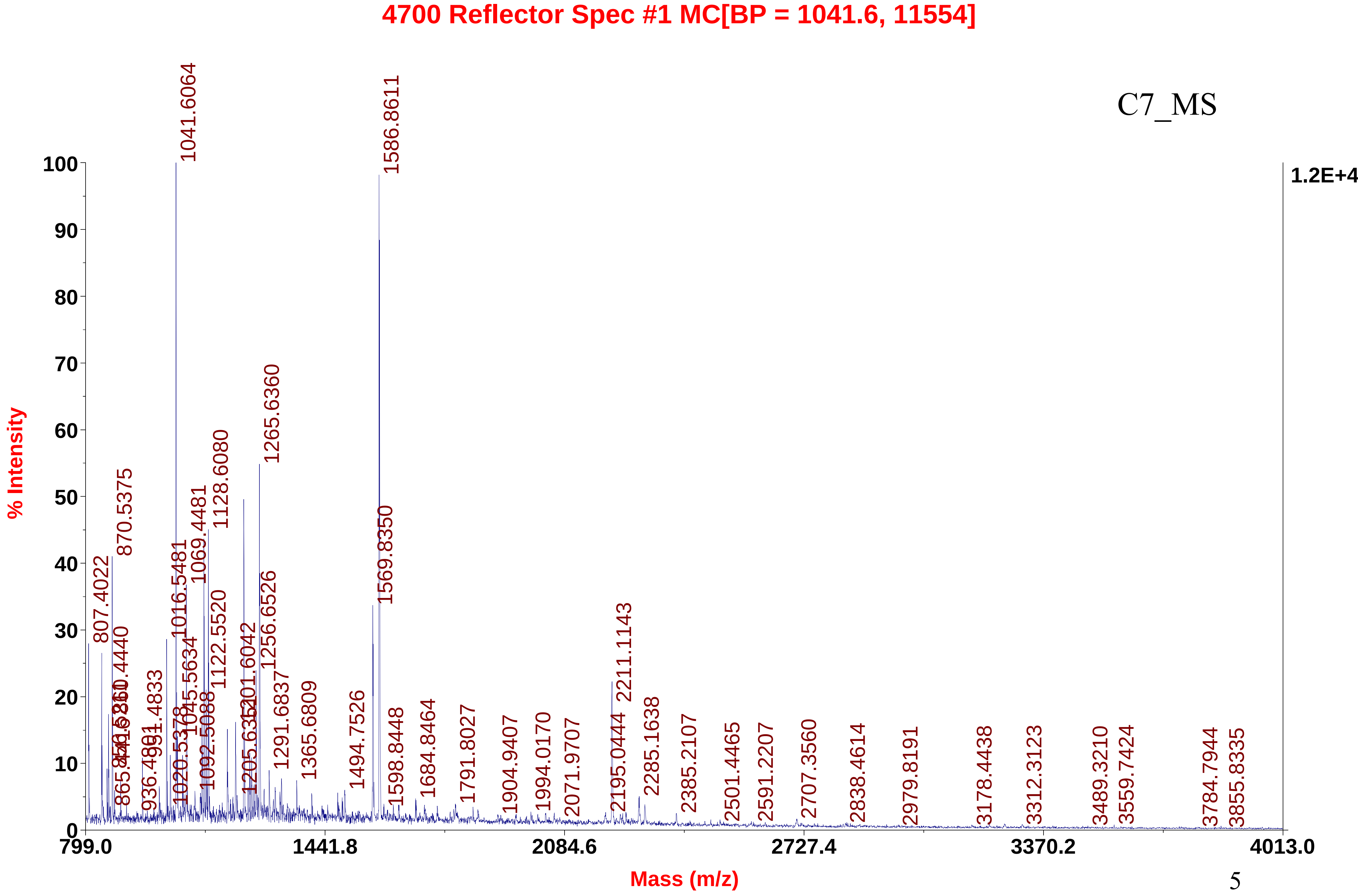

*Applied Biosystems 4700 Proteomics Analyzer 7044*

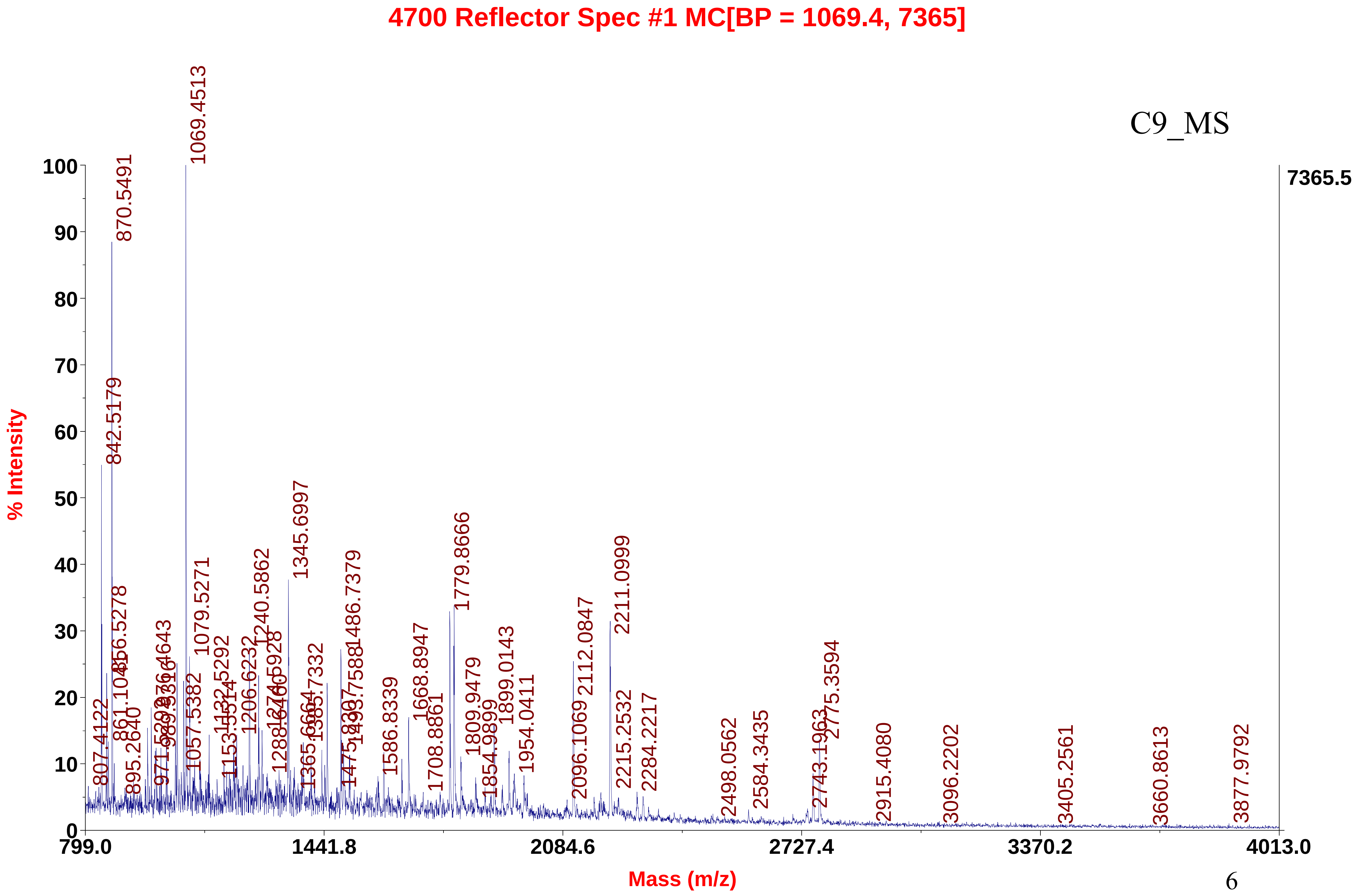

*Applied Biosystems 4700 Proteomics Analyzer 7044*

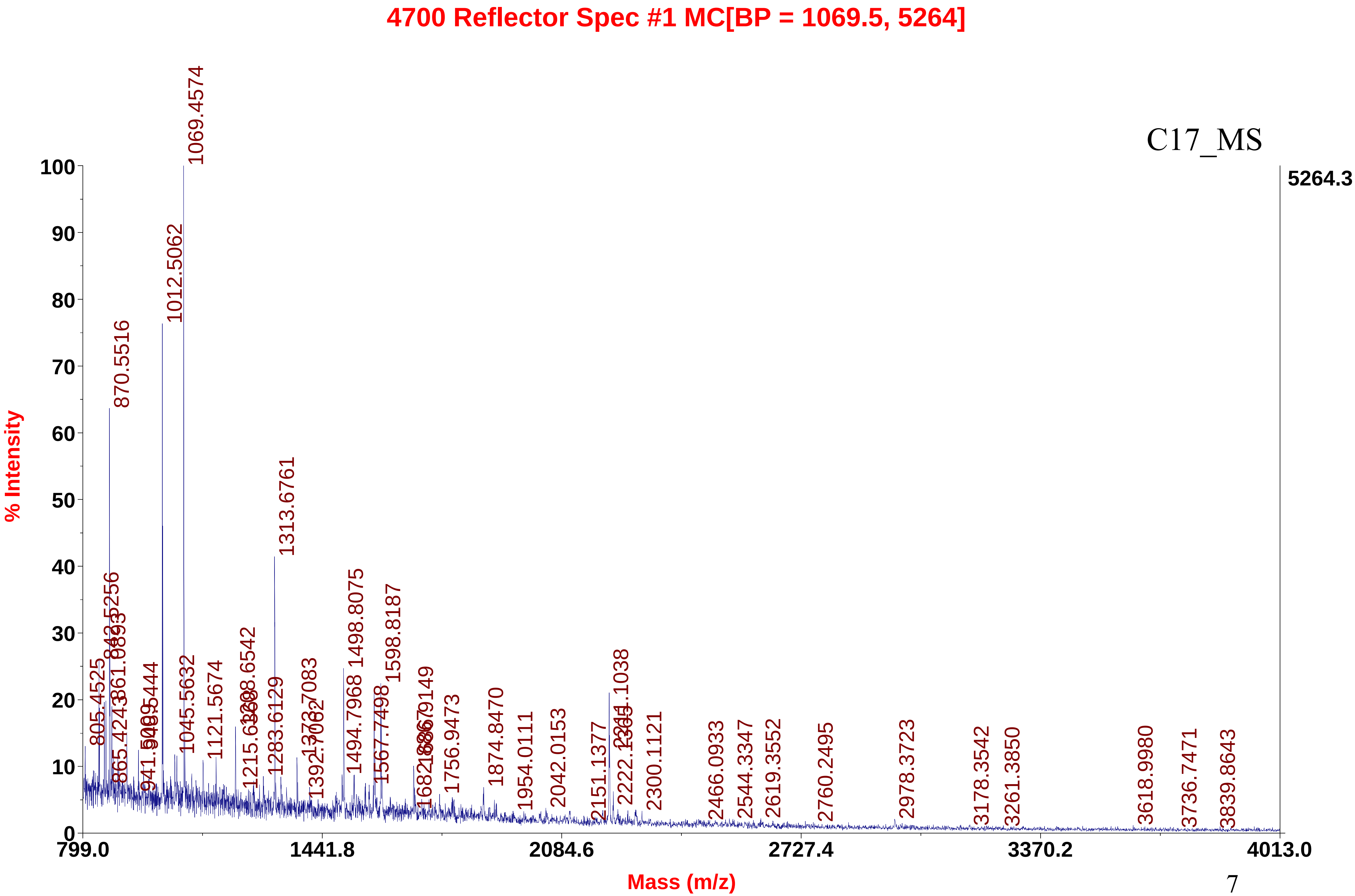

*Applied Biosystems 4700 Proteomics Analyzer 7044*

**4700 Reflector Spec #1 MC[BP = 1069.4, 8536]**

C18_MS

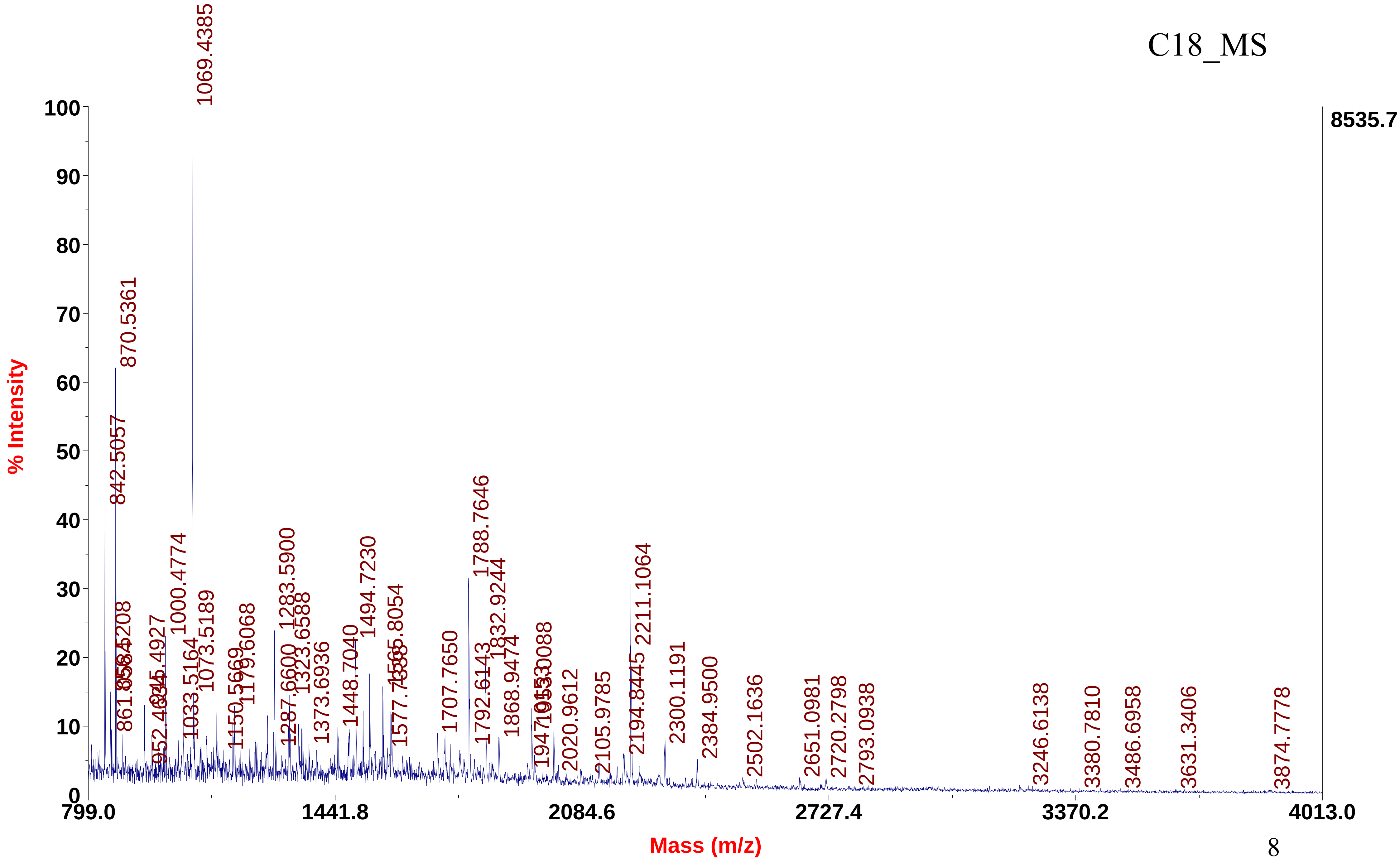

*Applied Biosystems 4700 Proteomics Analyzer 7044*

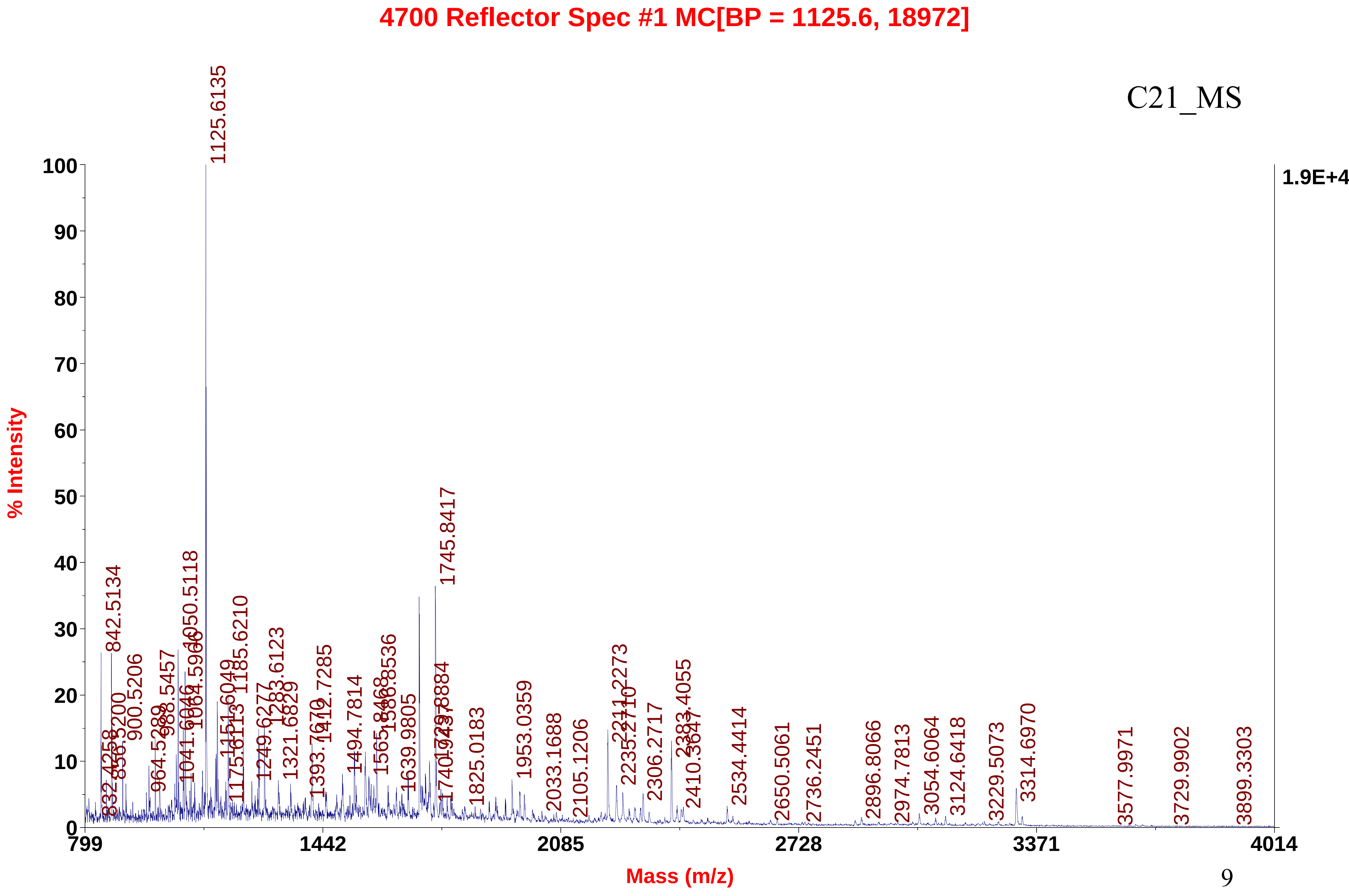

Applied Biosystems 4700 Proteomics Analyzer 7044

4700 Reflector Spec #1 MC[BP = 1069.5, 7045]

C22_MS

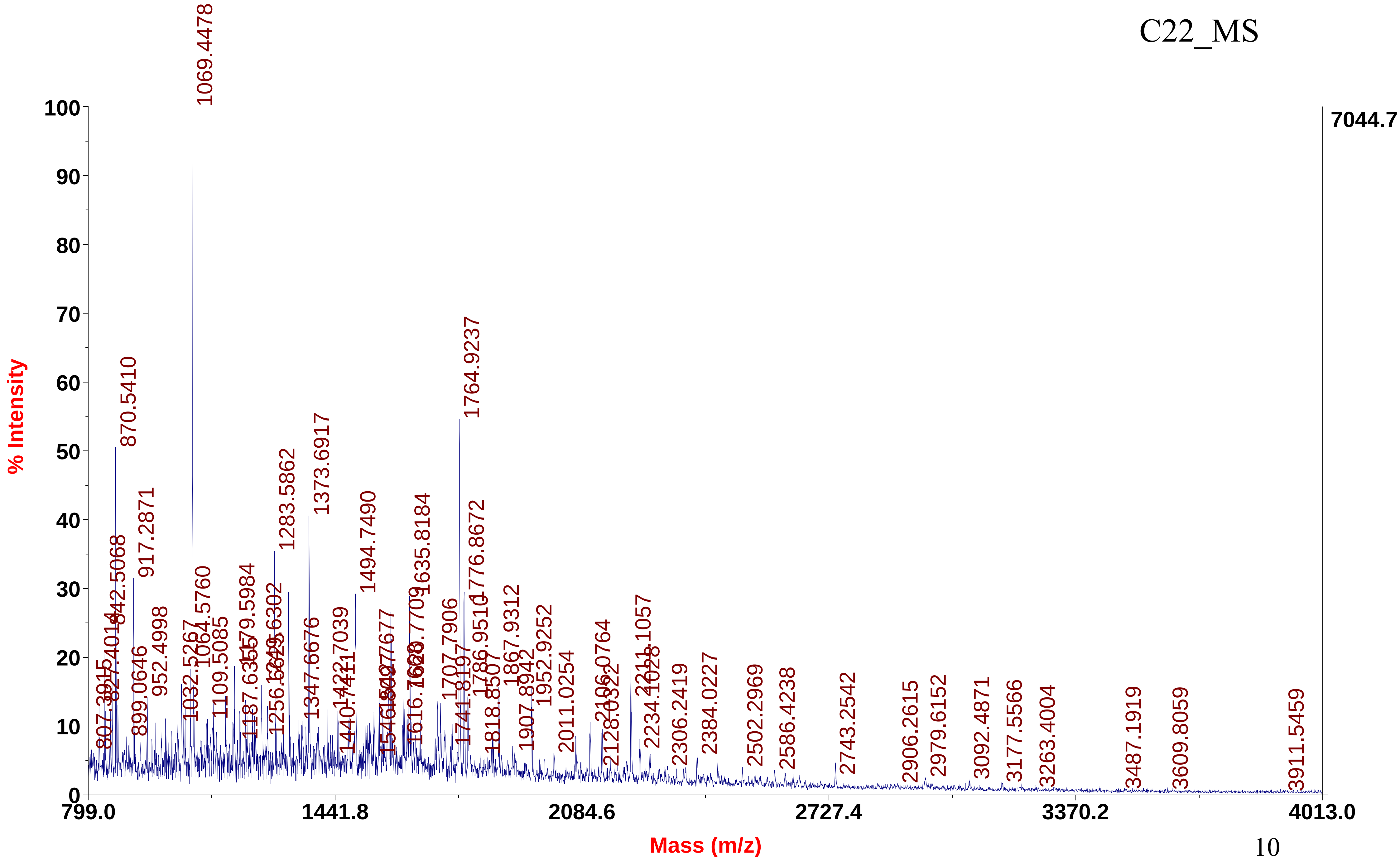

Applied Biosystems 4700 Proteomics Analyzer 7044

4700 Reflector Spec #1 MC[BP = 1069.4, 7187]

C23_MS

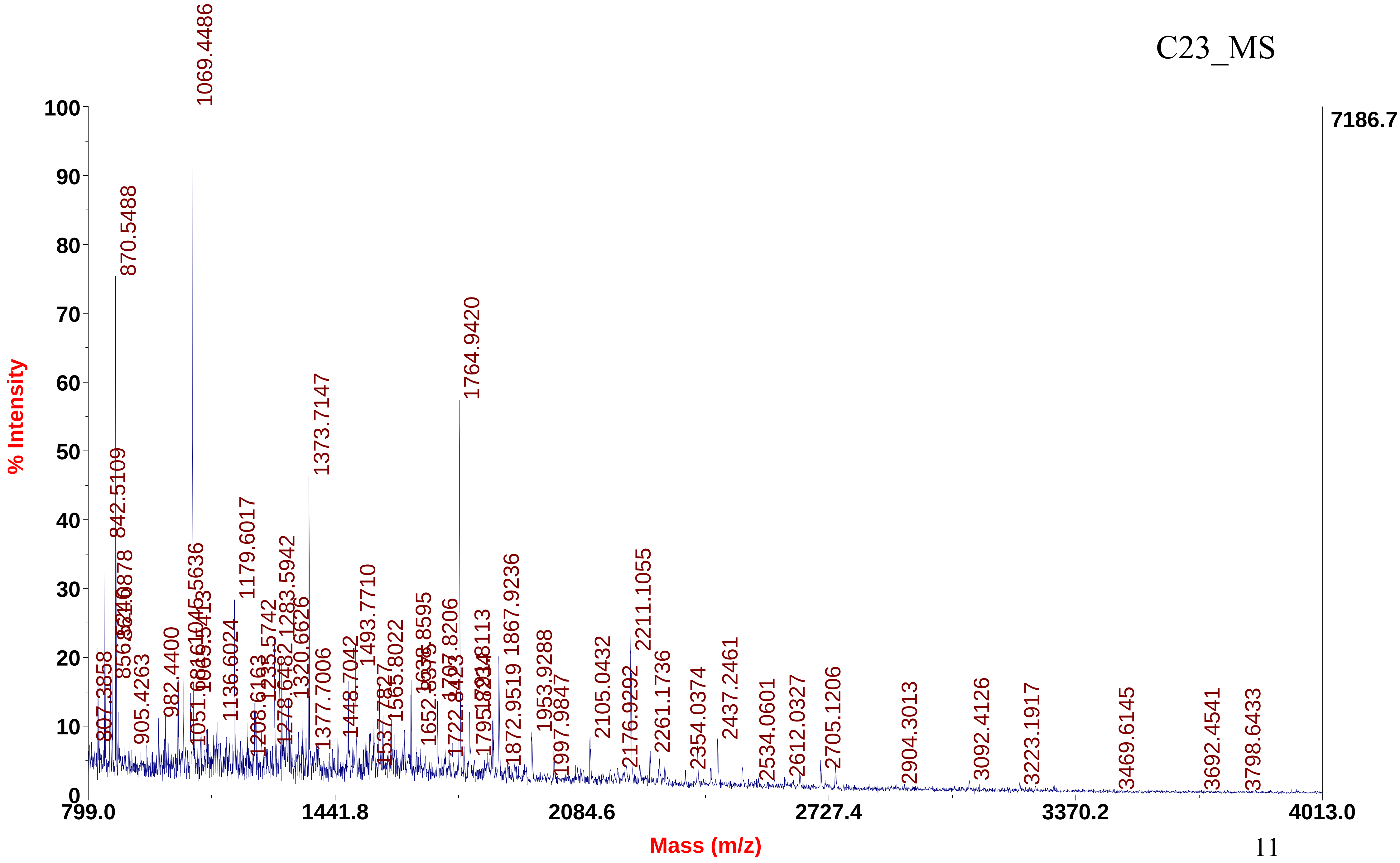

*Applied Biosystems 4700 Proteomics Analyzer 7044*

**4700 Reflector Spec #1 MC[BP = 1069.5, 7780]**

D1_MS

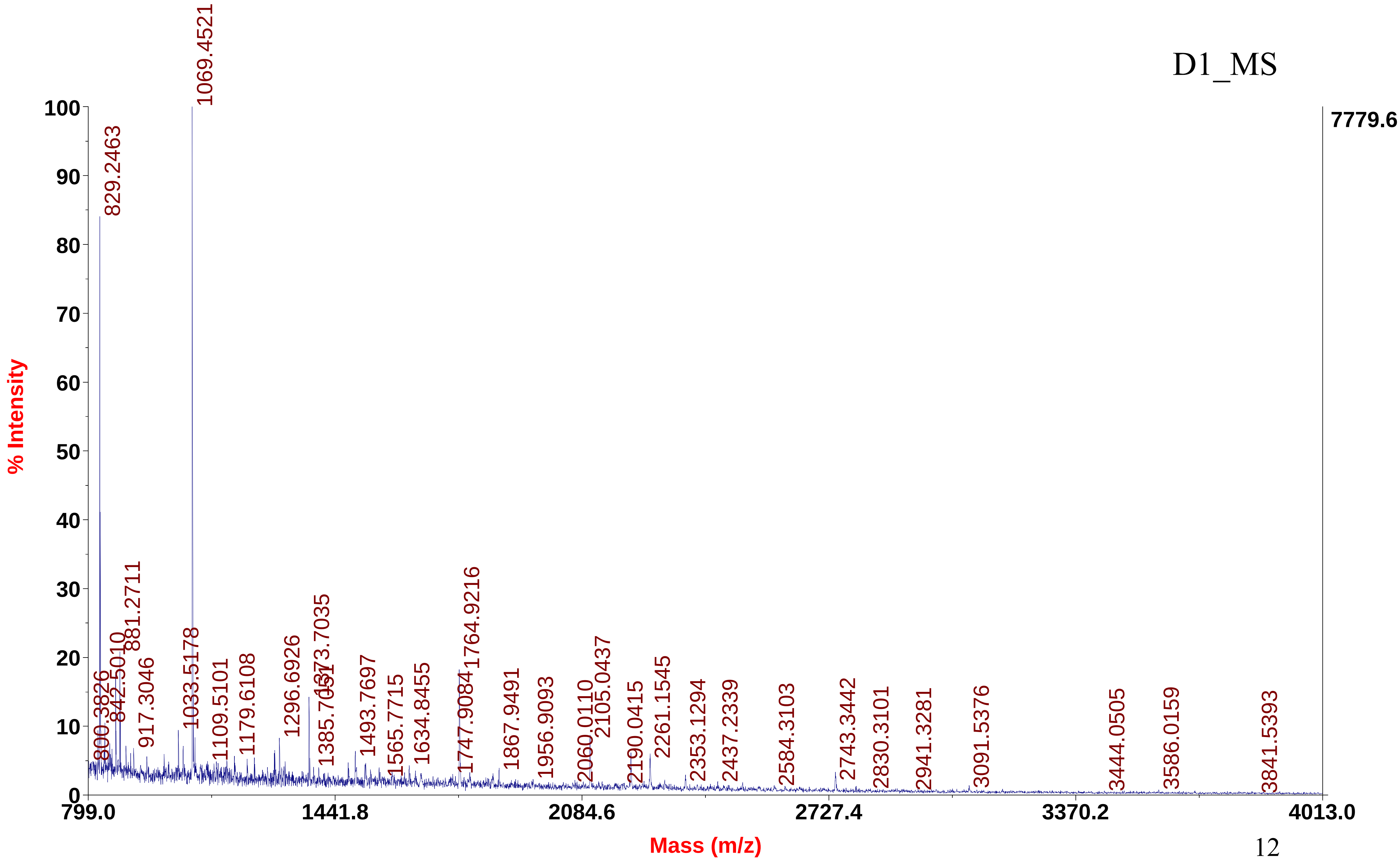

**4700 Reflector Spec #1 MC[BP = 1069.4, 5633]**

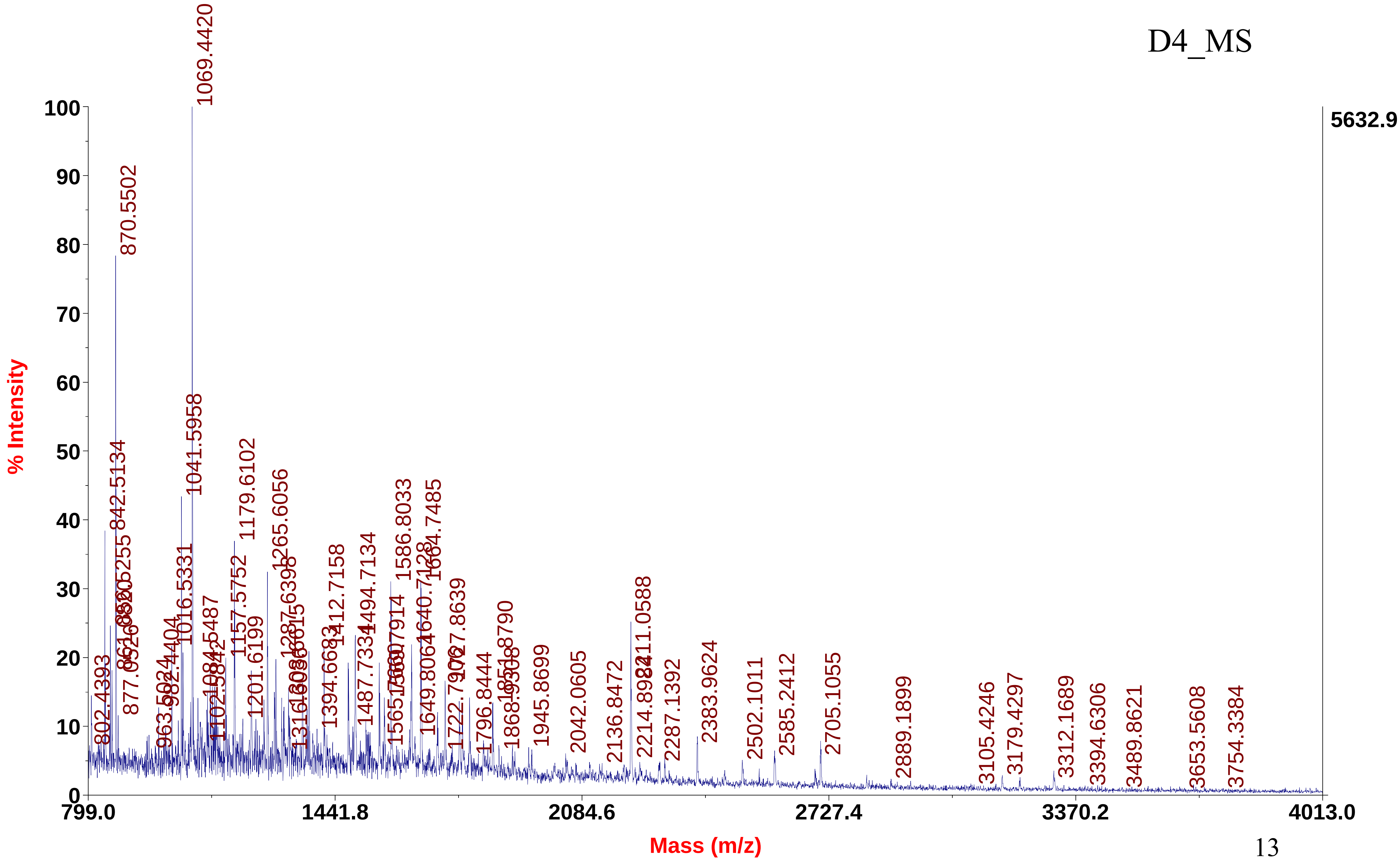

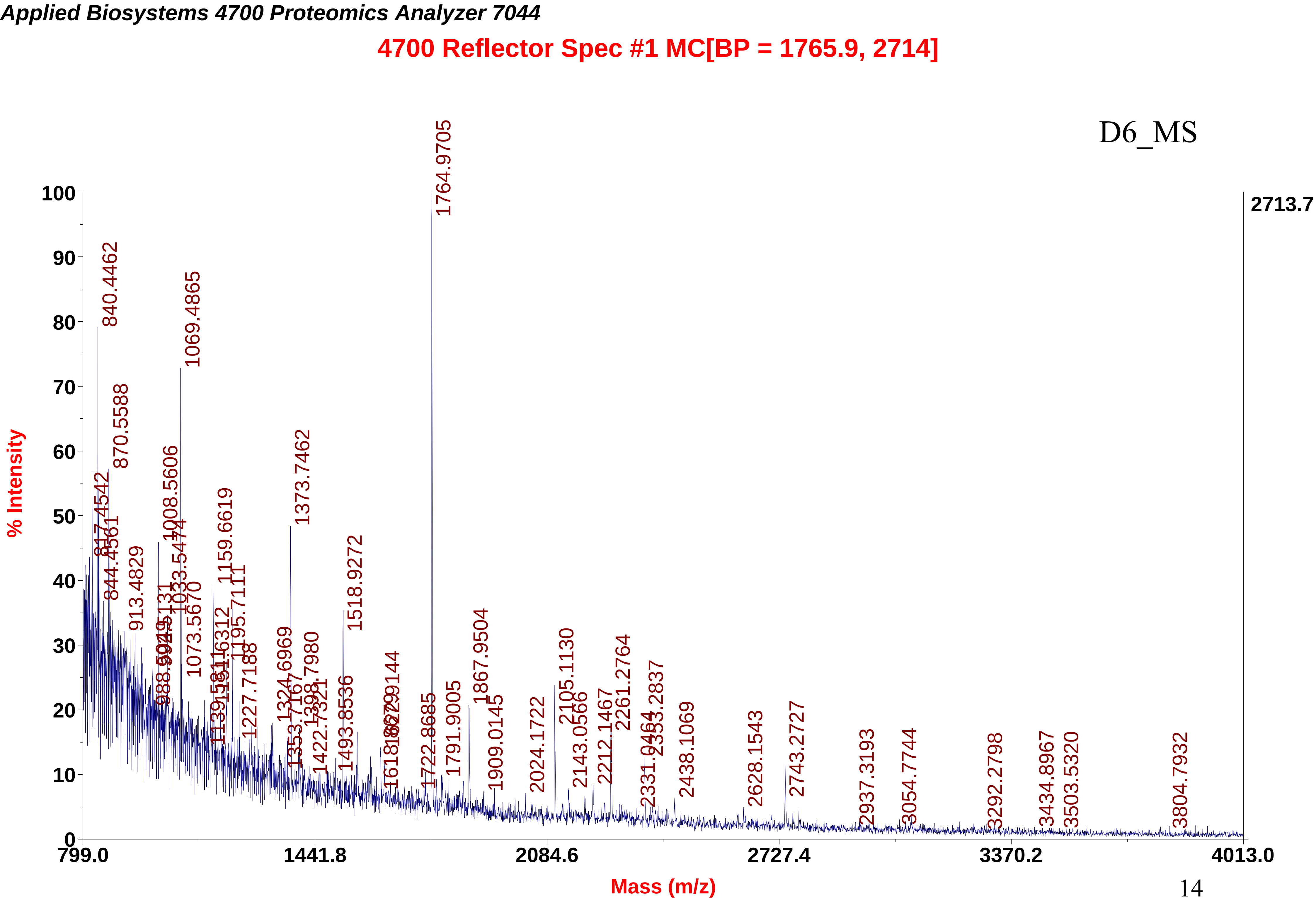
Applied Biosystems 4700 Proteomics Analyzer 7044
4700 Reflector Spec #1 MC[BP = 1765.9, 2714]
D6_MS
% Intensity
Mass (m/z)
2713.7
1764.9705
840.4462
1069.4865
870.5588
1373.7462
1008.5606
1159.6619
1518.9272
913.4829
1073.5670
1867.9504
2105.1130
2261.2764
2333.2837
2438.1069
2628.1543
2743.2727
2937.3193
3054.7744
3292.2798
3434.8967
3503.5320
3804.7932
1791.9005
1909.0145
2024.1722
2143.0566
2212.1467
1722.8685
1324.6969
1398.7980
1227.7188
1195.7111
1493.8536
1422.7321

*Applied Biosystems 4700 Proteomics Analyzer 7044*

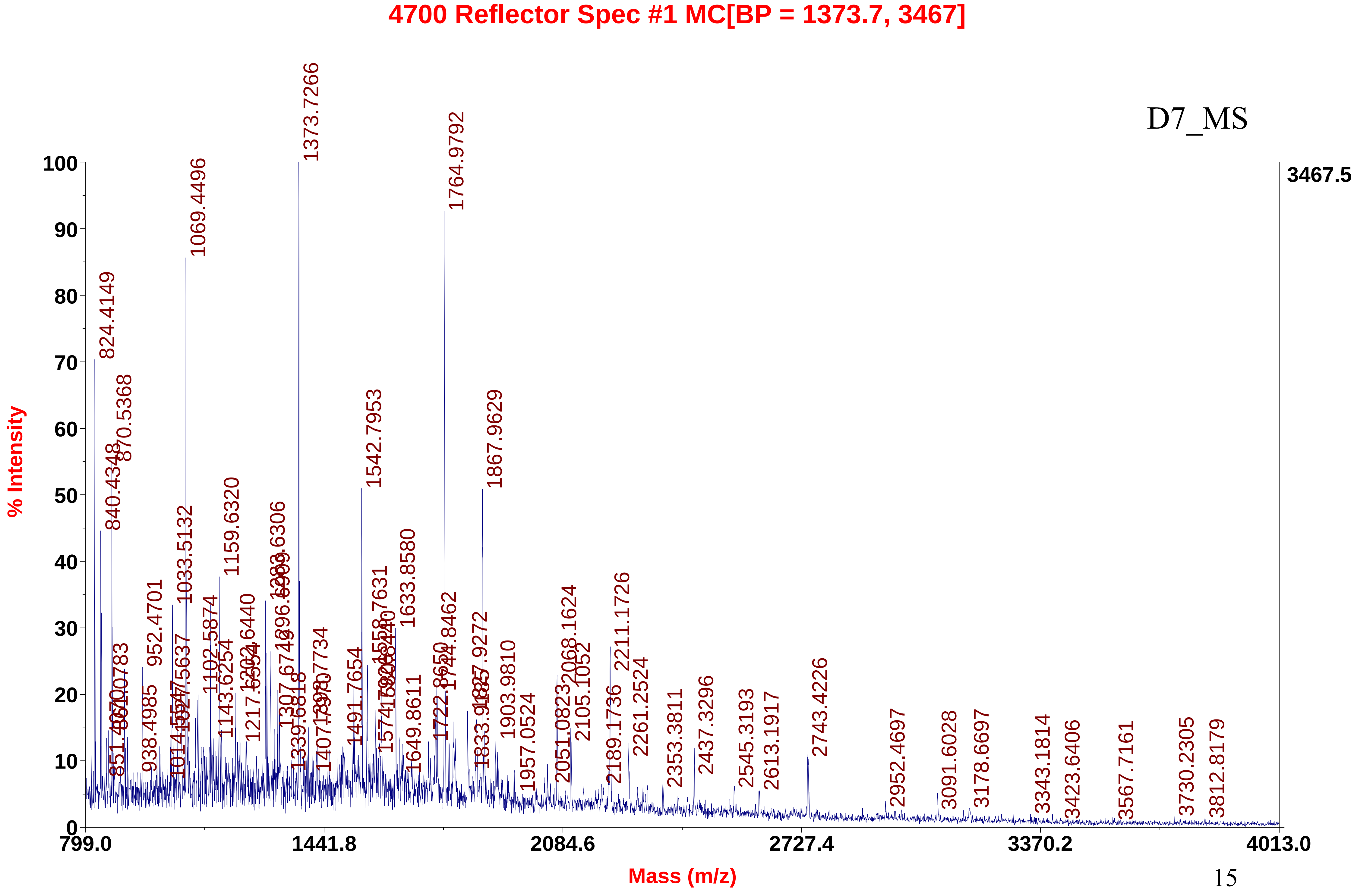

*Applied Biosystems 4700 Proteomics Analyzer 7044*

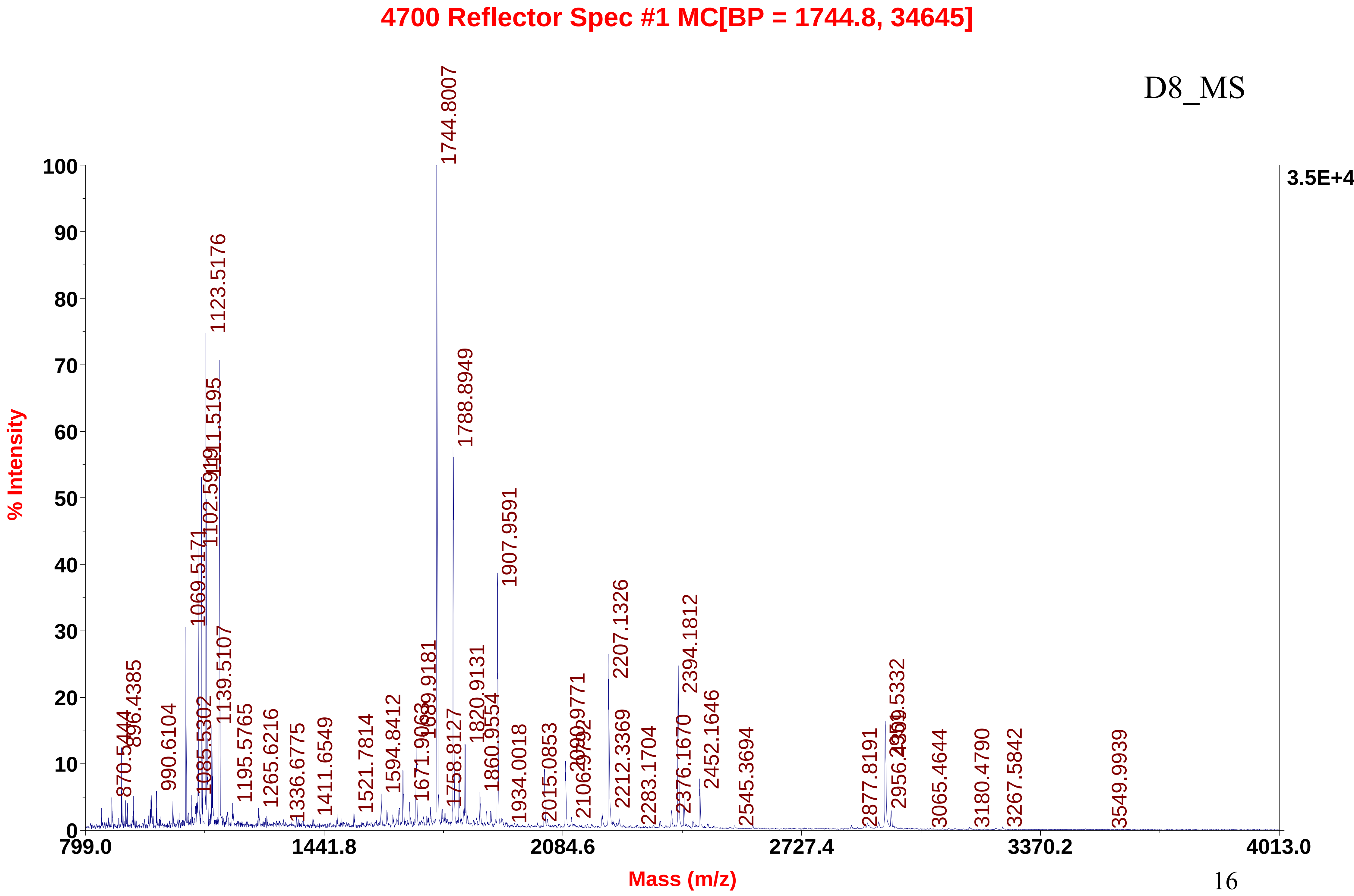

Applied Biosystems 4700 Proteomics Analyzer 7044

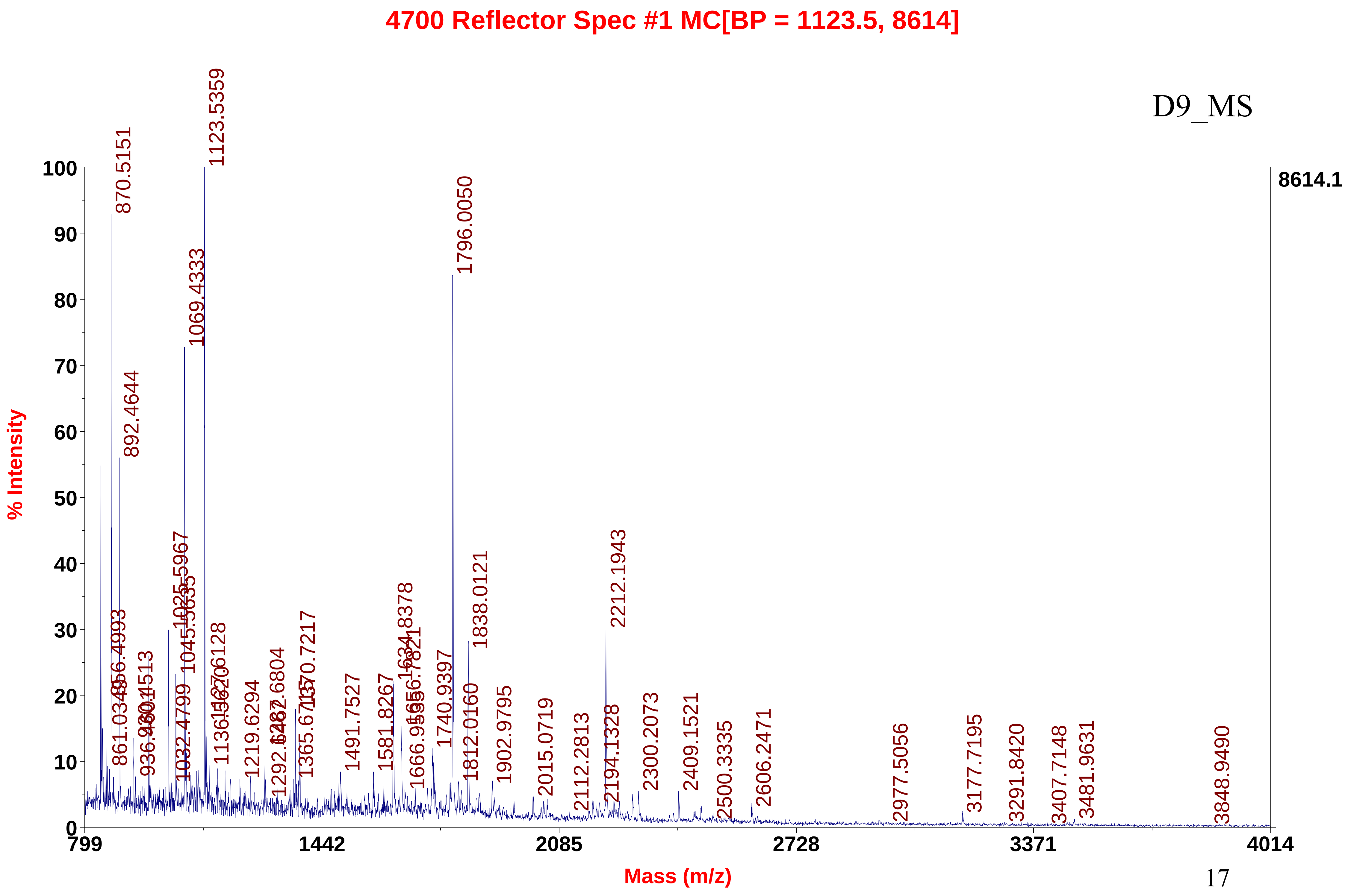

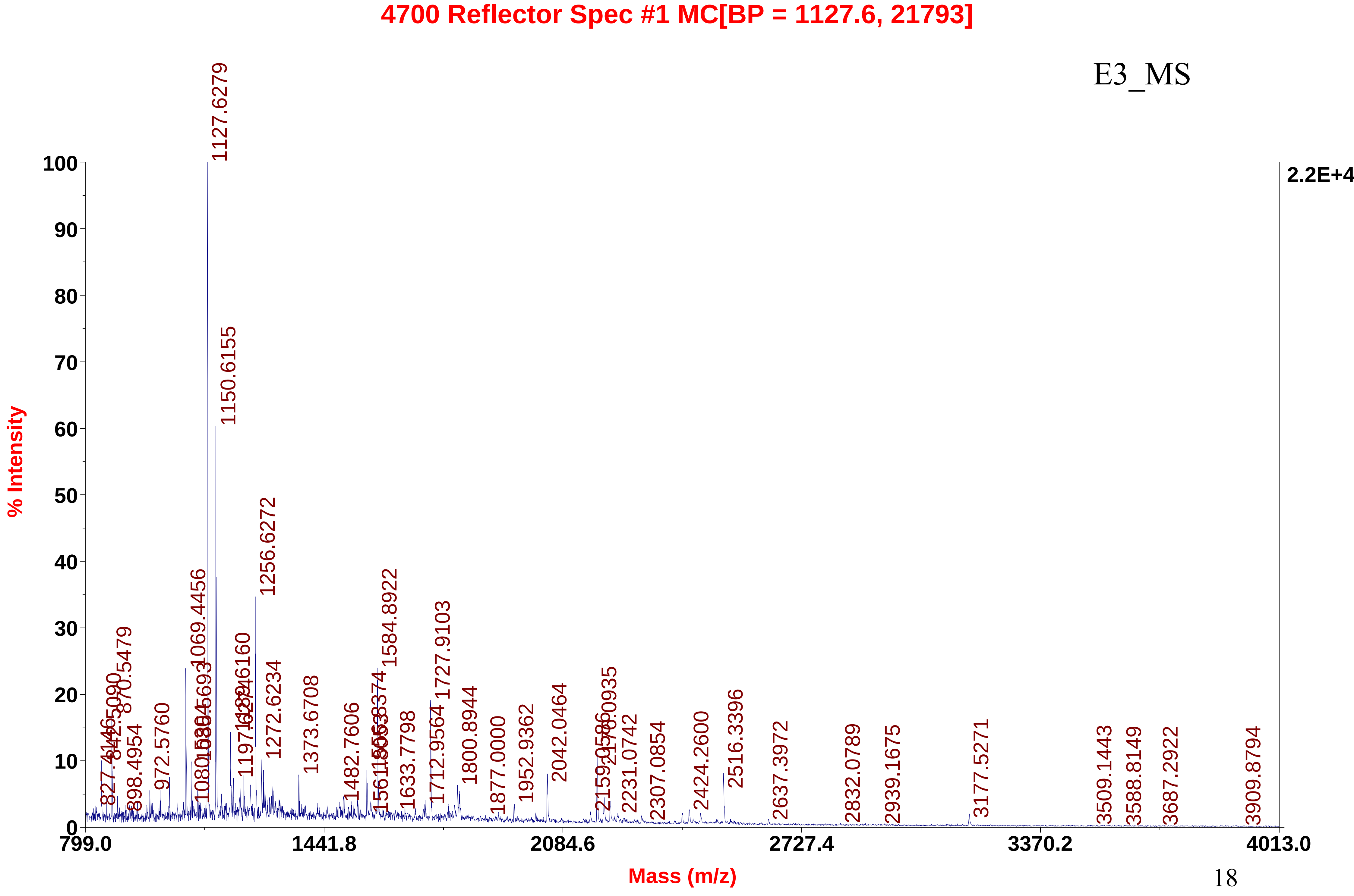
4700 Reflector Spec #1 MC[BP = 1127.6, 21793]
E3_MS
% Intensity
Mass (m/z)
2.2E+4
1127.6279
1150.6155
1256.6272
1069.4456
1584.8922
1727.9103
870.5479
842.5090
827.4146
898.4954
972.5760
1197.6160
1272.6234
1373.6708
1482.7606
1633.7798
1712.9564
1800.8944
1877.0000
1952.9362
2042.0464
2231.0742
2307.0854
2424.2600
2516.3396
2637.3972
2832.0789
2939.1675
3177.5271
3509.1443
3588.8149
3687.2922
3909.8794
799.0
1441.8
2084.6
2727.4
3370.2
4013.0

*Applied Biosystems 4700 Proteomics Analyzer 7044*

**4700 Reflector Spec #1 MC[BP = 936.5, 14840]**

E11_MS

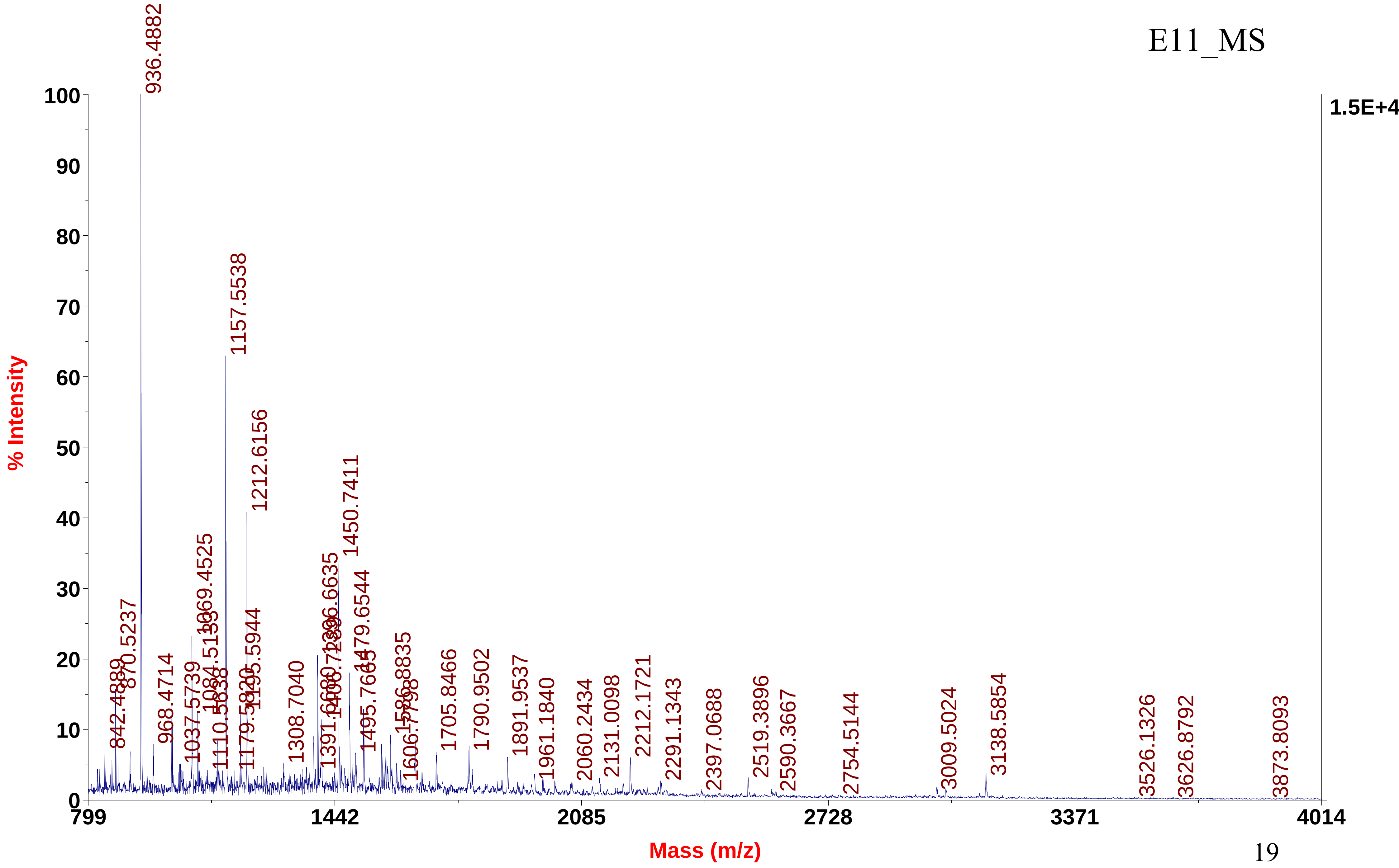

*Applied Biosystems 4700 Proteomics Analyzer 7044*

**4700 Reflector Spec #1 MC[BP = 1011.6, 32877]**

E15_MS

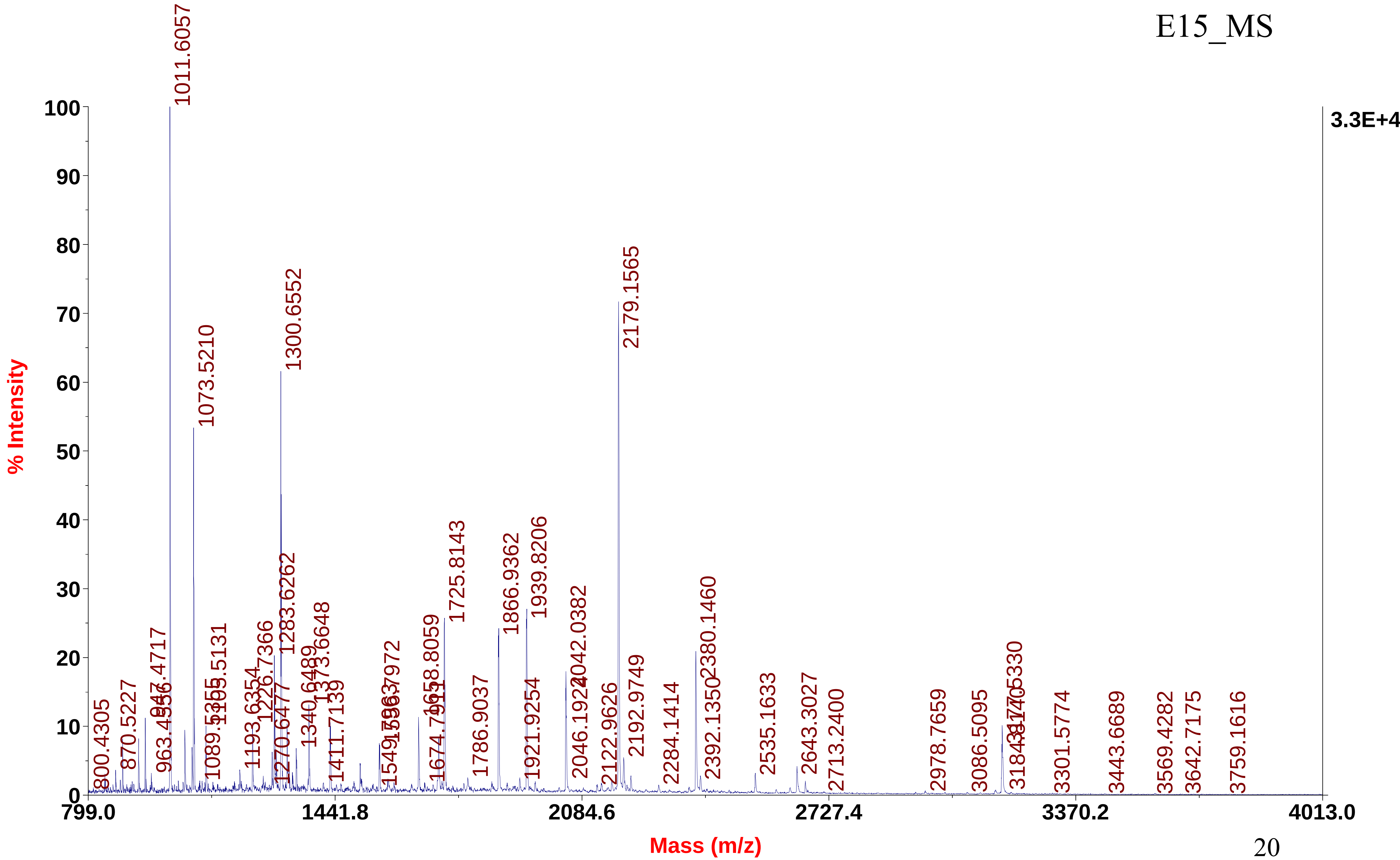

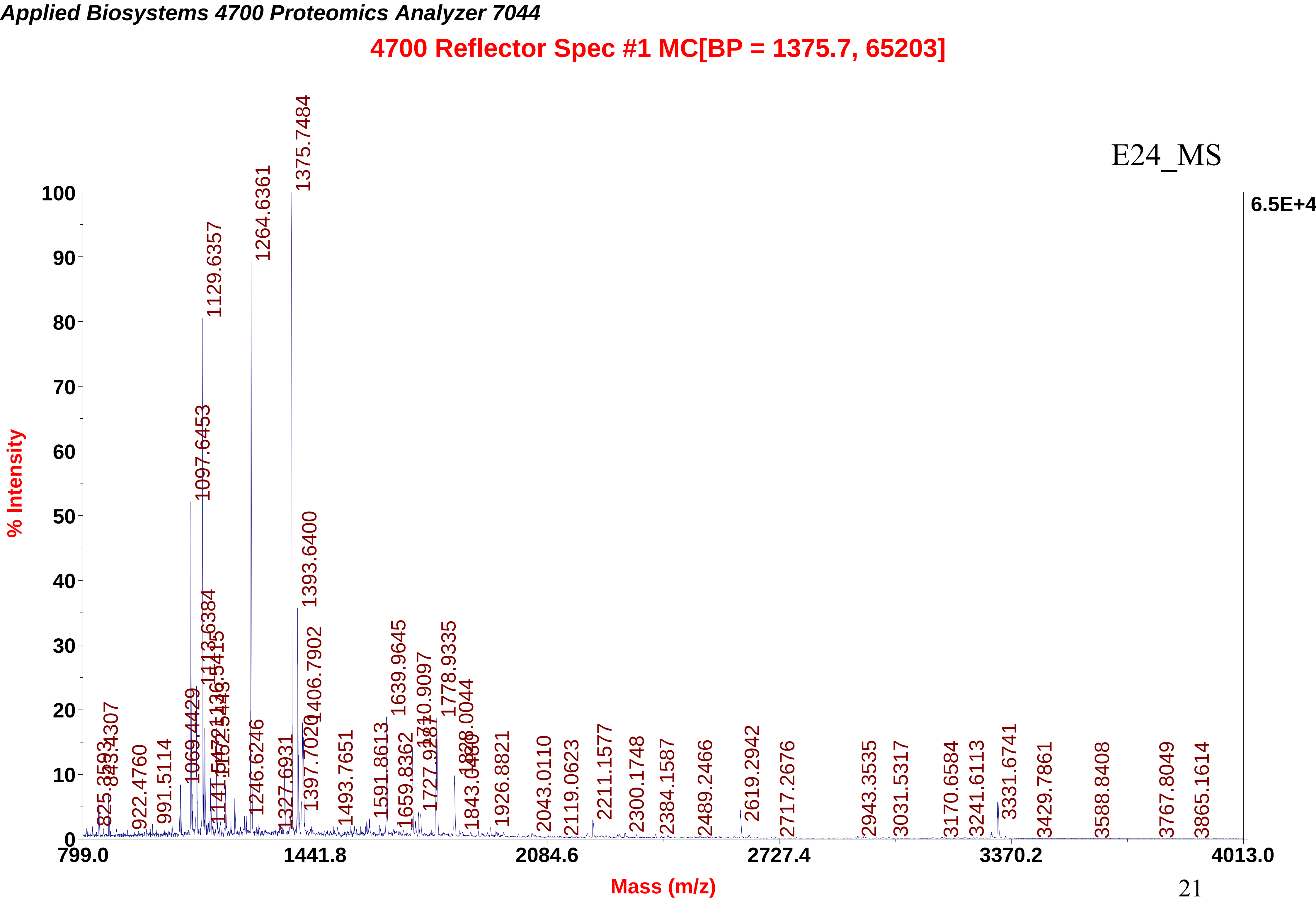
Applied Biosystems 4700 Proteomics Analyzer 7044
4700 Reflector Spec #1 MC[BP = 1375.7, 65203]
E24_MS
% Intensity
Mass (m/z)
6.5E+4
100
90
80
70
60
50
40
30
20
10
0
799.0
1441.8
2084.6
2727.4
3370.2
4013.0
843.4307
922.4760
991.5114
1069.4429
1097.6453
1113.6384
1129.6357
1246.6246
1264.6361
1327.6931
1375.7484
1393.6400
1397.7020
1406.7902
1493.7651
1591.8613
1639.9645
1659.8362
1710.9097
1778.9335
1828.0044
1926.8821
2043.0110
2119.0623
2211.1577
2300.1748
2384.1587
2489.2466
2619.2942
2717.2676
2943.3535
3031.5317
3170.6584
3241.6113
3331.6741
3429.7861
3588.8408
3767.8049
3865.1614

*Applied Biosystems 4700 Proteomics Analyzer 7044*

**4700 Reflector Spec #1 MC[BP = 1727.9, 7809]**

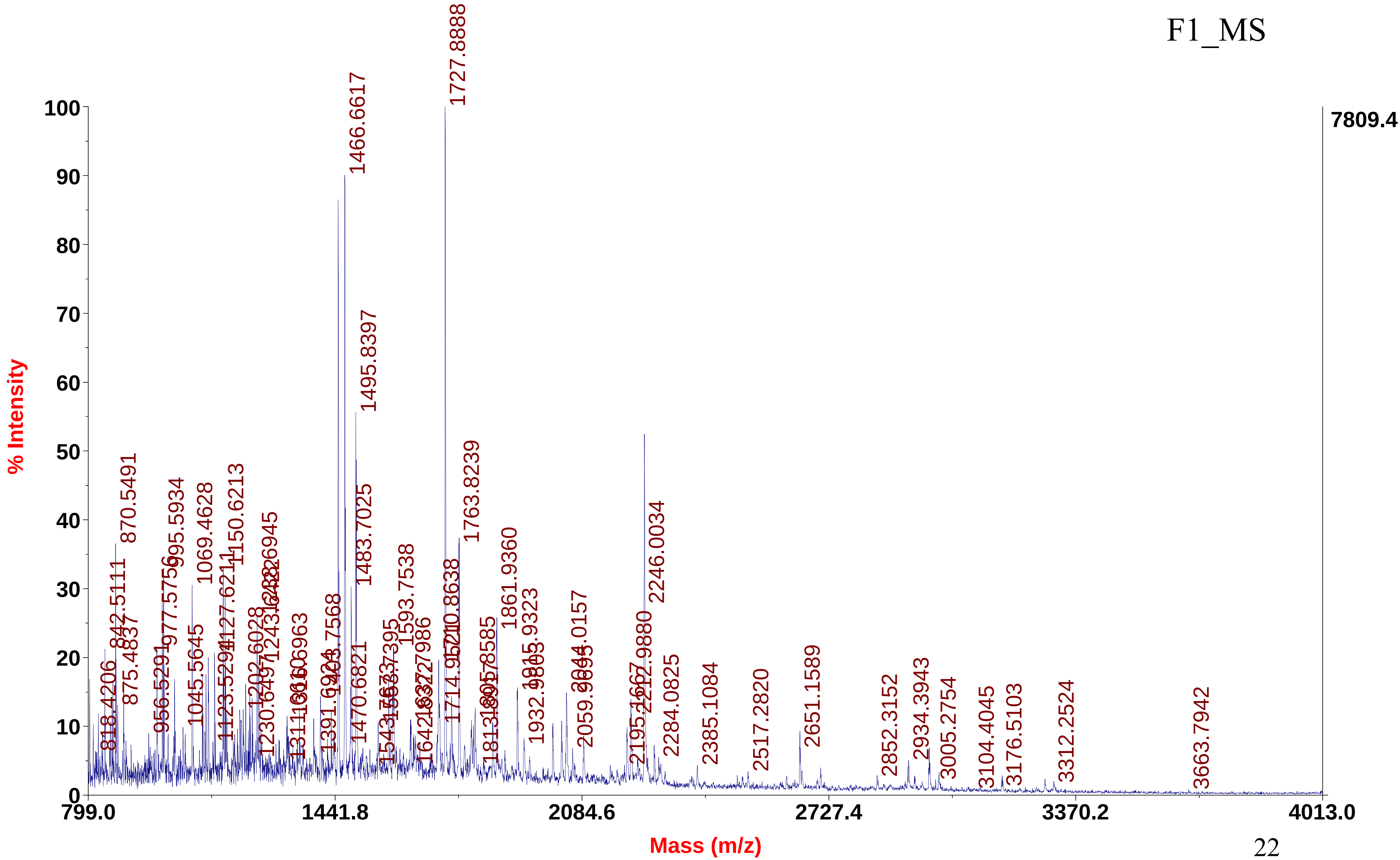

*Applied Biosystems 4700 Proteomics Analyzer 7044*

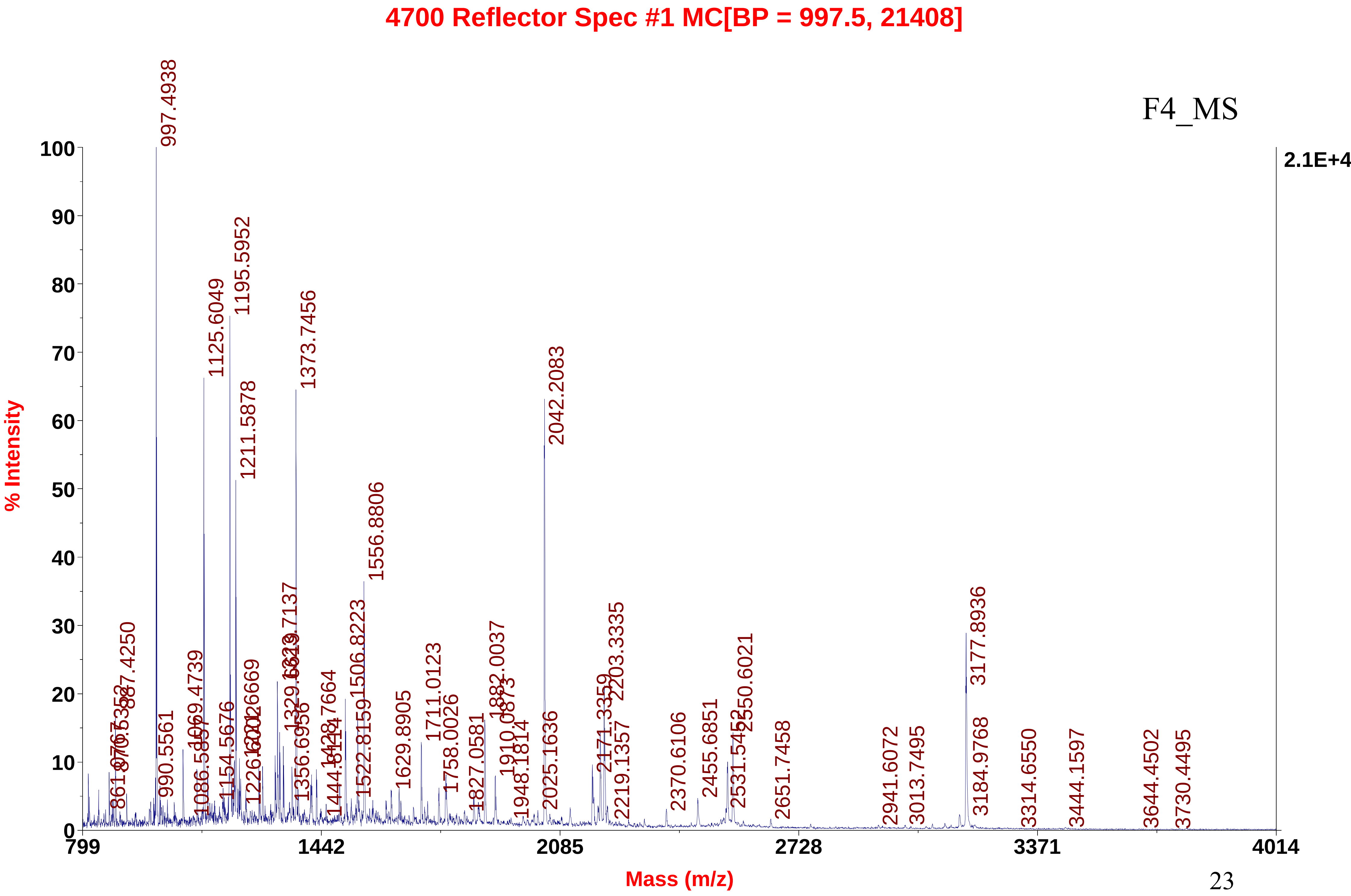

*Applied Biosystems 4700 Proteomics Analyzer 7044*

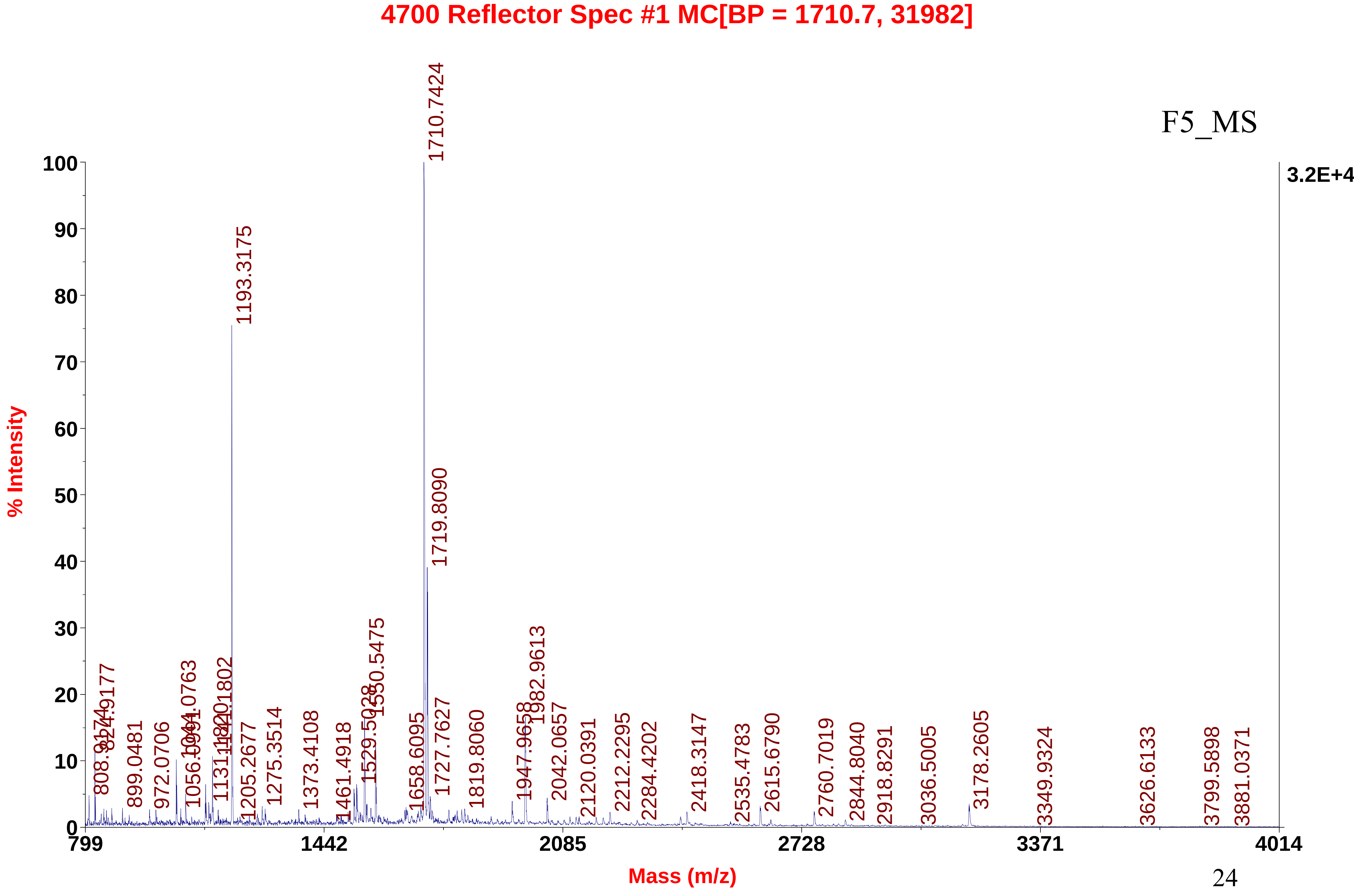

*Applied Biosystems 4700 Proteomics Analyzer 7044*

**4700 Reflector Spec #1 MC[BP = 997.5, 8259]**

F8_MS

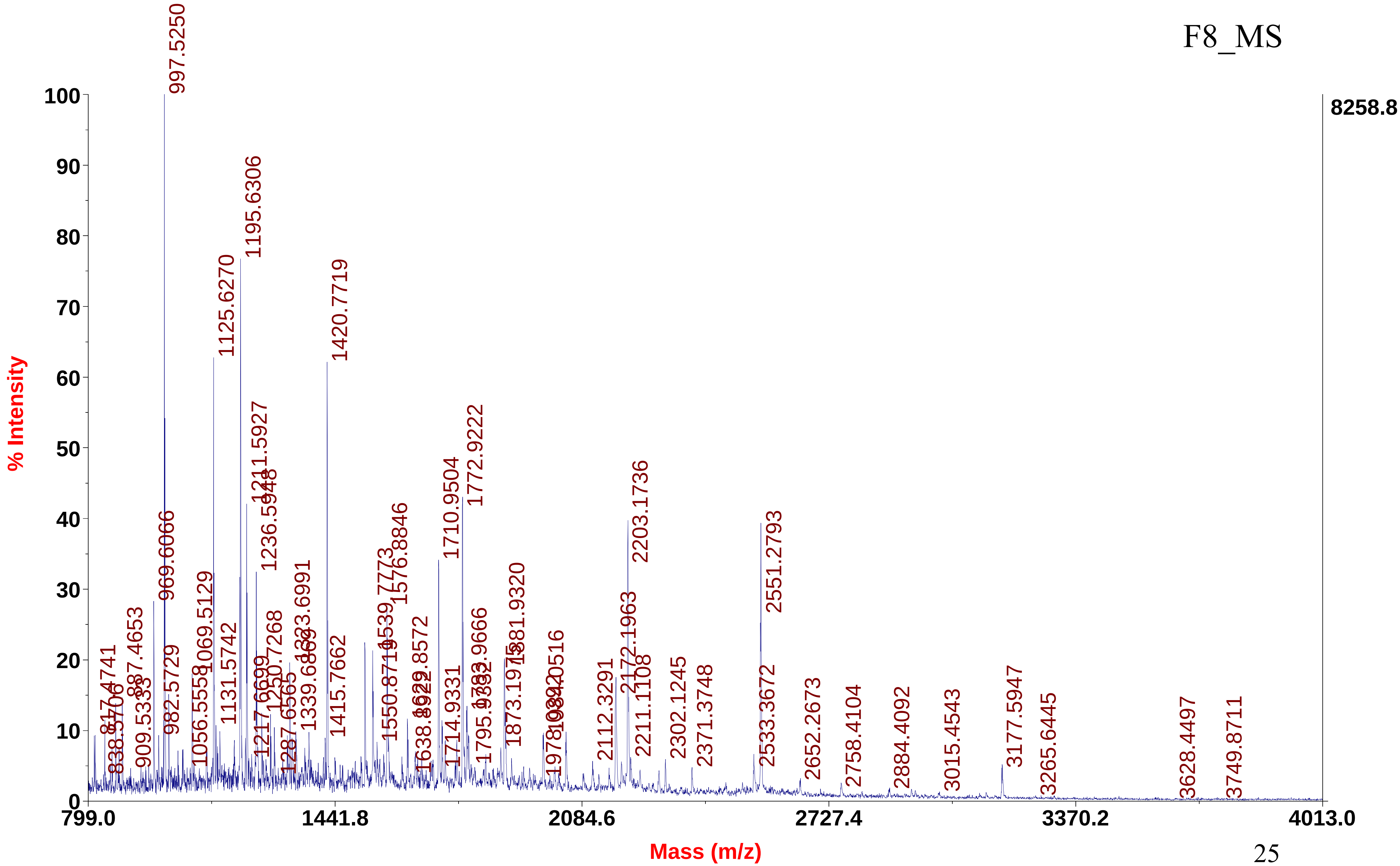

*Applied Biosystems 4700 Proteomics Analyzer 7044*

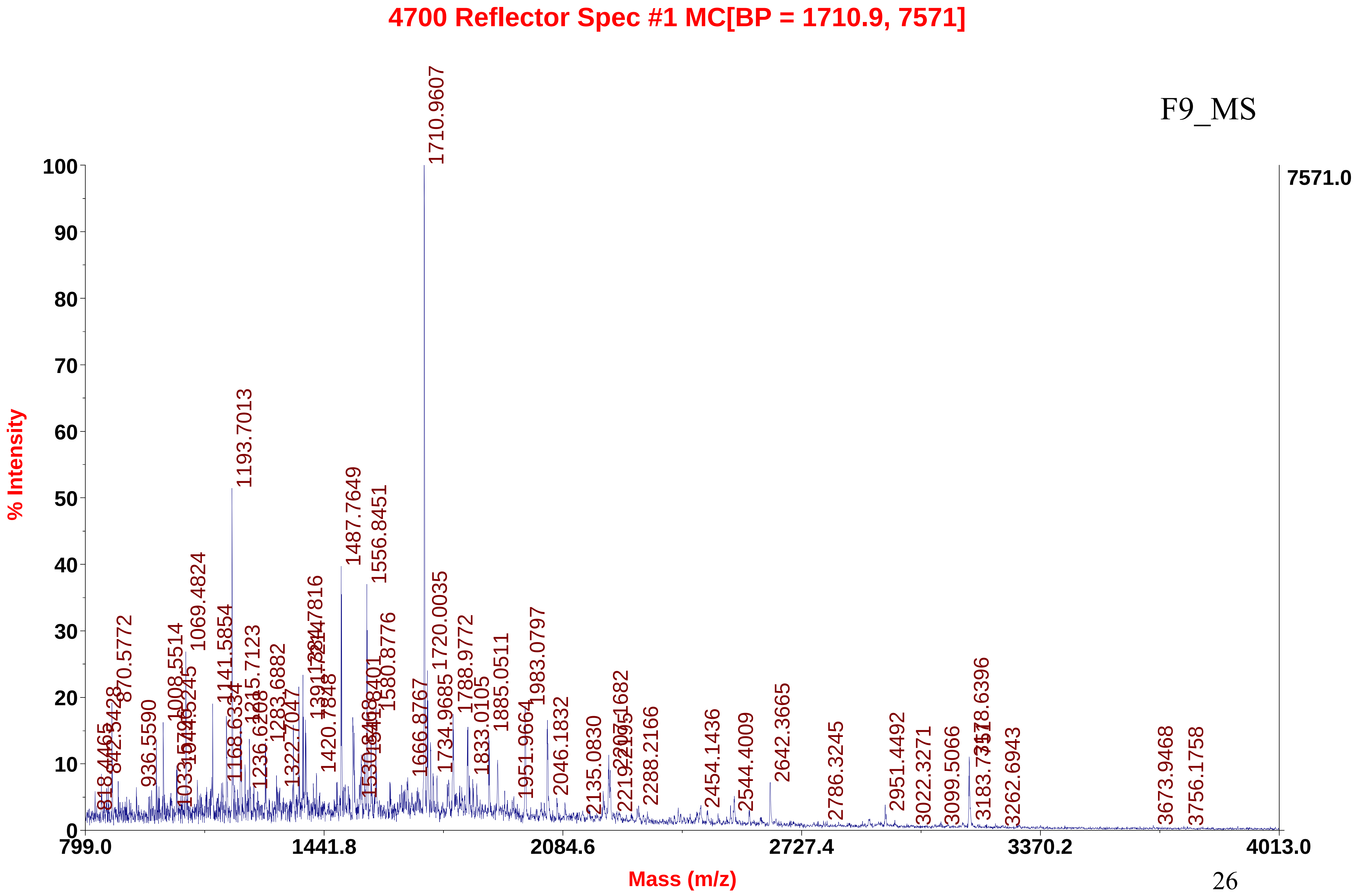

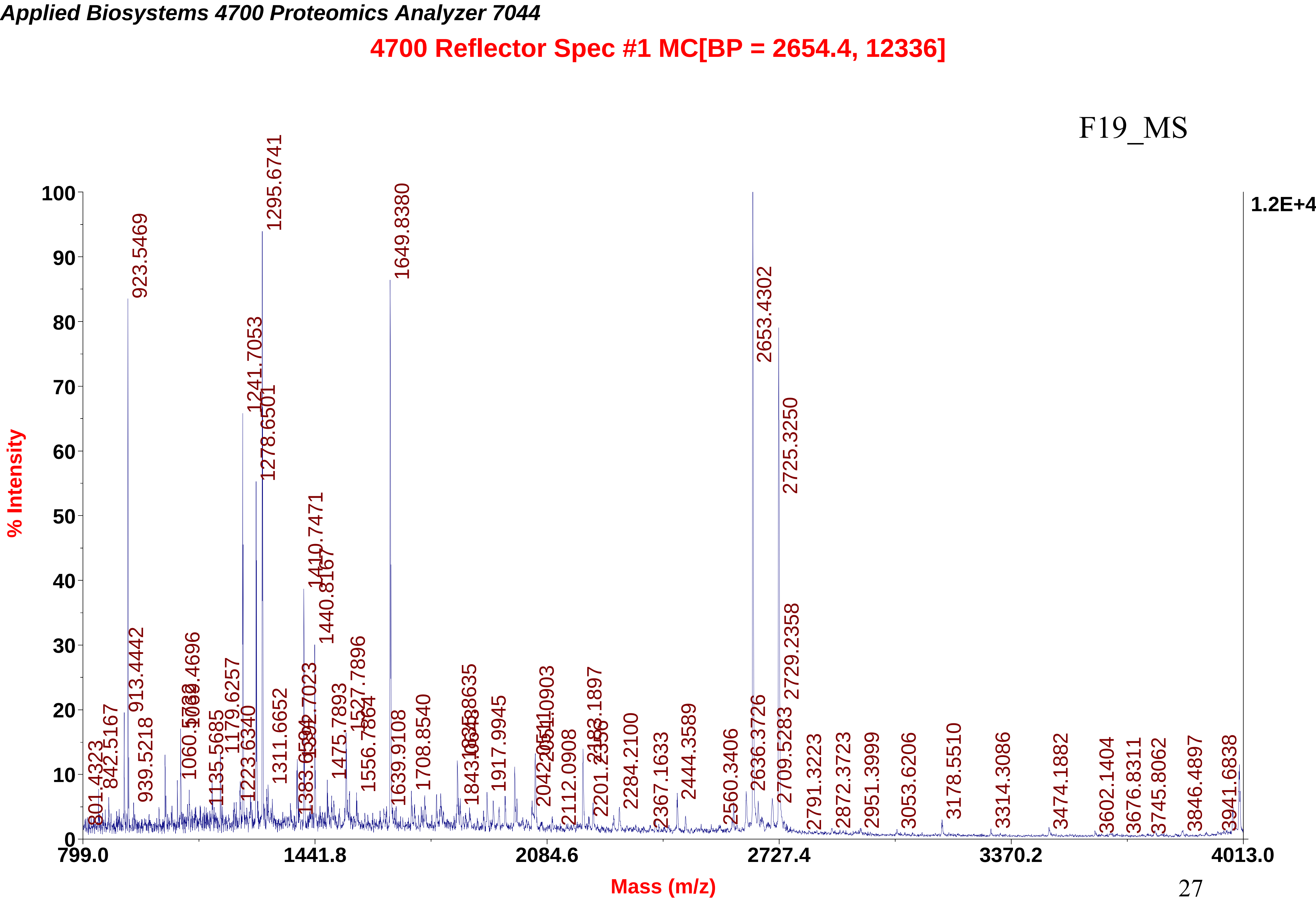
Applied Biosystems 4700 Proteomics Analyzer 7044
4700 Reflector Spec #1 MC[BP = 2654.4, 12336]
% Intensity
Mass (m/z)
1.2E+4
0
10
20
30
40
50
60
70
80
90
100
799.0
1441.8
2084.6
2727.4
3370.2
4013.0
801.4323
842.5167
913.4442
923.5469
939.5218
1135.5685
1179.6257
1223.6340
1241.7053
1278.6501
1295.6741
1311.6652
1410.7471
1440.8167
1475.7893
1527.7896
1556.7864
1639.9108
1649.8380
1708.8540
1917.9945
2112.0908
2183.1897
2284.2100
2367.1633
2444.3589
2560.3406
2636.3726
2653.4302
2709.5283
2725.3250
2729.2358
2791.3223
2872.3723
2951.3999
3053.6206
3178.5510
3314.3086
3474.1882
3602.1404
3676.8311
3745.8062
3846.4897
3941.6838

F19_MS

*Applied Biosystems 4700 Proteomics Analyzer 7044*

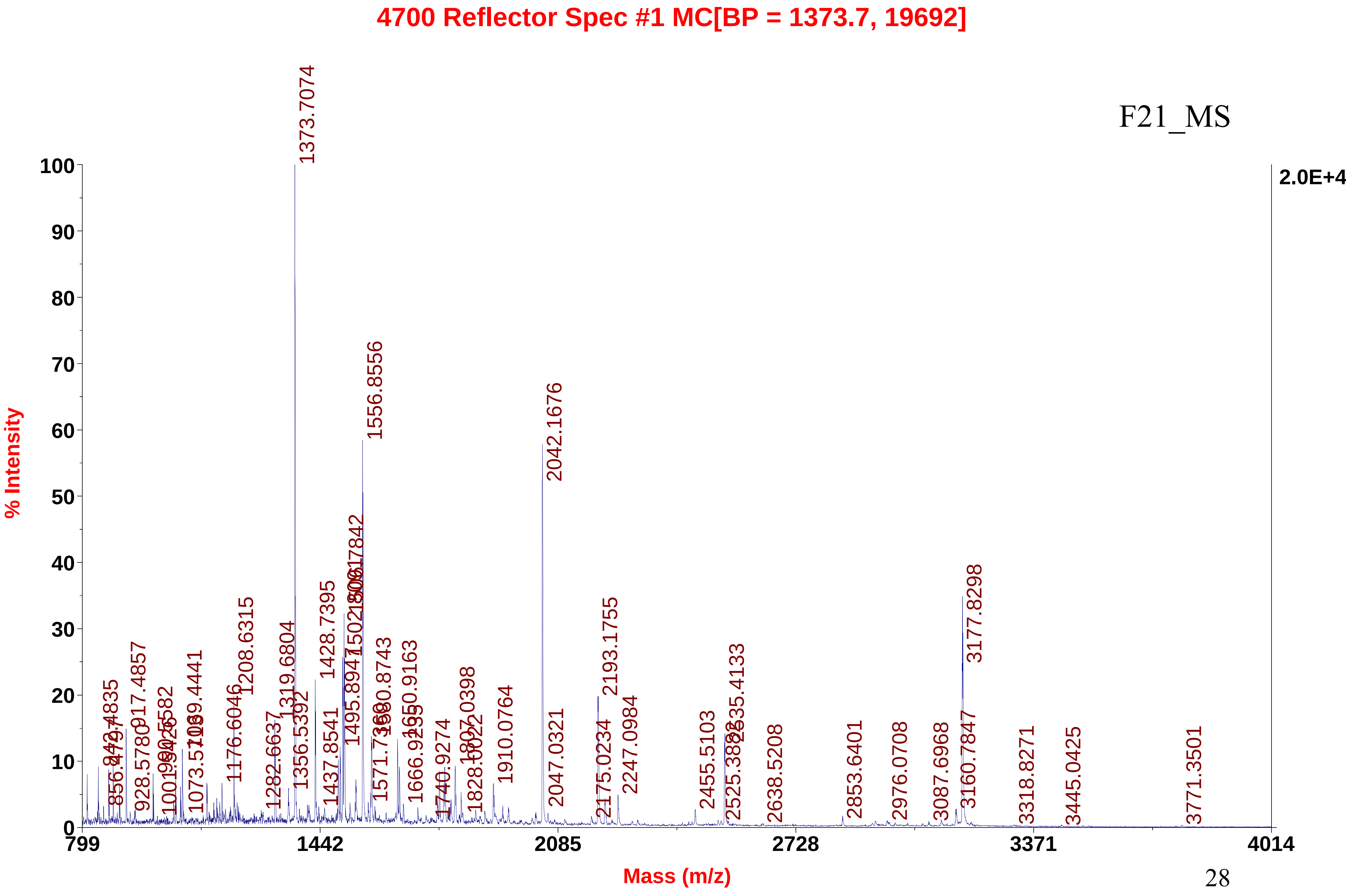

*Applied Biosystems 4700 Proteomics Analyzer 7044*

**4700 Reflector Spec #1 MC[BP = 2043.0, 43645]**

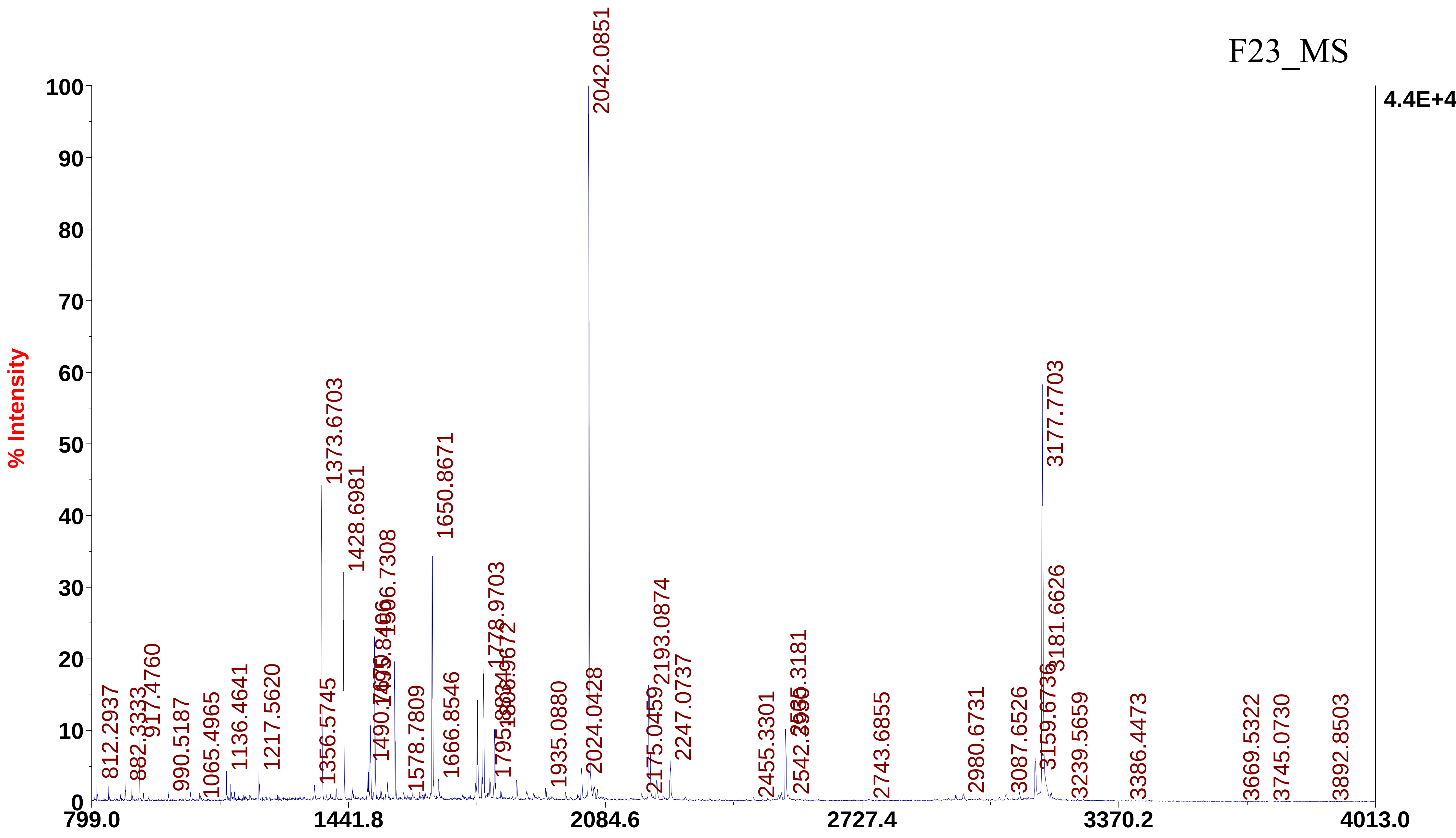

*Applied Biosystems 4700 Proteomics Analyzer 7044*

**4700 Reflector Spec #1 MC[BP = 1420.8, 10267]**

G10_MS

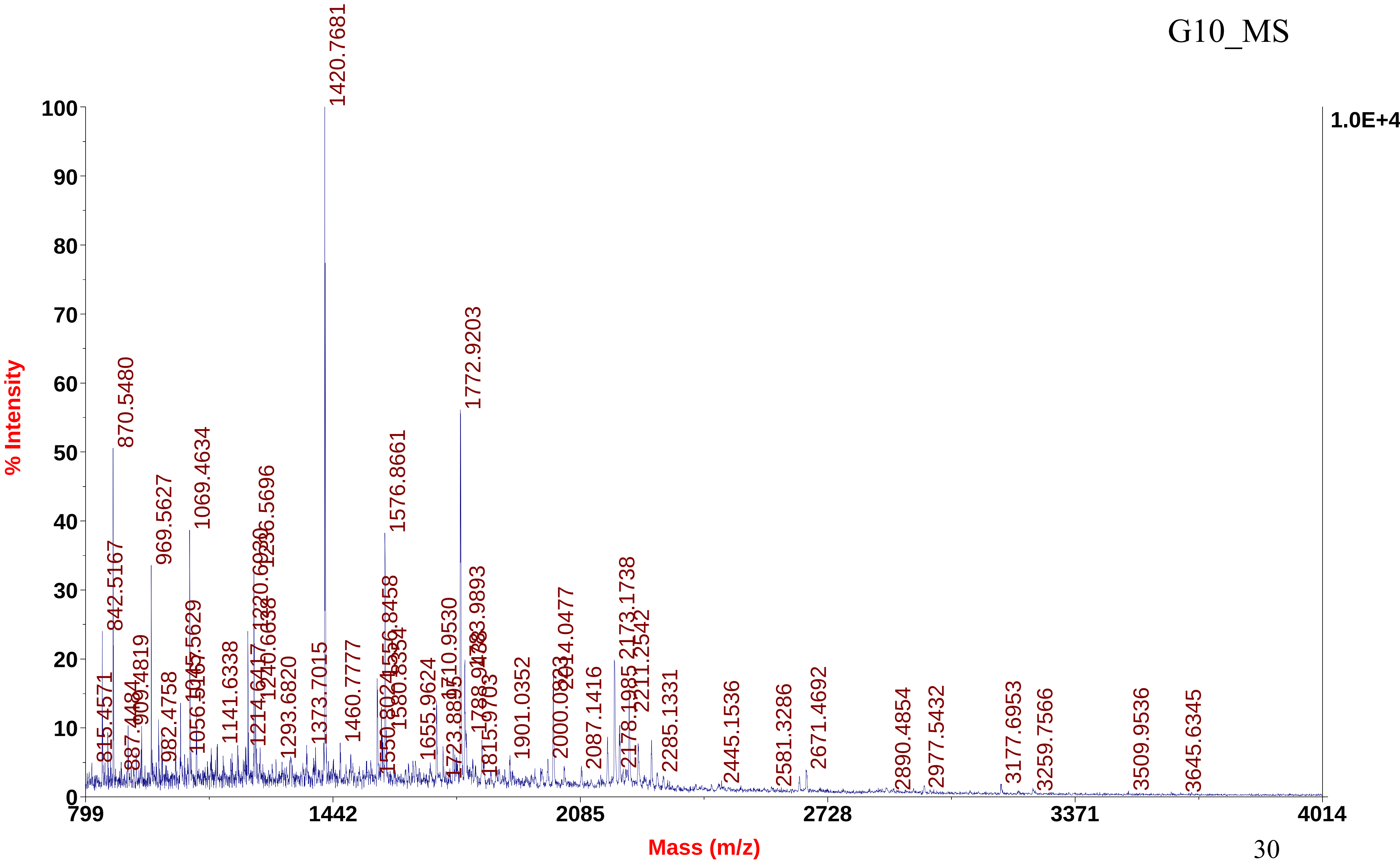

4700 MS/MS Precursor 1201.65 Spec #1 MC[BP = 1184.5, 12670]

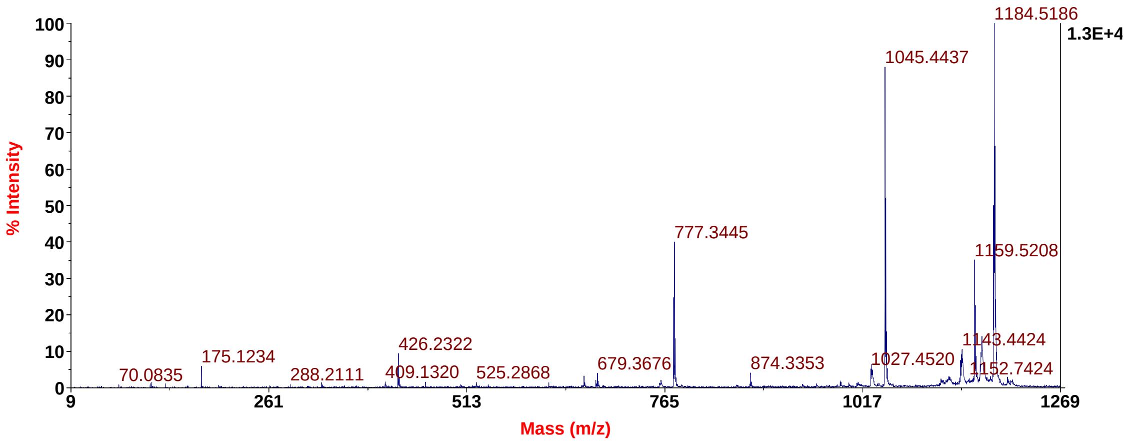


G:\...\C6_MSMS_1201.6477_22.t2d
Acquired:

4700 MS/MS Precursor 1122.62 Spec #1 MC[BP = 175.1, 17361]

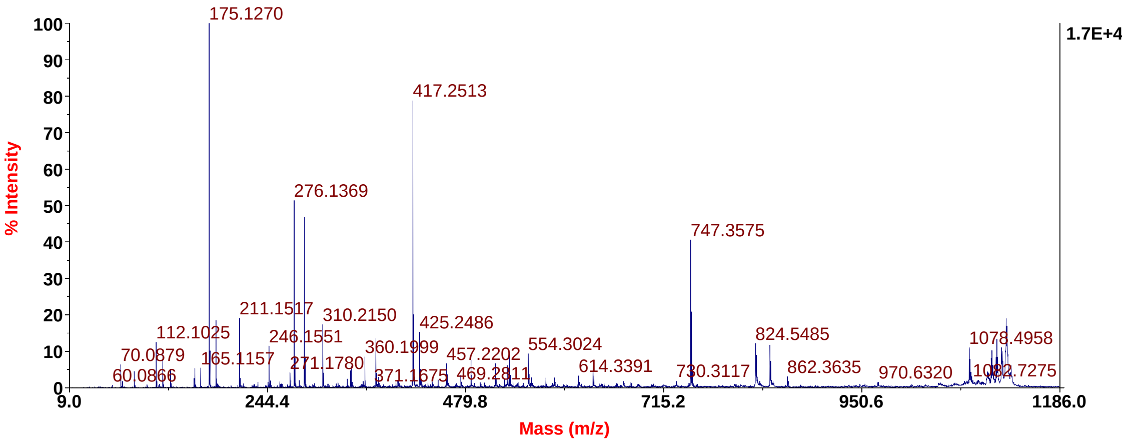


G:\...\C6_MSMS_1122.6207_18.t2d
Acquired:

4700 MS/MS Precursor 1116.66 Spec #1 MC[BP = 175.1, 14970]

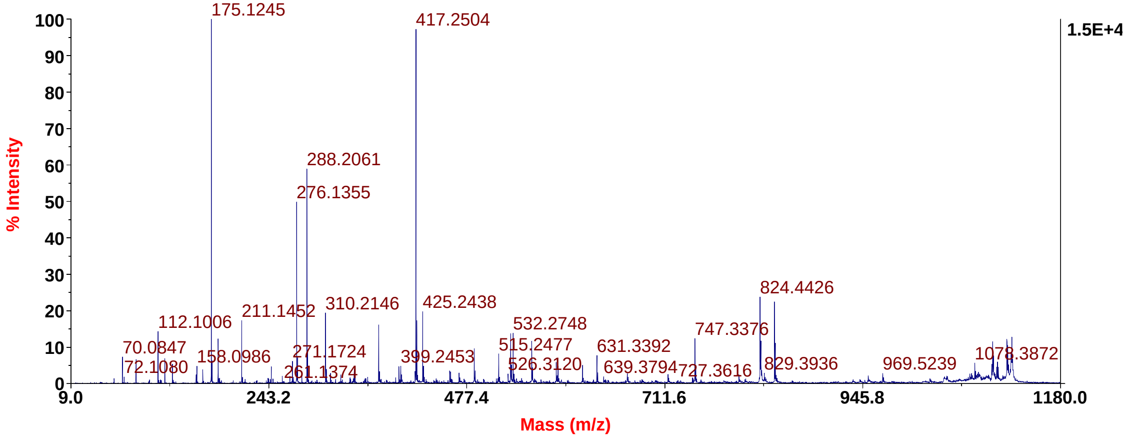


G:\...\C6_MSMS_1116.6627_14.t2d
Acquired:

4700 MS/MS Precursor 1041.67 Spec #1 MC[BP = 360.2, 15787]

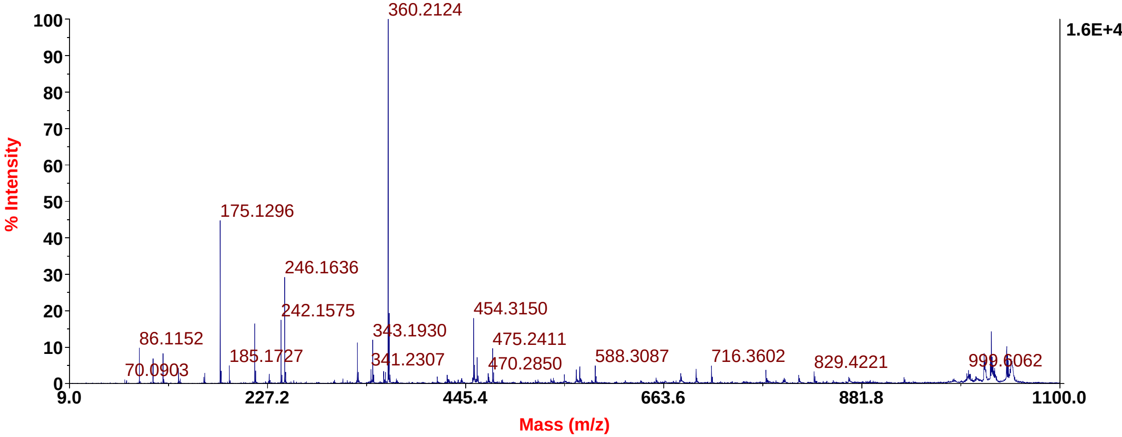


G:\...\C6_MSMS_1041.6664_13.t2d
Acquired:

4700 MS/MS Precursor 1016.61 Spec #1 MC[BP = 999.5, 12440]

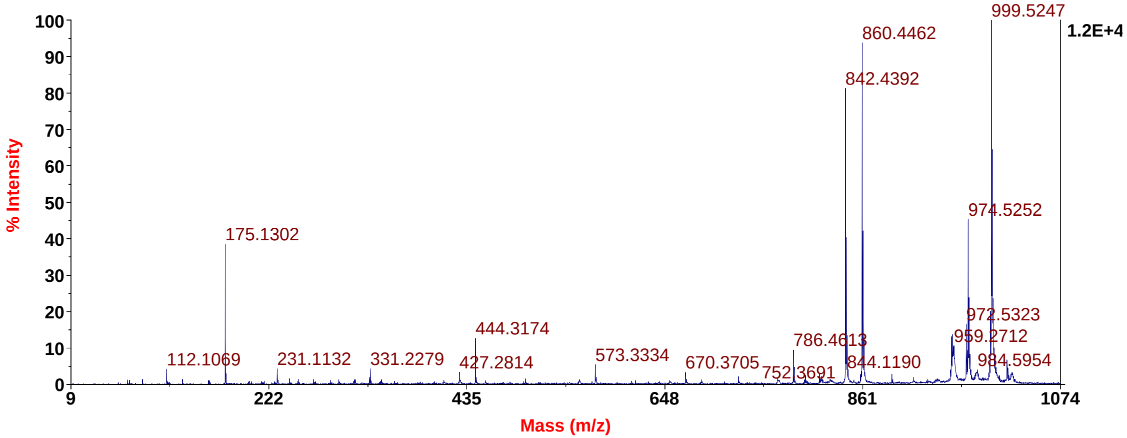


G:\...\C6_MSMS_1016.6128_20.t2d
Acquired:

C6_MSMS_1

4700 MS/MS Precursor 1586.9 Spec #1 MC[BP = 175.1, 6639]

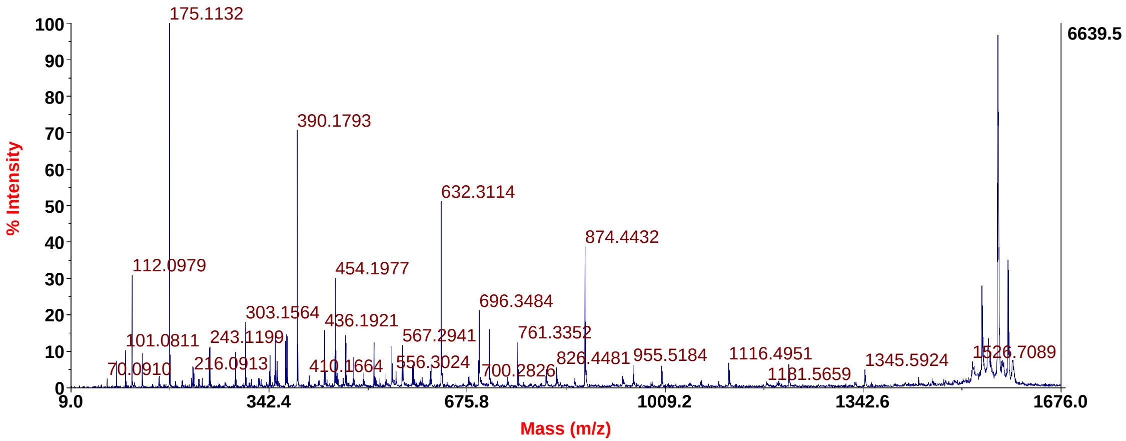


G:\...\C6_MSMS_1586.8973_15.t2d
Acquired:

4700 MS/MS Precursor 1265.69 Spec #1 MC[BP = 175.1, 8812]

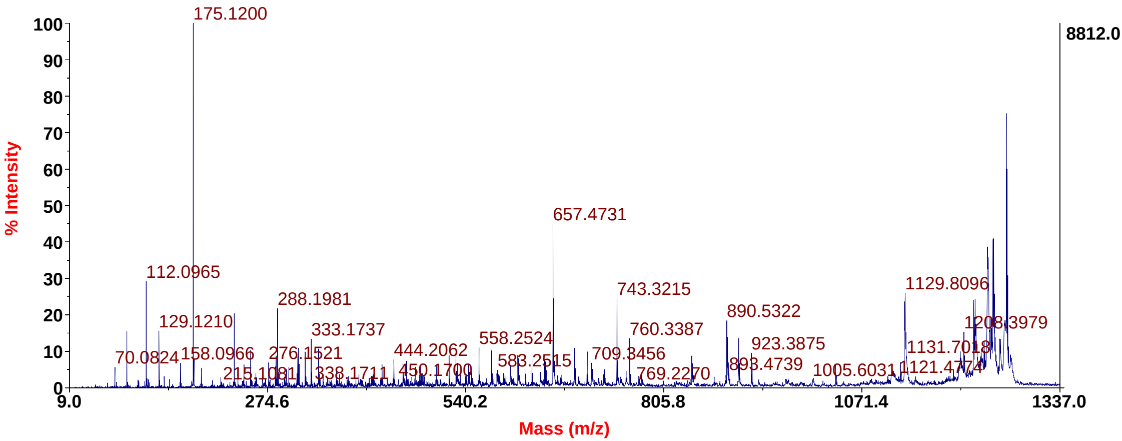


G:\...\C6_MSMS_1265.6862_16.t2d
Acquired:

4700 MS/MS Precursor 1256.71 Spec #1 MC[BP = 1239.6, 5801]

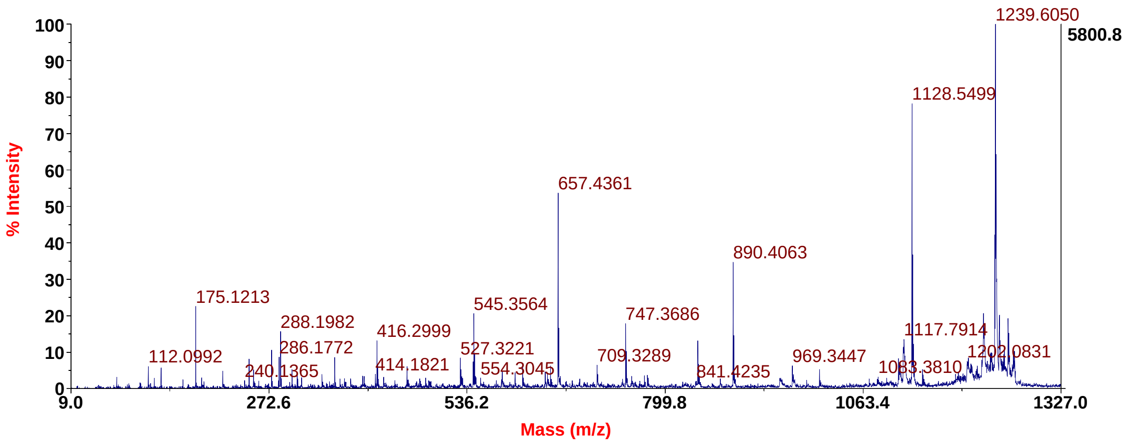


G:\...\C6_MSMS_1256.7070_17.t2d
Acquired:

4700 MS/MS Precursor 1244.77 Spec #1 MC[BP = 1116.6, 5692]

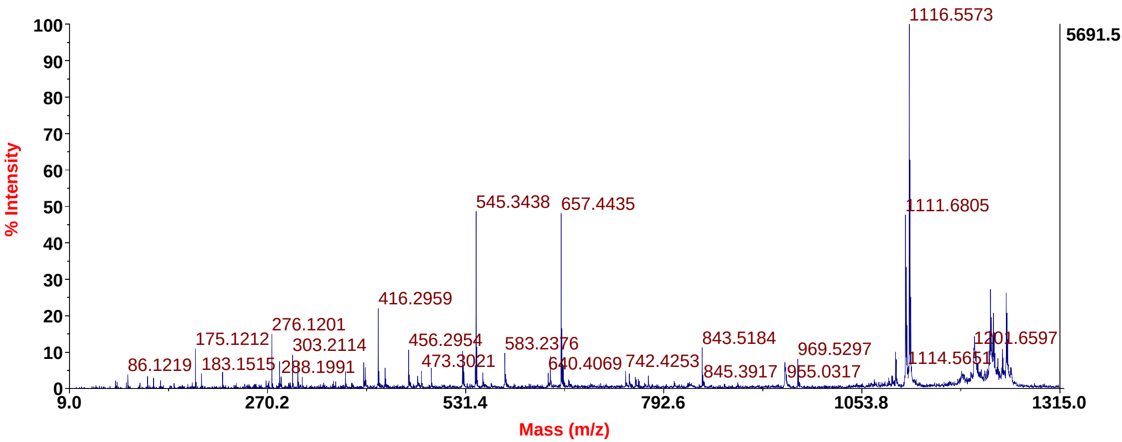


G:\...\C6_MSMS_1244.7698_21.t2d
Acquired:

4700 MS/MS Precursor 1223.73 Spec #1 MC[BP = 175.1, 5165]

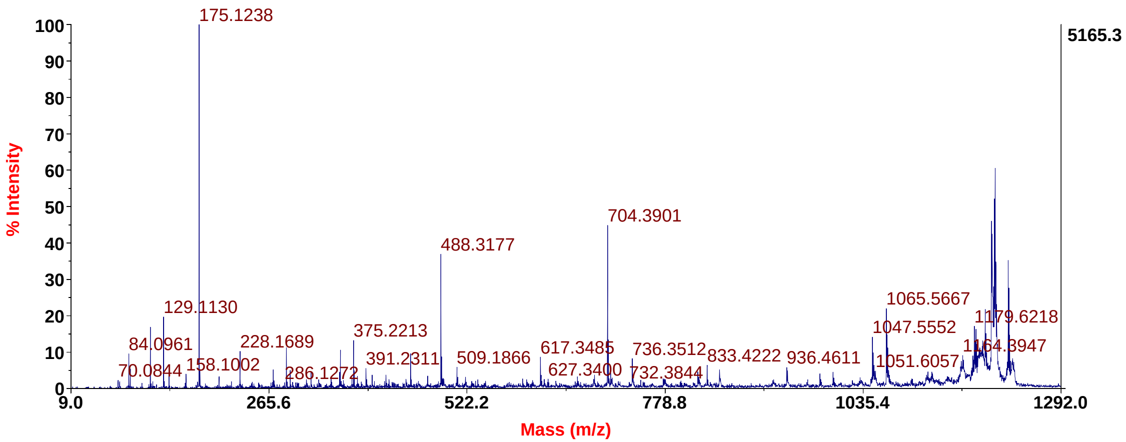


G:\...\C6_MSMS_1223.7274_19.t2d
Acquired:

C6_MSMS_2

**4700 MS/MS Precursor 1128.61 Spec #1 MC[BP = 1111.5, 1384]**

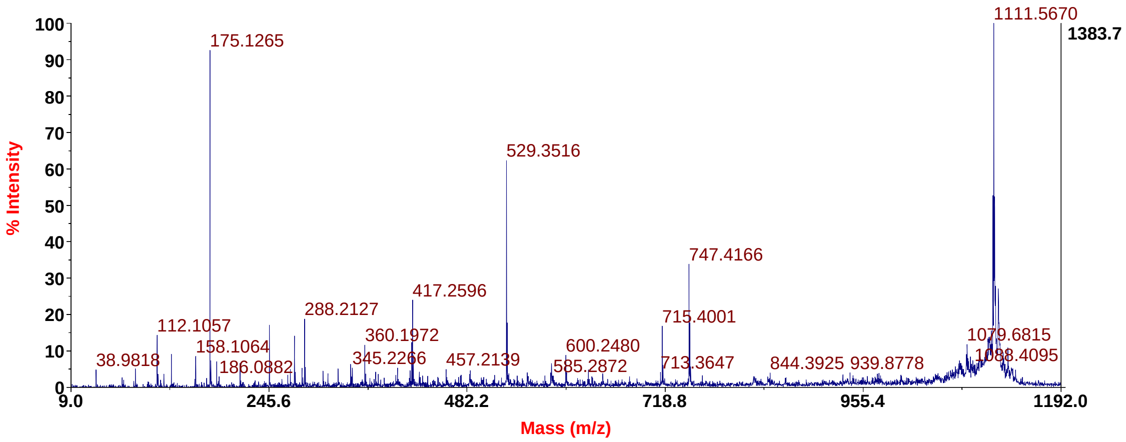


G:\...\C7_MSMS_1128.6085_19.t2d
Acquired:

**4700 MS/MS Precursor 1116.61 Spec #1 MC[BP = 175.1, 1152]**

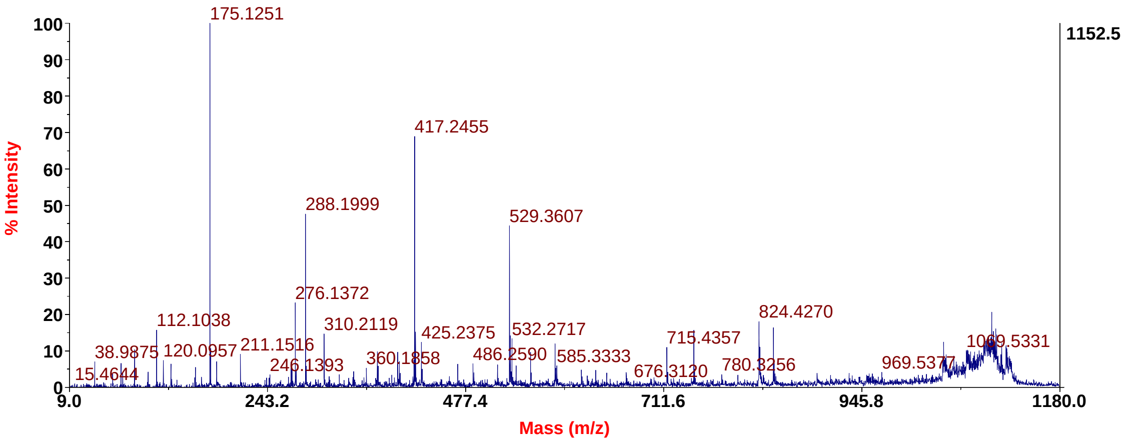


G:\...\C7_MSMS_1116.6068_18.t2d
Acquired:

**4700 MS/MS Precursor 1069.45 Spec #1 MC[BP = 311.2, 437]**

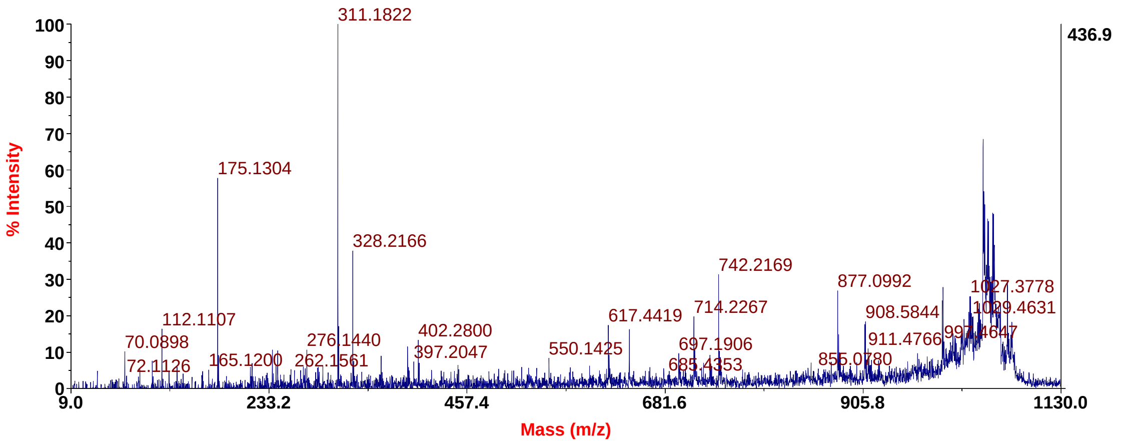


G:\...\C7_MSMS_1069.4493_20.t2d
Acquired:

**4700 MS/MS Precursor 1041.61 Spec #1 MC[BP = 360.2, 2772]**

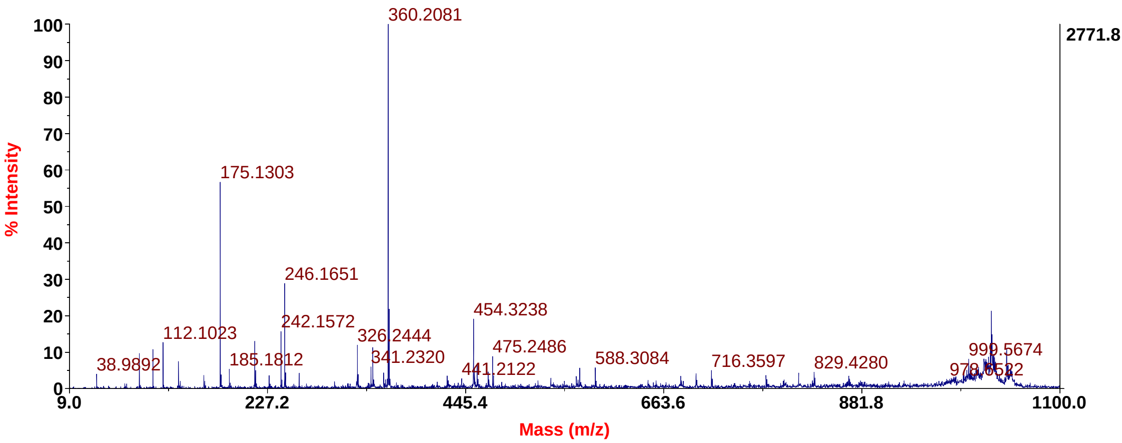


G:\...\C7_MSMS_1041.6071_14.t2d
Acquired:

**4700 MS/MS Precursor 870.538 Spec #1 MC[BP = 100.1, 1698]**

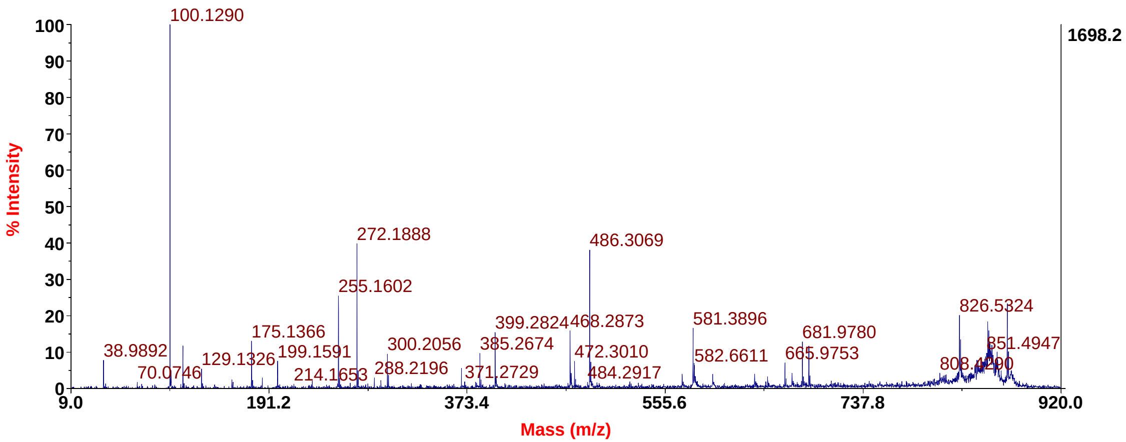


G:\...\C7_MSMS_870.5376_22.t2d
Acquired:

C7_MSMS_1

4700 MS/MS Precursor 1586.86 Spec #1 MC[BP = 175.1, 1752]

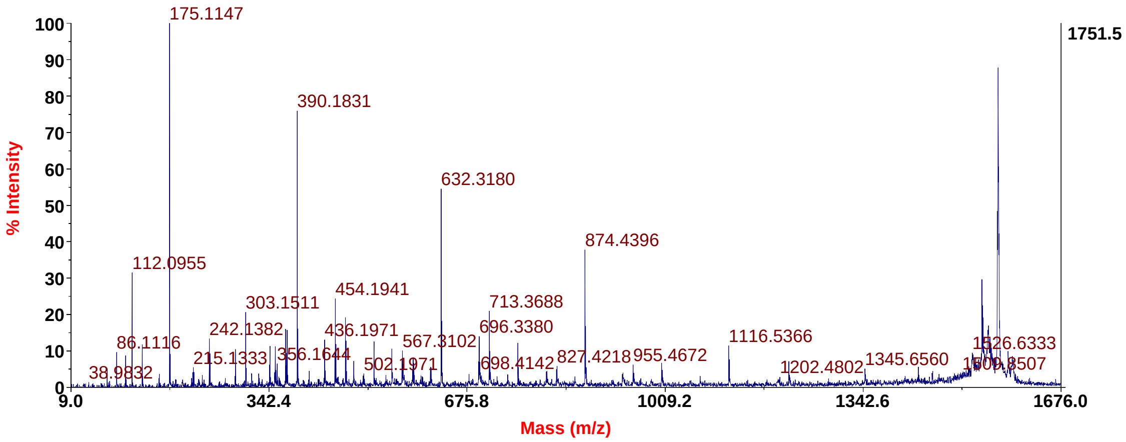


G:\...\C7_MSMS_1586.8617_13.t2d
Acquired:

4700 MS/MS Precursor 1569.84 Spec #1 MC[BP = 175.1, 661]

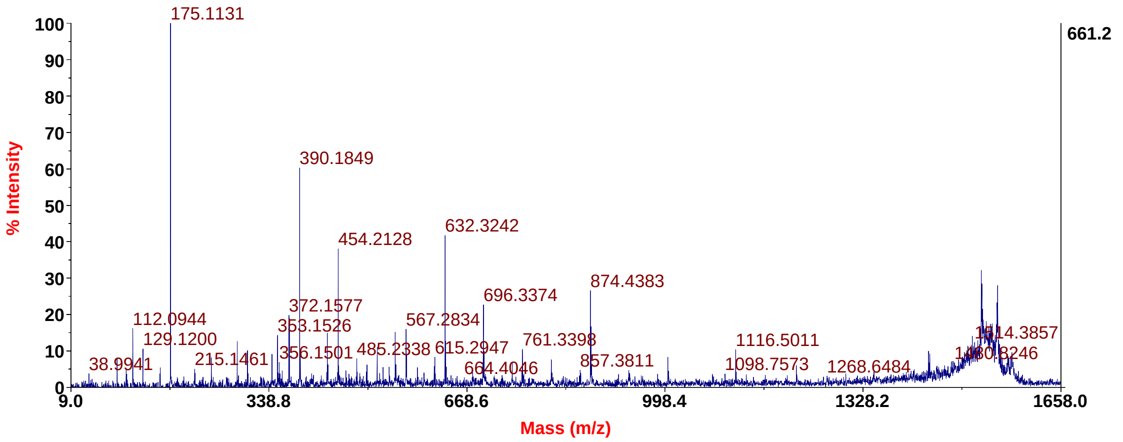


G:\...\C7_MSMS_1569.8358_17.t2d
Acquired:

4700 MS/MS Precursor 1265.64 Spec #1 MC[BP = 175.1, 1140]

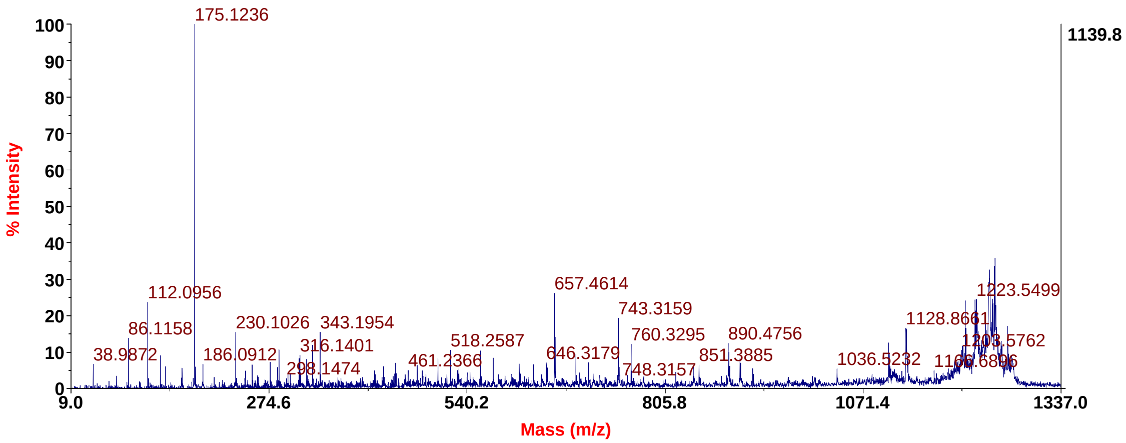


G:\...\C7_MSMS_1265.6366_15.t2d
Acquired:

4700 MS/MS Precursor 1256.65 Spec #1 MC[BP = 1239.6, 720]

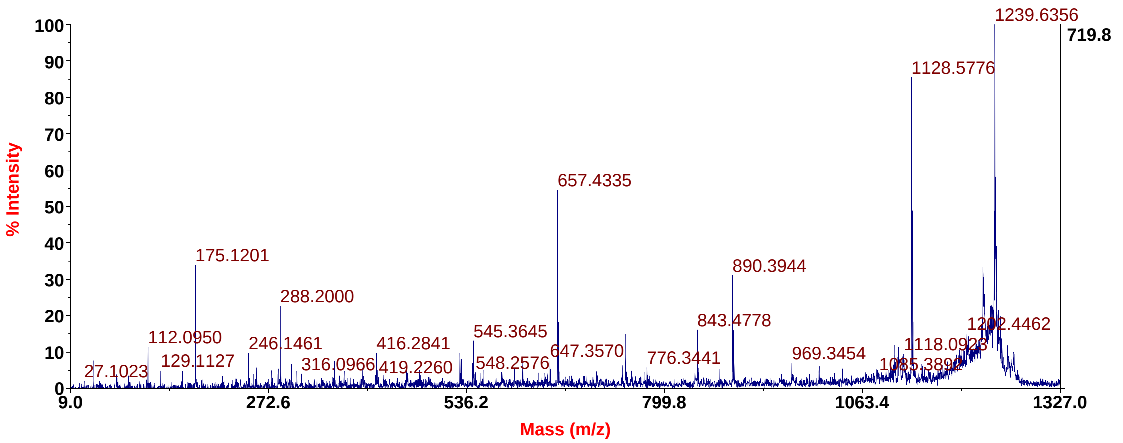


G:\...\C7_MSMS_1256.6512_21.t2d
Acquired:

4700 MS/MS Precursor 1223.67 Spec #1 MC[BP = 175.1, 855]

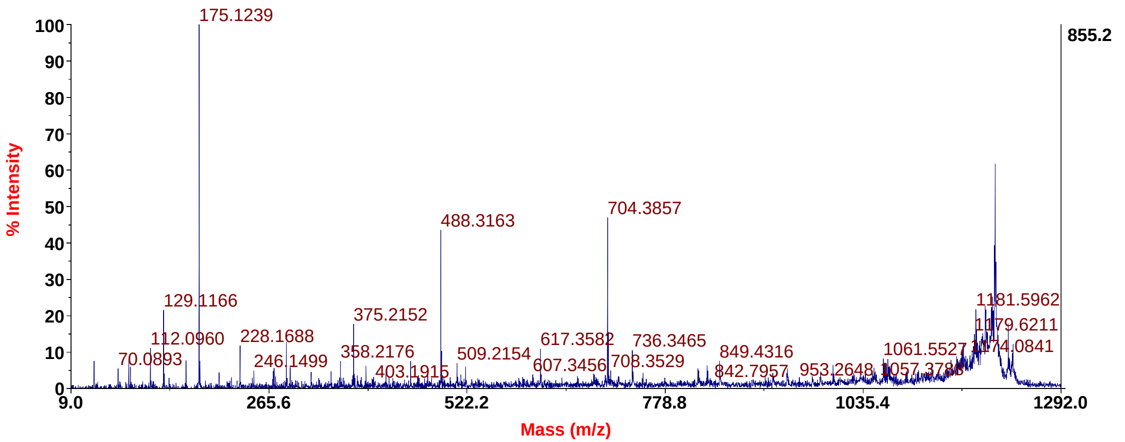


G:\...\C7_MSMS_1223.6694_16.t2d
Acquired:

C7_MSMS_2

4700 MS/MS Precursor 1449.78 Spec #1 MC[BP = 175.1, 395]

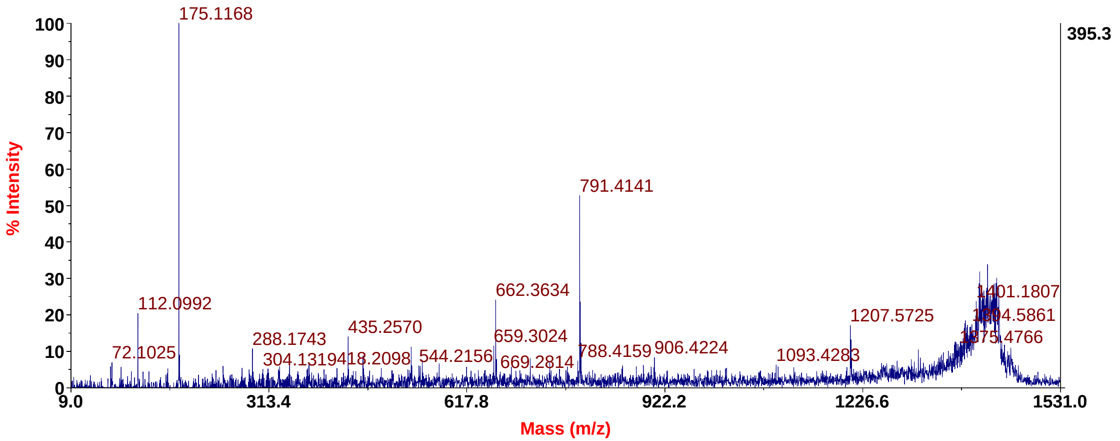


G:\...\C9_MSMS_1449.7772_22.t2d
Acquired:

4700 MS/MS Precursor 1345.7 Spec #1 MC[BP = 757.5, 411]

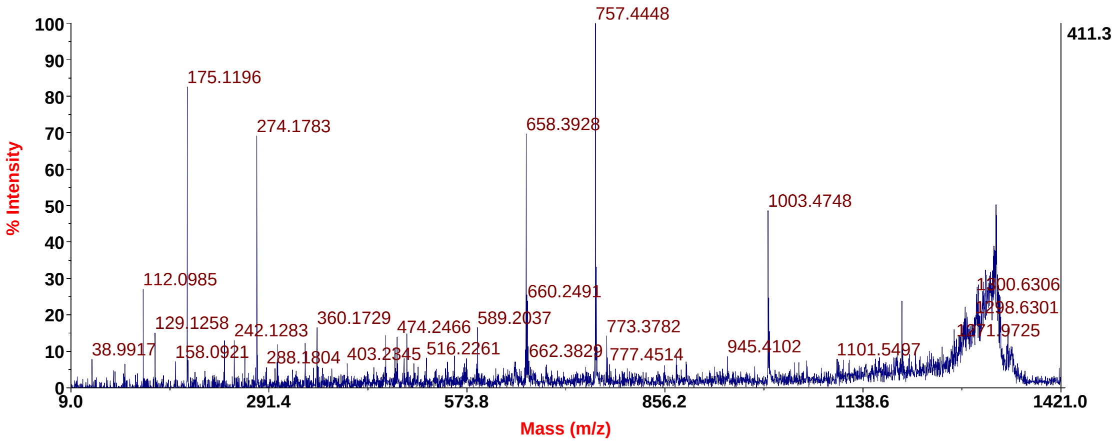


G:\...\C9_MSMS_1345.7010_19.t2d
Acquired:

4700 MS/MS Precursor 1240.59 Spec #1 MC[BP = 175.1, 267]

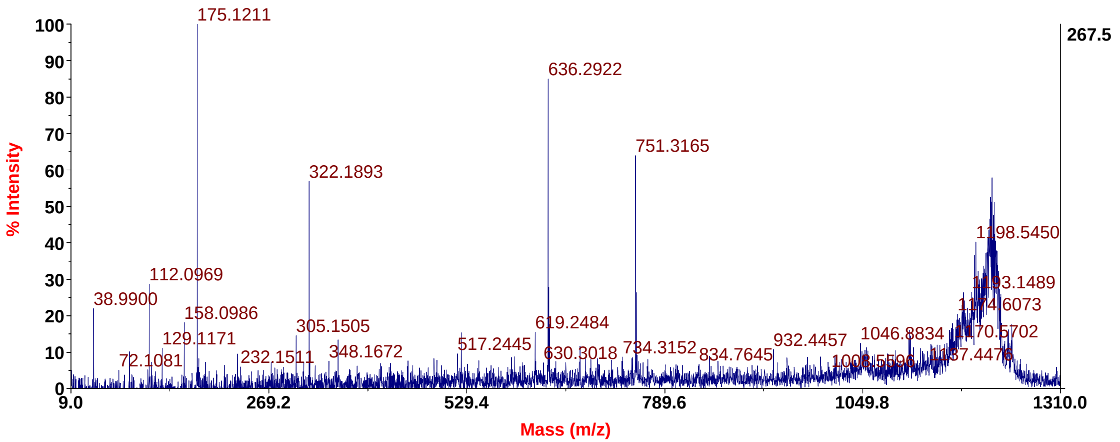


G:\...\C9_MSMS_1240.5851_21.t2d
Acquired:

4700 MS/MS Precursor 1069.45 Spec #1 MC[BP = 311.2, 1012]

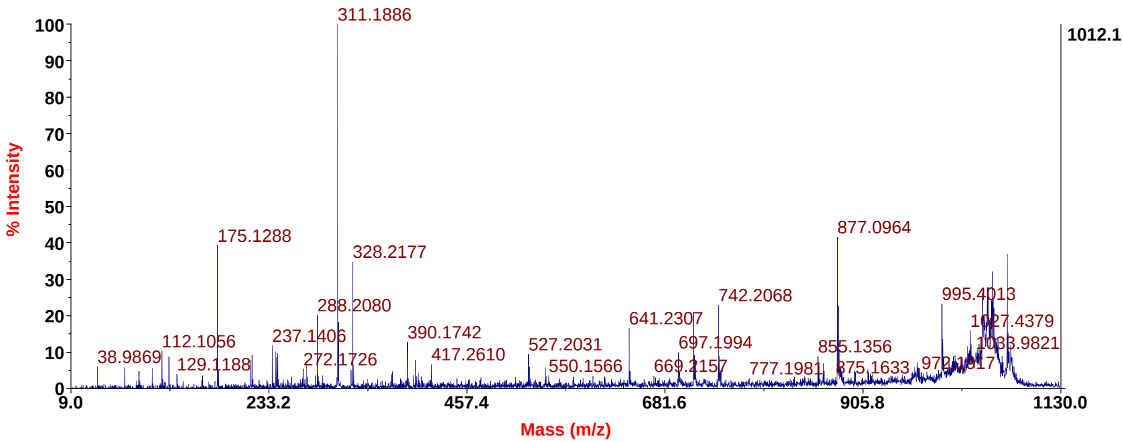


G:\...\C9_MSMS_1069.4508_13.t2d
Acquired:

4700 MS/MS Precursor 870.549 Spec #1 MC[BP = 100.1, 2144]

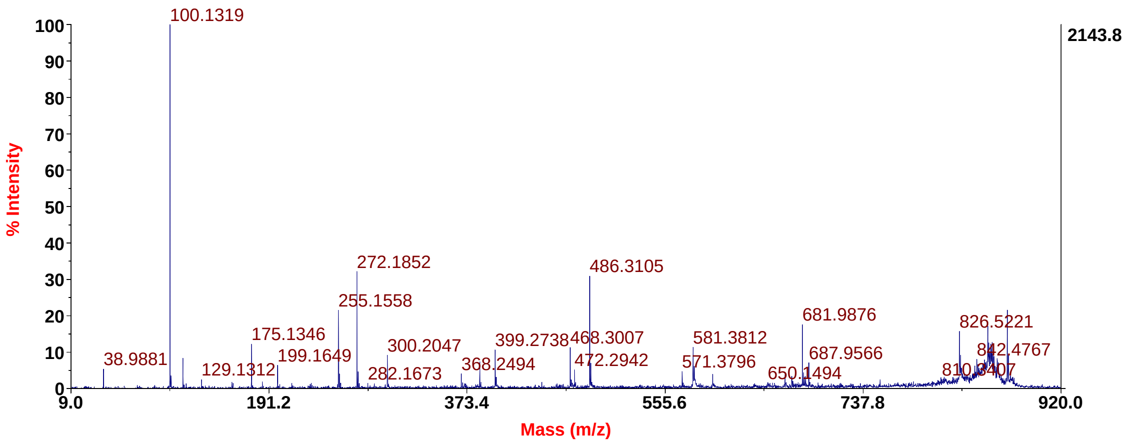


G:\...\C9_MSMS_870.5493_14.t2d
Acquired:

# C9_MSMS_1

**4700 MS/MS Precursor 2112.08 Spec #1 MC[BP = 1955.8, 533]**

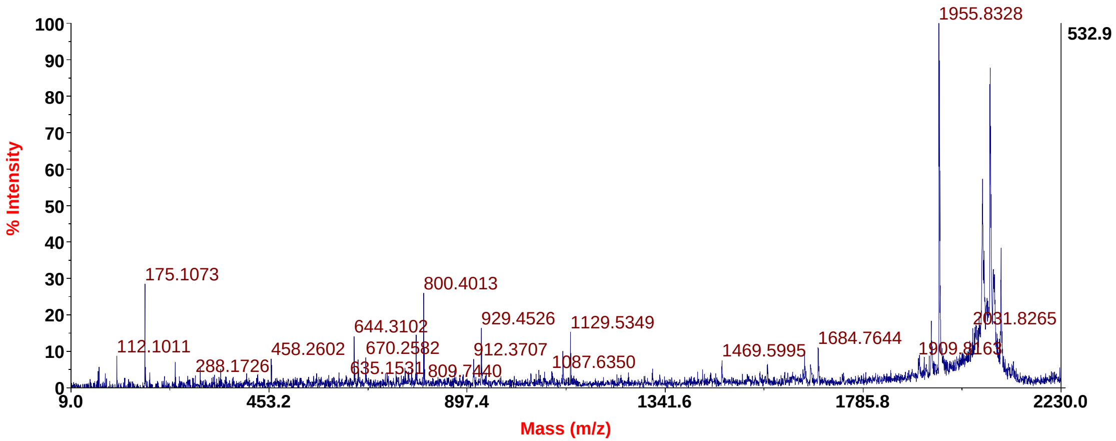


G:\...\C9_MSMS_2112.0830_17.t2d
Acquired:

**4700 MS/MS Precursor 1899.01 Spec #1 MC[BP = 1859.8, 147]**

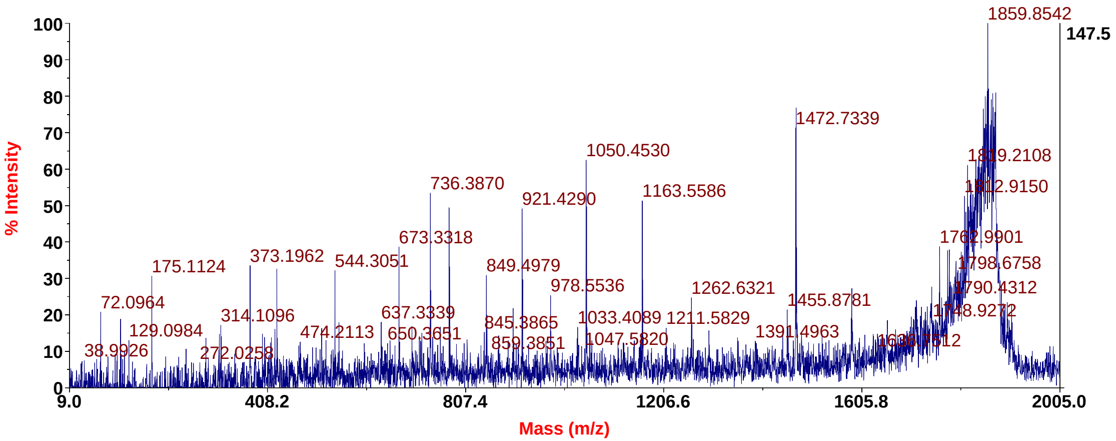


G:\...\C9_MSMS_1899.0127_20.t2d
Acquired:

**4700 MS/MS Precursor 1790.91 Spec #1 MC[BP = 1086.5, 908]**

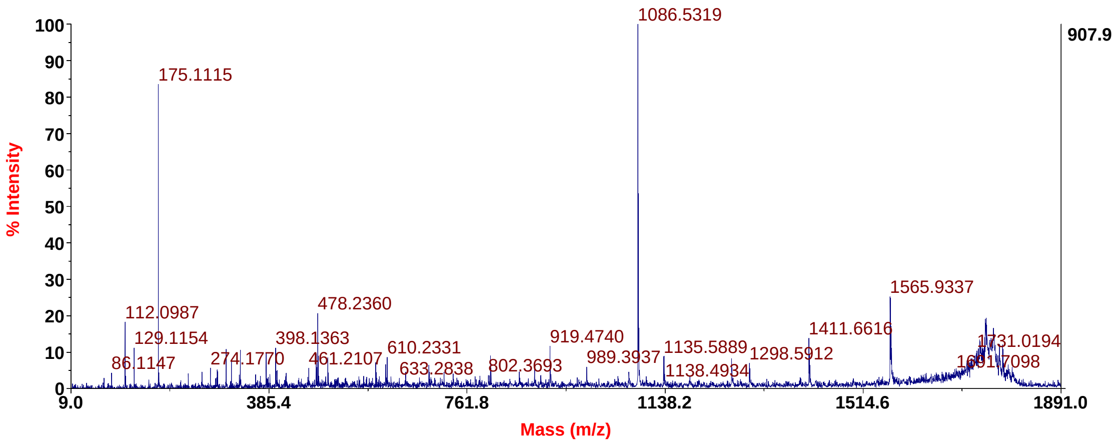


G:\...\C9_MSMS_1790.9106_15.t2d
Acquired:

**4700 MS/MS Precursor 1779.87 Spec #1 MC[BP = 175.1, 417]**

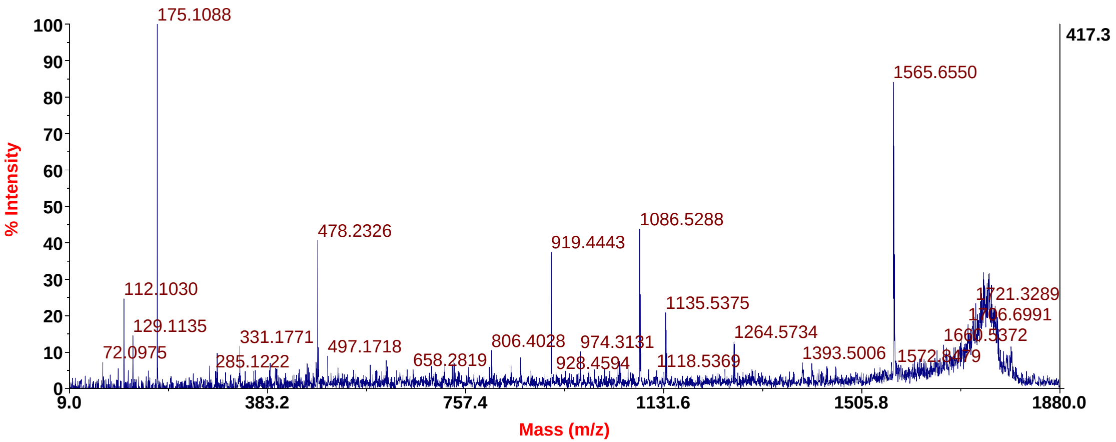


G:\...\C9_MSMS_1779.8698_16.t2d
Acquired:

**4700 MS/MS Precursor 1486.74 Spec #1 MC[BP = 175.1, 309]**

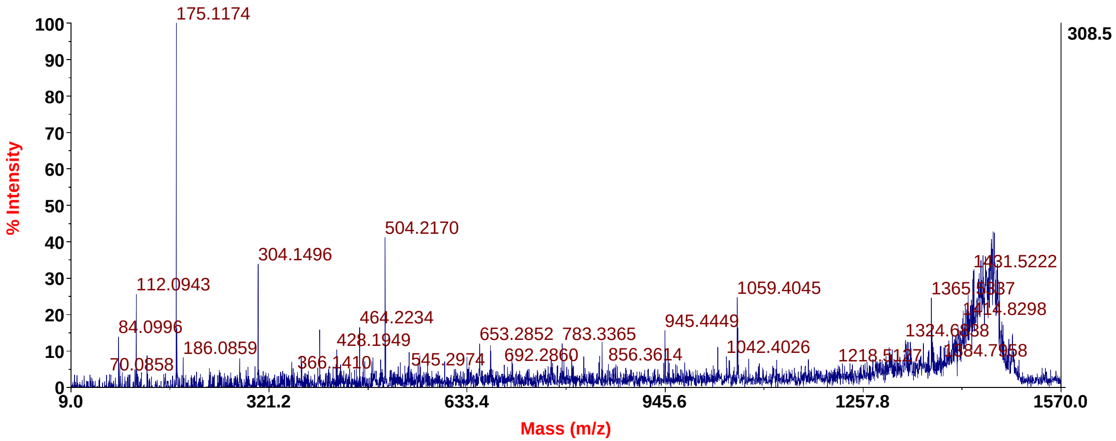


G:\...\C9_MSMS_1486.7386_18.t2d
Acquired:

C9_MSMS_2

**4700 MS/MS Precursor 1208.66 Spec #1 MC[BP = 1190.8, 137]**

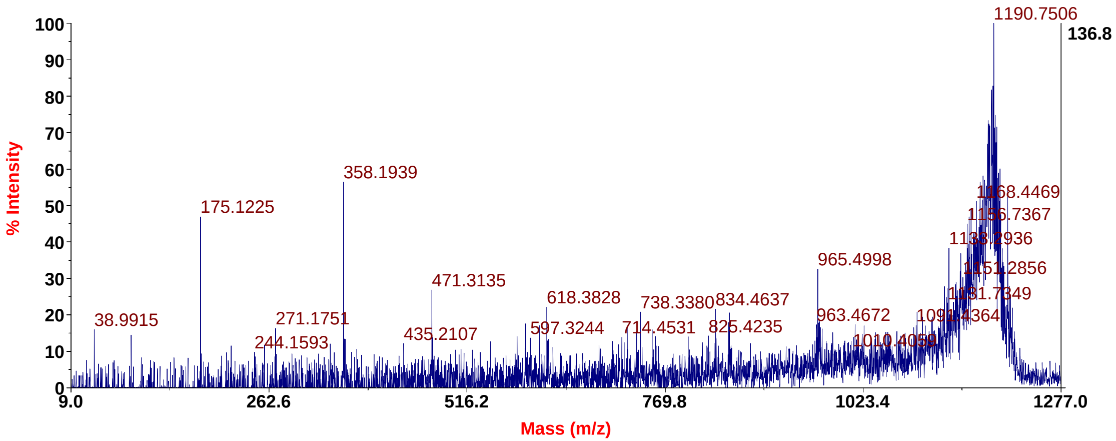


G:\...\C17_MSMS_1208.6561_22.t2d
Acquired:

**4700 MS/MS Precursor 1069.46 Spec #1 MC[BP = 1069.4, 785]**

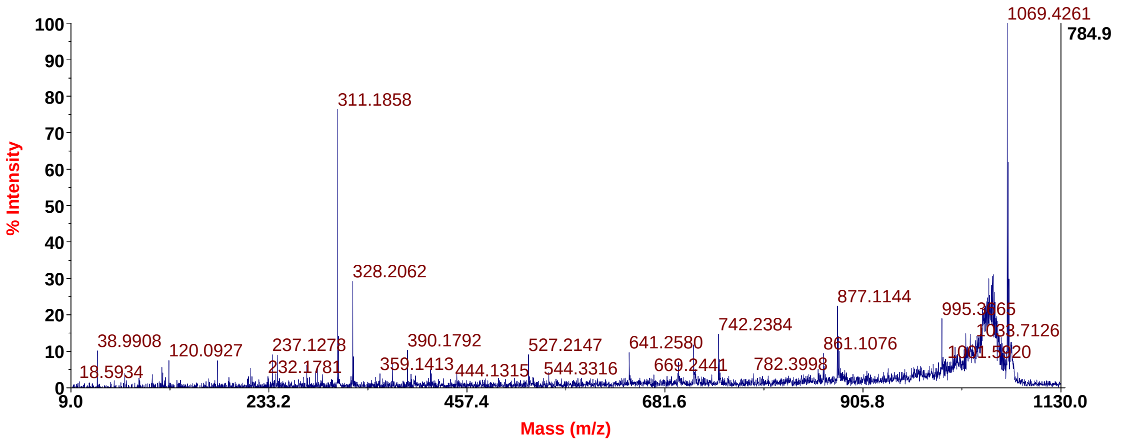


G:\...\C17_MSMS_1069.4579_13.t2d
Acquired:

**4700 MS/MS Precursor 1012.51 Spec #1 MC[BP = 1012.5, 712]**

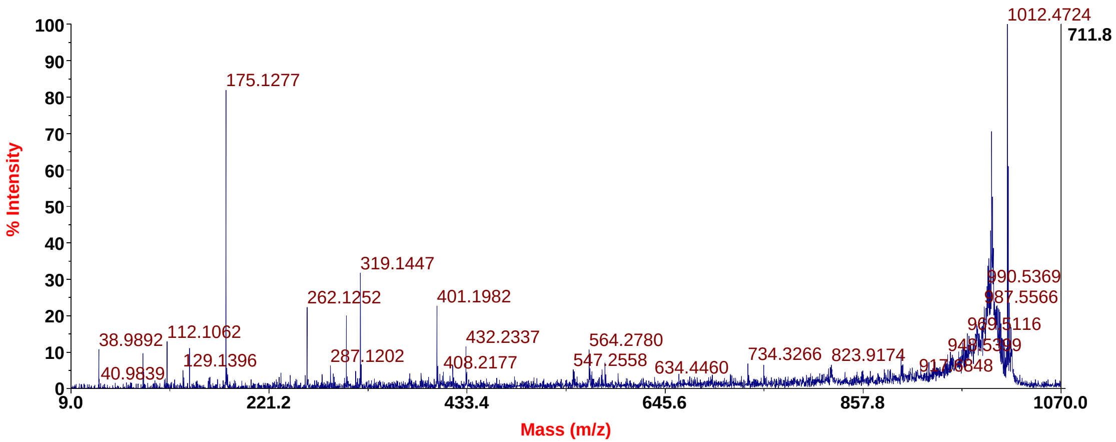


G:\...\C17_MSMS_1012.5068_14.t2d
Acquired:

**4700 MS/MS Precursor 917.327 Spec #1 MC[BP = 899.3, 1476]**

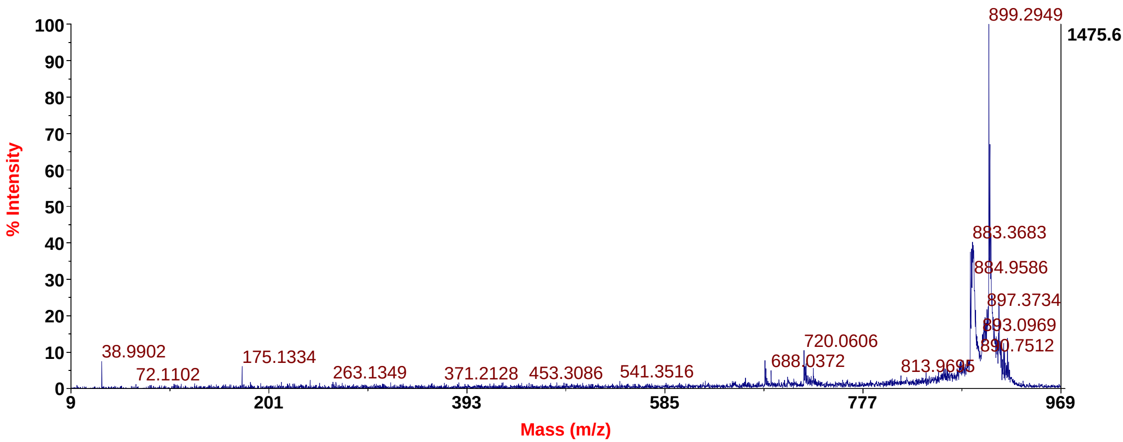


G:\...\C17_MSMS_917.3273_20.t2d
Acquired:

**4700 MS/MS Precursor 870.552 Spec #1 MC[BP = 870.5, 1248]**

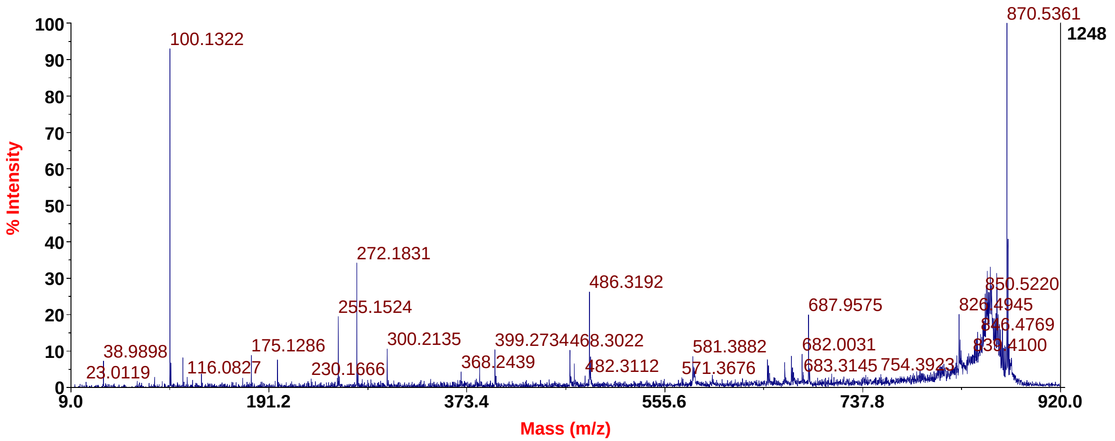


G:\...\C17_MSMS_870.5518_15.t2d
Acquired:

C17_MSMS_1

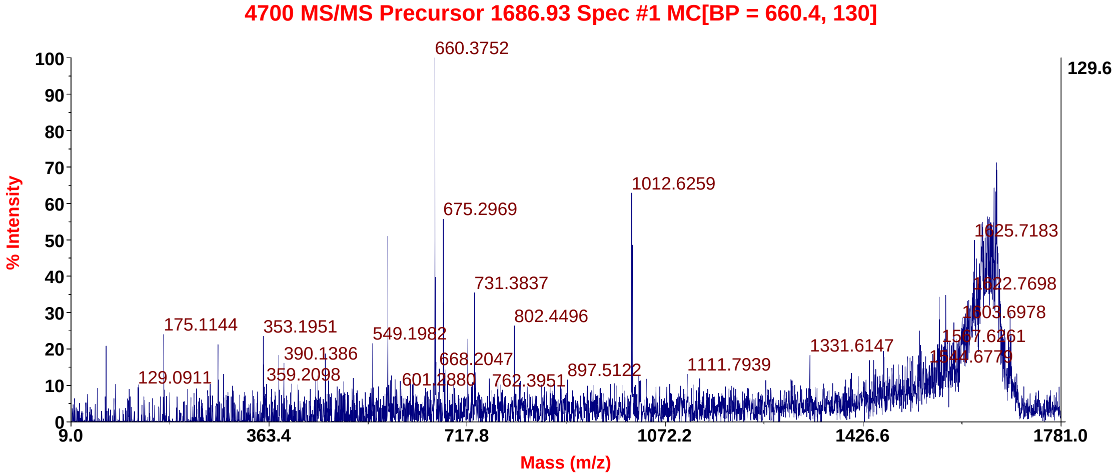


G:\...\C17_MSMS_1686.9269_21.t2d
Acquired:

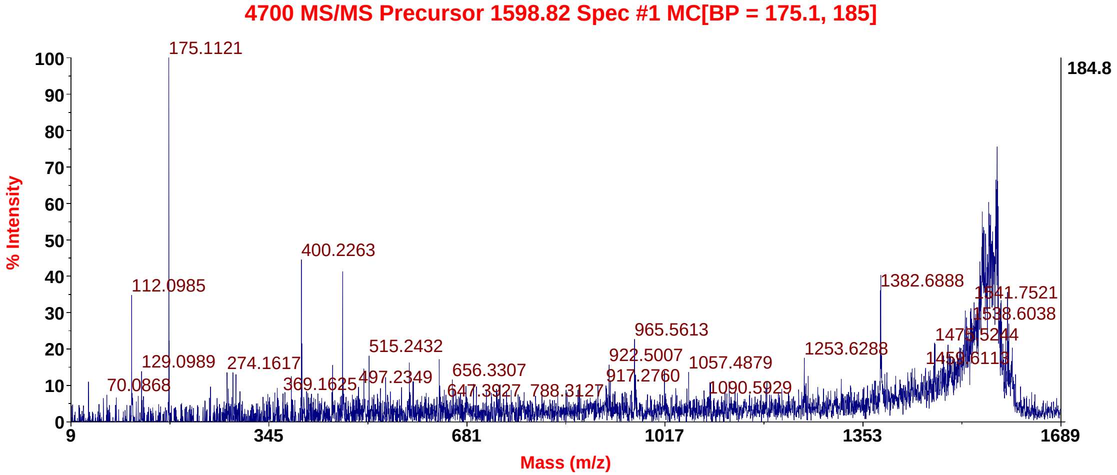


G:\...\C17_MSMS_1598.8199_18.t2d
Acquired:

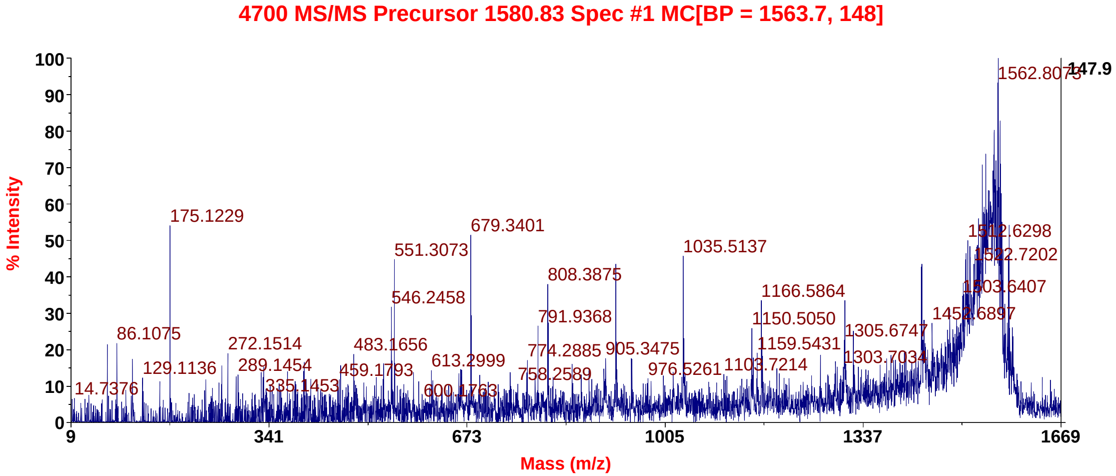


G:\...\C17_MSMS_1580.8340_19.t2d
Acquired:

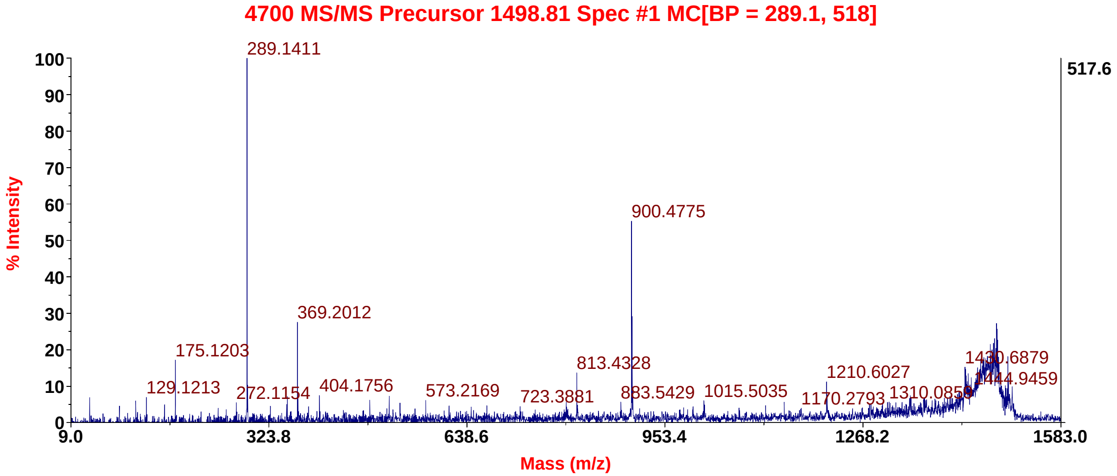


G:\...\C17_MSMS_1498.8085_17.t2d
Acquired:

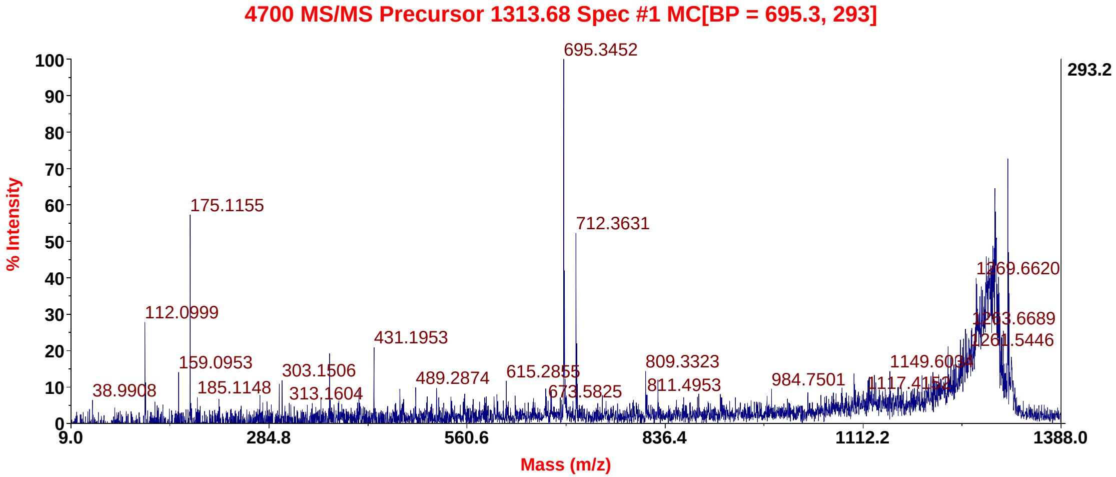


G:\...\C17_MSMS_1313.6760_16.t2d
Acquired:

C17_MSMS_2

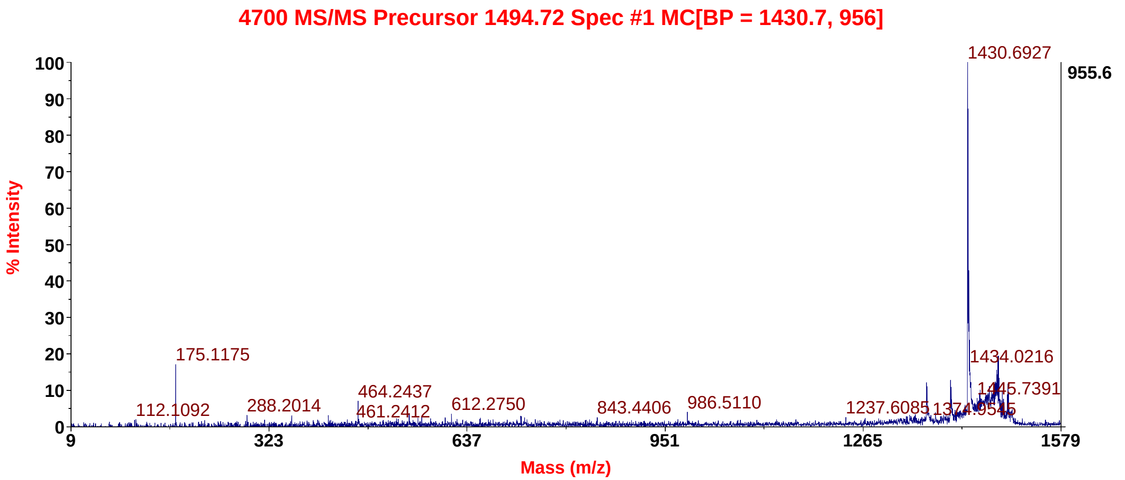


G:\...\C18_MSMS_1494.7206_19.t2d
Acquired:

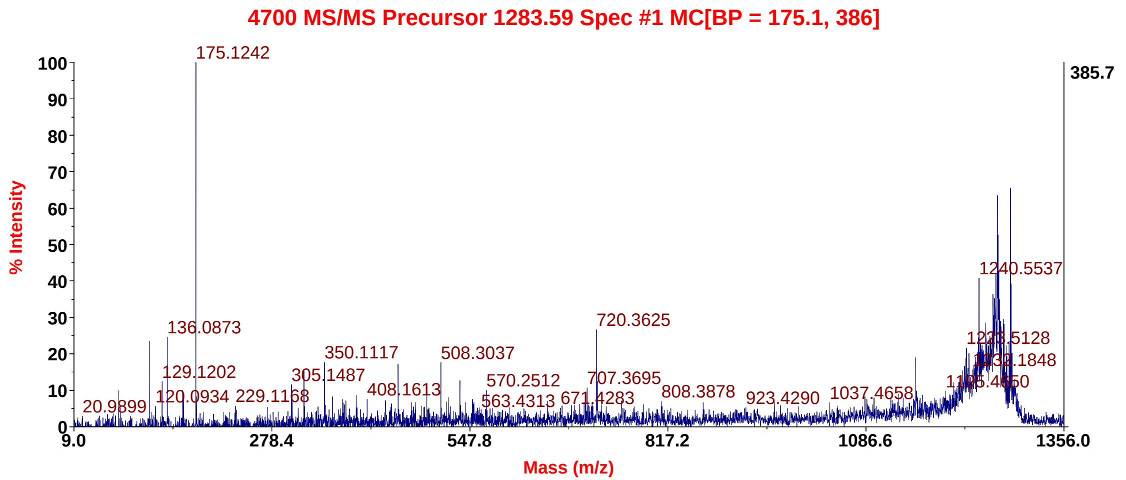


G:\...\C18_MSMS_1283.5887_17.t2d
Acquired:

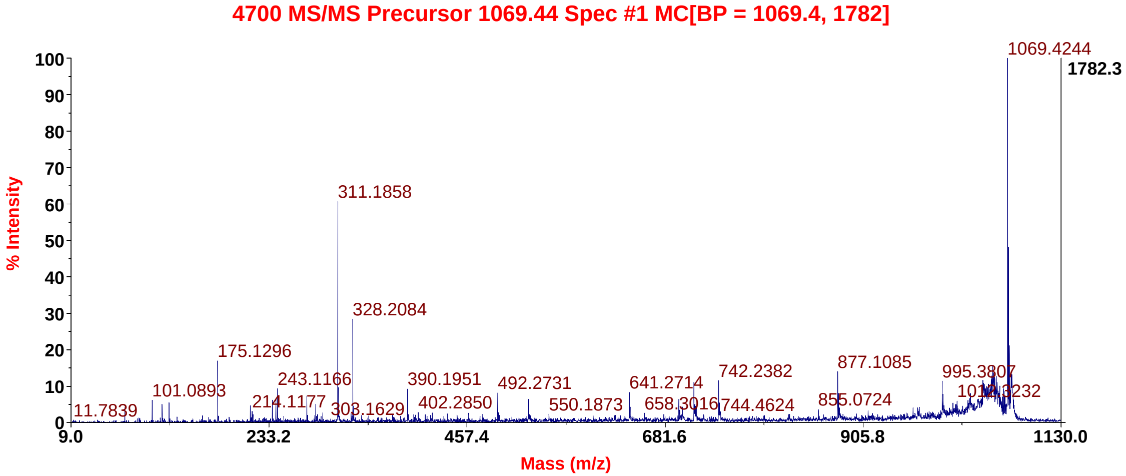


G:\...\C18_MSMS_1069.4391_13.t2d
Acquired:

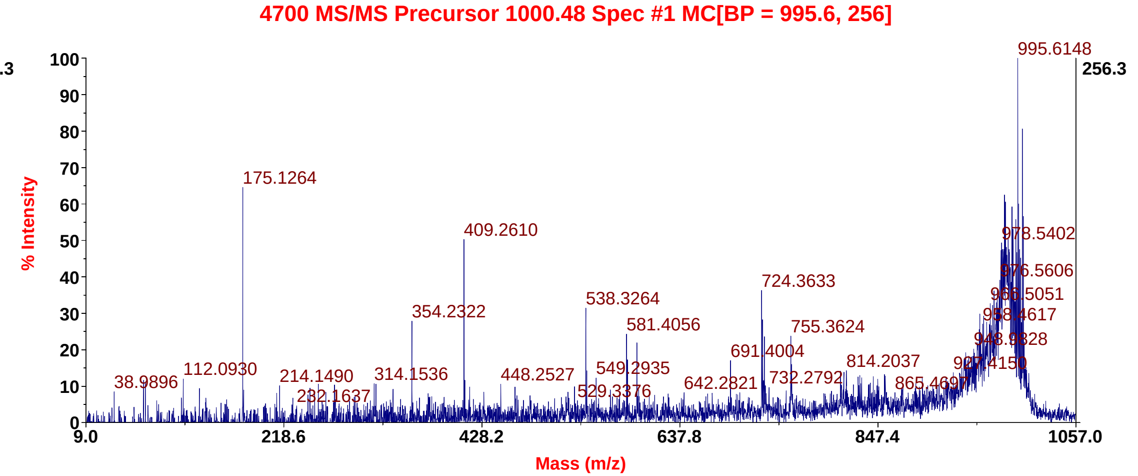


G:\...\C18_MSMS_1000.4761_21.t2d
Acquired:

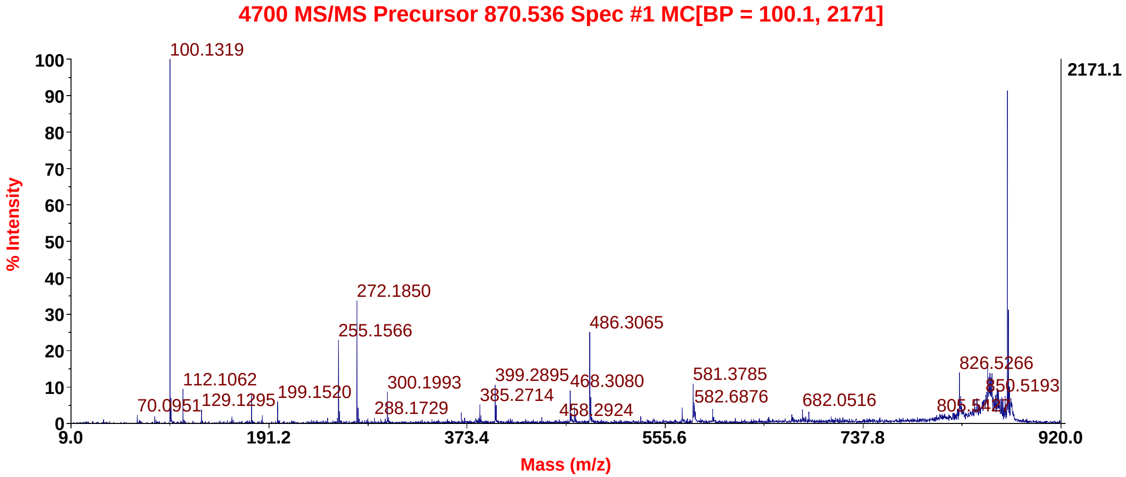


G:\...\C18_MSMS_870.5362_15.t2d
Acquired:

# C18_MSMS_1

**4700 MS/MS Precursor 1832.93 Spec #1 MC[BP = 854.4, 2169]**

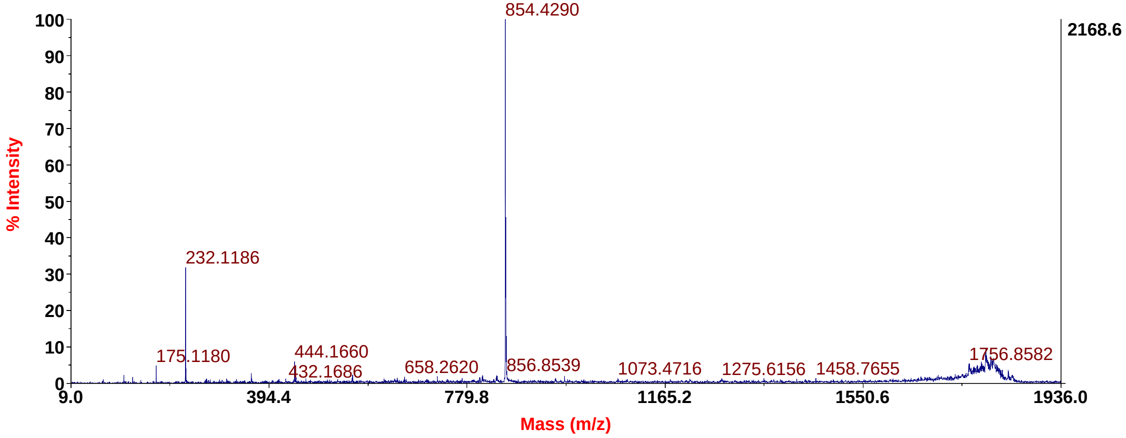


G:\...\C18_MSMS_1832.9258_16.t2d
Acquired:

**4700 MS/MS Precursor 1788.76 Spec #1 MC[BP = 232.1, 453]**

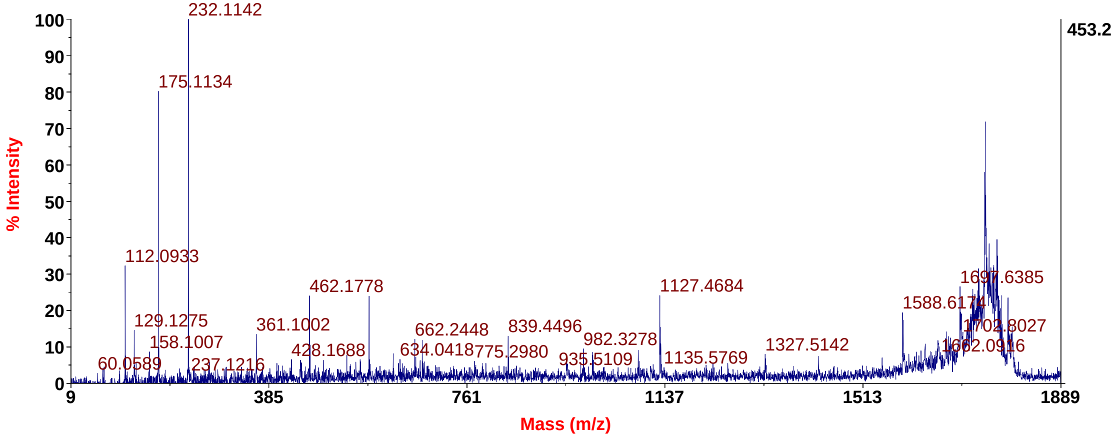


G:\...\C18_MSMS_1788.7620_14.t2d
Acquired:

**4700 MS/MS Precursor 1565.8 Spec #1 MC[BP = 1437.6, 203]**

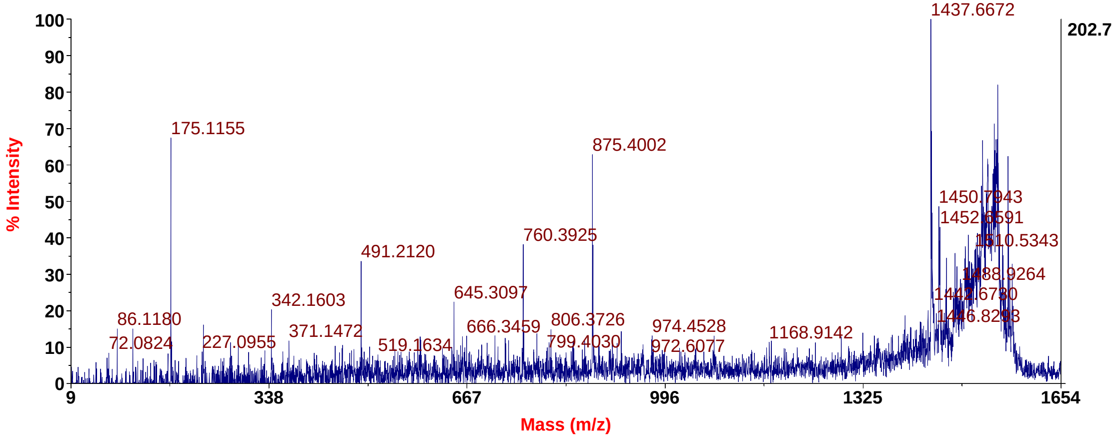


G:\...\C18_MSMS_1565.8037_20.t2d
Acquired:

**4700 MS/MS Precursor 1531.68 Spec #1 MC[BP = 175.1, 377]**

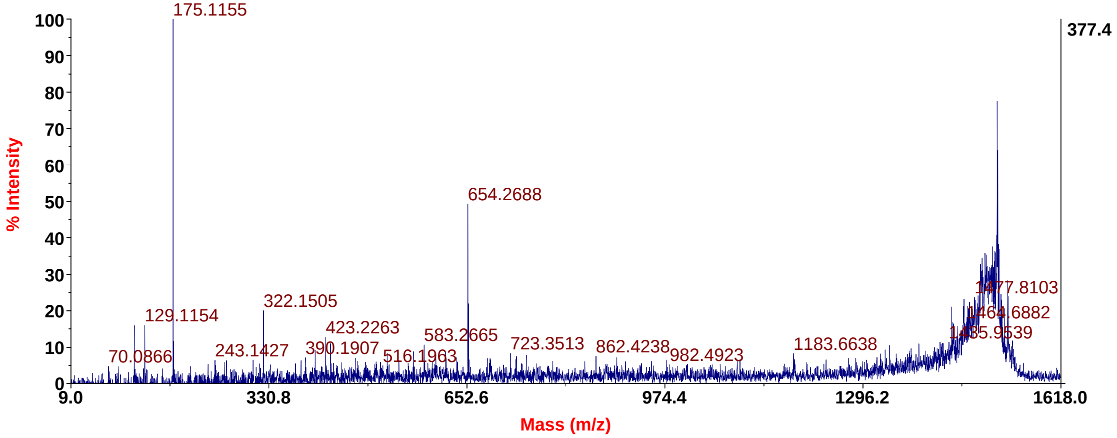


G:\...\C18_MSMS_1531.6827_18.t2d
Acquired:

**4700 MS/MS Precursor 1514.67 Spec #1 MC[BP = 175.1, 233]**

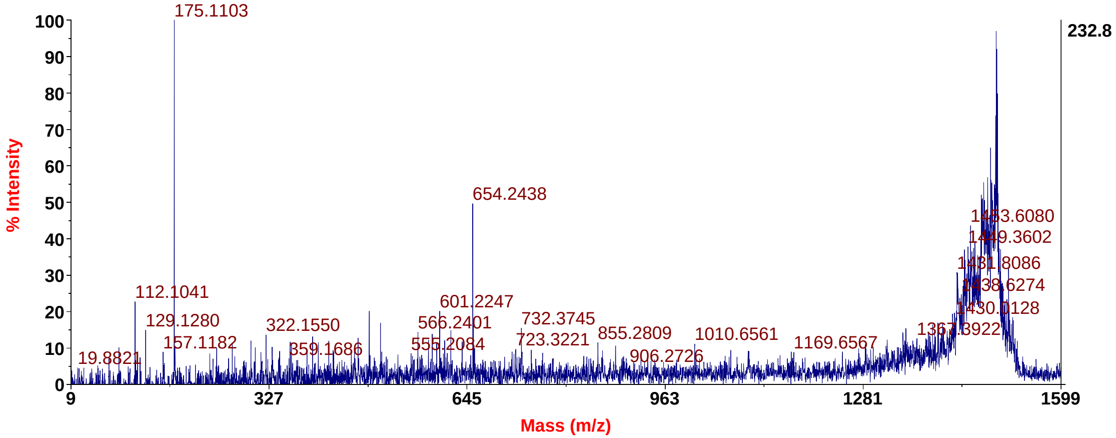


G:\...\C18_MSMS_1514.6671_22.t2d
Acquired:

C18_MSMS_2

4700 MS/MS Precursor 1156.6 Spec #1 MC[BP = 1153.6, 658]

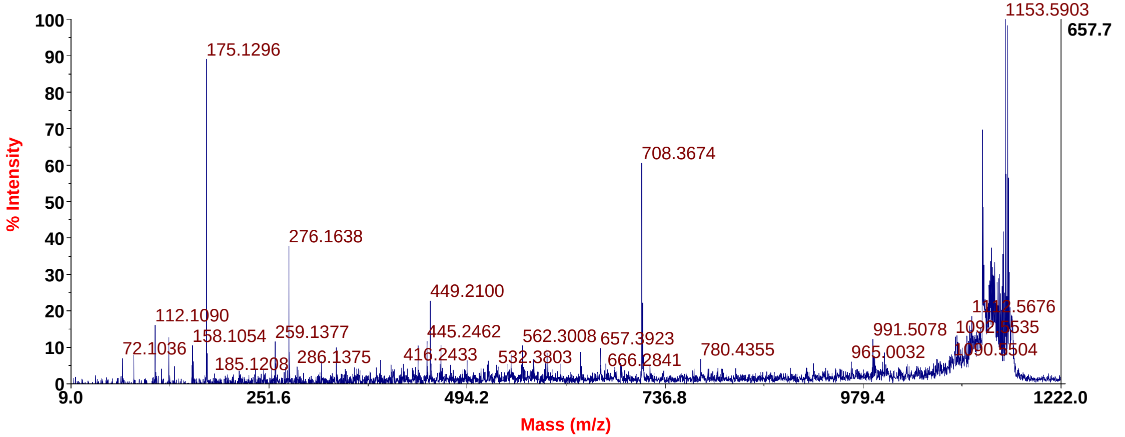


G:\...\C21_MSMS_1156.5970_21.t2d
Acquired:

4700 MS/MS Precursor 1125.61 Spec #1 MC[BP = 1125.6, 4614]

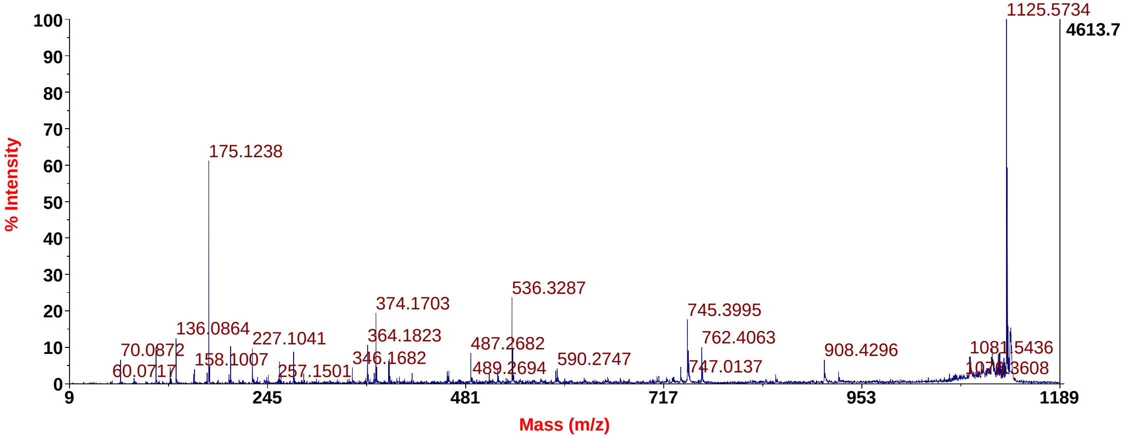


G:\...\C21_MSMS_1125.6141_13.t2d
Acquired:

4700 MS/MS Precursor 1069.46 Spec #1 MC[BP = 1069.4, 1294]

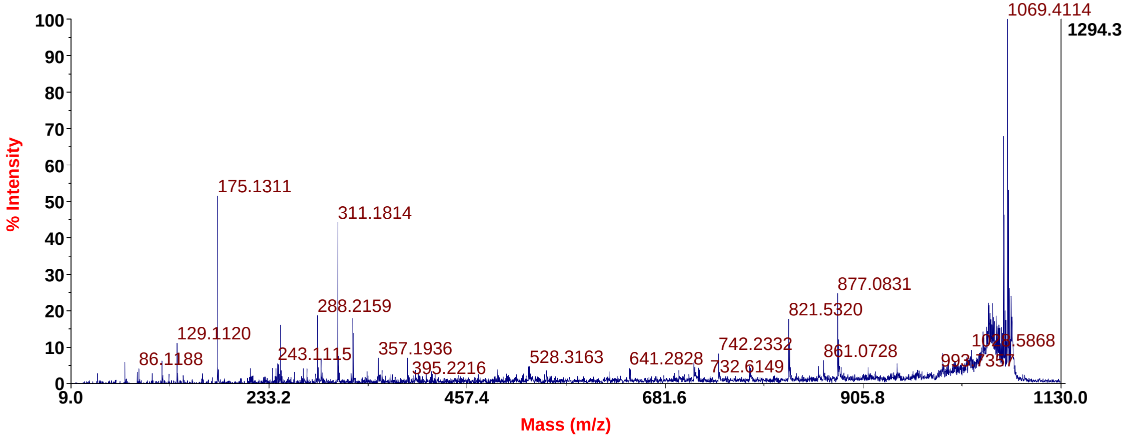


G:\...\C21_MSMS_1069.4606_19.t2d
Acquired:

4700 MS/MS Precursor 1050.51 Spec #1 MC[BP = 1045.6, 962]

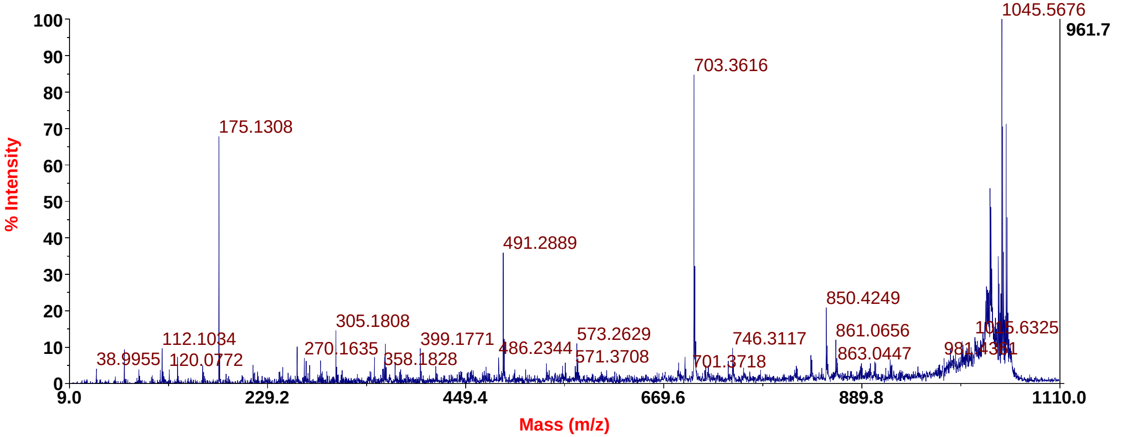


G:\...\C21_MSMS_1050.5127_17.t2d
Acquired:

4700 MS/MS Precursor 870.538 Spec #1 MC[BP = 870.5, 2408]

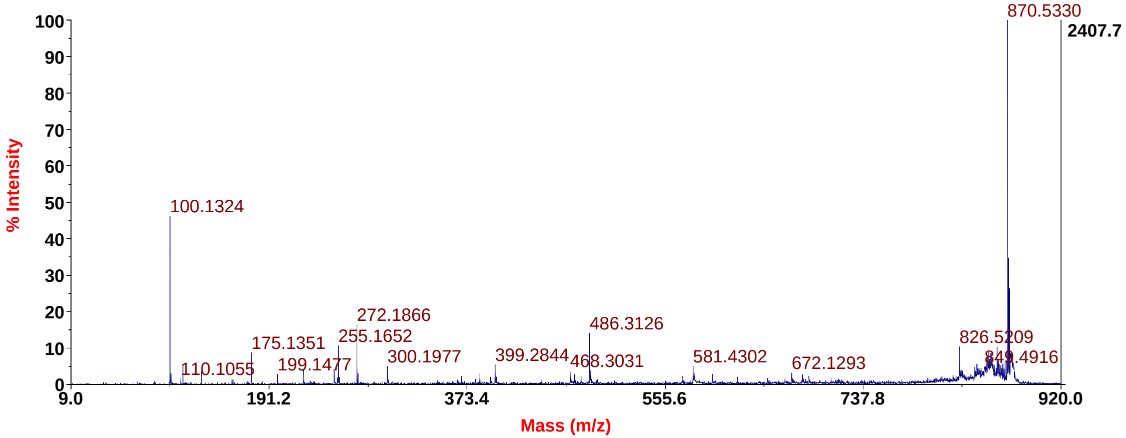


G:\...\C21_MSMS_870.5378_22.t2d
Acquired:

C21_MSMS_1

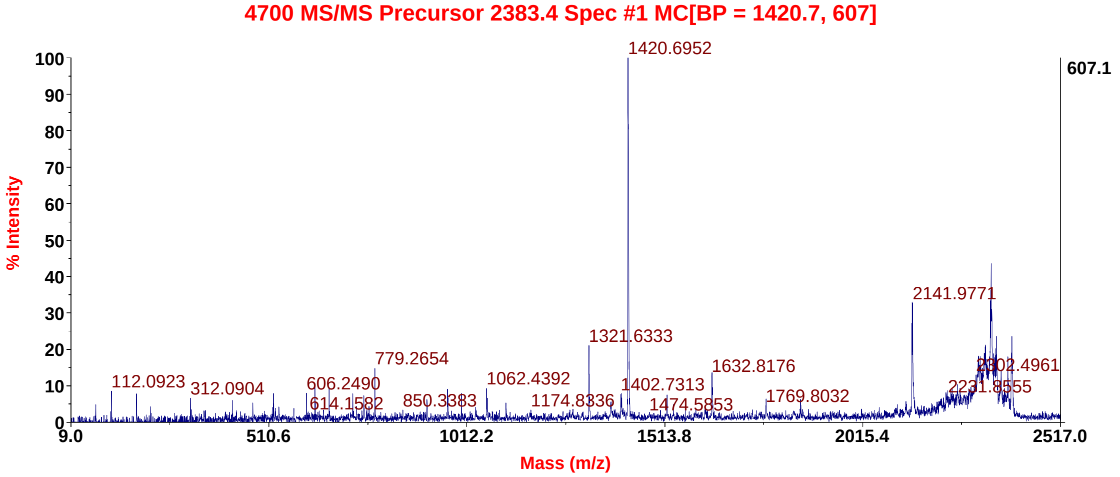


G:\...\C21_MSMS_2383.4028_16.t2d
Acquired:

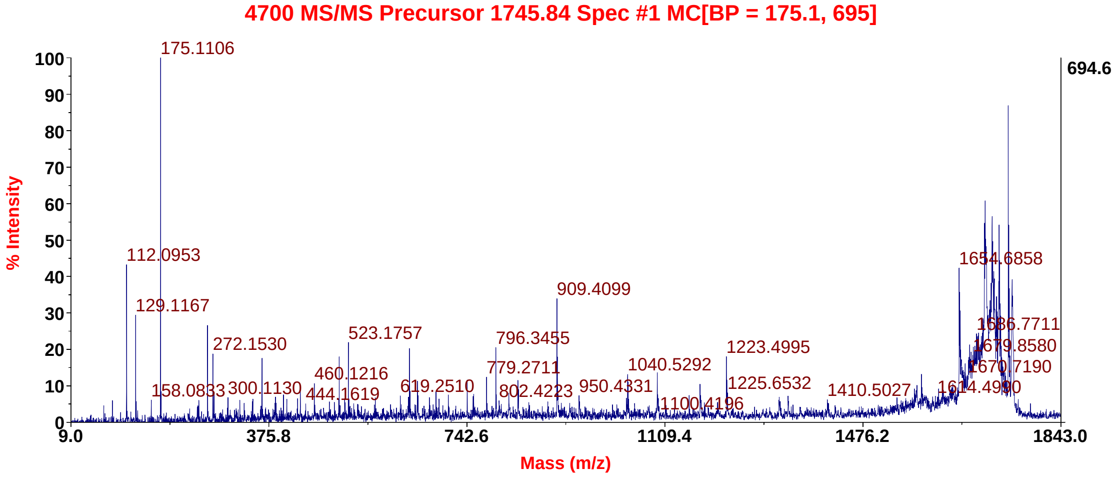


G:\...\C21_MSMS_1745.8405_14.t2d
Acquired:

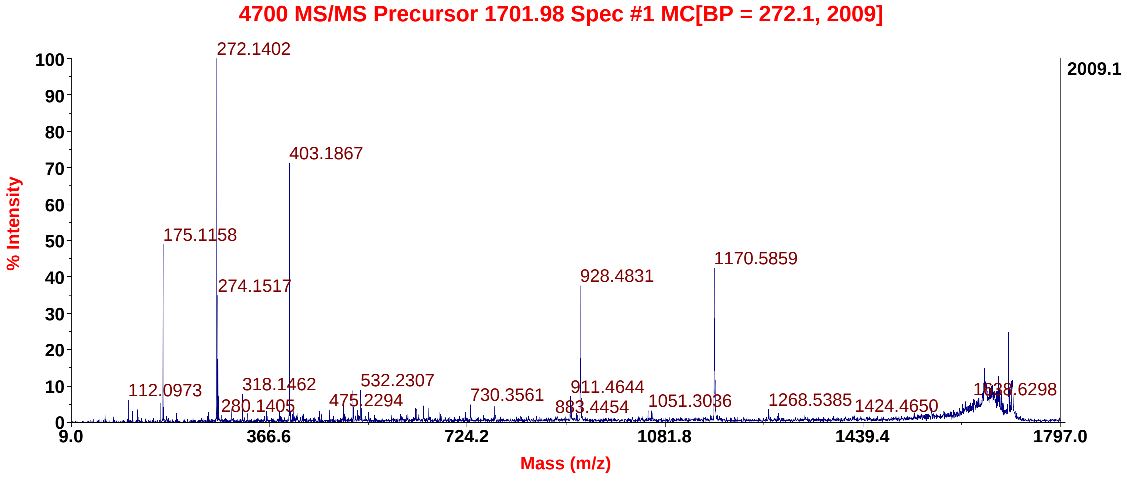


G:\...\C21_MSMS_1701.9822_15.t2d
Acquired:

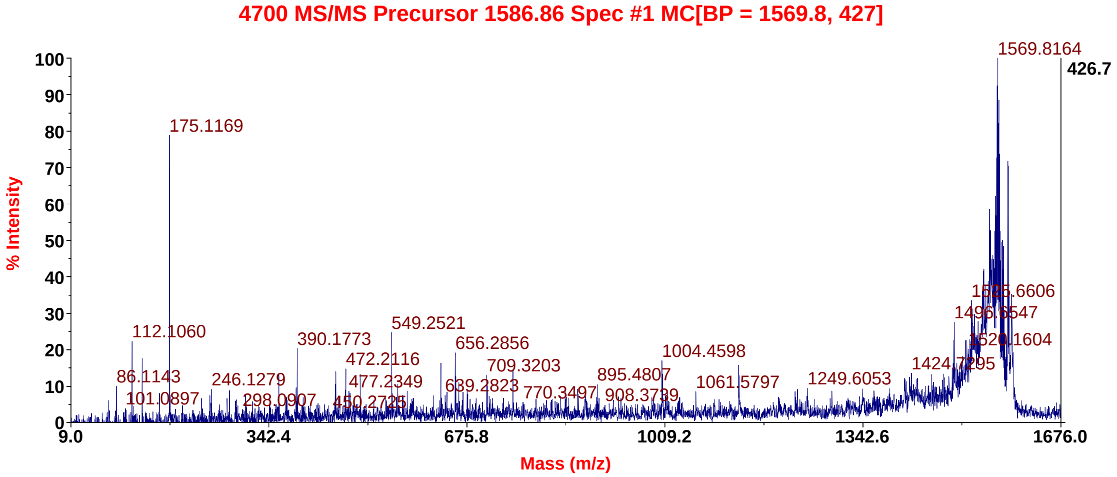


G:\...\C21_MSMS_1586.8553_18.t2d
Acquired:

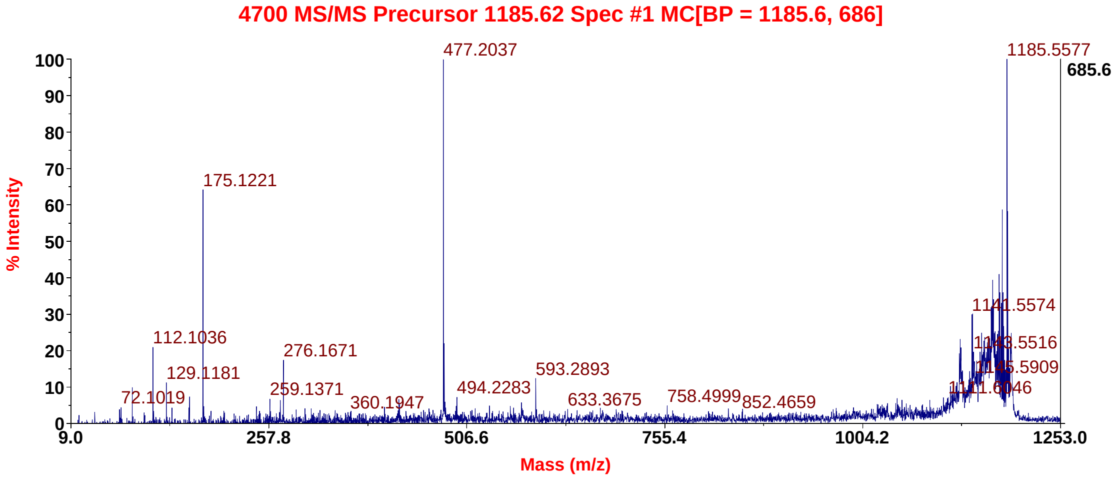


G:\...\C21_MSMS_1185.6232_20.t2d
Acquired:

C21_MSMS_2

4700 MS/MS Precursor 1320.62 Spec #1 MC[BP = 1320.6, 354]

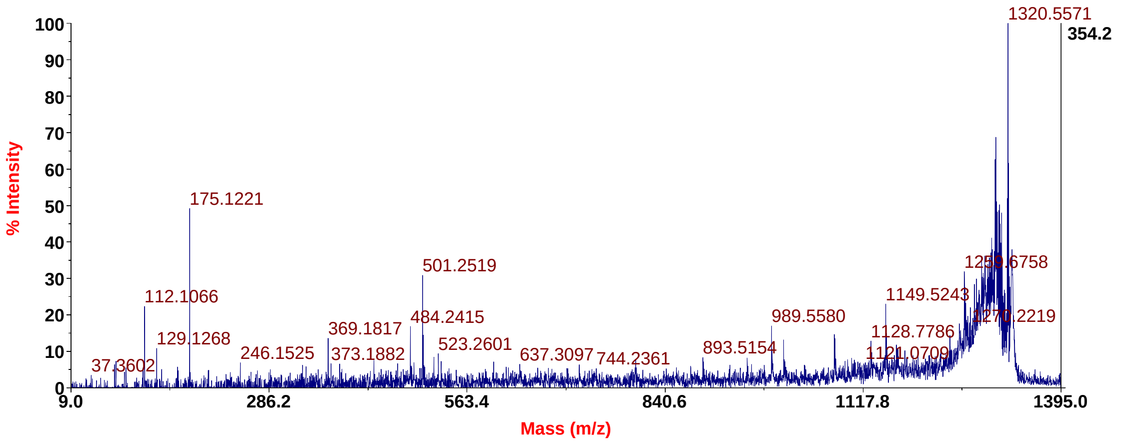


G:\...\C22_MSMS_1320.6204_20.t2d
Acquired:

4700 MS/MS Precursor 1283.59 Spec #1 MC[BP = 1283.6, 438]

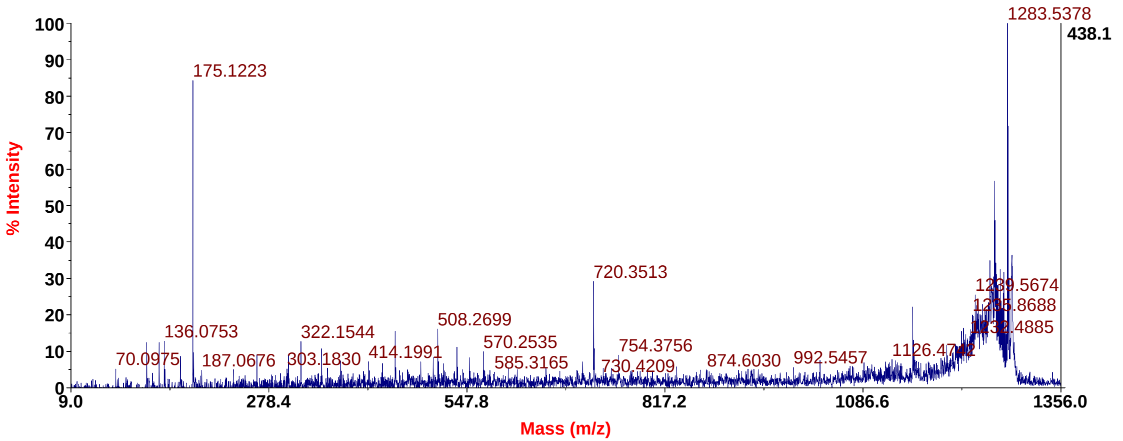


G:\...\C22_MSMS_1283.5857_16.t2d
Acquired:

4700 MS/MS Precursor 1069.45 Spec #1 MC[BP = 1069.4, 2231]

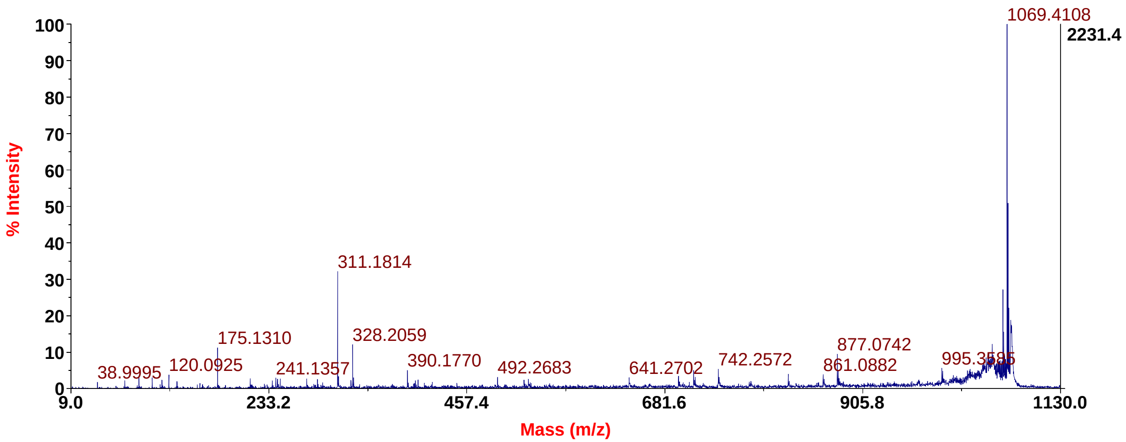


G:\...\C22_MSMS_1069.4487_14.t2d
Acquired:

4700 MS/MS Precursor 917.287 Spec #1 MC[BP = 899.3, 3555]

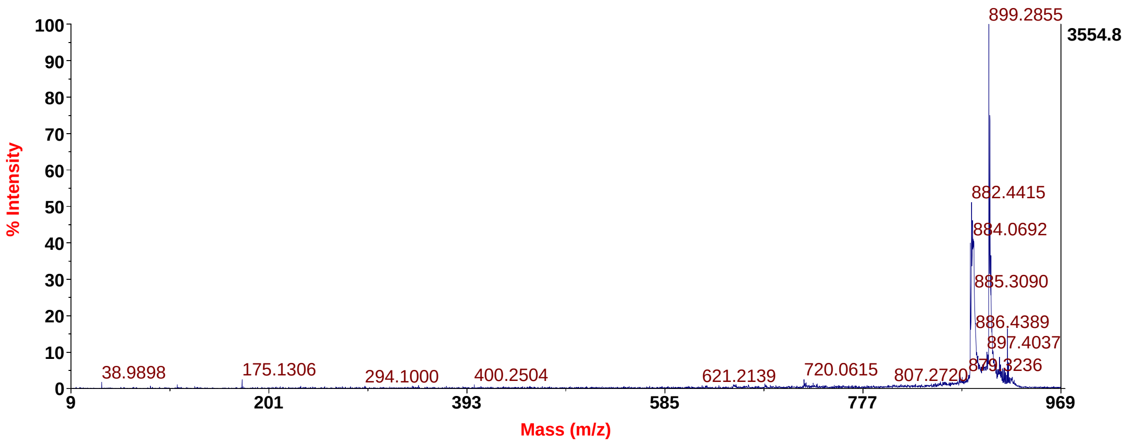


G:\...\C22_MSMS_917.2870_19.t2d
Acquired:

4700 MS/MS Precursor 870.541 Spec #1 MC[BP = 870.5, 1840]

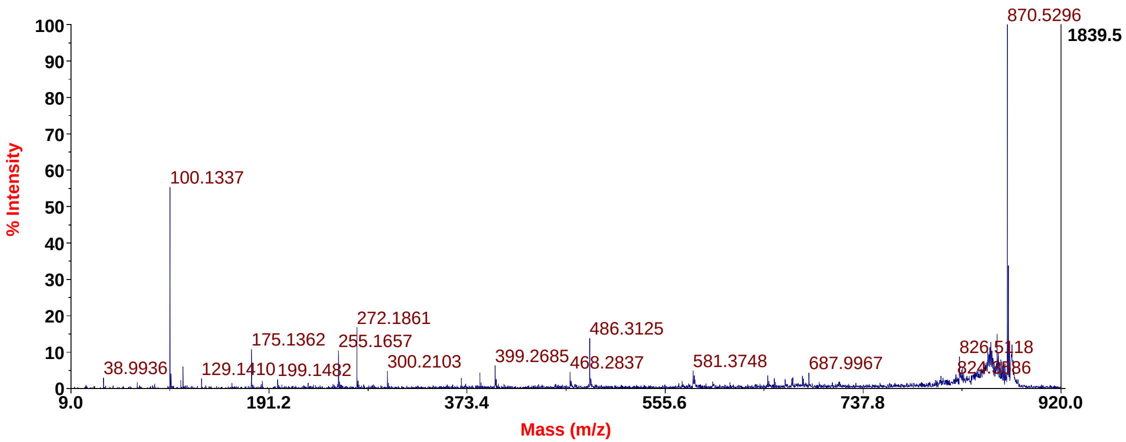


G:\...\C22_MSMS_870.5411_18.t2d
Acquired:

C22_MSMS_1

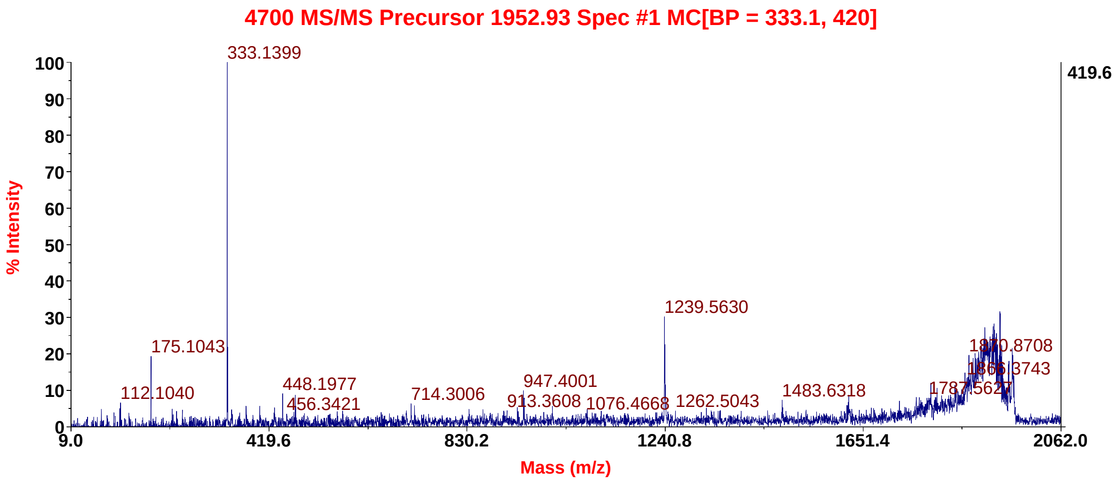


G:\...\C22_MSMS_1952.9259_22.t2d
Acquired:

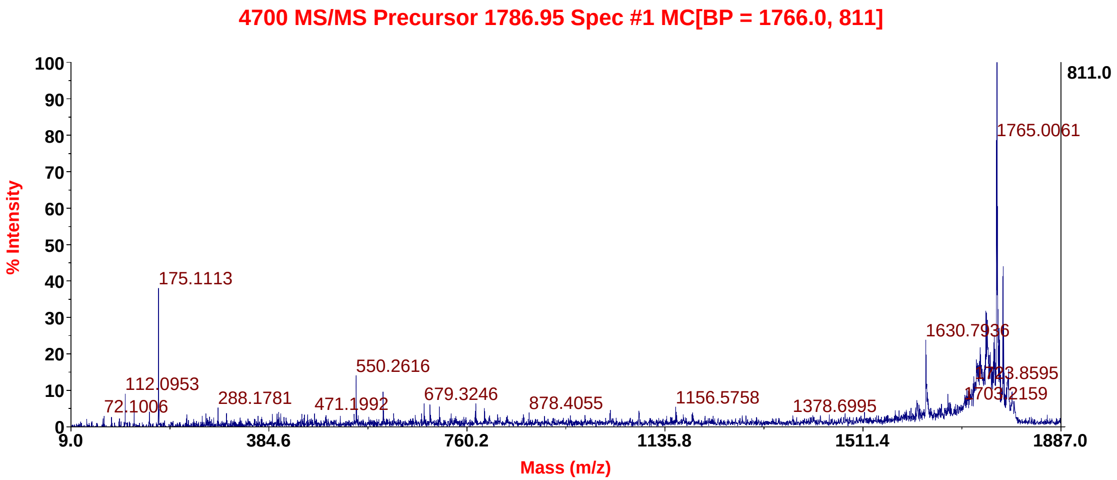


G:\...\C22_MSMS_1786.9526_21.t2d
Acquired:

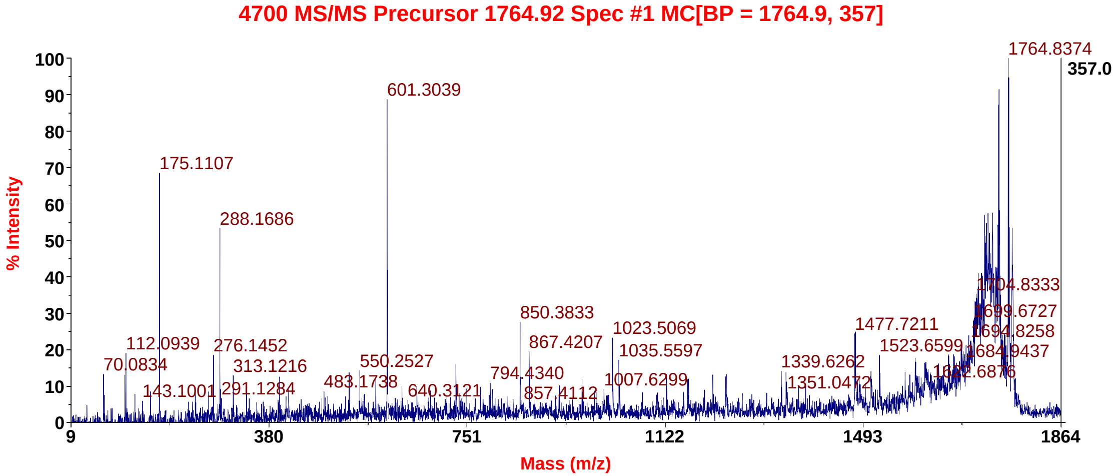


G:\...\C22_MSMS_1764.9220_13.t2d
Acquired:

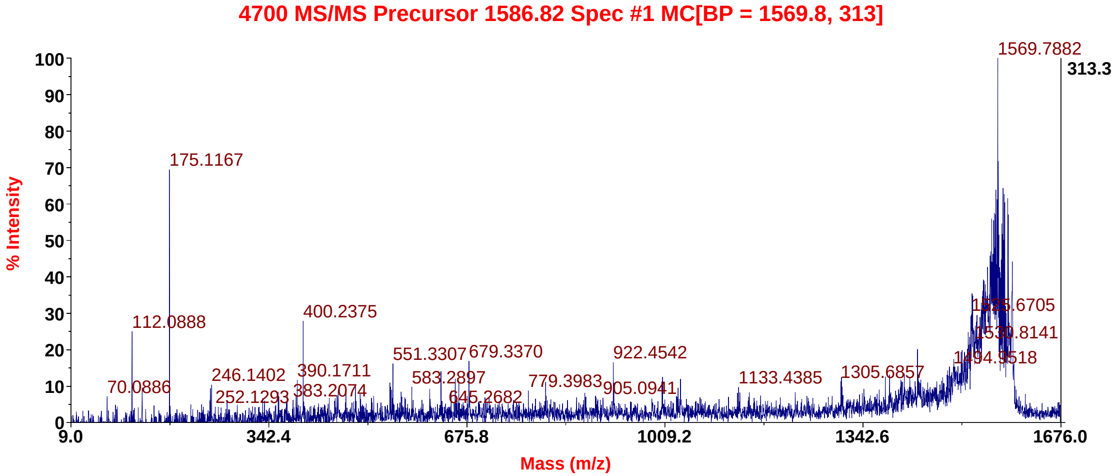


G:\...\C22_MSMS_1586.8243_17.t2d
Acquired:

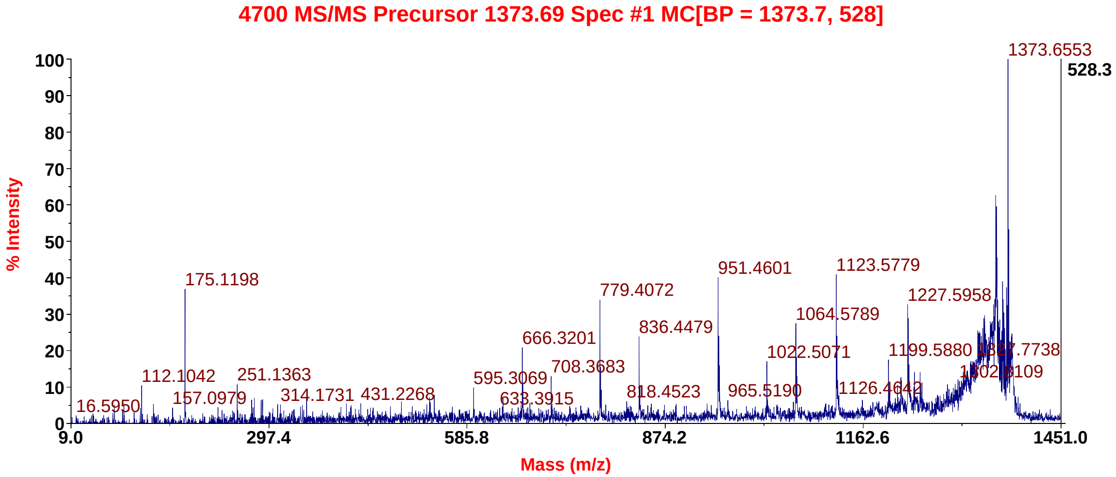


G:\...\C22_MSMS_1373.6915_15.t2d
Acquired:

C22_MSMS_2

4700 MS/MS Precursor 1320.66 Spec #1 MC[BP = 1308.7, 208]

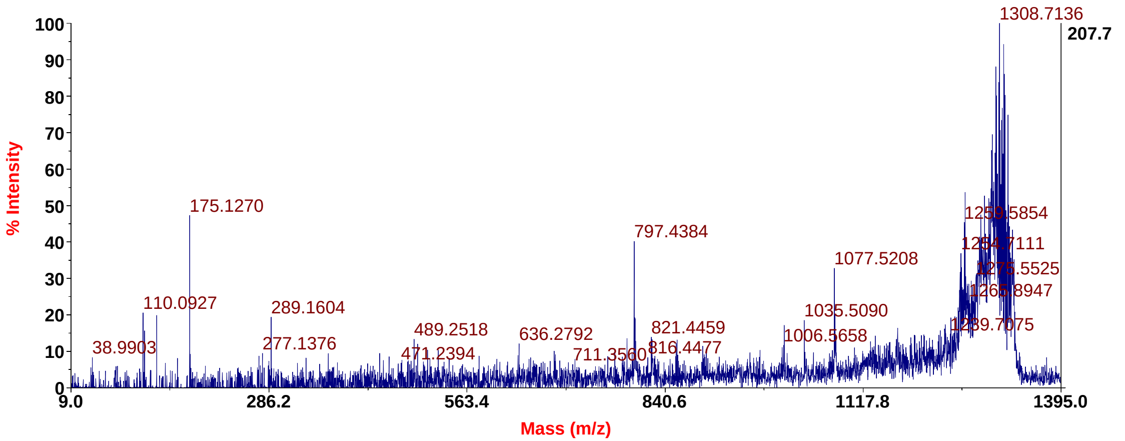


G:\...\C23_MSMS_1320.6620_22.t2d
Acquired:

4700 MS/MS Precursor 1302.7 Spec #1 MC[BP = 1296.6, 370]

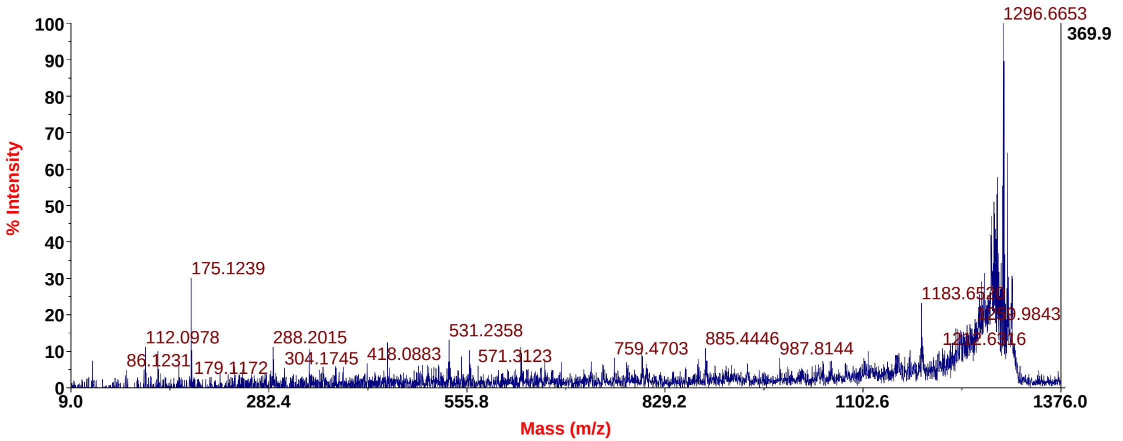


G:\...\C23_MSMS_1302.6974_20.t2d
Acquired:

4700 MS/MS Precursor 1283.59 Spec #1 MC[BP = 1283.5, 350]

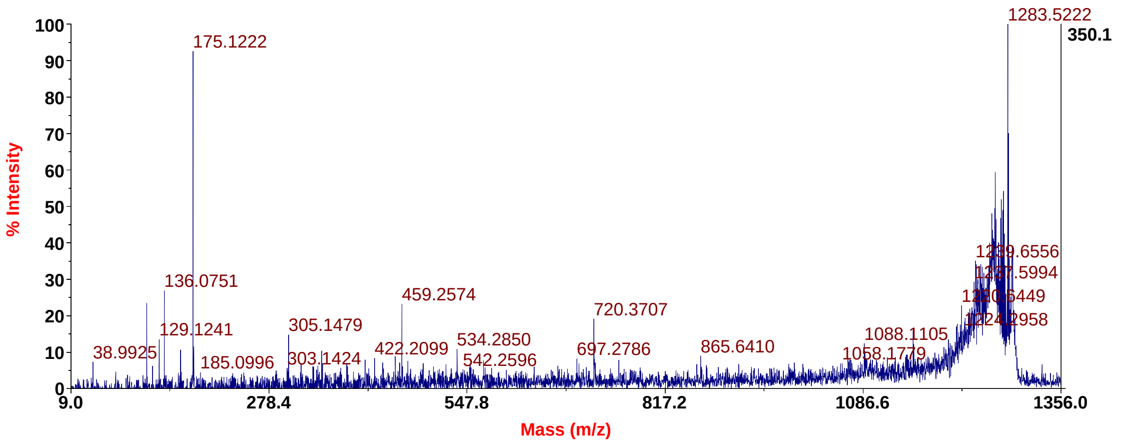


G:\...\C23_MSMS_1283.5897_18.t2d
Acquired:

4700 MS/MS Precursor 1069.45 Spec #1 MC[BP = 1069.4, 2621]

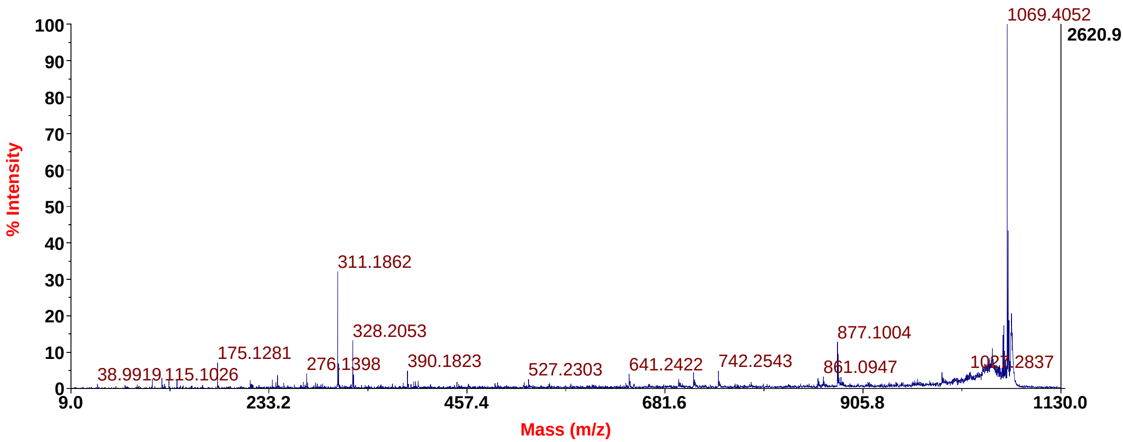


G:\...\C23_MSMS_1069.4485_14.t2d
Acquired:

4700 MS/MS Precursor 870.549 Spec #1 MC[BP = 870.5, 2762]

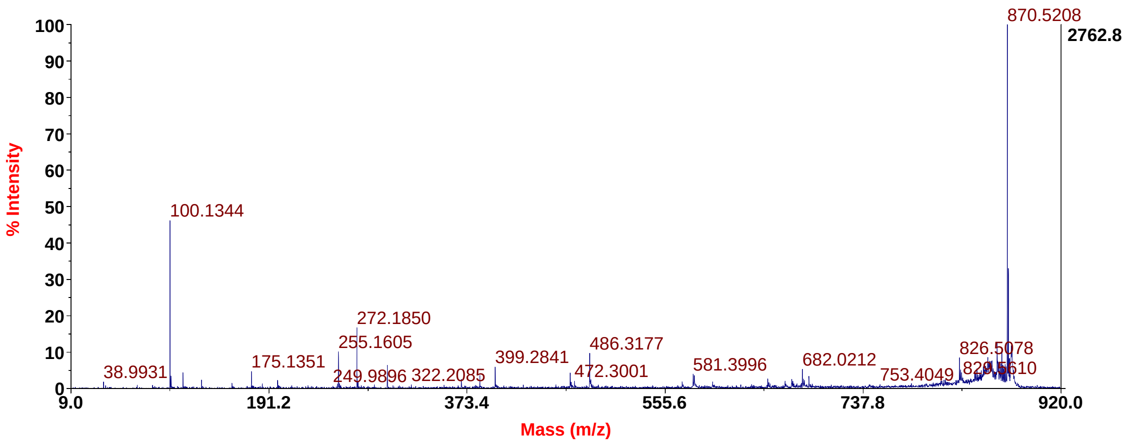


G:\...\C23_MSMS_870.5490_16.t2d
Acquired:

C23_MSMS_1

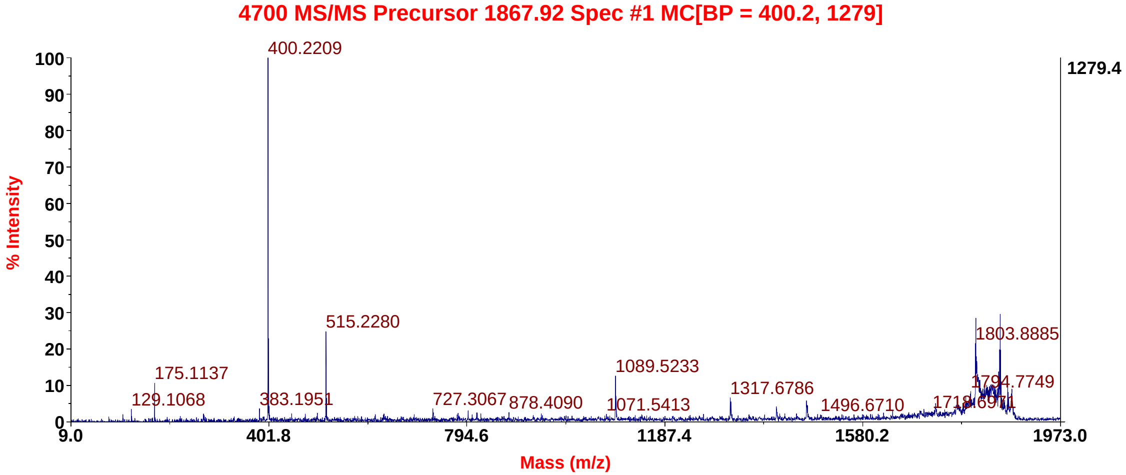


G:\...\C23_MSMS_1867.9232_17.t2d
Acquired:

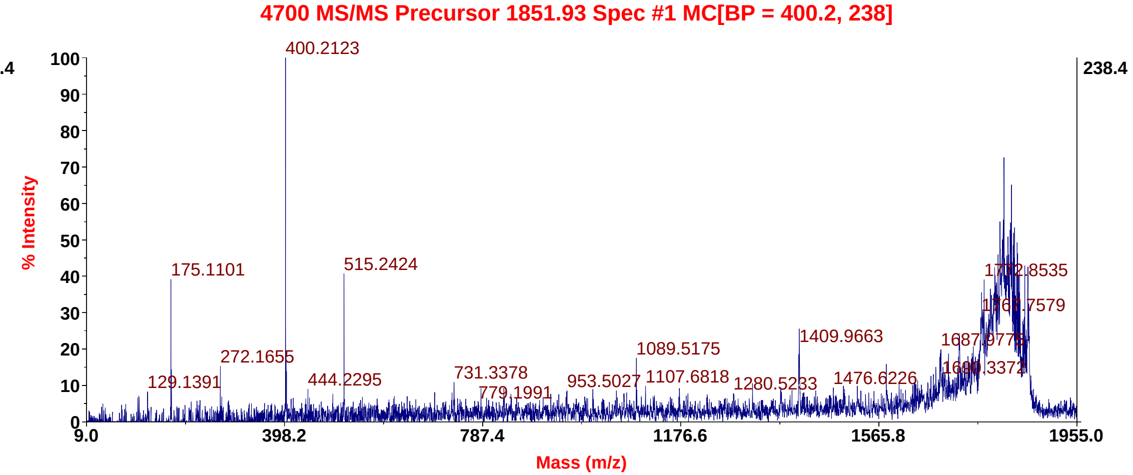


G:\...\C23_MSMS_1851.9325_21.t2d
Acquired:

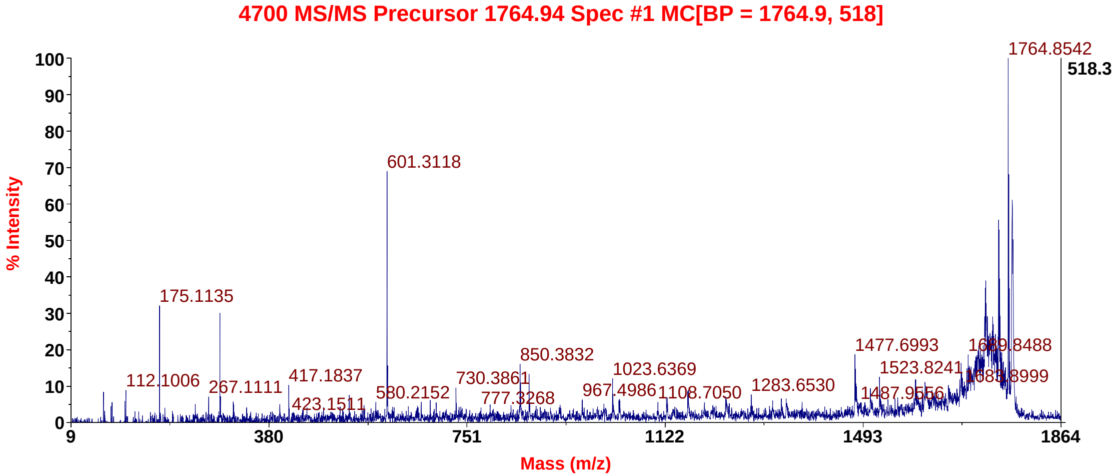


G:\...\C23_MSMS_1764.9413_13.t2d
Acquired:

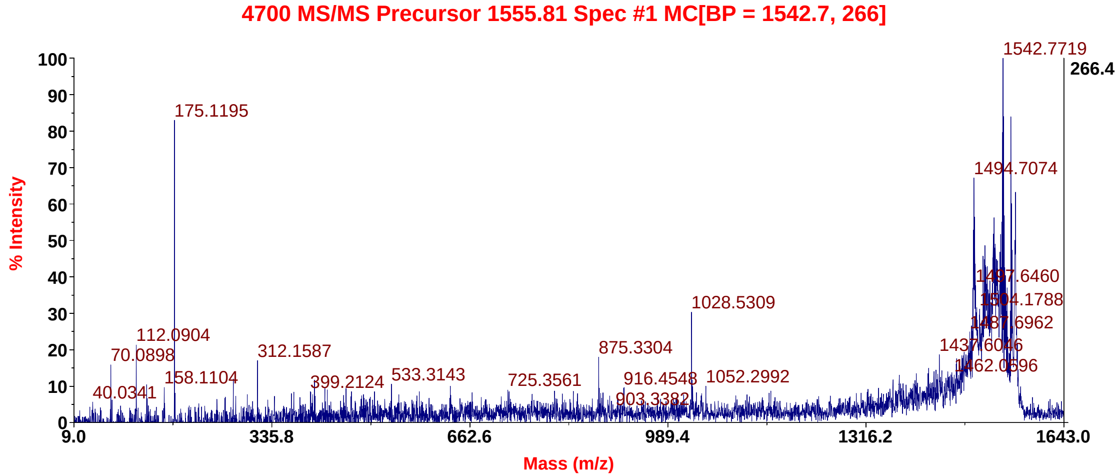


G:\...\C23_MSMS_1555.8104_19.t2d
Acquired:

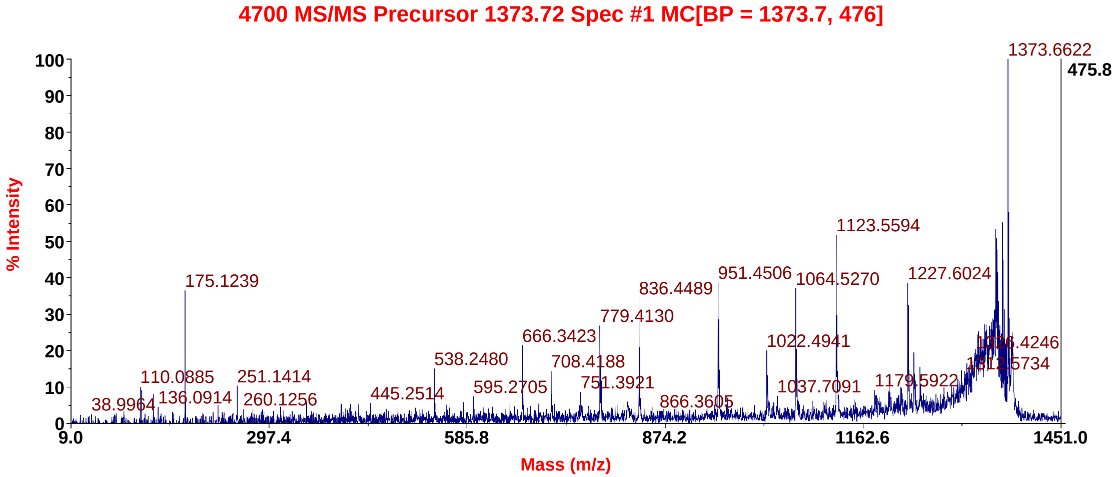


G:\...\C23_MSMS_1373.7177_15.t2d
Acquired:

C23_MSMS_2

**4700 MS/MS Precursor 1069.45 Spec #1 MC[BP = 1069.4, 5277]**

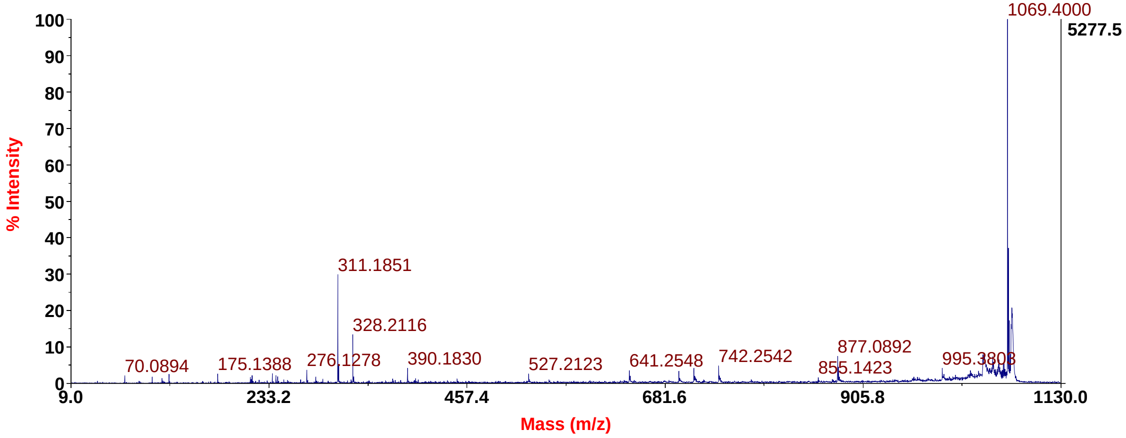


G:\...\D1_MSMS_1069.4513_13.t2d
Acquired:

**4700 MS/MS Precursor 881.271 Spec #1 MC[BP = 881.2, 1391]**

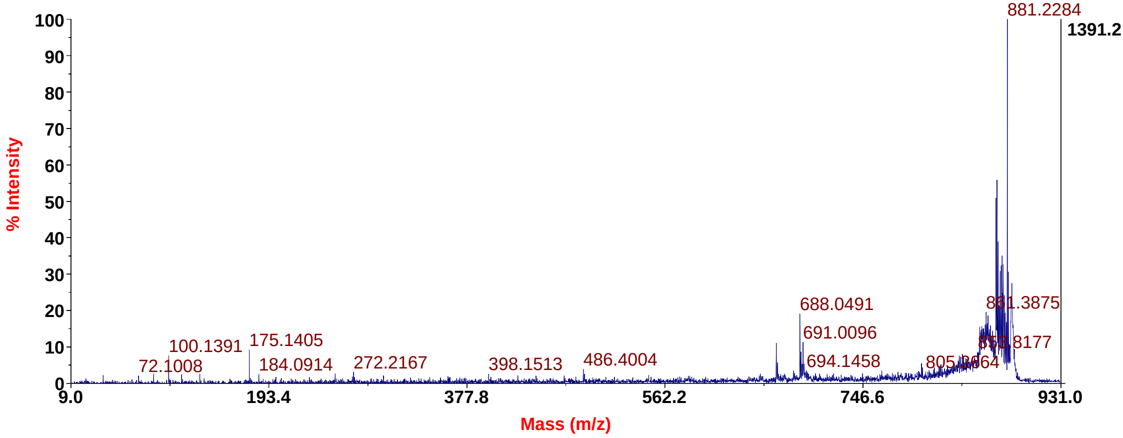


G:\...\D1_MSMS_881.2711_16.t2d
Acquired:

**4700 MS/MS Precursor 870.549 Spec #1 MC[BP = 870.5, 2383]**

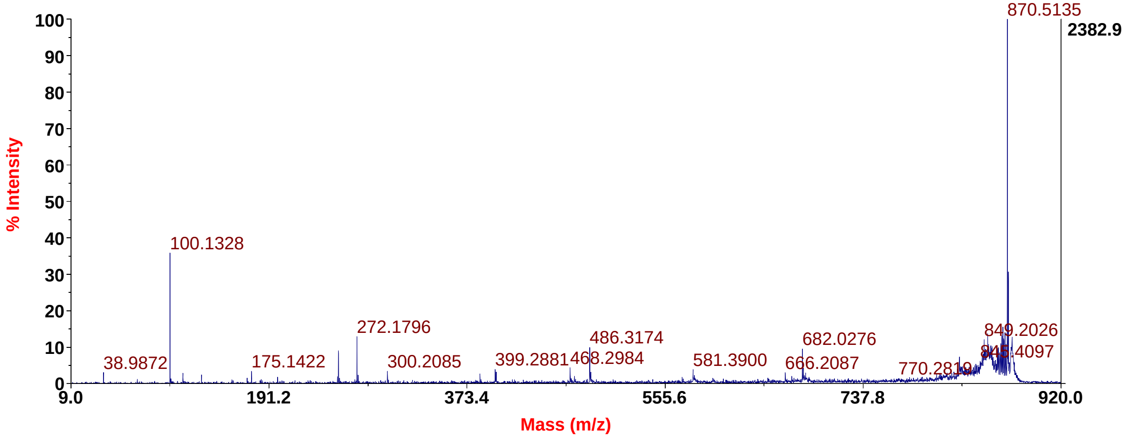


G:\...\D1_MSMS_870.5490_19.t2d
Acquired:

**4700 MS/MS Precursor 829.246 Spec #1 MC[BP = 829.2, 5702]**

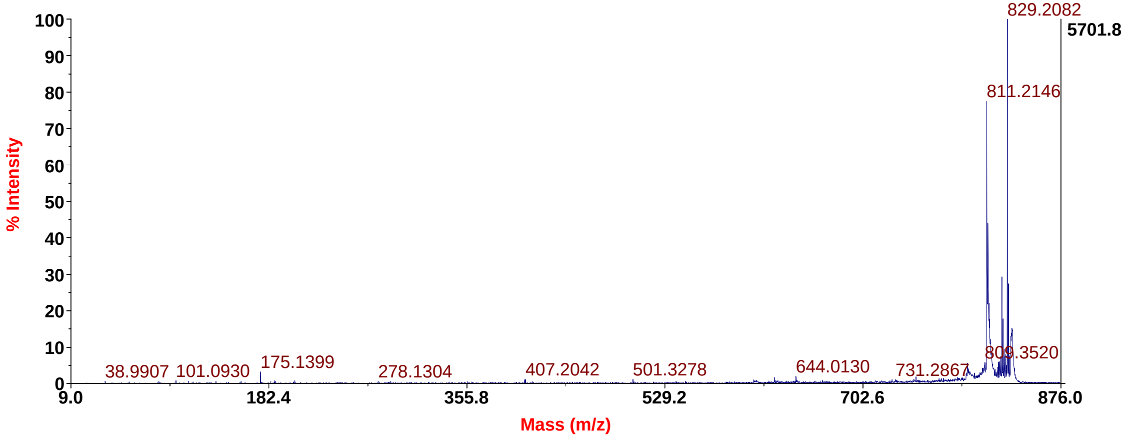


G:\...\D1_MSMS_829.2465_14.t2d
Acquired:

**4700 MS/MS Precursor 824.414 Spec #1 MC[BP = 824.4, 738]**

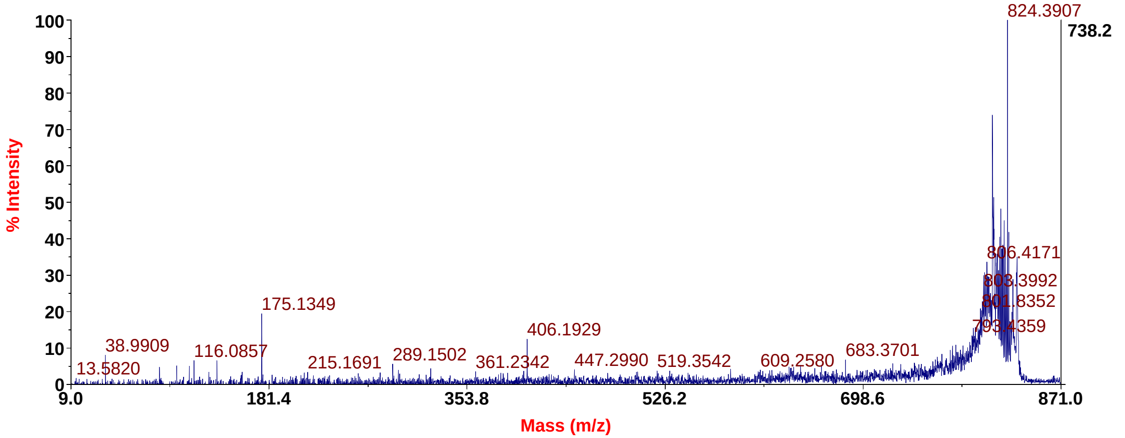


G:\...\D1_MSMS_824.4144_21.t2d
Acquired:

# D1_MSMS_1

**4700 MS/MS Precursor 2261.15 Spec #1 MC[BP = 928.4, 133]**

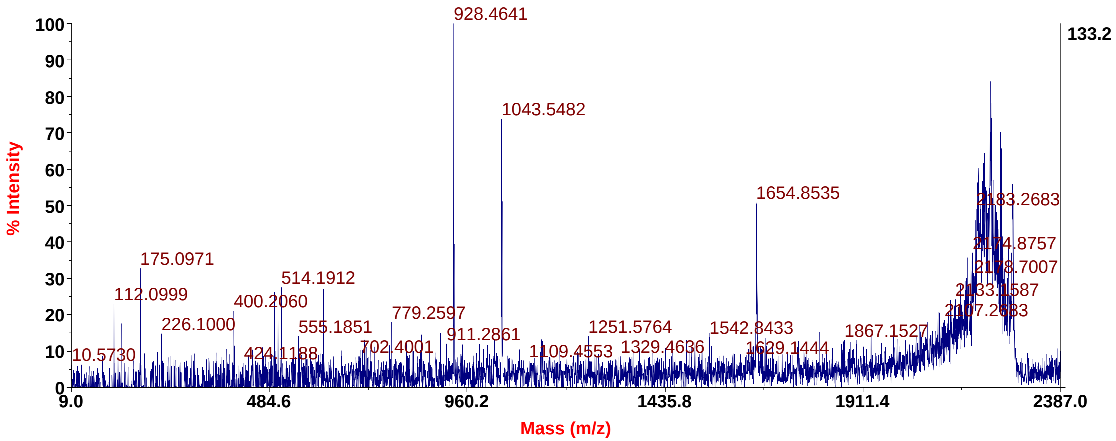


G:\...\D1_MSMS_2261.1531_20.t2d
Acquired:

**4700 MS/MS Precursor 2105.04 Spec #1 MC[BP = 2064.1, 109]**

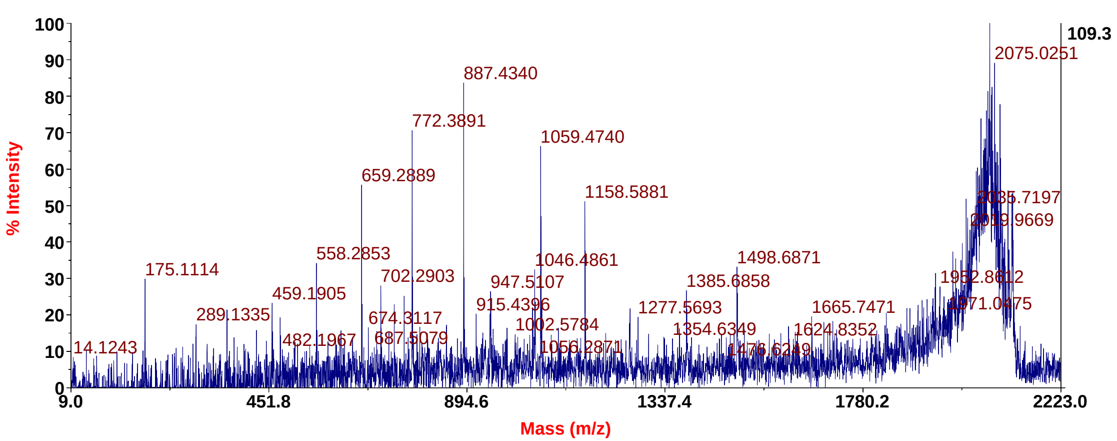


G:\...\D1_MSMS_2105.0444_18.t2d
Acquired:

**4700 MS/MS Precursor 1764.92 Spec #1 MC[BP = 1772.4, 347]**

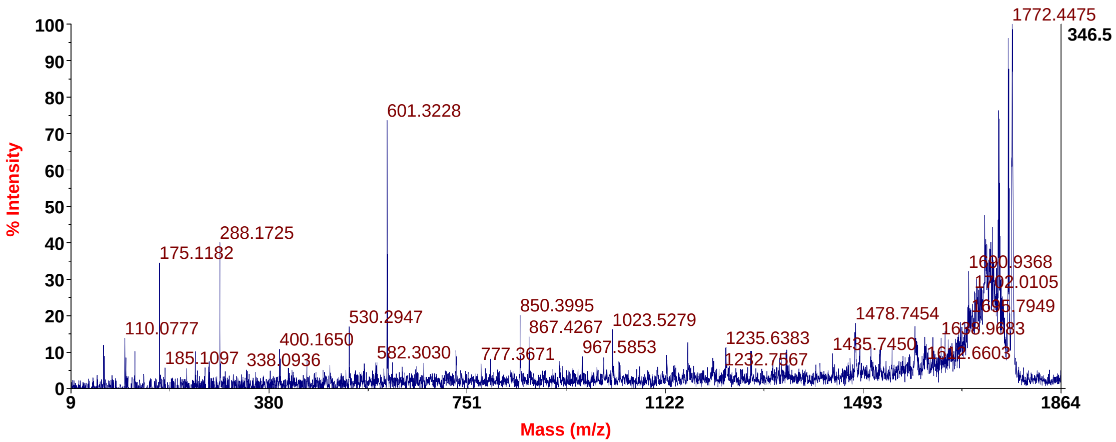


G:\...\D1_MSMS_1764.9210_15.t2d
Acquired:

**4700 MS/MS Precursor 1373.7 Spec #1 MC[BP = 1365.6, 243]**

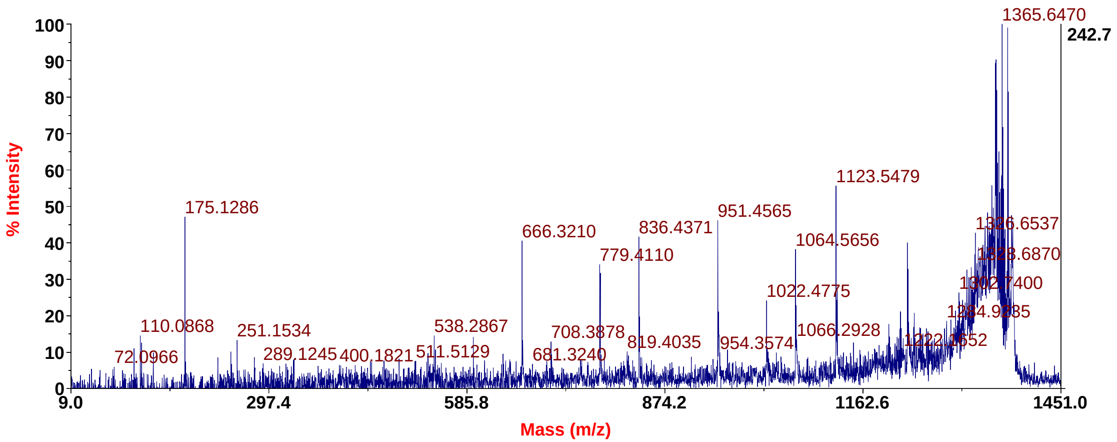


G:\...\D1_MSMS_1373.7015_17.t2d
Acquired:

**4700 MS/MS Precursor 1283.63 Spec #1 MC[BP = 720.4, 230]**

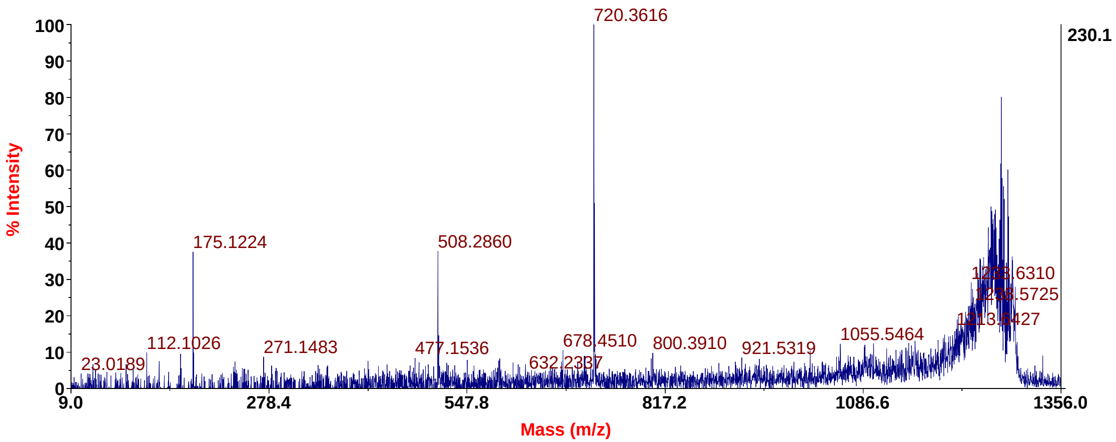


G:\...\D1_MSMS_1283.6307_22.t2d
Acquired:

# D1_MSMS_2

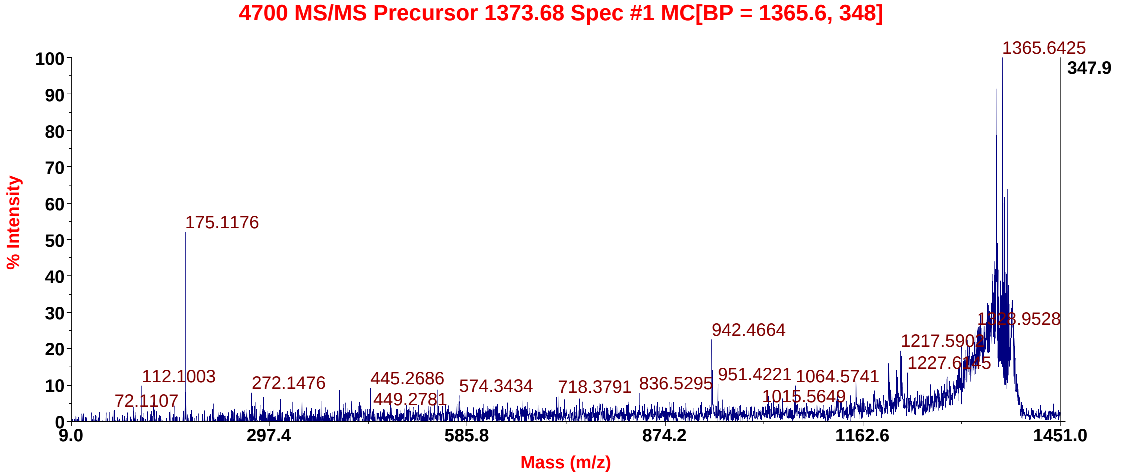


C:\...\D4_MSMS_1373.6793_22.t2d
Acquired:

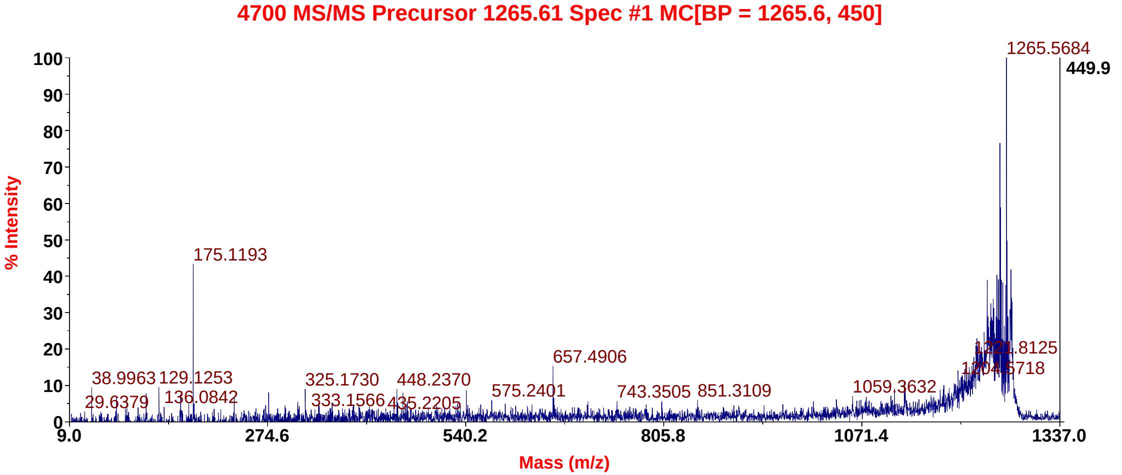


C:\...\D4_MSMS_1265.6061_19.t2d
Acquired:

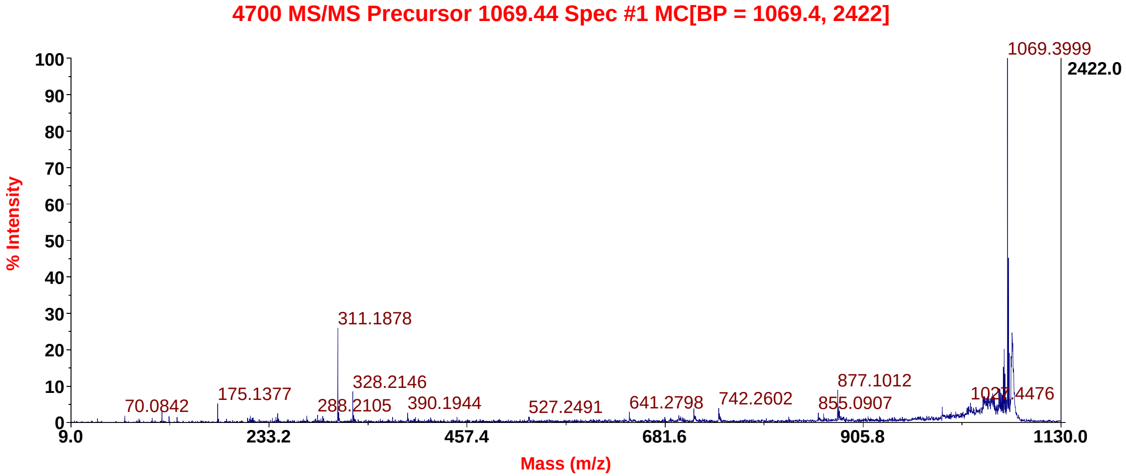


C:\...\D4_MSMS_1069.4435_13.t2d
Acquired:

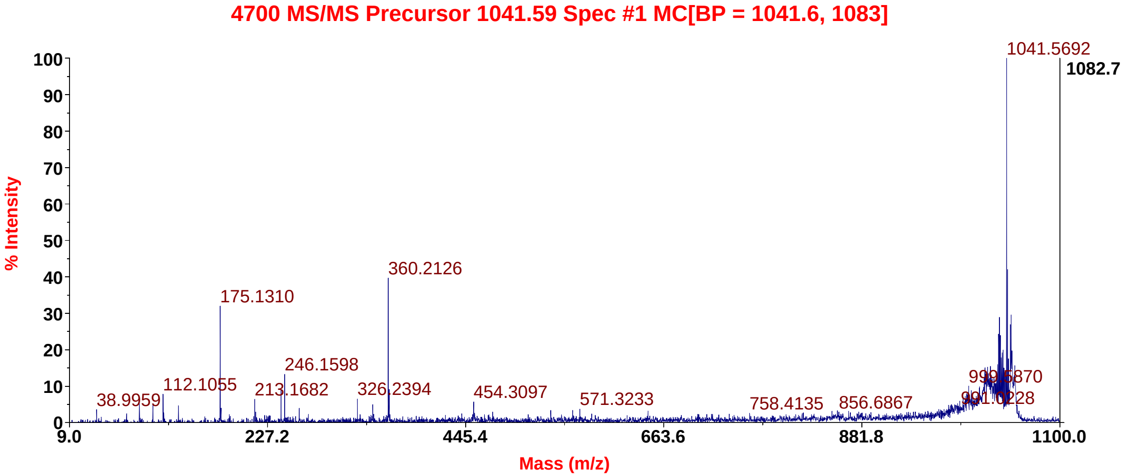


C:\...\D4_MSMS_1041.5940_17.t2d
Acquired:

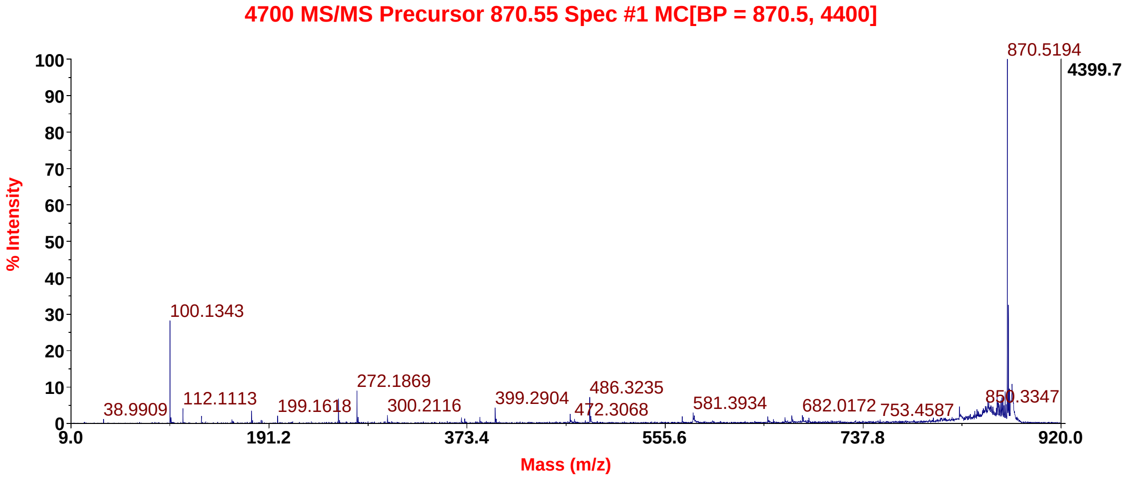


C:\...\D4_MSMS_870.5504_14.t2d
Acquired:

D4_MSMS_1

4700 MS/MS Precursor 1727.86 Spec #1 MC[BP = 1174.7, 339]

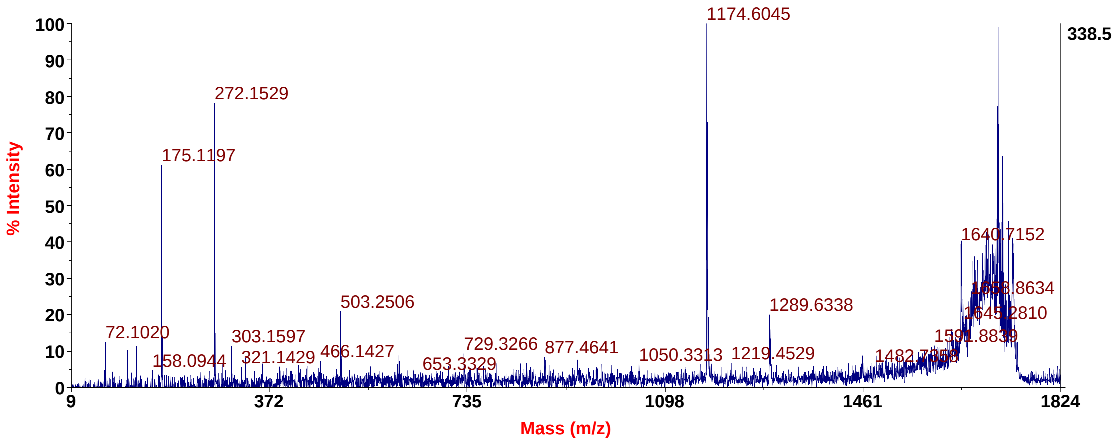


C:\...\D4_MSMS_1727.8623_20.t2d
Acquired:

4700 MS/MS Precursor 1664.75 Spec #1 MC[BP = 946.5, 446]

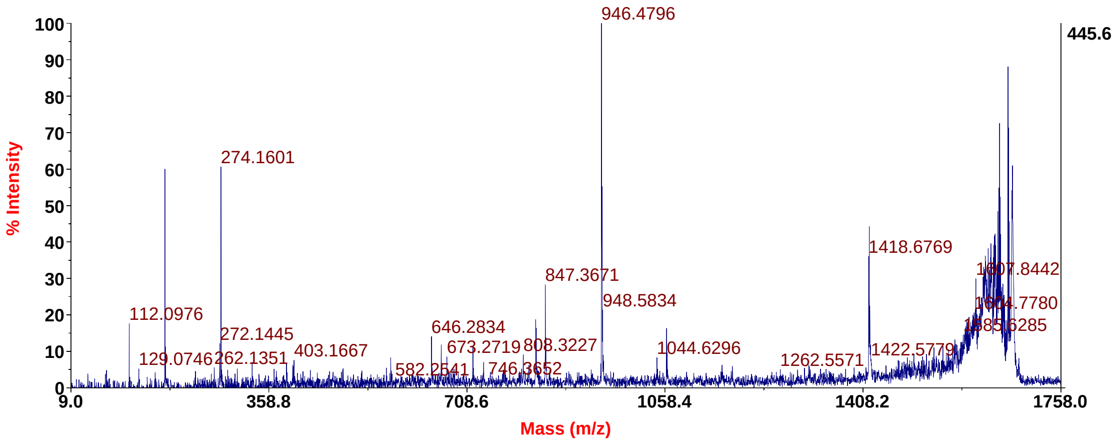


C:\...\D4_MSMS_1664.7468_15.t2d
Acquired:

4700 MS/MS Precursor 1640.71 Spec #1 MC[BP = 1623.6, 547]

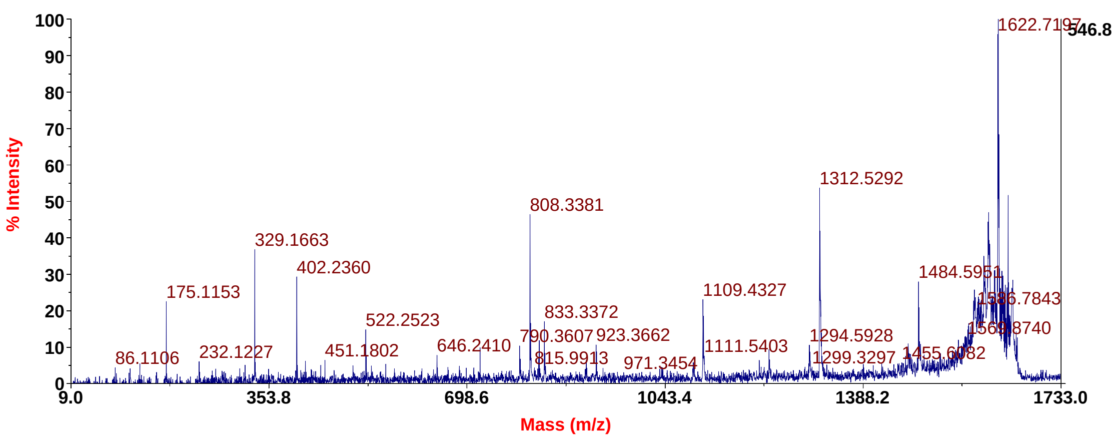


C:\...\D4_MSMS_1640.7118_18.t2d
Acquired:

4700 MS/MS Precursor 1586.8 Spec #1 MC[BP = 1569.8, 492]

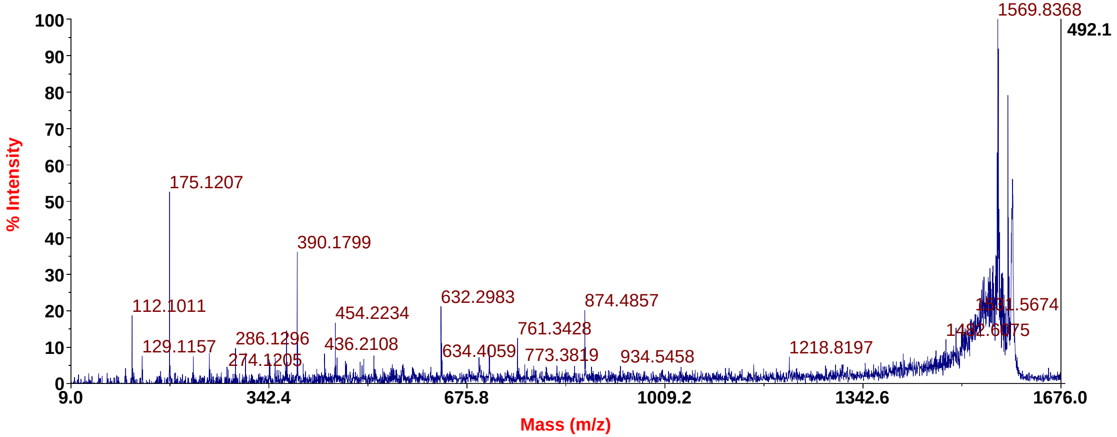


C:\...\D4_MSMS_1586.8022_16.t2d
Acquired:

4700 MS/MS Precursor 1412.71 Spec #1 MC[BP = 500.2, 185]

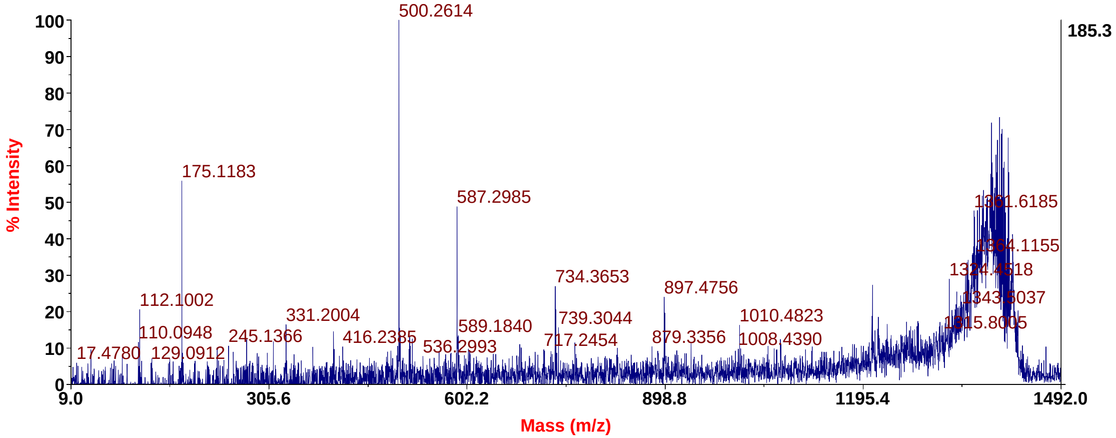


C:\...\D4_MSMS_1412.7148_21.t2d
Acquired:

D4_MSMS_2

4700 MS/MS Precursor 870.559 Spec #1 MC[BP = 870.5, 3409]

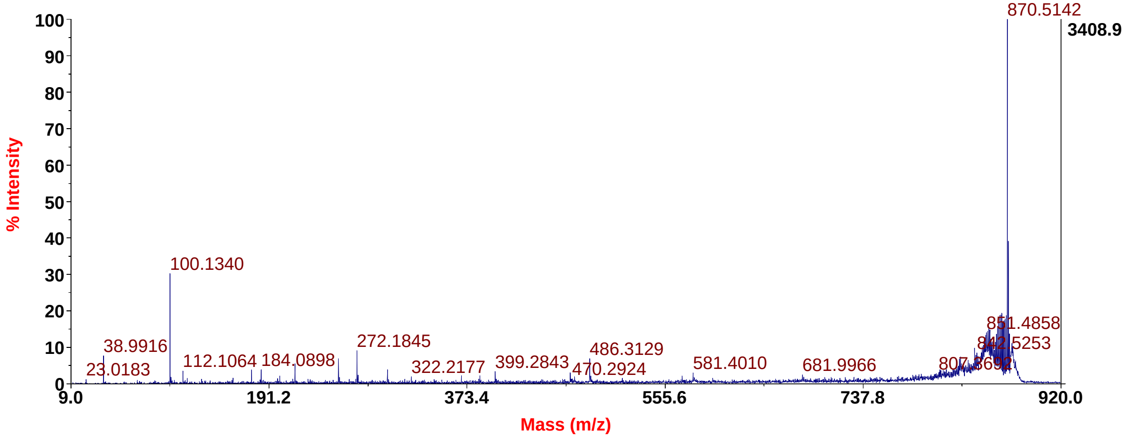


C:\...\D6_MSMS_870.5590_17.t2d
Acquired:

4700 MS/MS Precursor 840.446 Spec #1 MC[BP = 776.4, 3191]

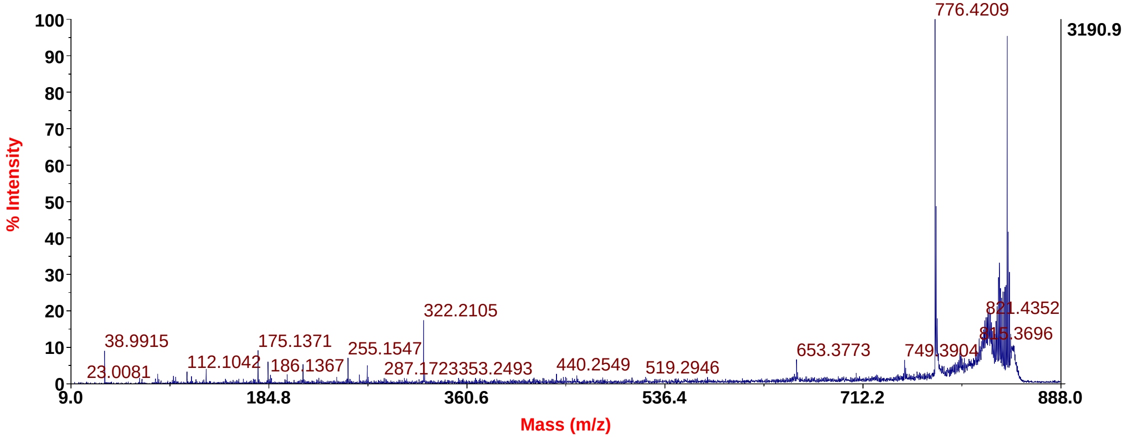


C:\...\D6_MSMS_840.4464_15.t2d
Acquired:

4700 MS/MS Precursor 830.424 Spec #1 MC[BP = 824.4, 1789]

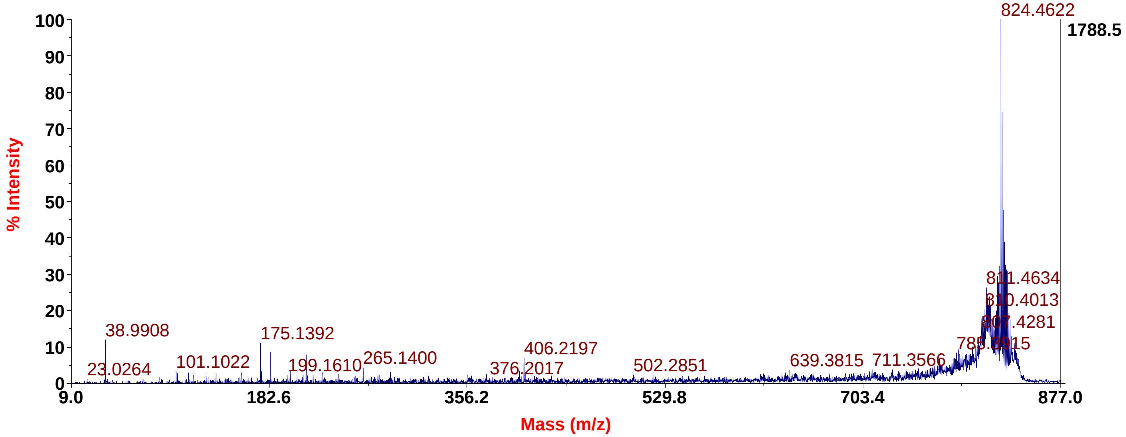


C:\...\D6_MSMS_830.4238_22.t2d
Acquired:

4700 MS/MS Precursor 824.451 Spec #1 MC[BP = 824.4, 1974]

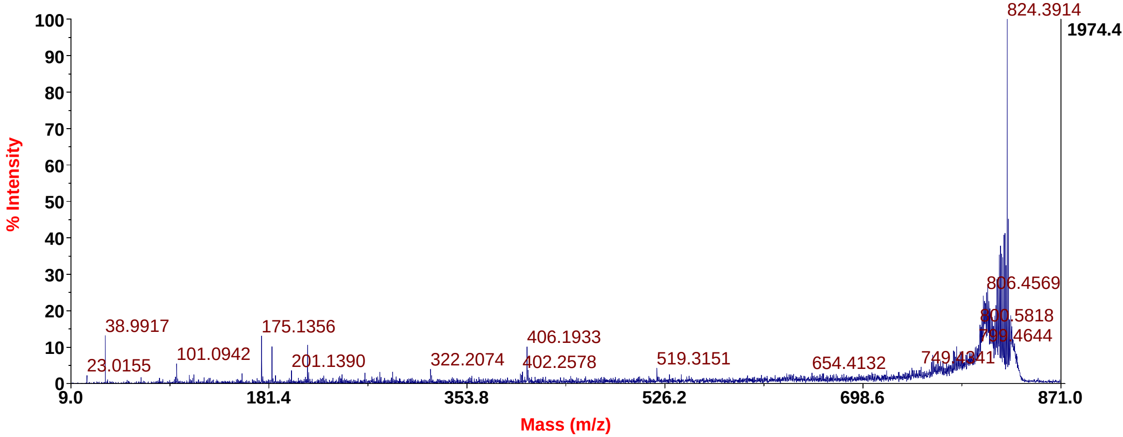


C:\...\D6_MSMS_824.4510_20.t2d
Acquired:

4700 MS/MS Precursor 800.434 Spec #1 MC[BP = 797.4, 1142]

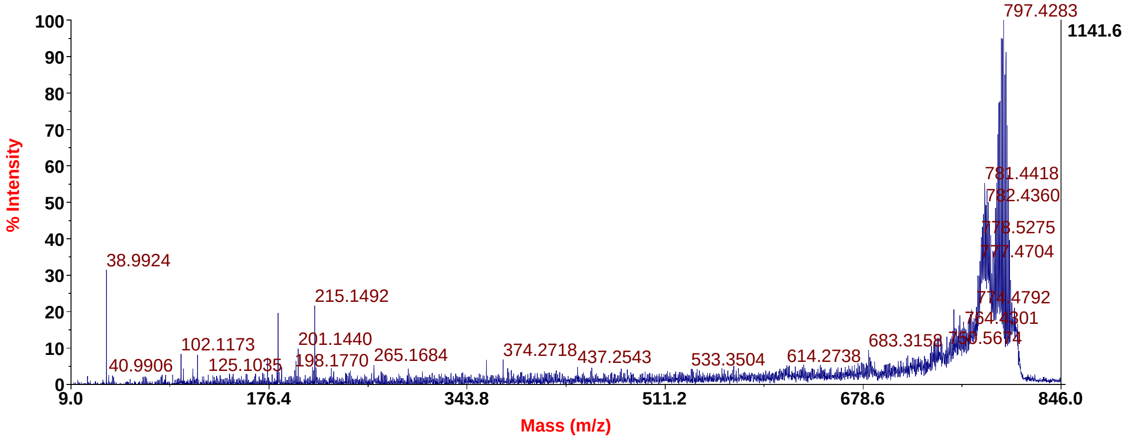


C:\...\D6_MSMS_800.4340_18.t2d
Acquired:

D6_MSMS_1

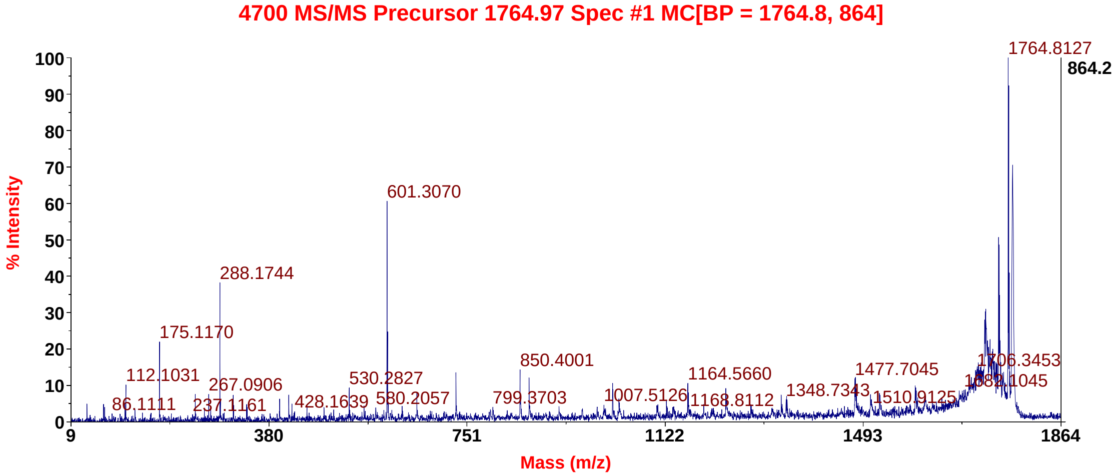


C:\...\D6_MSMS_1764.9702_13.t2d
Acquired:

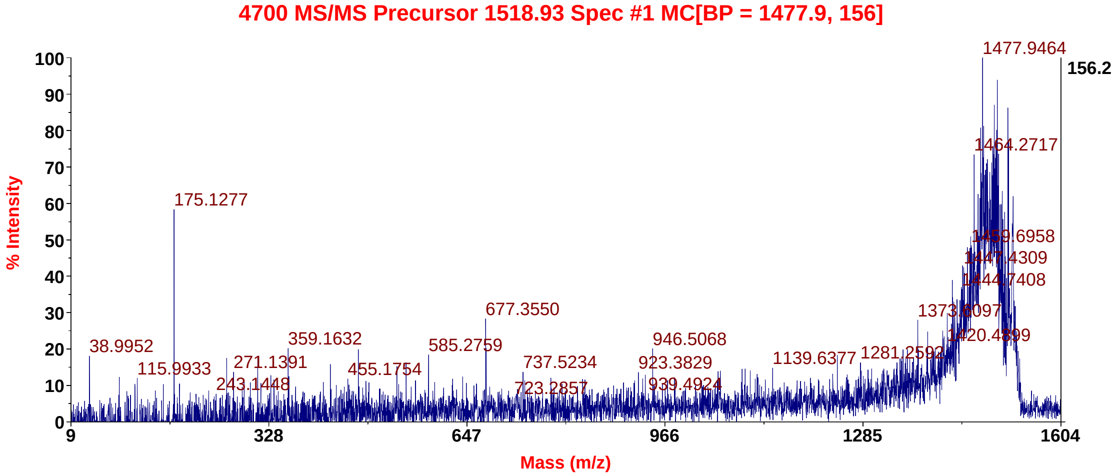


C:\...\D6_MSMS_1518.9275_19.t2d
Acquired:

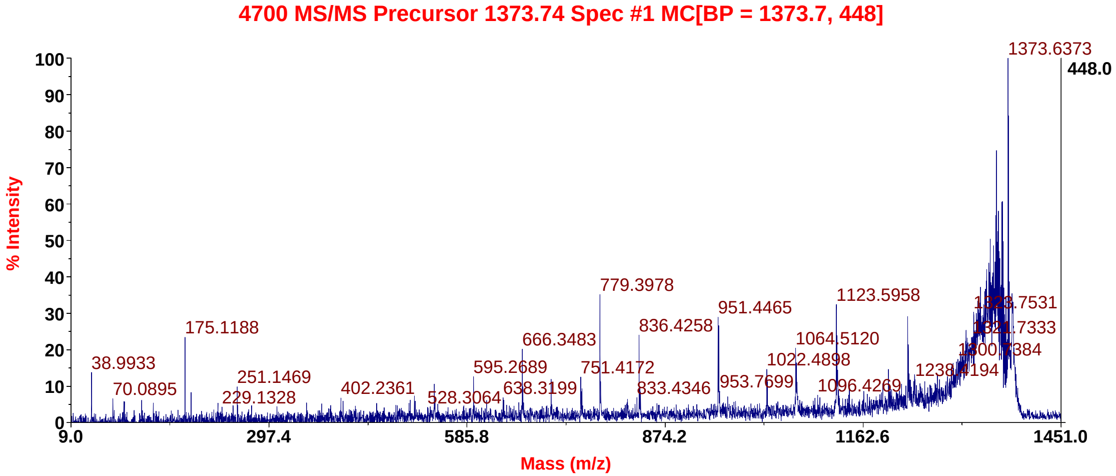


C:\...\D6_MSMS_1373.7434_16.t2d
Acquired:

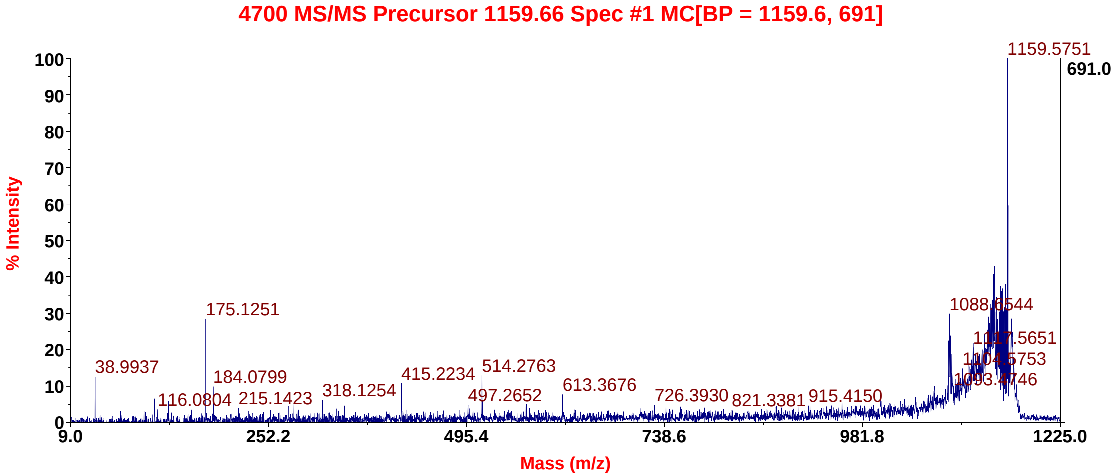


C:\...\D6_MSMS_1159.6624_21.t2d
Acquired:

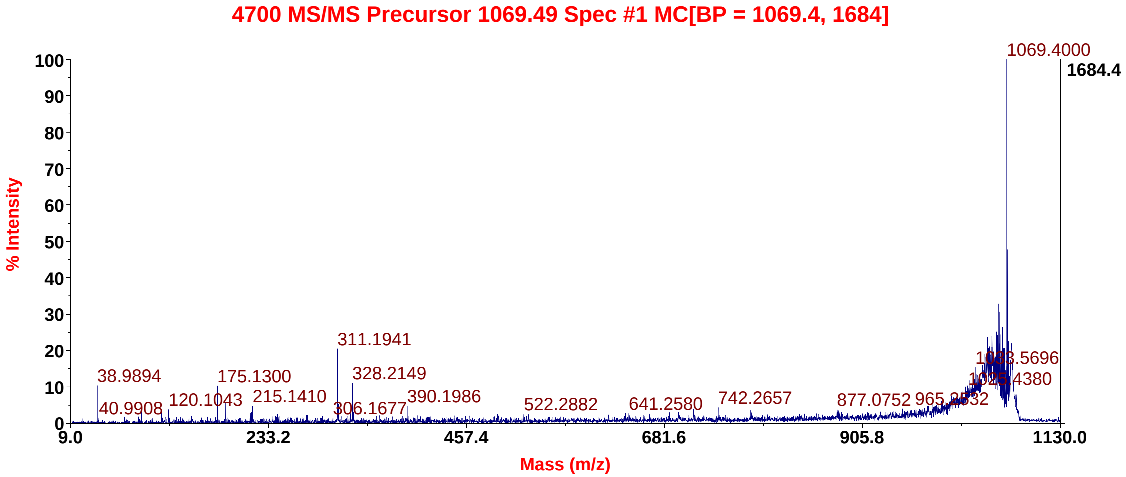


C:\...\D6_MSMS_1069.4851_14.t2d
Acquired:

D6_MSMS_2

4700 MS/MS Precursor 1542.8 Spec #1 MC[BP = 1542.7, 650]

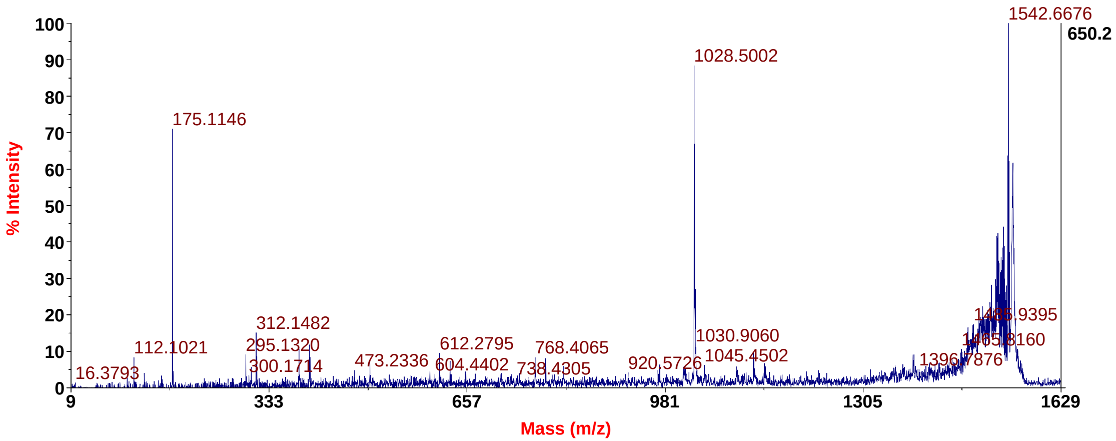


C:\...\D7_MSMS_1542.7958_20.t2d
Acquired:

4700 MS/MS Precursor 1373.73 Spec #1 MC[BP = 1373.7, 1286]

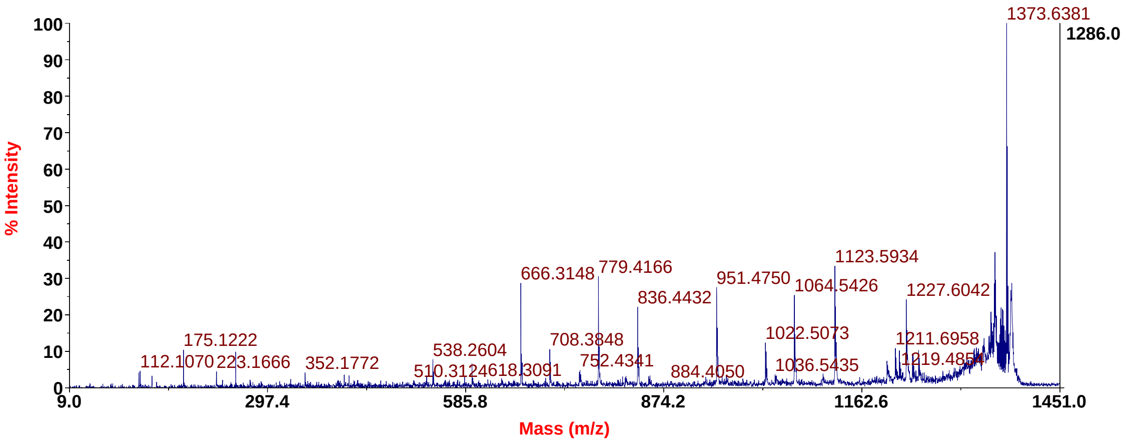


C:\...\D7_MSMS_1373.7271_14.t2d
Acquired:

4700 MS/MS Precursor 1283.63 Spec #1 MC[BP = 720.3, 819]

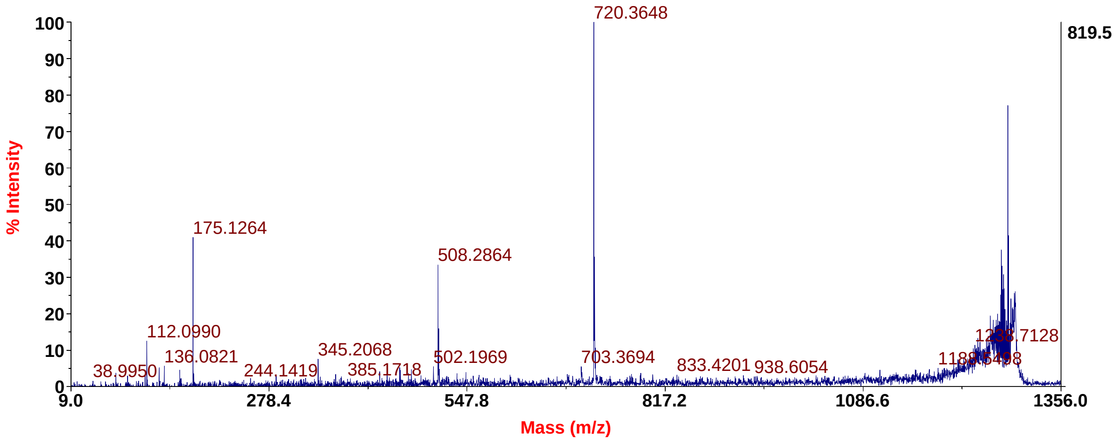


C:\...\D7_MSMS_1283.6295_21.t2d
Acquired:

4700 MS/MS Precursor 1069.45 Spec #1 MC[BP = 1069.4, 2637]

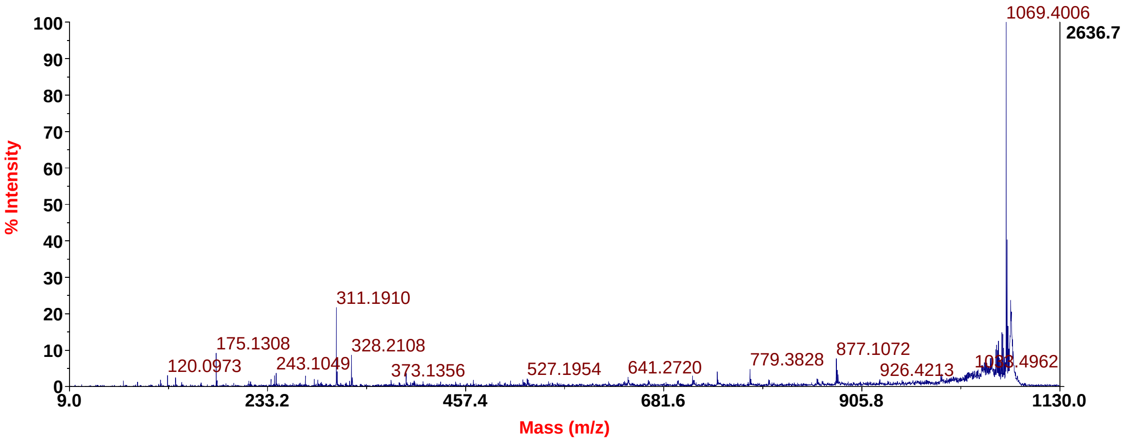


C:\...\D7_MSMS_1069.4509_16.t2d
Acquired:

4700 MS/MS Precursor 824.415 Spec #1 MC[BP = 824.4, 4586]

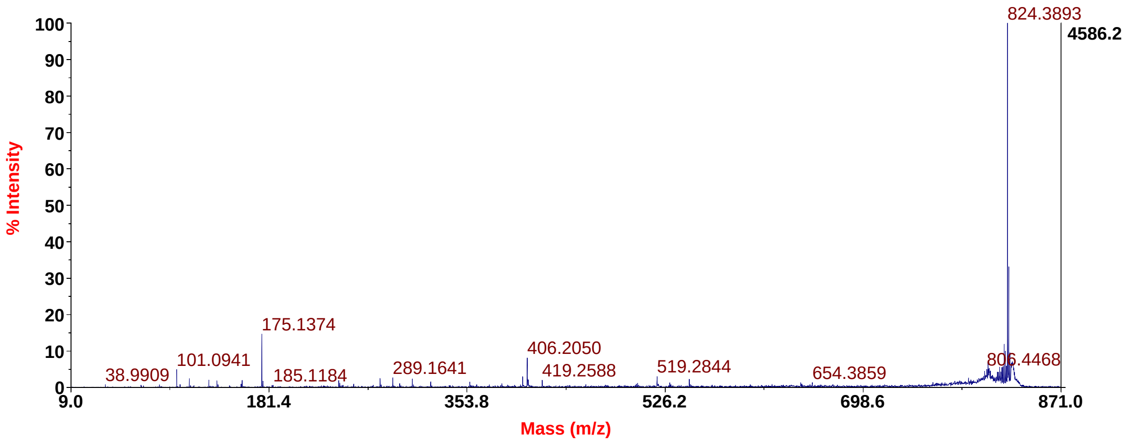


C:\...\D7_MSMS_824.4151_17.t2d
Acquired:

# D7_MSMS_1

**4700 MS/MS Precursor 2068.16 Spec #1 MC[BP = 2027.9, 272]**

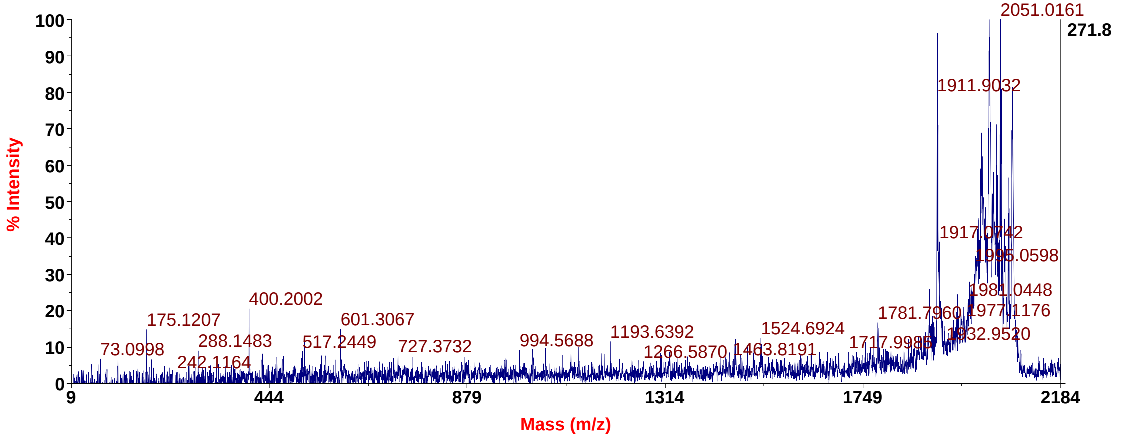


C:\...\D7_MSMS_2068.1602_18.t2d
Acquired:

**4700 MS/MS Precursor 1867.96 Spec #1 MC[BP = 400.2, 2725]**

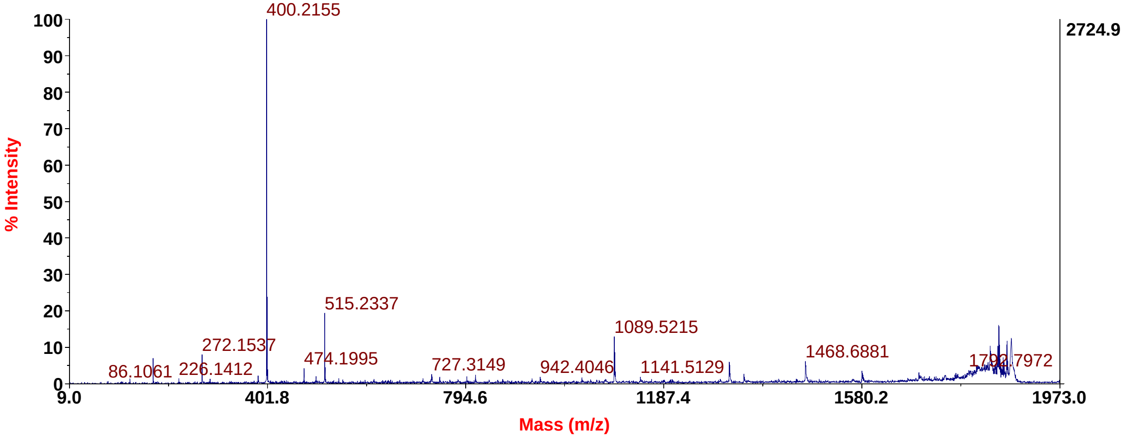


C:\...\D7_MSMS_1867.9620_15.t2d
Acquired:

**4700 MS/MS Precursor 1764.98 Spec #1 MC[BP = 1764.8, 1333]**

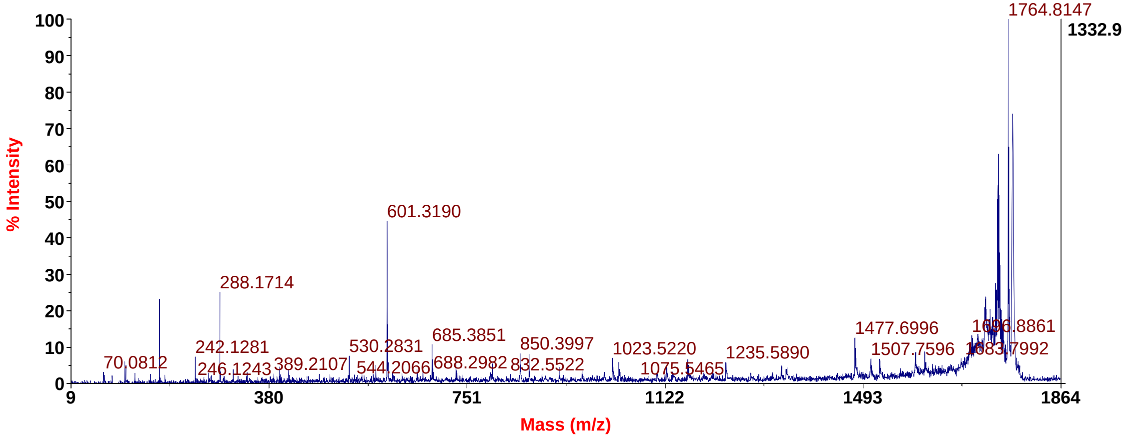


C:\...\D7_MSMS_1764.9777_13.t2d
Acquired:

**4700 MS/MS Precursor 1744.85 Spec #1 MC[BP = 1723.8, 272]**

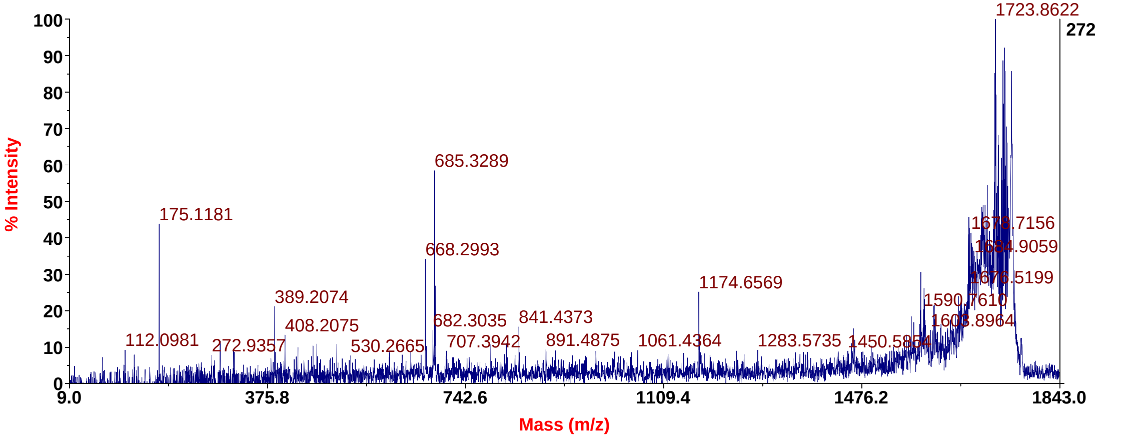


C:\...\D7_MSMS_1744.8477_22.t2d
Acquired:

**4700 MS/MS Precursor 1633.86 Spec #1 MC[BP = 1633.8, 243]**

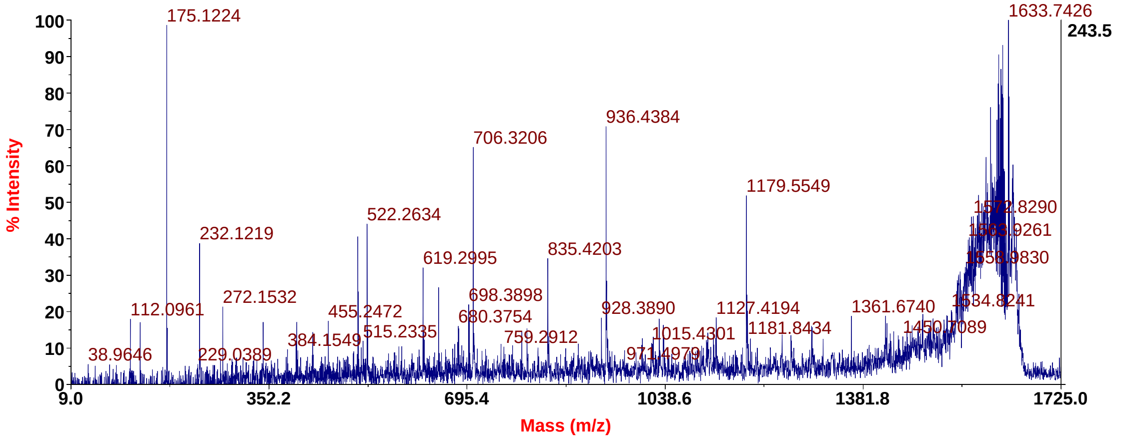


C:\...\D7_MSMS_1633.8584_19.t2d
Acquired:

D7_MSMS_2

4700 MS/MS Precursor 1744.8 Spec #1 MC[BP = 685.3, 13925]

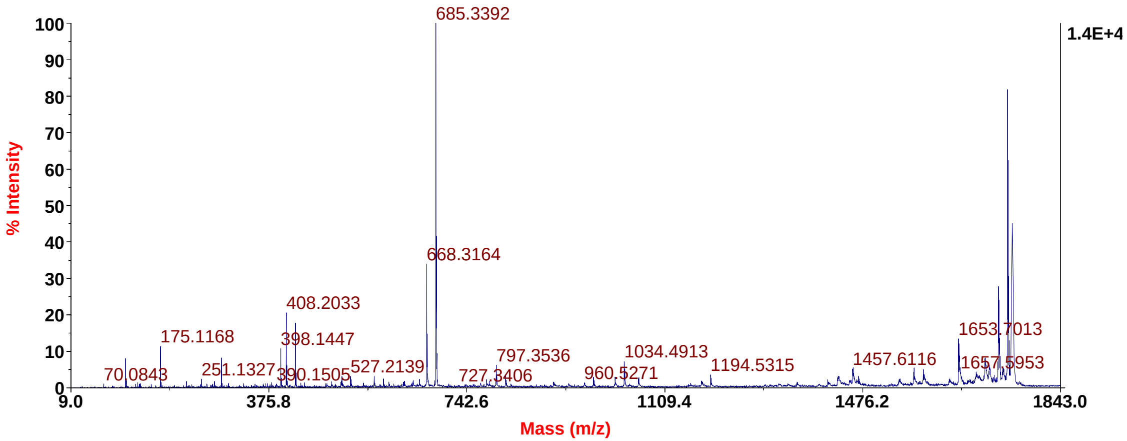


C:\...\D8_MSMS_1744.7999_13.t2d
Acquired:

4700 MS/MS Precursor 1159.66 Spec #1 MC[BP = 1159.6, 19948]

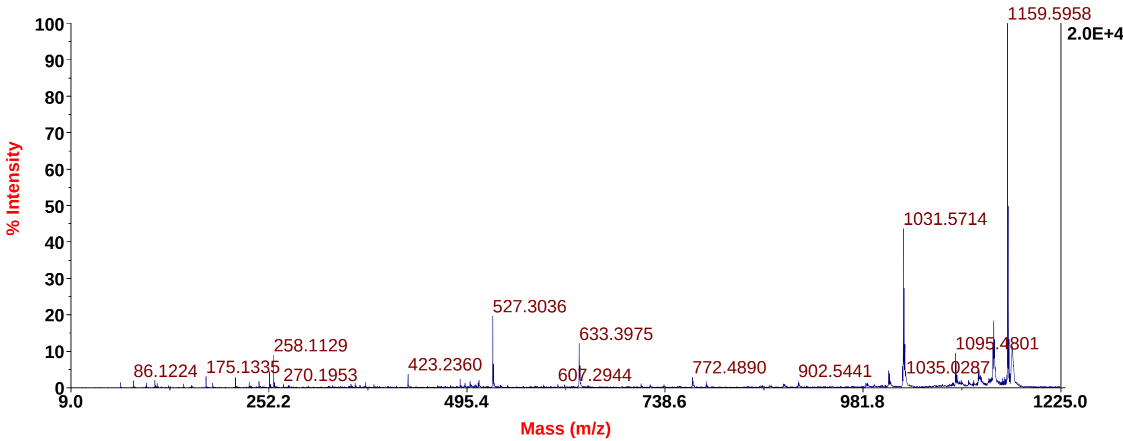


C:\...\D8_MSMS_1159.6556_15.t2d
Acquired:

4700 MS/MS Precursor 1123.52 Spec #1 MC[BP = 1123.4, 17079]

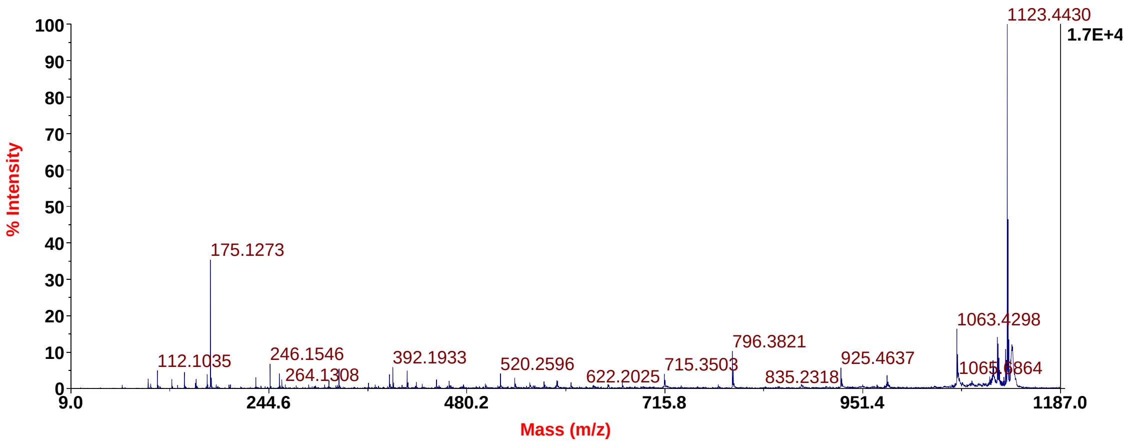


C:\...\D8_MSMS_1123.5184_17.t2d
Acquired:

4700 MS/MS Precursor 1111.52 Spec #1 MC[BP = 1111.4, 13037]

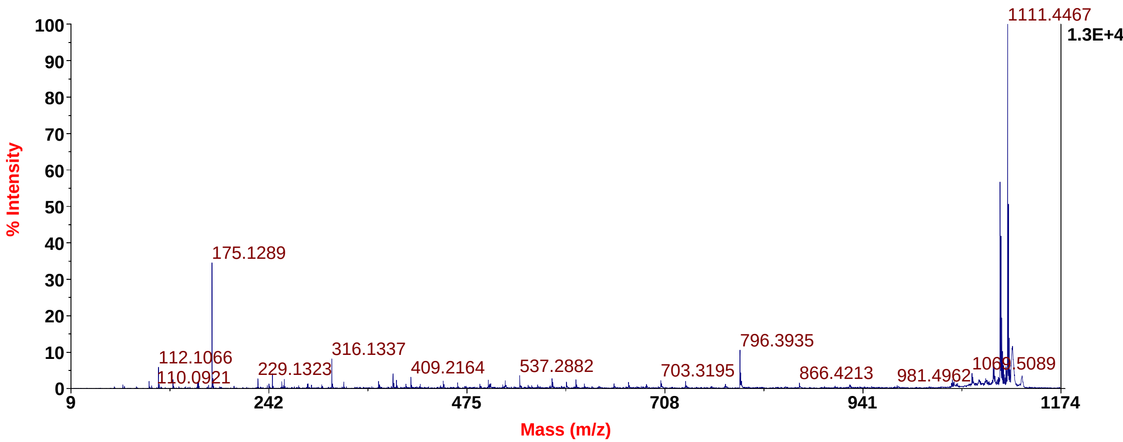


C:\...\D8_MSMS_1111.5206_20.t2d
Acquired:

4700 MS/MS Precursor 1102.59 Spec #1 MC[BP = 1102.5, 10374]

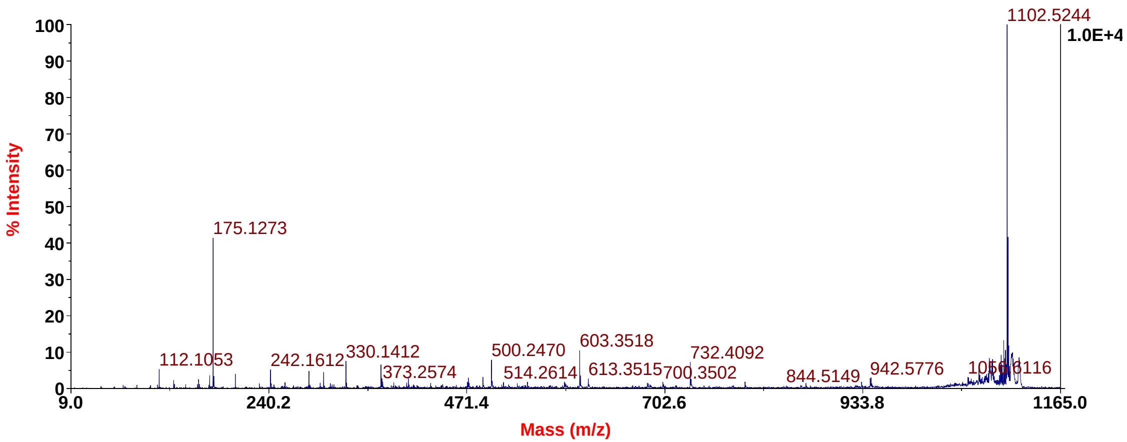


C:\...\D8_MSMS_1102.5925_21.t2d
Acquired:

D8_MSMS_1

**4700 MS/MS Precursor 2951.53 Spec #1 MC[BP = 1313.4, 2394]**

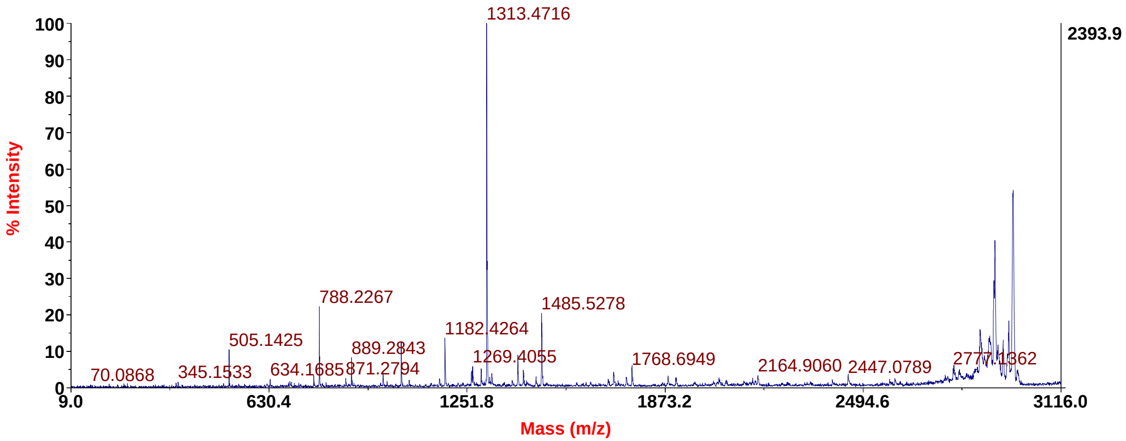


C:\...\D8_MSMS_2951.5327_22.t2d
Acquired:

**4700 MS/MS Precursor 2394.18 Spec #1 MC[BP = 659.3, 6714]**

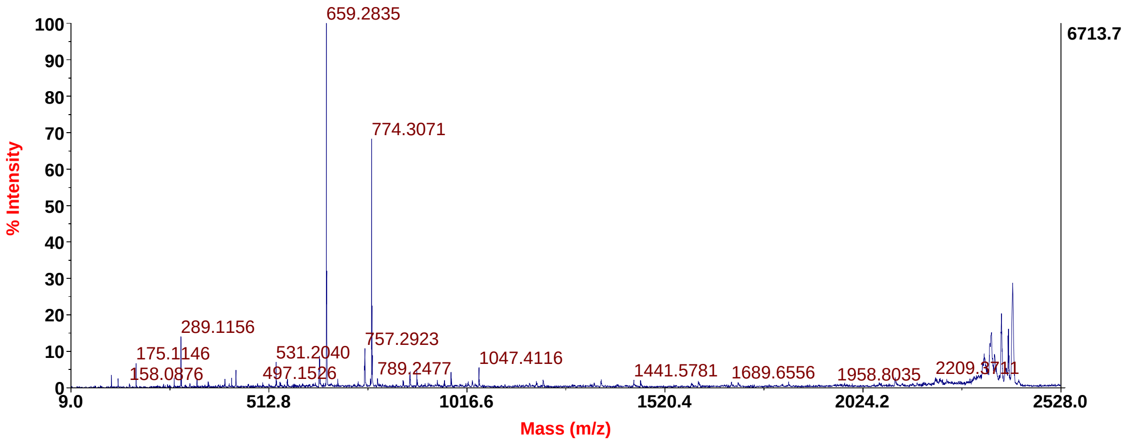


C:\...\D8_MSMS_2394.1802_18.t2d
Acquired:

**4700 MS/MS Precursor 2207.13 Spec #1 MC[BP = 359.2, 4071]**

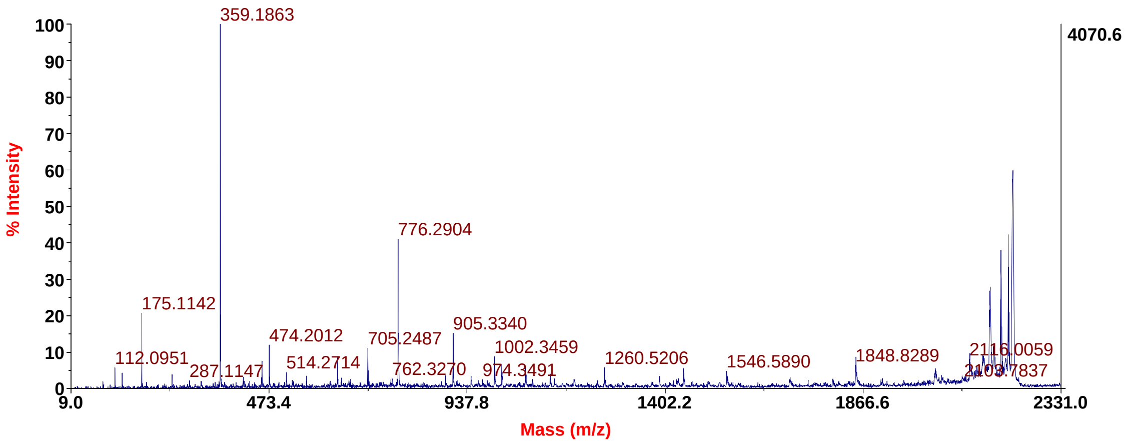


C:\...\D8_MSMS_2207.1326_19.t2d
Acquired:

**4700 MS/MS Precursor 1907.96 Spec #1 MC[BP = 359.2, 8616]**

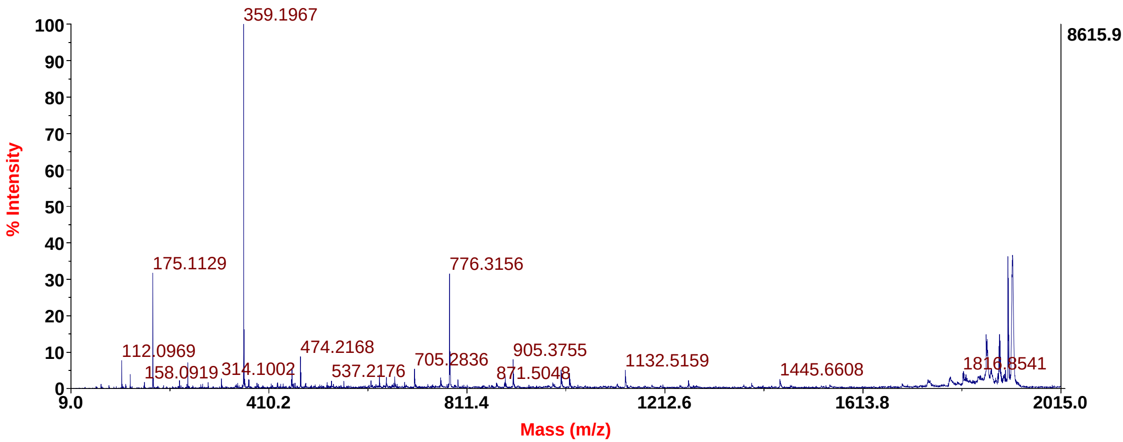


C:\...\D8_MSMS_1907.9579_16.t2d
Acquired:

**4700 MS/MS Precursor 1788.89 Spec #1 MC[BP = 1788.8, 5181]**

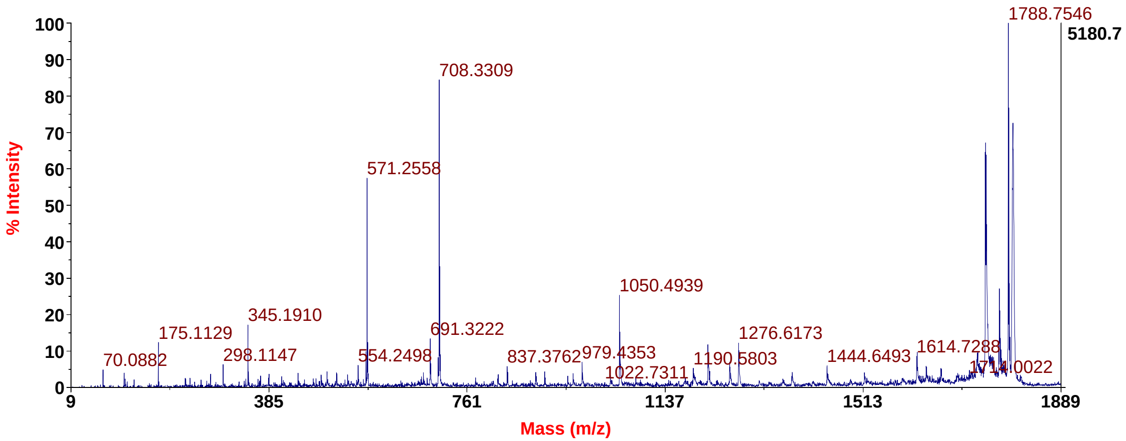


C:\...\D8_MSMS_1788.8934_14.t2d
Acquired:

# D8_MSMS_2

4700 MS/MS Precursor 1069.43 Spec #1 MC[BP = 1069.4, 5068]

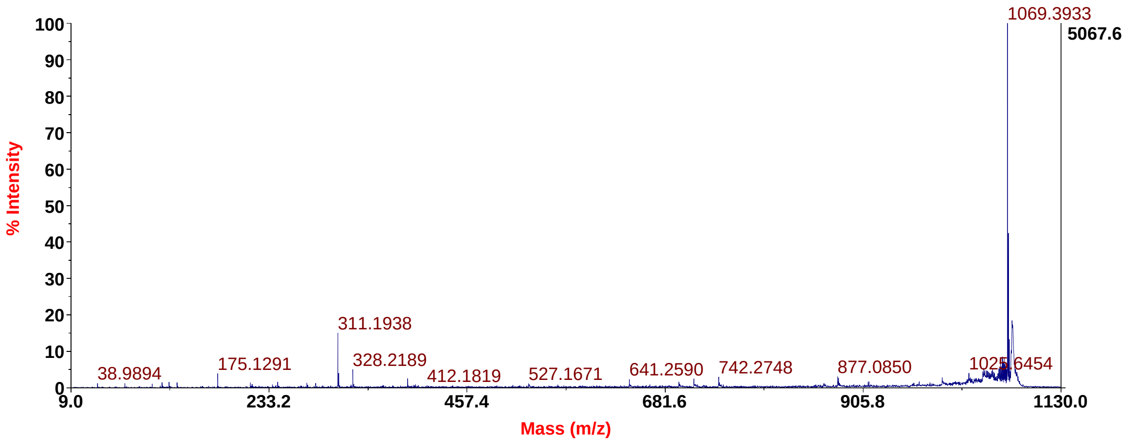


C:\...\D9_MSMS_1069.4333_13.t2d
Acquired:

4700 MS/MS Precursor 1025.6 Spec #1 MC[BP = 1025.6, 2229]

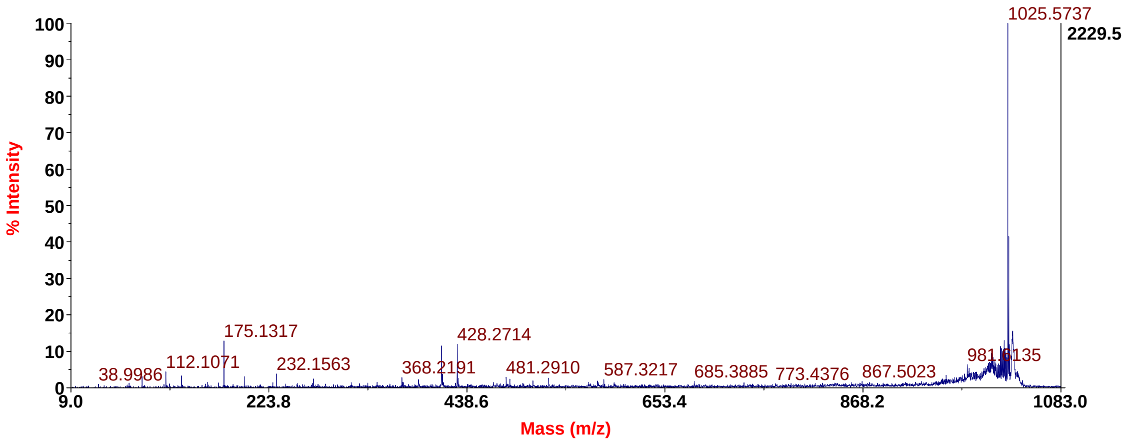


C:\...\D9_MSMS_1025.5963_17.t2d
Acquired:

4700 MS/MS Precursor 972.56 Spec #1 MC[BP = 972.6, 1926]

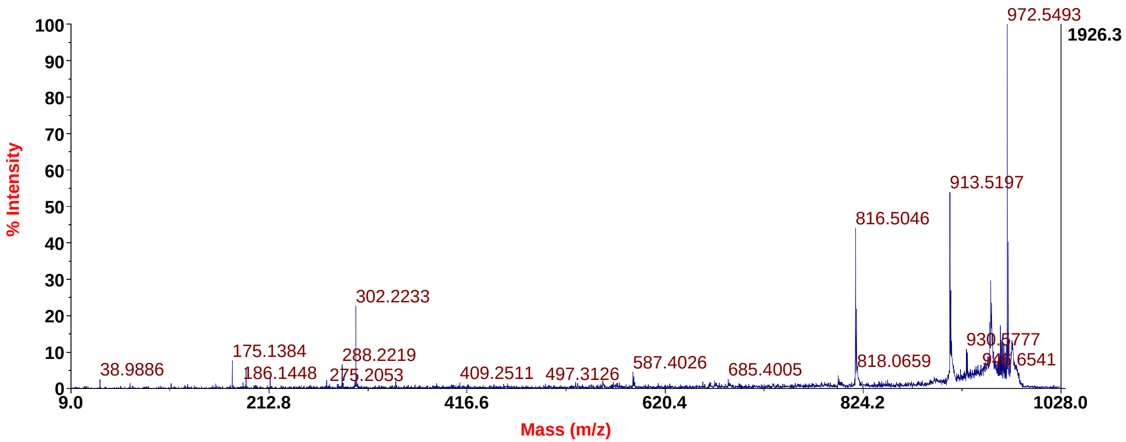


C:\...\D9_MSMS_972.5602_18.t2d
Acquired:

4700 MS/MS Precursor 892.464 Spec #1 MC[BP = 892.5, 5444]

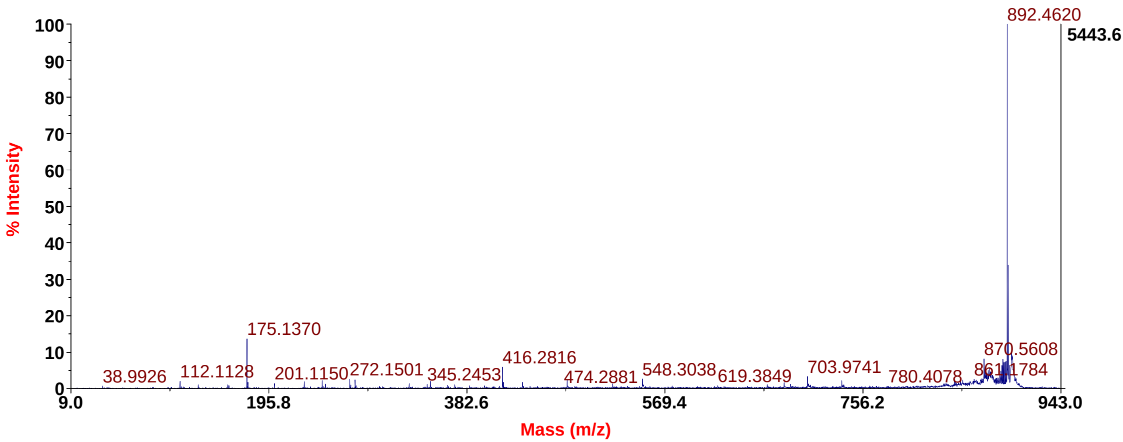


C:\...\D9_MSMS_892.4644_15.t2d
Acquired:

4700 MS/MS Precursor 870.515 Spec #1 MC[BP = 870.5, 9557]

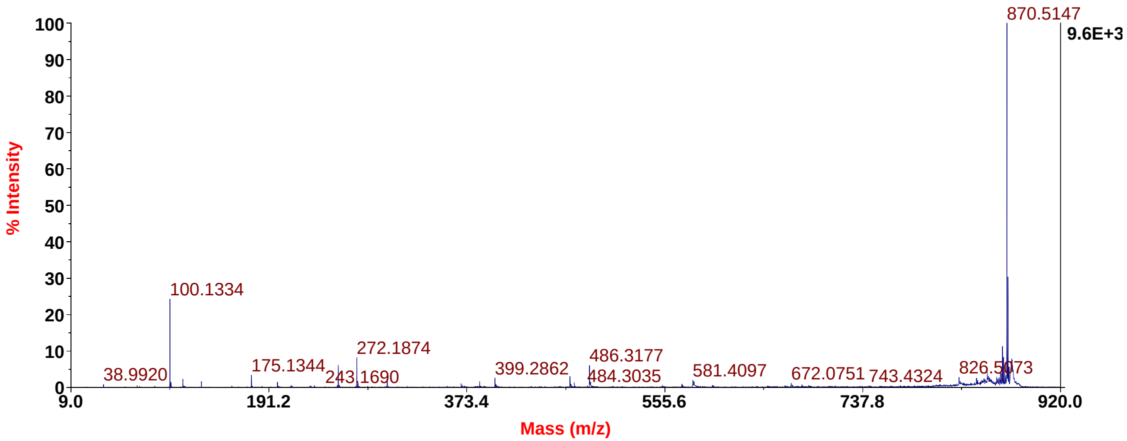


C:\...\D9_MSMS_870.5150_12.t2d
Acquired:

# D9_MSMS_1

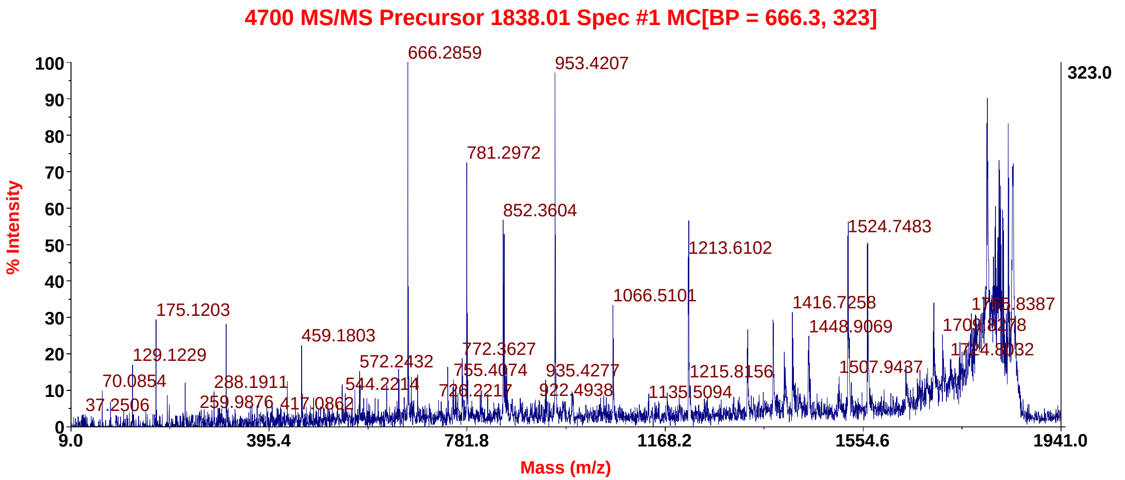


C:\...\D9_MSMS_1838.0127_14.t2d
Acquired:

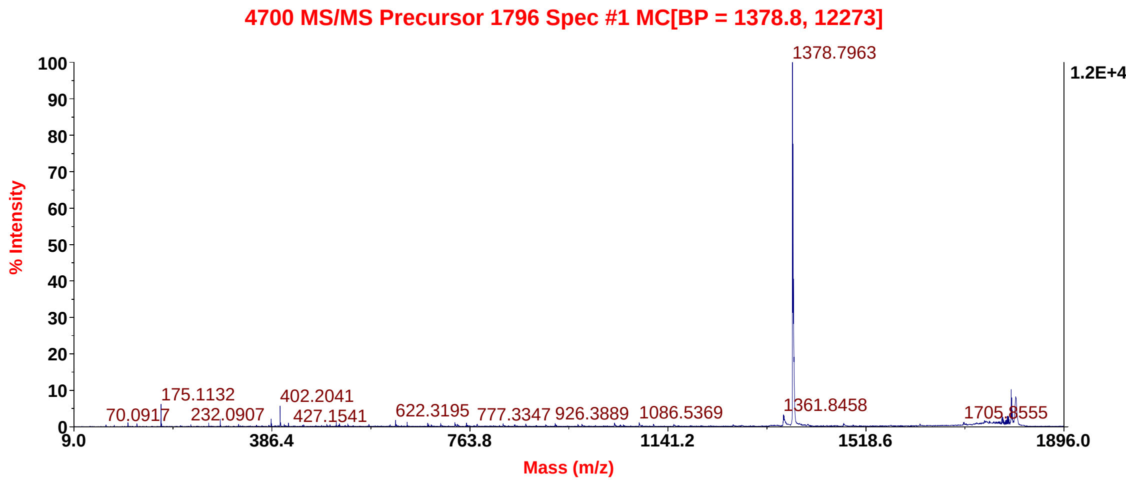


C:\...\D9_MSMS_1796.0029_10.t2d
Acquired:

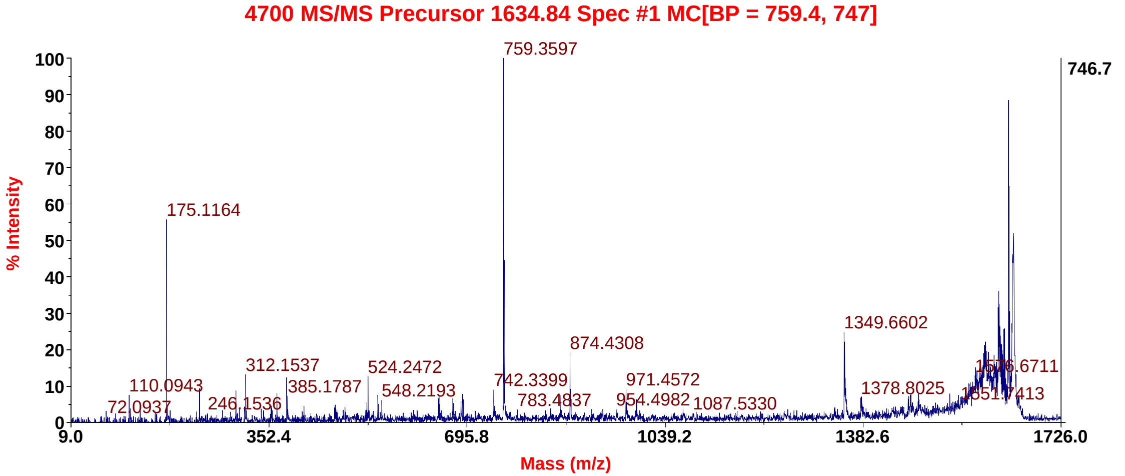


C:\...\D9_MSMS_1634.8364_16.t2d
Acquired:

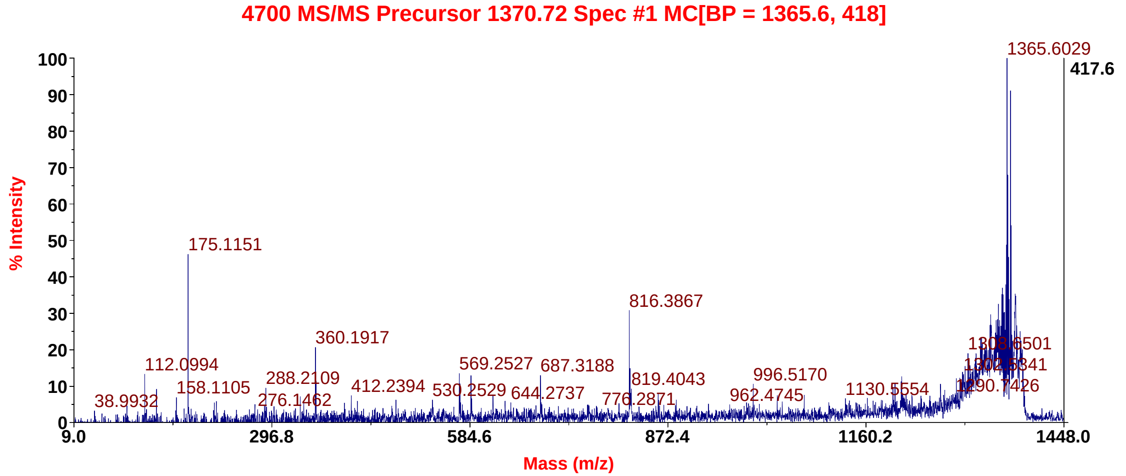


C:\...\D9_MSMS_1370.7231_19.t2d
Acquired:

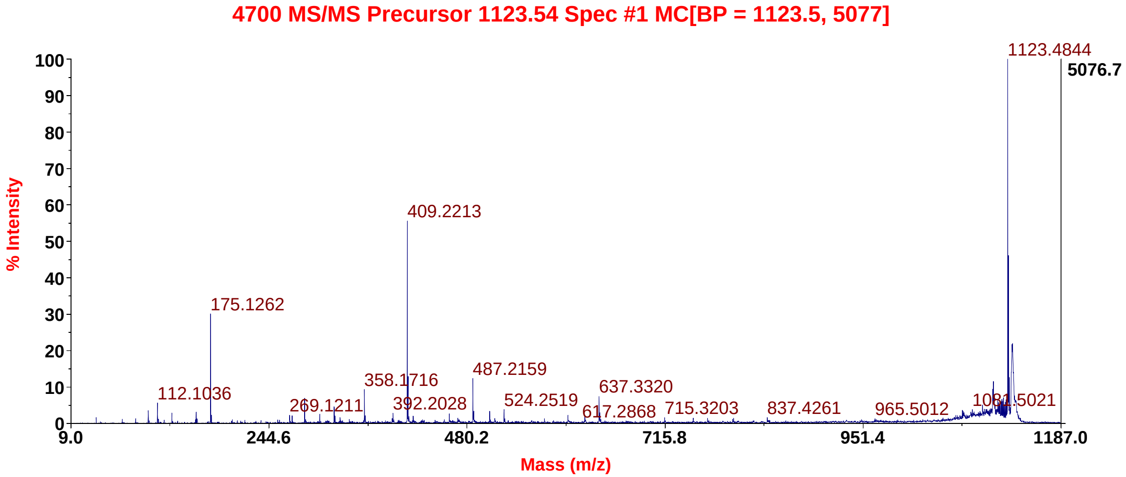


C:\...\D9_MSMS_1123.5361_11.t2d
Acquired:

D9_MSMS_2

4700 MS/MS Precursor 1256.63 Spec #1 MC[BP = 1256.6, 3829]

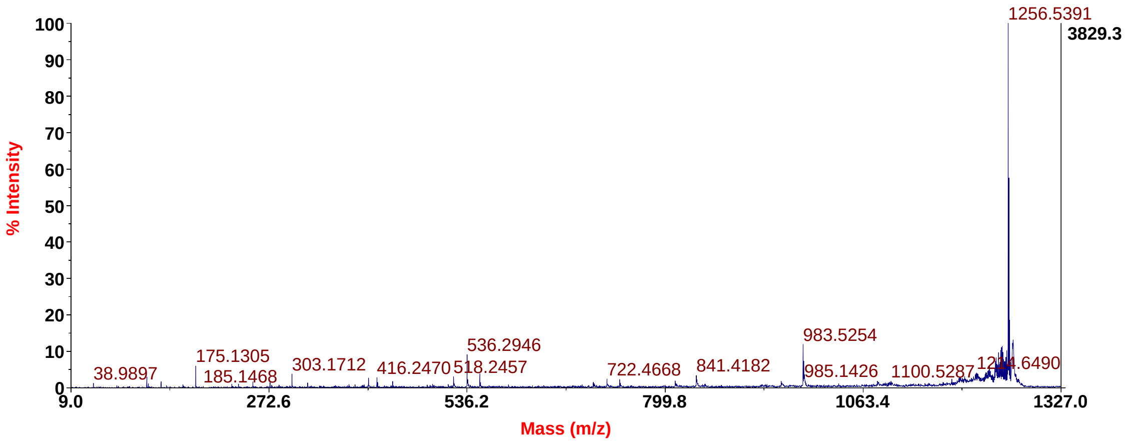


C:\...\E3_MSMS_1256.6276_15.t2d
Acquired:

4700 MS/MS Precursor 1189.62 Spec #1 MC[BP = 1189.5, 1704]

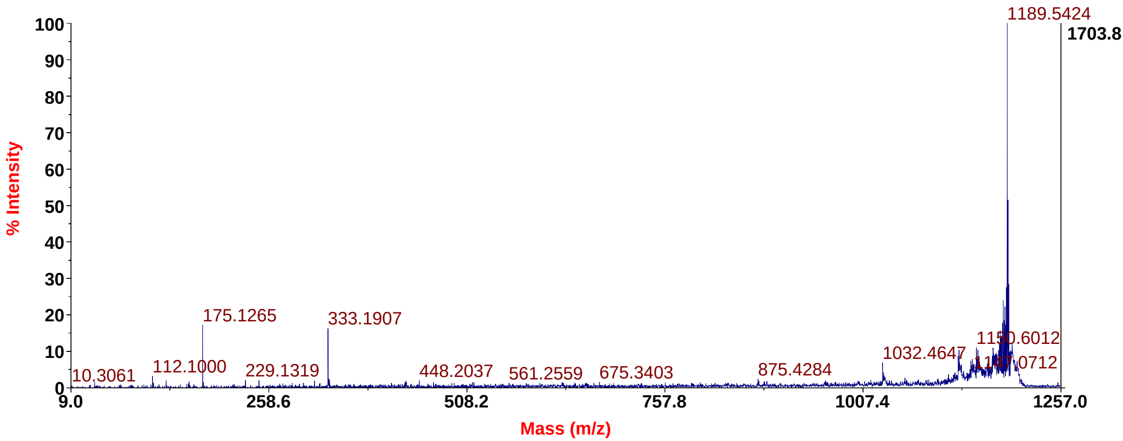


C:\...\E3_MSMS_1189.6152_22.t2d
Acquired:

4700 MS/MS Precursor 1150.62 Spec #1 MC[BP = 1150.5, 8897]

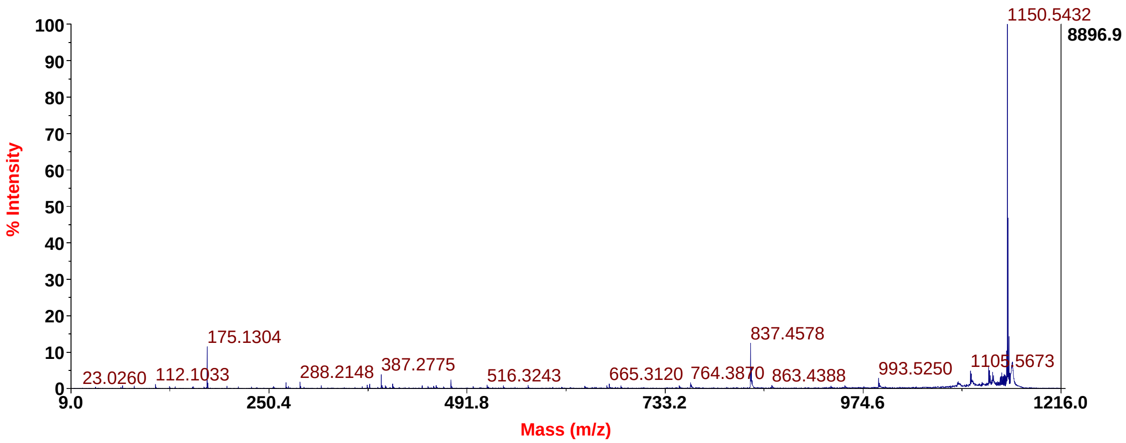


C:\...\E3_MSMS_1150.6163_14.t2d
Acquired:

4700 MS/MS Precursor 1127.63 Spec #1 MC[BP = 1127.5, 12547]

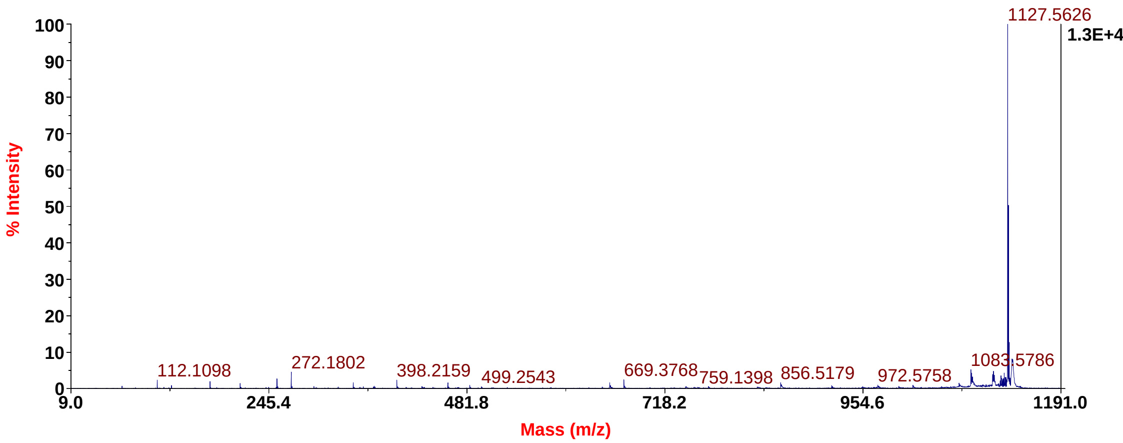


C:\...\E3_MSMS_1127.6288_13.t2d
Acquired:

4700 MS/MS Precursor 1069.45 Spec #1 MC[BP = 1069.4, 3995]

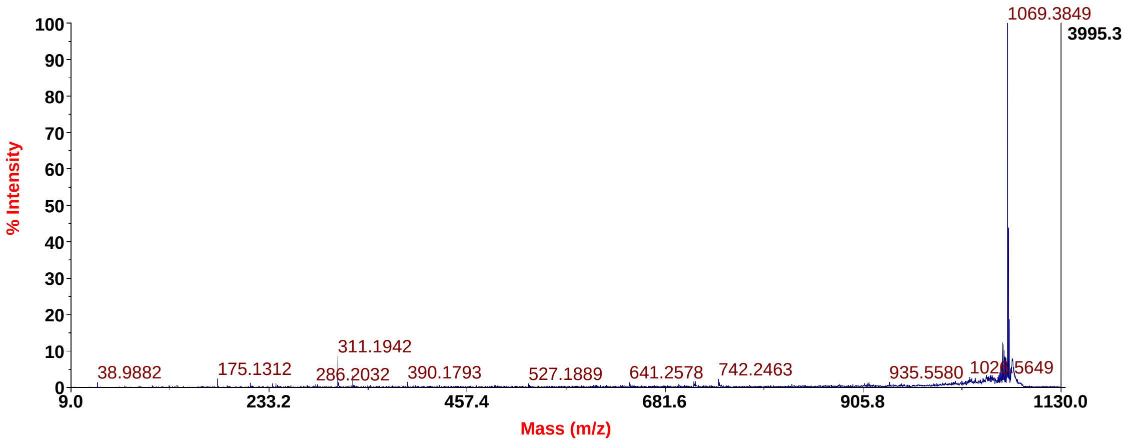


C:\...\E3_MSMS_1069.4465_18.t2d
Acquired:

E3_MSMS_1

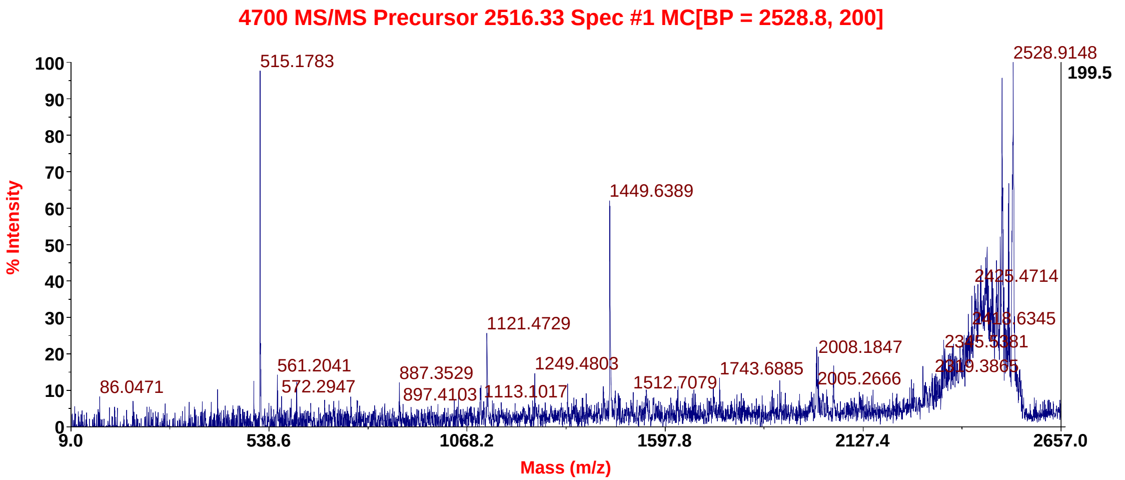


C:\...\E3_MSMS_2516.3337_21.t2d
Acquired:

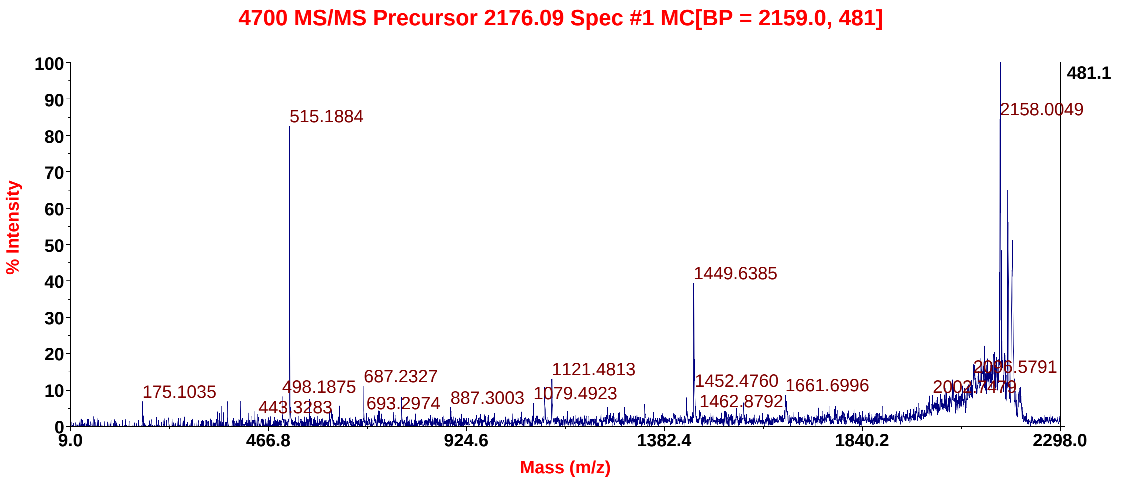


C:\...\E3_MSMS_2176.0933_19.t2d
Acquired:

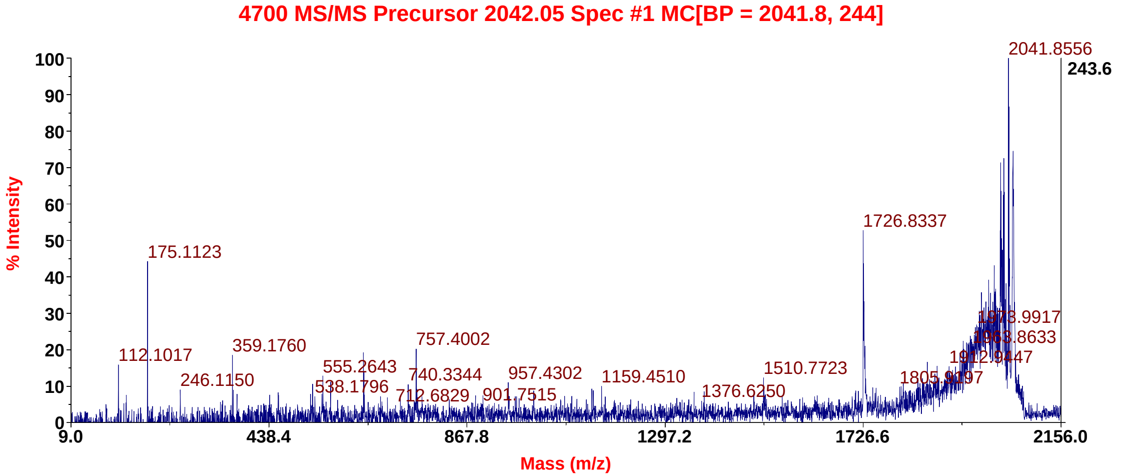


C:\...\E3_MSMS_2042.0453_20.t2d
Acquired:

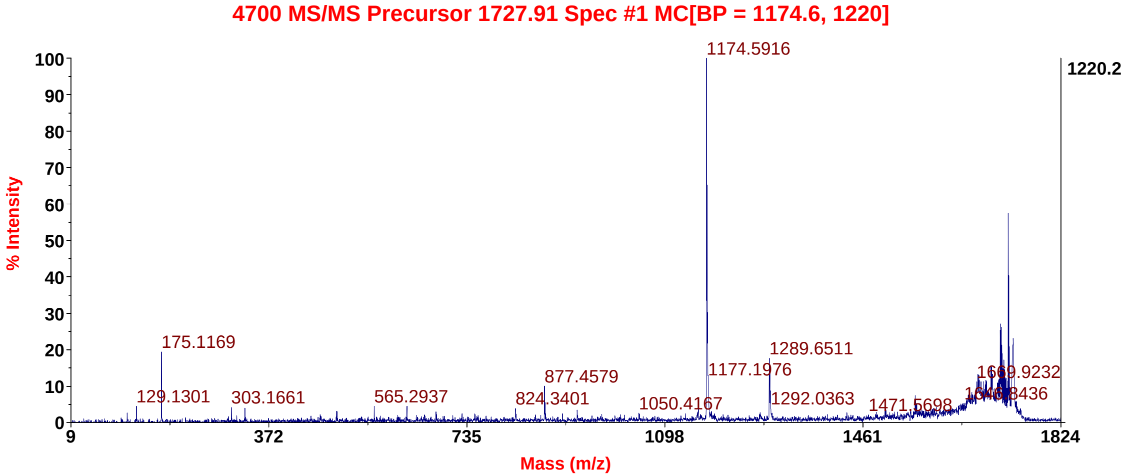


C:\...\E3_MSMS_1727.9110_17.t2d
Acquired:

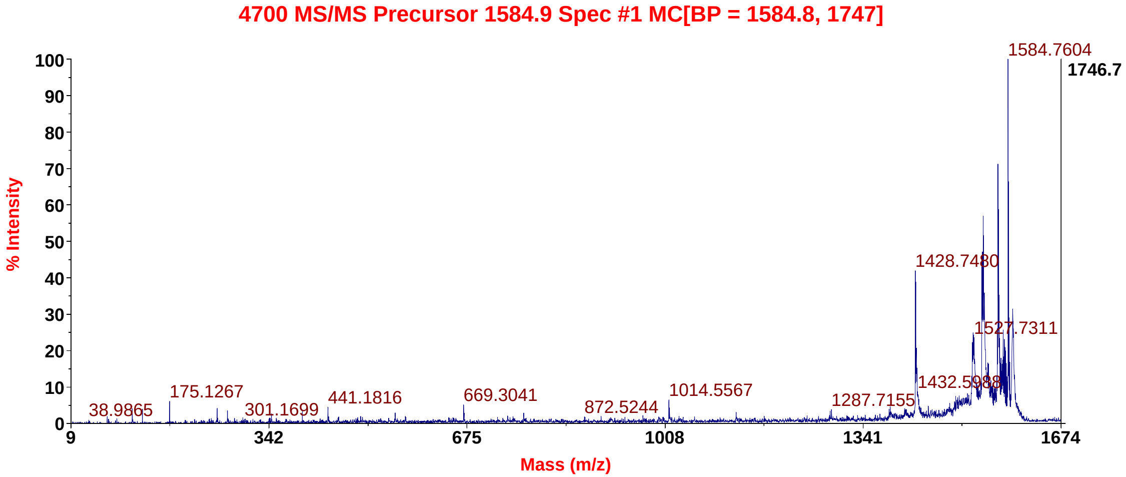


C:\...\E3_MSMS_1584.8954_16.t2d
Acquired:

E3_MSMS_2

**4700 MS/MS Precursor 1212.62 Spec #1 MC[BP = 1212.5, 5703]**

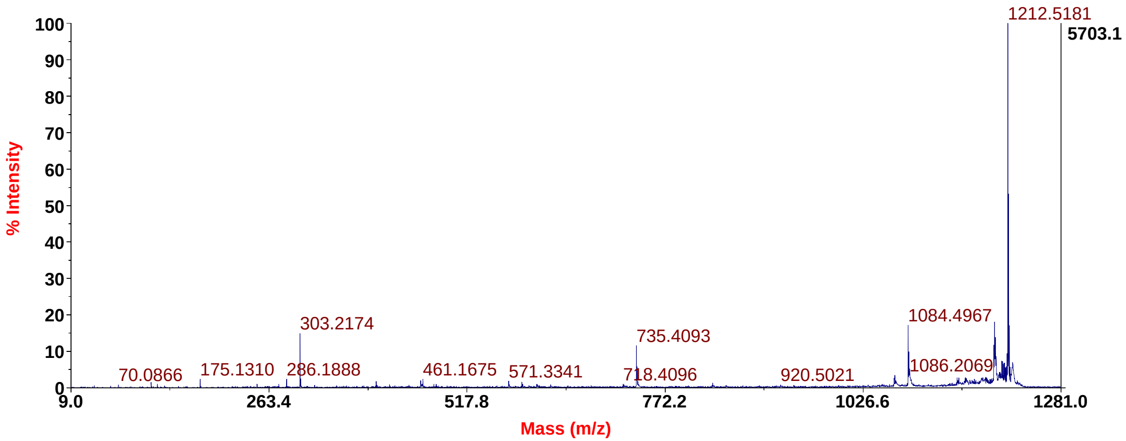


C:\...\E11_MSMS_1212.6158_15.t2d
Acquired:

**4700 MS/MS Precursor 1156.57 Spec #1 MC[BP = 1157.5, 8980]**

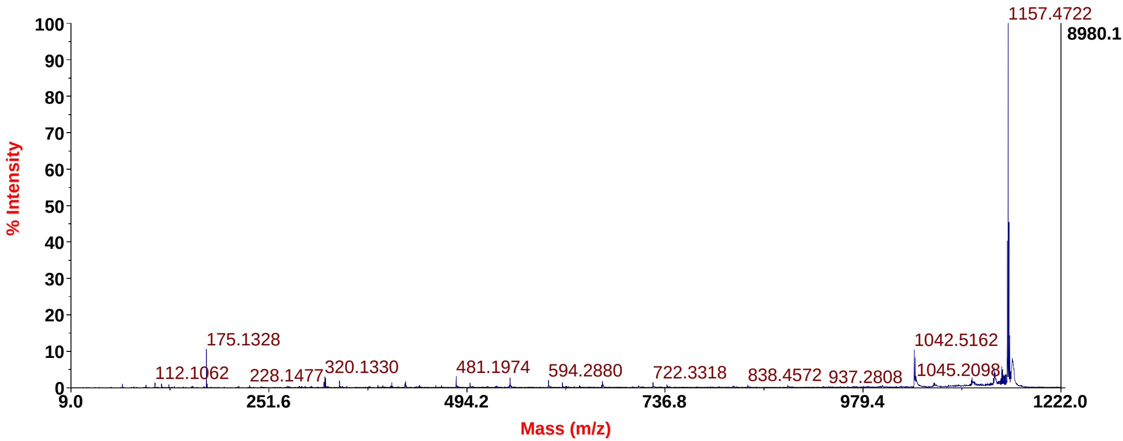


C:\...\E11_MSMS_1156.5725_19.t2d
Acquired:

**4700 MS/MS Precursor 1069.45 Spec #1 MC[BP = 1069.4, 5773]**

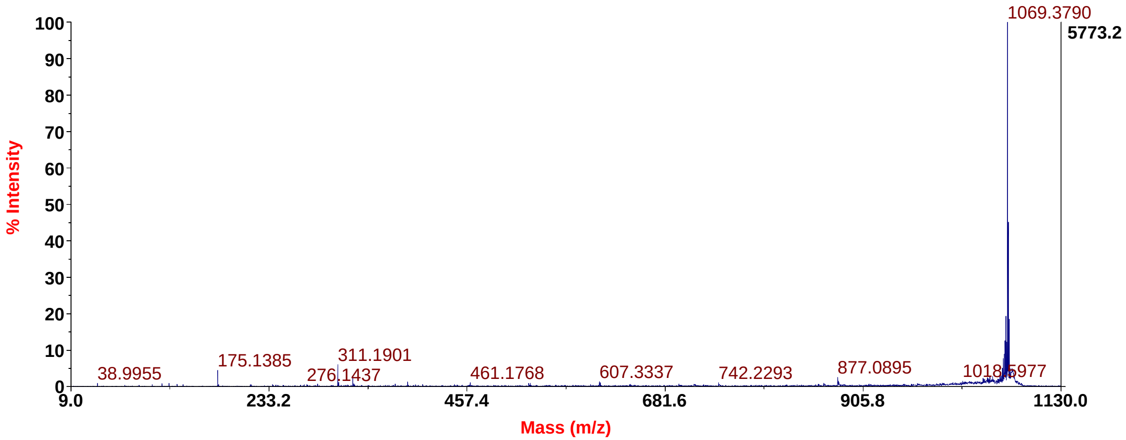


C:\...\E11_MSMS_1069.4521_18.t2d
Acquired:

**4700 MS/MS Precursor 1017.55 Spec #1 MC[BP = 1017.5, 4351]**

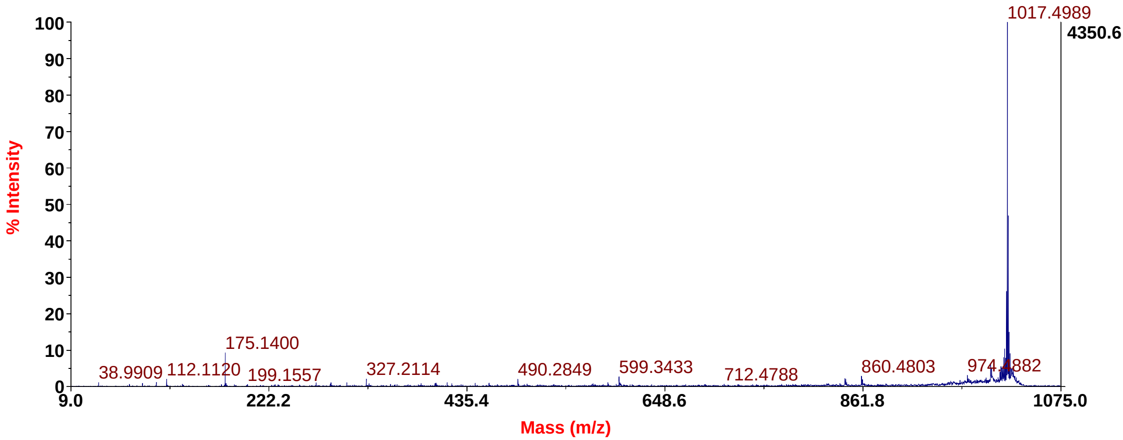


C:\...\E11_MSMS_1017.5524_22.t2d
Acquired:

**4700 MS/MS Precursor 936.488 Spec #1 MC[BP = 936.4, 20676]**

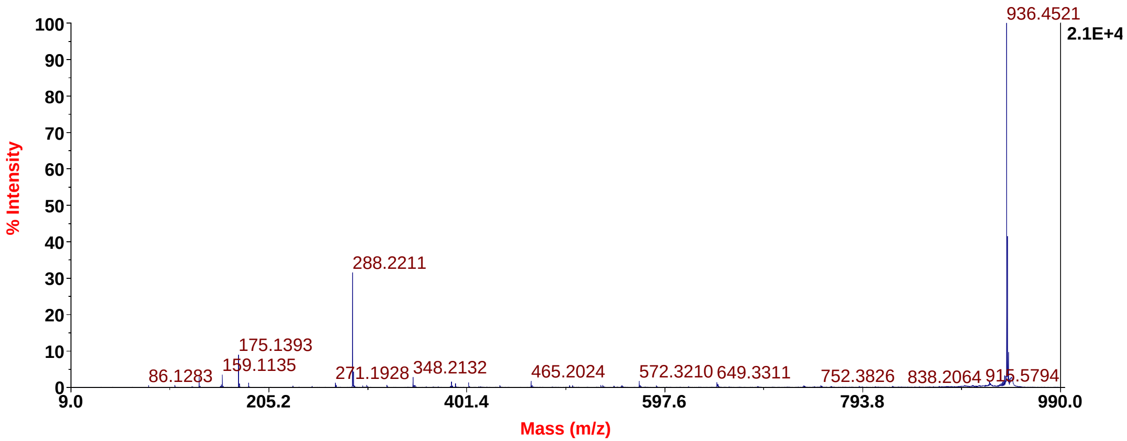


C:\...\E11_MSMS_936.4884_13.t2d
Acquired:

E11_MSMS_1

4700 MS/MS Precursor 1650.92 Spec #1 MC[BP = 1650.8, 955]

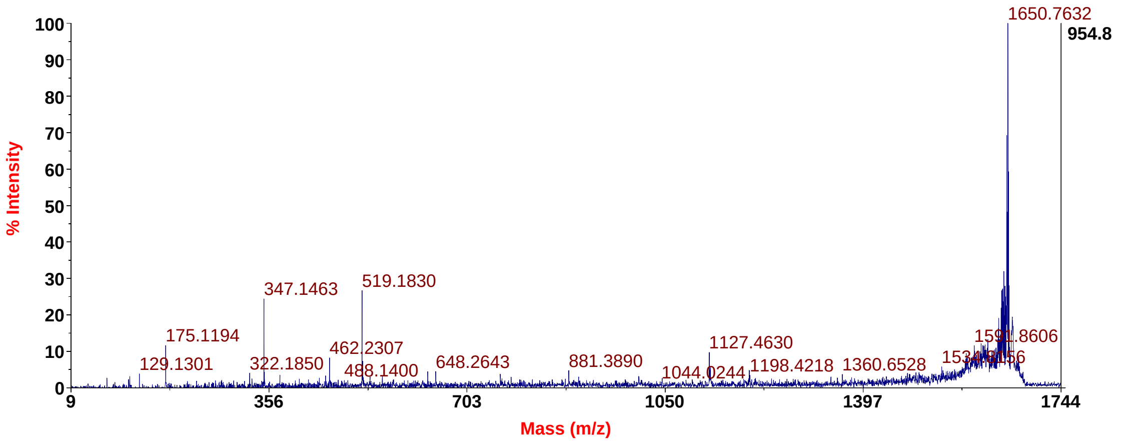


C:\...\E11_MSMS_1650.9176_21.t2d
Acquired:

4700 MS/MS Precursor 1516.78 Spec #1 MC[BP = 1516.6, 653]

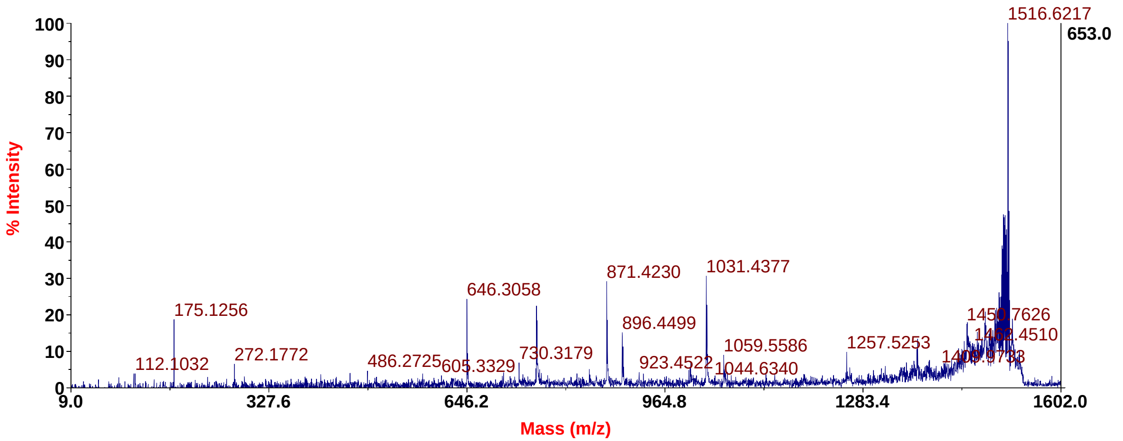


C:\...\E11_MSMS_1516.7791_20.t2d
Acquired:

4700 MS/MS Precursor 1479.66 Spec #1 MC[BP = 1479.5, 2360]

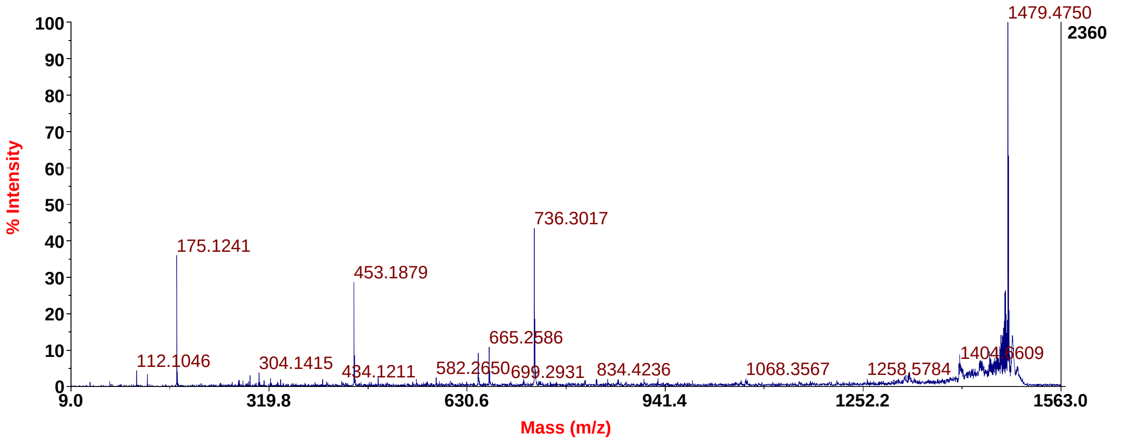


C:\...\E11_MSMS_1479.6553_17.t2d
Acquired:

4700 MS/MS Precursor 1450.74 Spec #1 MC[BP = 1450.6, 3983]

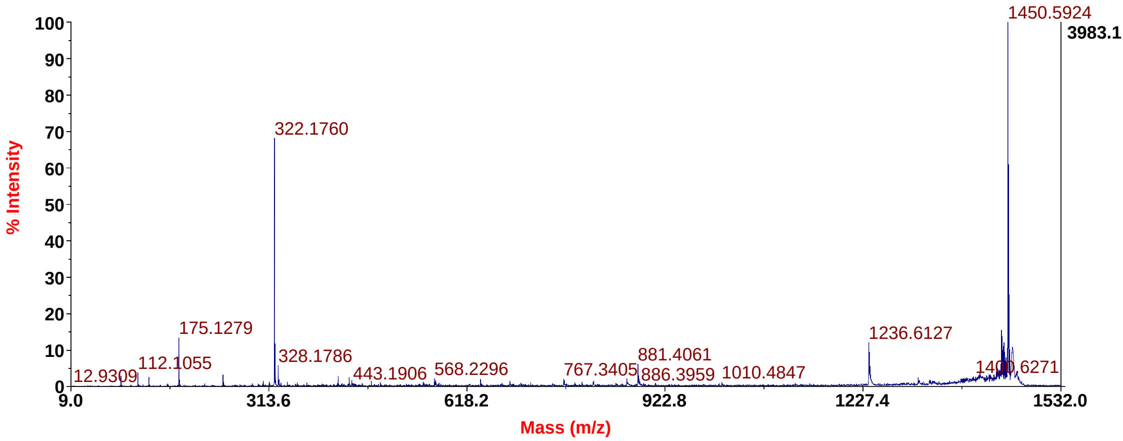


C:\...\E11_MSMS_1450.7412_14.t2d
Acquired:

4700 MS/MS Precursor 1396.66 Spec #1 MC[BP = 1396.5, 1274]

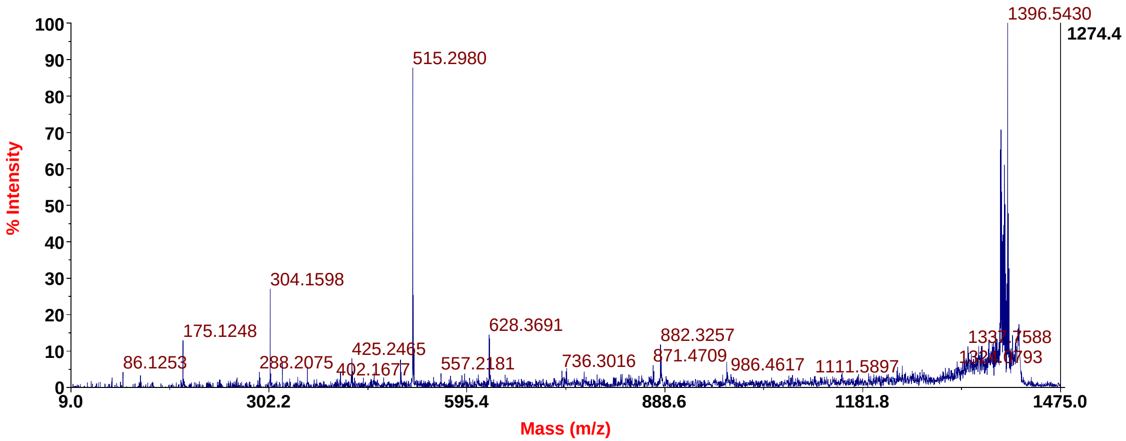


C:\...\E11_MSMS_1396.6628_16.t2d
Acquired:

# E11_MSMS_2

4700 MS/MS Precursor 1725.81 Spec #1 MC[BP = 1725.7, 3804]

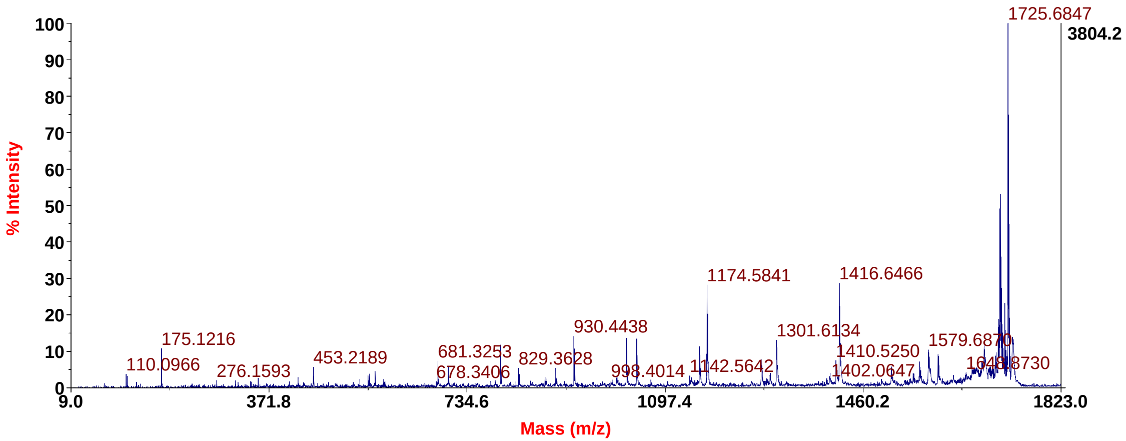


C:\...\E15_MSMS_1725.8132_16.t2d
Acquired:

4700 MS/MS Precursor 1300.65 Spec #1 MC[BP = 1300.6, 28900]

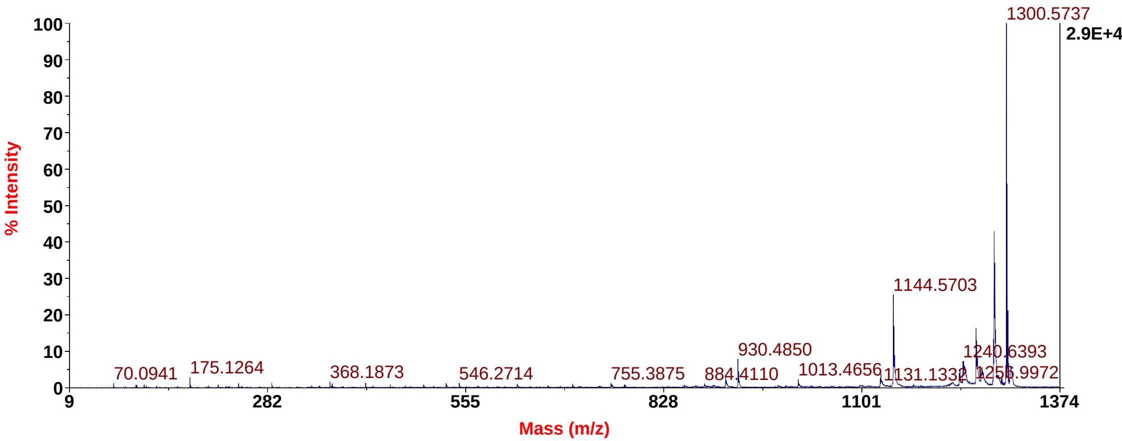


C:\...\E15_MSMS_1300.6538_15.t2d
Acquired:

4700 MS/MS Precursor 1283.63 Spec #1 MC[BP = 1283.6, 10006]

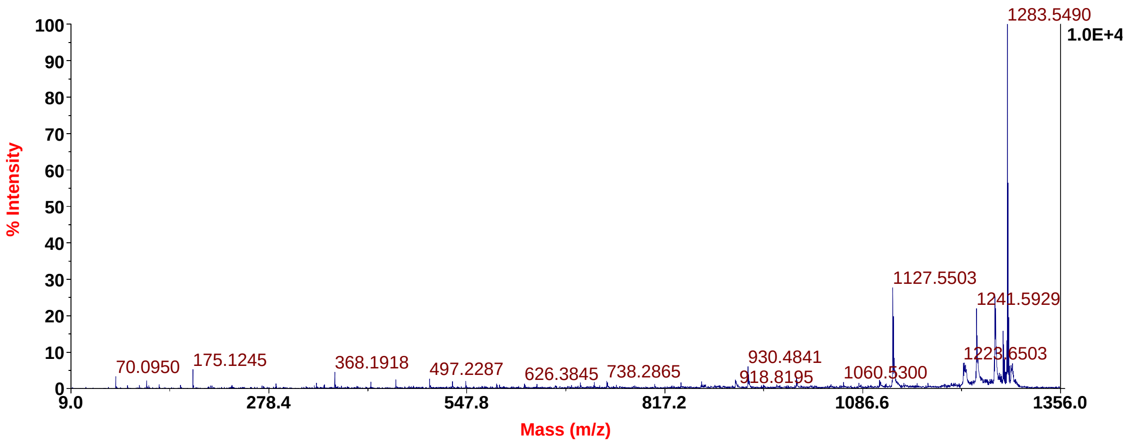


C:\...\E15_MSMS_1283.6262_22.t2d
Acquired:

4700 MS/MS Precursor 1073.52 Spec #1 MC[BP = 1073.5, 9601]

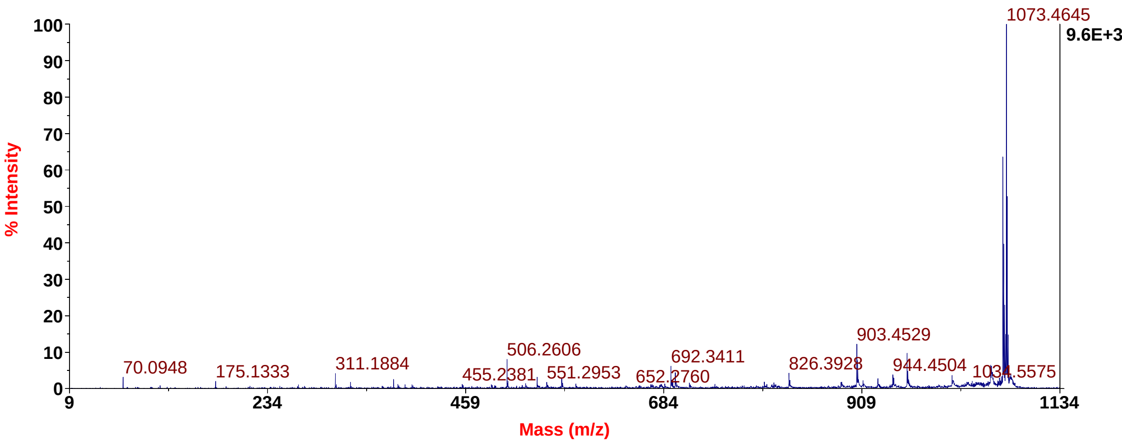


C:\...\E15_MSMS_1073.5228_17.t2d
Acquired:

4700 MS/MS Precursor 1011.61 Spec #1 MC[BP = 1011.6, 60595]

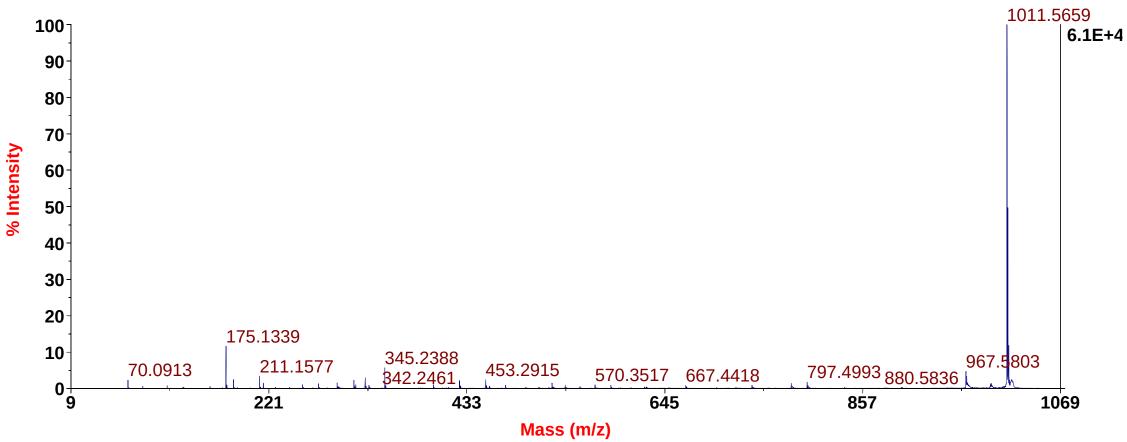


C:\...\E15_MSMS_1011.6050_14.t2d
Acquired:

E15_MSMS_1

**4700 MS/MS Precursor 2380.14 Spec #1 MC[BP = 2380.9, 2360]**

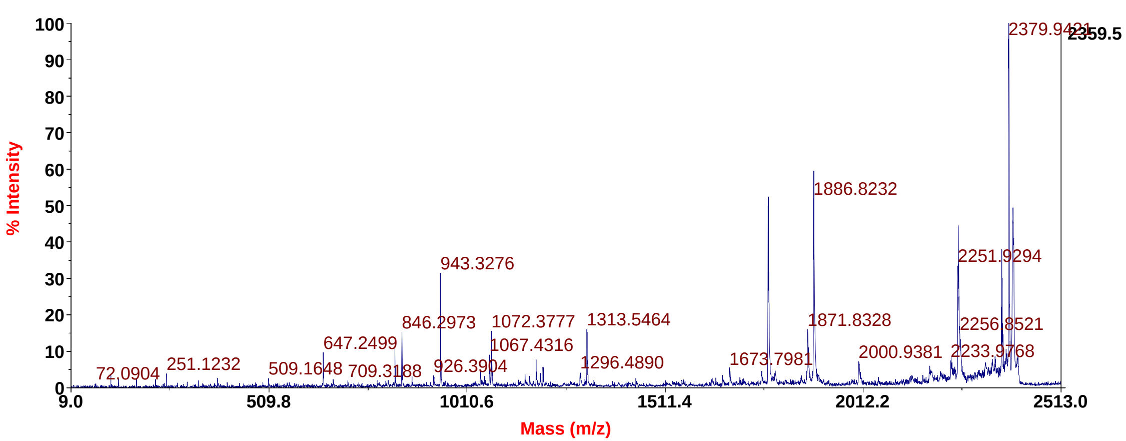


C:\...\E15_MSMS_2380.1445_21.t2d
Acquired:

**4700 MS/MS Precursor 2179.16 Spec #1 MC[BP = 647.3, 20840]**

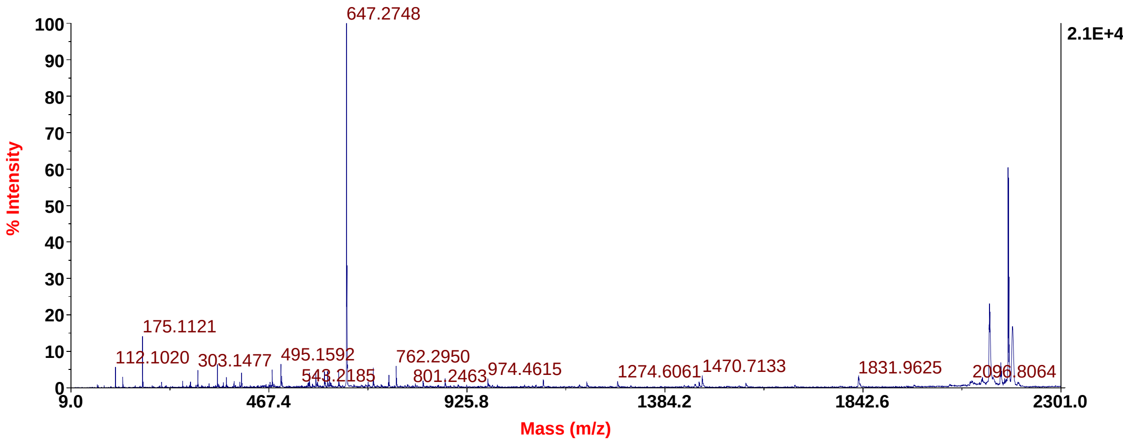


C:\...\E15_MSMS_2179.1560_13.t2d
Acquired:

**4700 MS/MS Precursor 2042.04 Spec #1 MC[BP = 2041.8, 3717]**

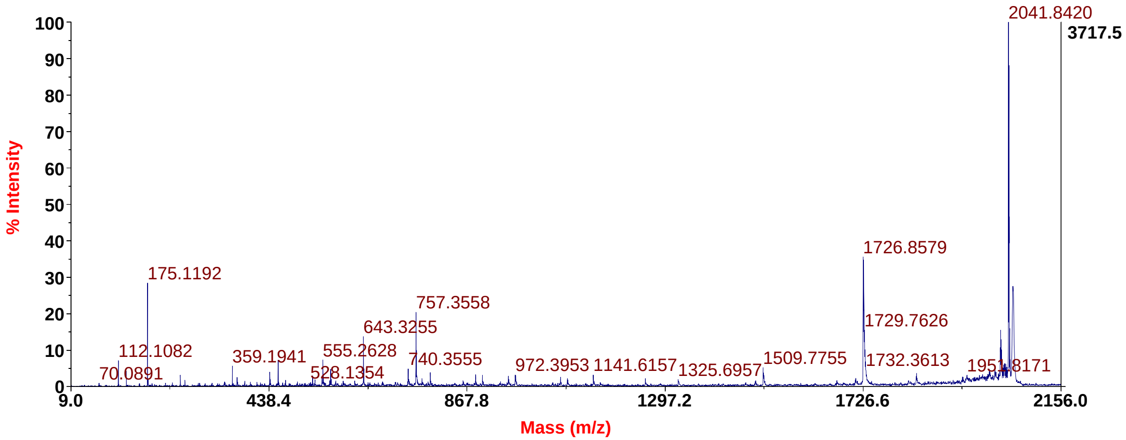


C:\...\E15_MSMS_2042.0374_20.t2d
Acquired:

**4700 MS/MS Precursor 1940.02 Spec #1 MC[BP = 1939.8, 4168]**

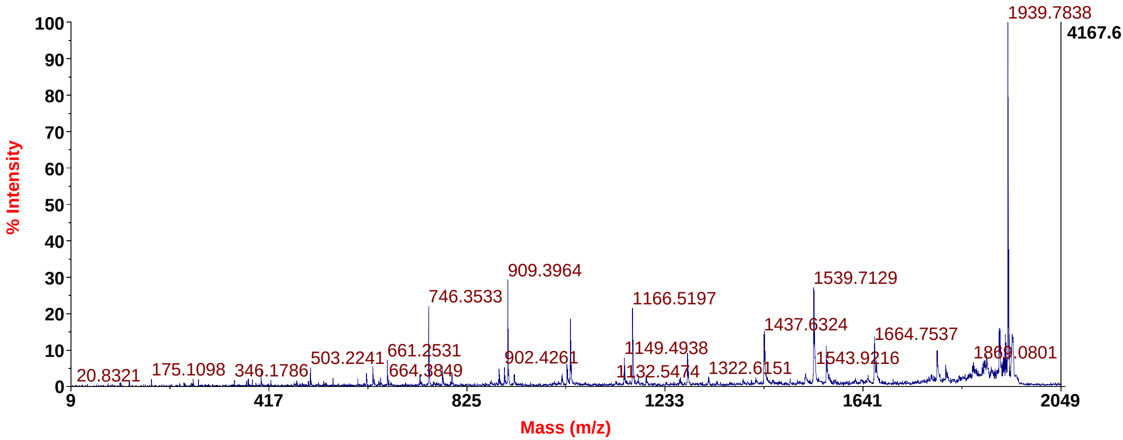


C:\...\E15_MSMS_1940.0164_19.t2d
Acquired:

**4700 MS/MS Precursor 1866.93 Spec #1 MC[BP = 1866.8, 3460]**

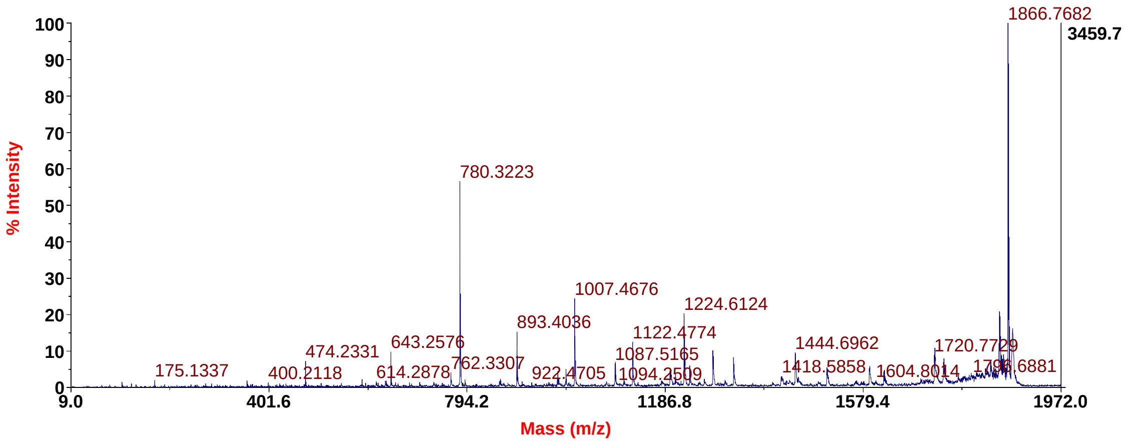


C:\...\E15_MSMS_1866.9338_18.t2d
Acquired:

E15_MSMS_2

4700 MS/MS Precursor 1375.75 Spec #1 MC[BP = 1375.6, 82577]

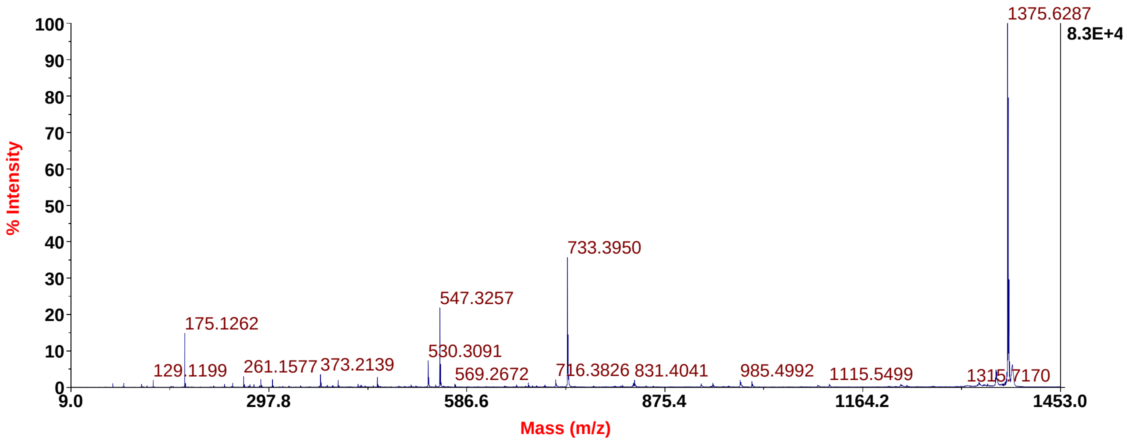


C:\...\E24_MSMS_1375.7471_13.t2d
Acquired:

4700 MS/MS Precursor 1264.63 Spec #1 MC[BP = 1264.5, 71304]

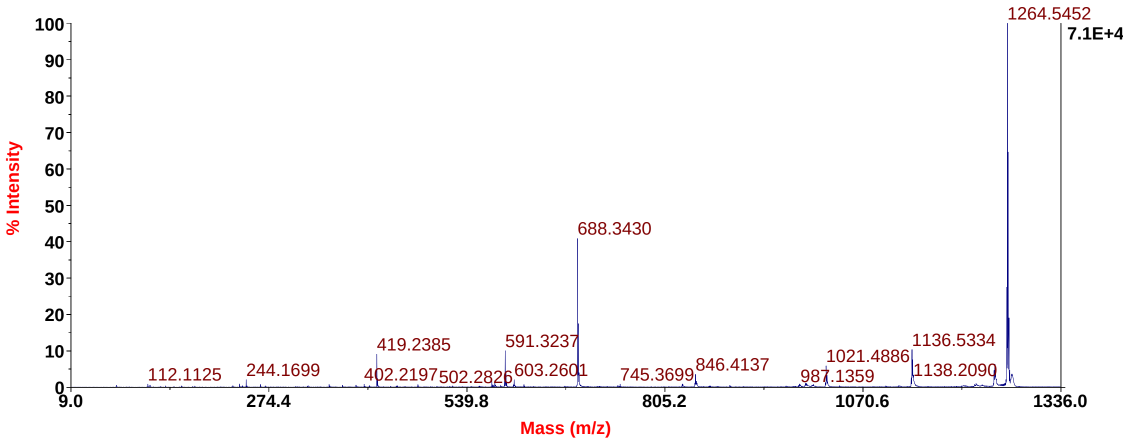


C:\...\E24_MSMS_1264.6343_14.t2d
Acquired:

4700 MS/MS Precursor 1129.64 Spec #1 MC[BP = 1129.6, 60284]

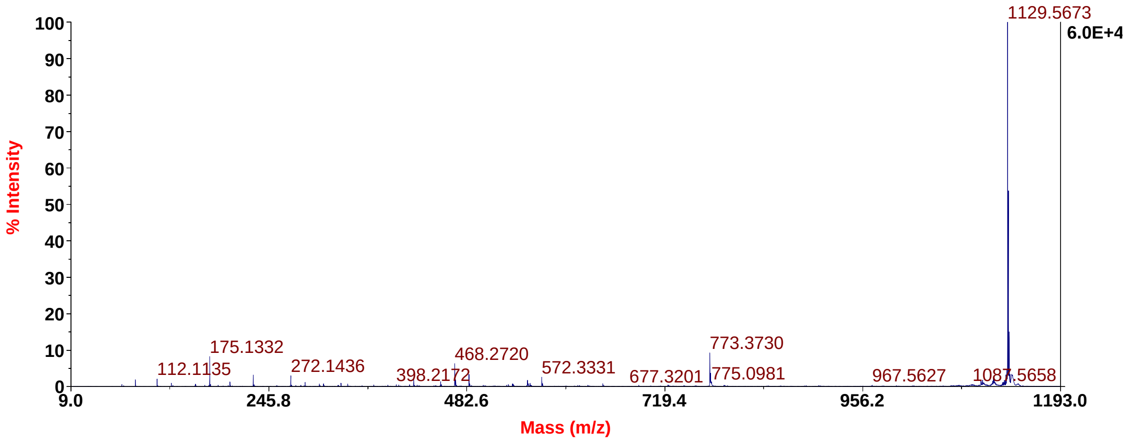


C:\...\E24_MSMS_1129.6353_15.t2d
Acquired:

4700 MS/MS Precursor 1113.64 Spec #1 MC[BP = 1113.6, 20226]

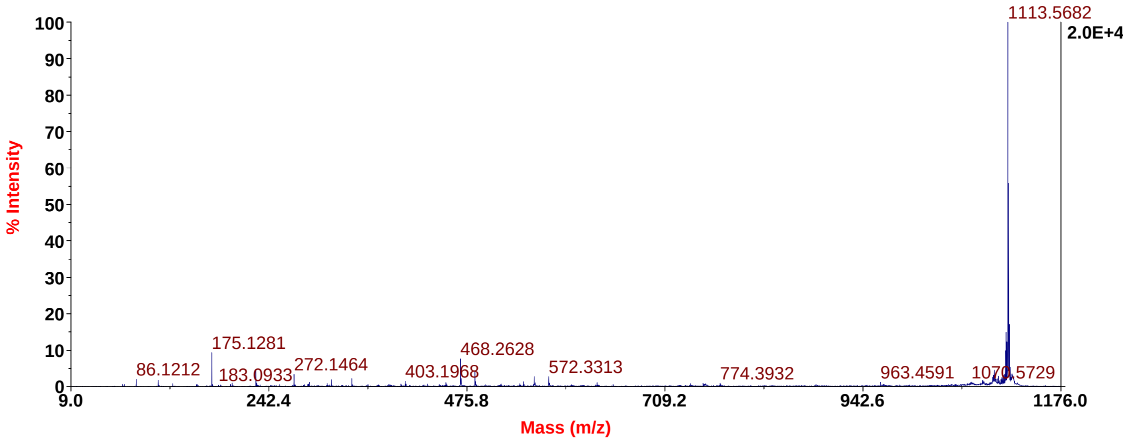


C:\...\E24_MSMS_1113.6406_22.t2d
Acquired:

4700 MS/MS Precursor 1097.64 Spec #1 MC[BP = 1097.6, 46162]

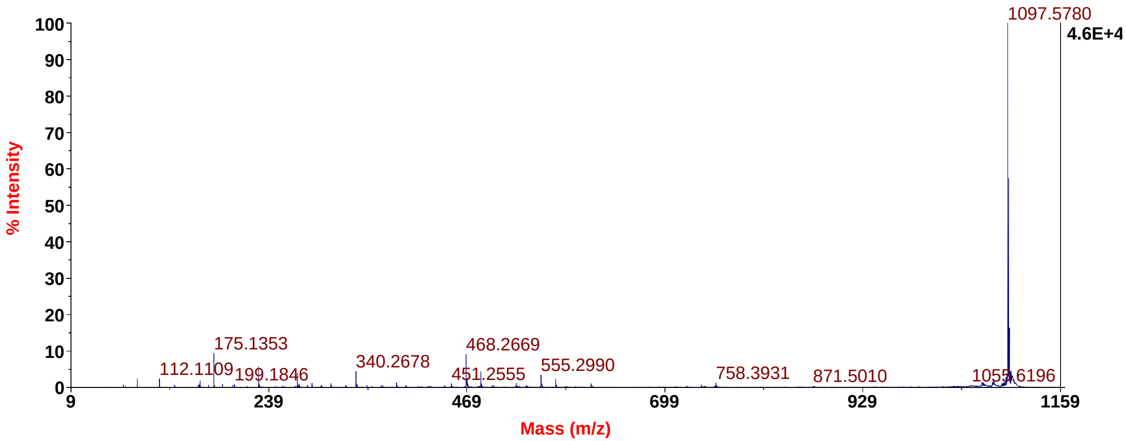


C:\...\E24_MSMS_1097.6448_16.t2d
Acquired:

E24_MSMS_1

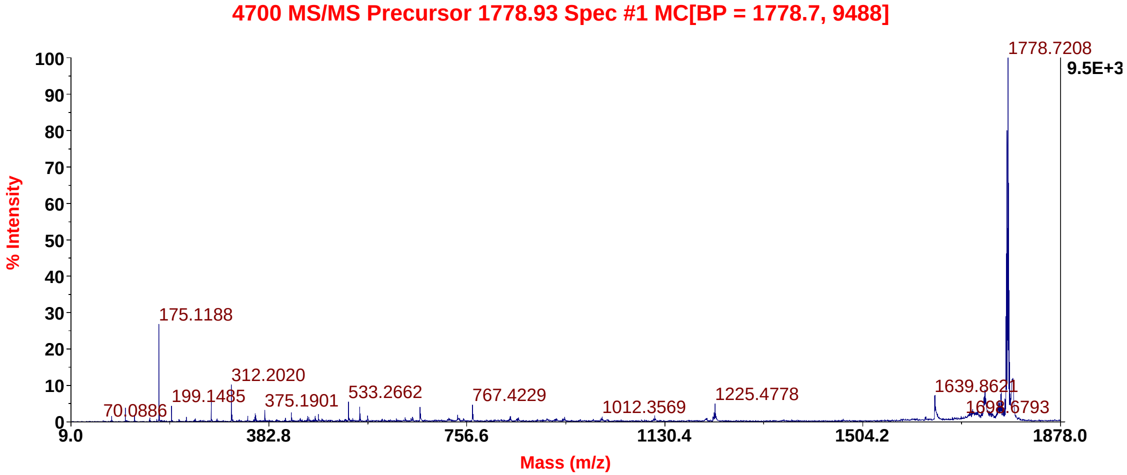


C:\...\E24_MSMS_1778.9310_19.t2d
Acquired:

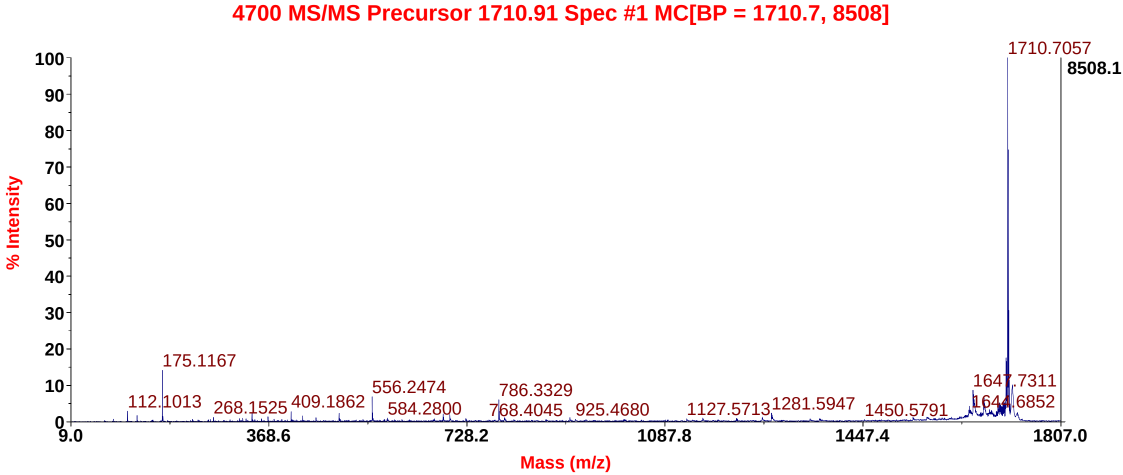


C:\...\E24_MSMS_1710.9093_20.t2d
Acquired:

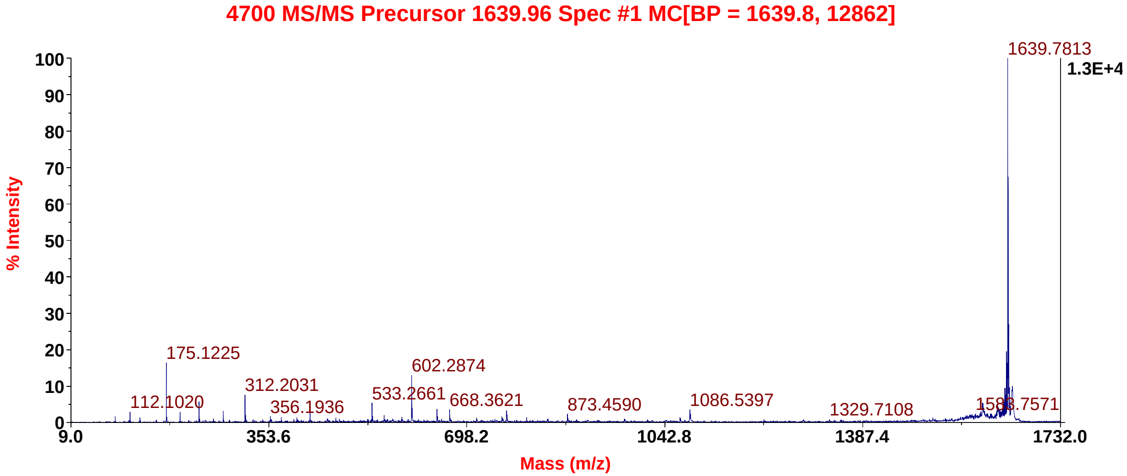


C:\...\E24_MSMS_1639.9624_18.t2d
Acquired:

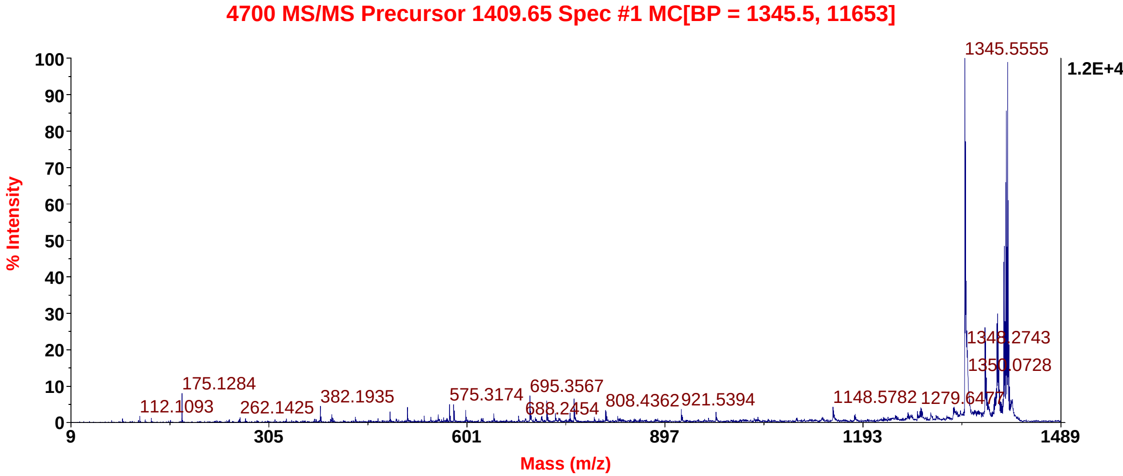


C:\...\E24_MSMS_1409.6488_21.t2d
Acquired:

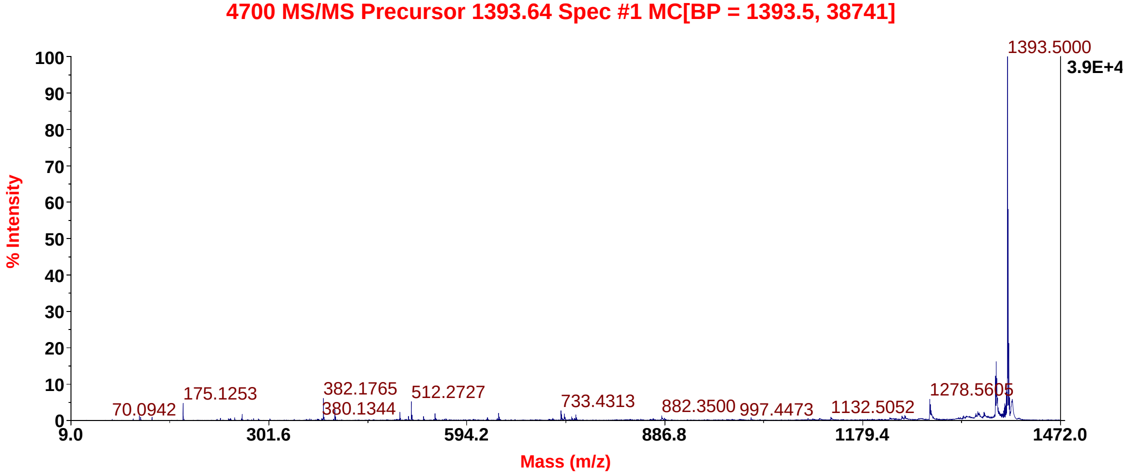


C:\...\E24_MSMS_1393.6389_17.t2d
Acquired:

E24_MSMS_2

4700 MS/MS Precursor 1495.84 Spec #1 MC[BP = 1495.7, 5452]

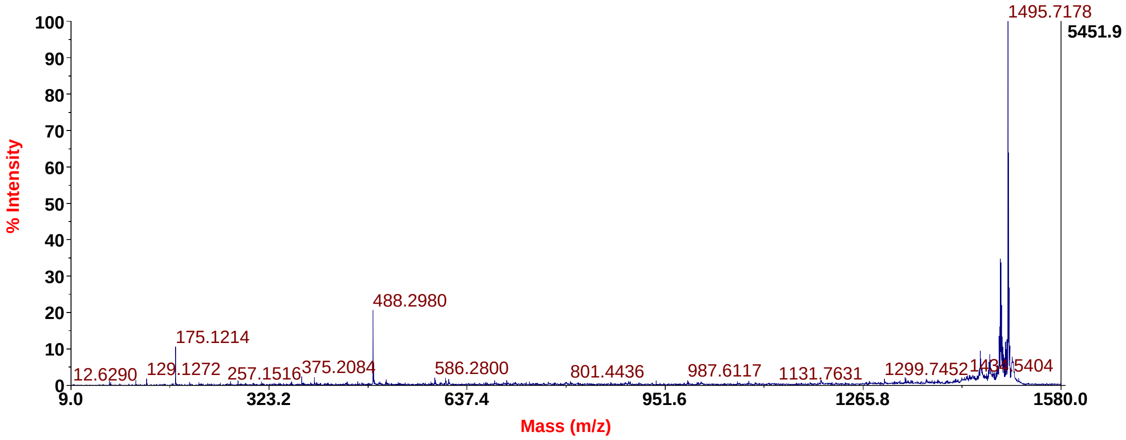


C:\...\F1_MSMS_1495.8385_17.t2d
Acquired:

4700 MS/MS Precursor 1483.7 Spec #1 MC[BP = 1483.6, 2554]

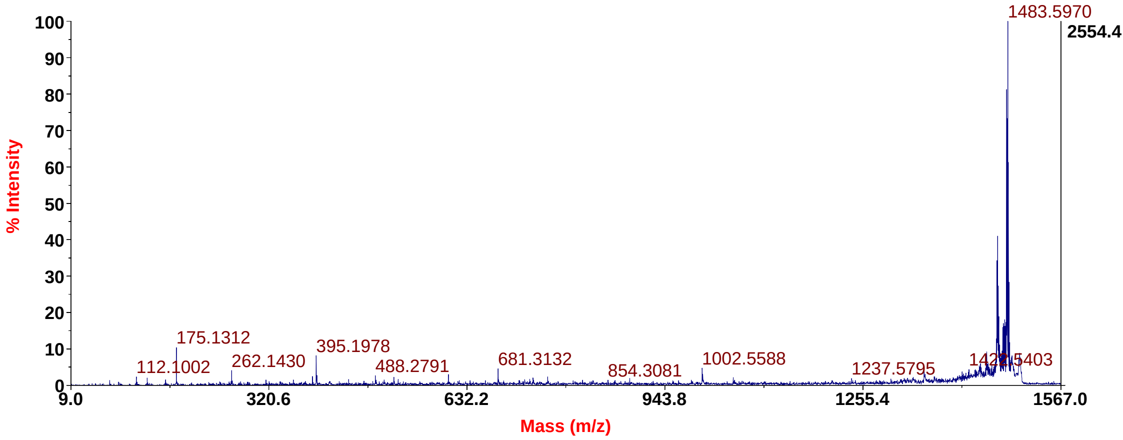


C:\...\F1_MSMS_1483.7003_21.t2d
Acquired:

4700 MS/MS Precursor 1466.66 Spec #1 MC[BP = 1466.5, 9715]

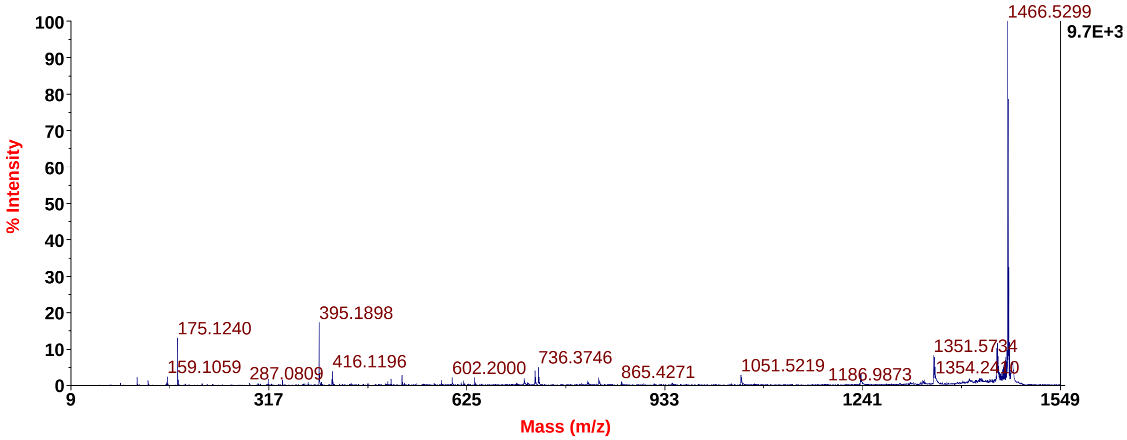


C:\...\F1_MSMS_1466.6602_14.t2d
Acquired:

4700 MS/MS Precursor 1449.64 Spec #1 MC[BP = 1449.5, 7345]

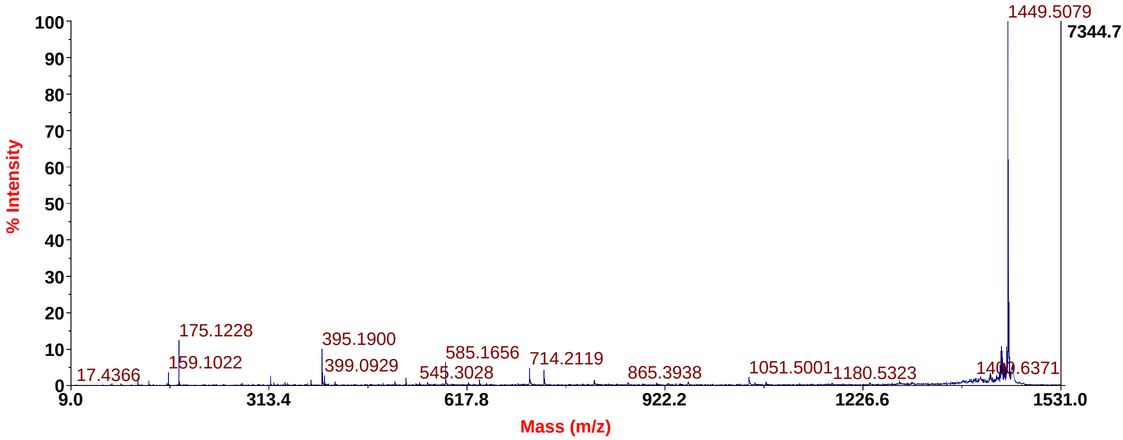


C:\...\F1_MSMS_1449.6410_15.t2d
Acquired:

4700 MS/MS Precursor 1150.62 Spec #1 MC[BP = 1150.6, 4377]

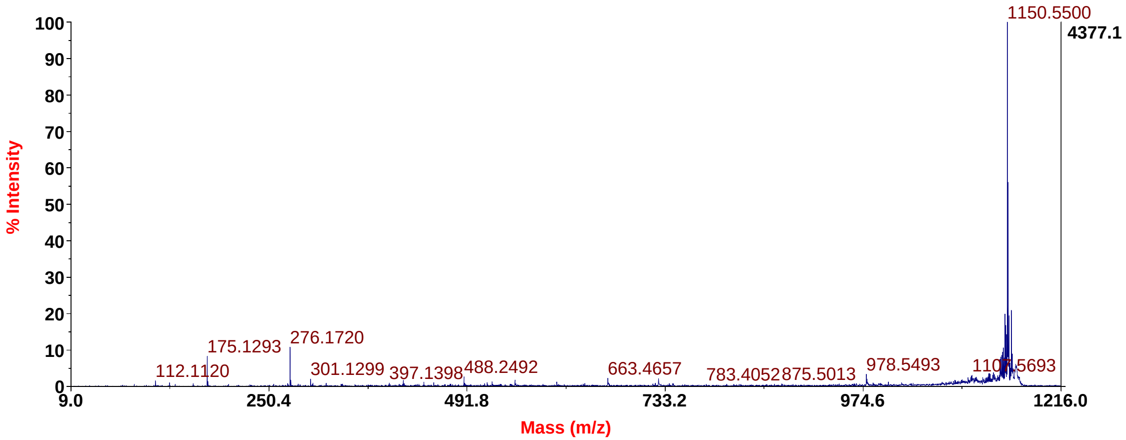


C:\...\F1_MSMS_1150.6195_20.t2d
Acquired:

F1_MSMS_1

**4700 MS/MS Precursor 2246 Spec #1 MC[BP = 335.1, 2419]**

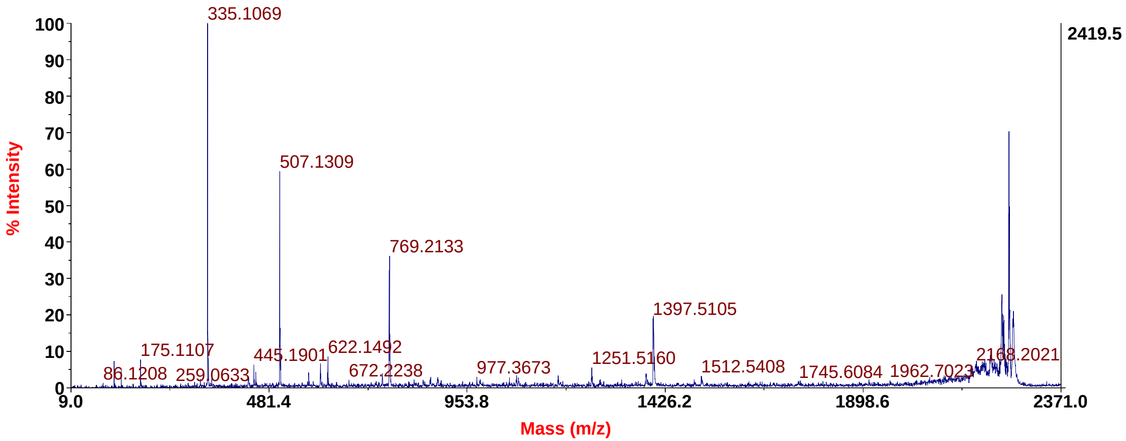


C:\...\F1_MSMS_2246.0007_16.t2d
Acquired:

**4700 MS/MS Precursor 1915.93 Spec #1 MC[BP = 1915.8, 840]**

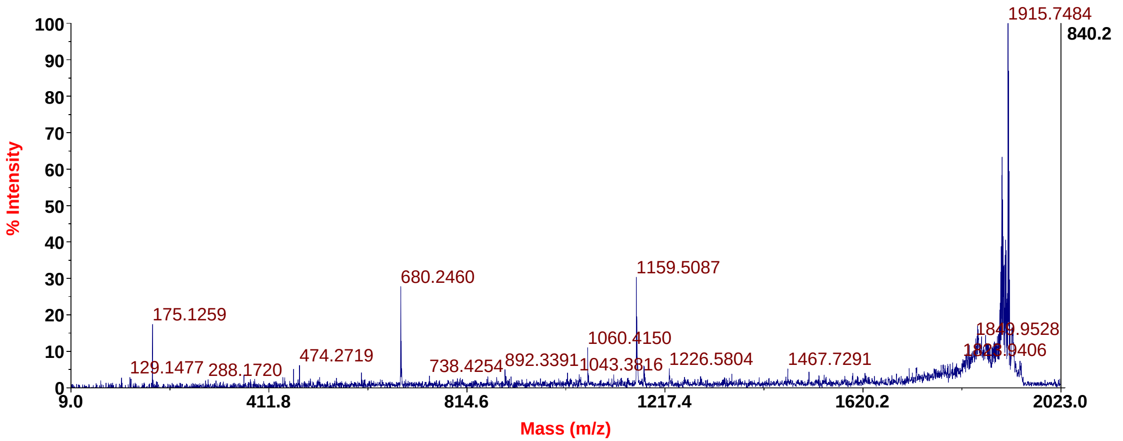


C:\...\F1_MSMS_1915.9301_22.t2d
Acquired:

**4700 MS/MS Precursor 1861.94 Spec #1 MC[BP = 1861.7, 1455]**

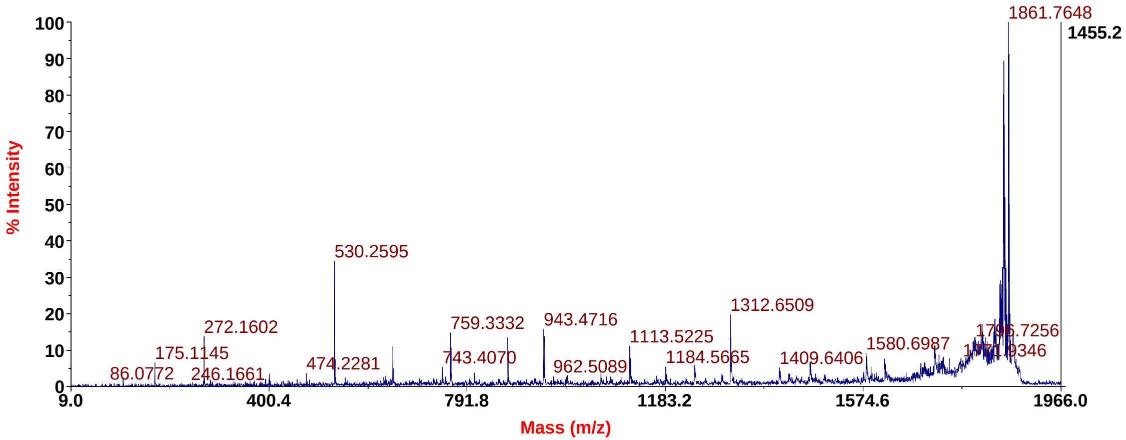


C:\...\F1_MSMS_1861.9357_19.t2d
Acquired:

**4700 MS/MS Precursor 1763.82 Spec #1 MC[BP = 1763.6, 2704]**

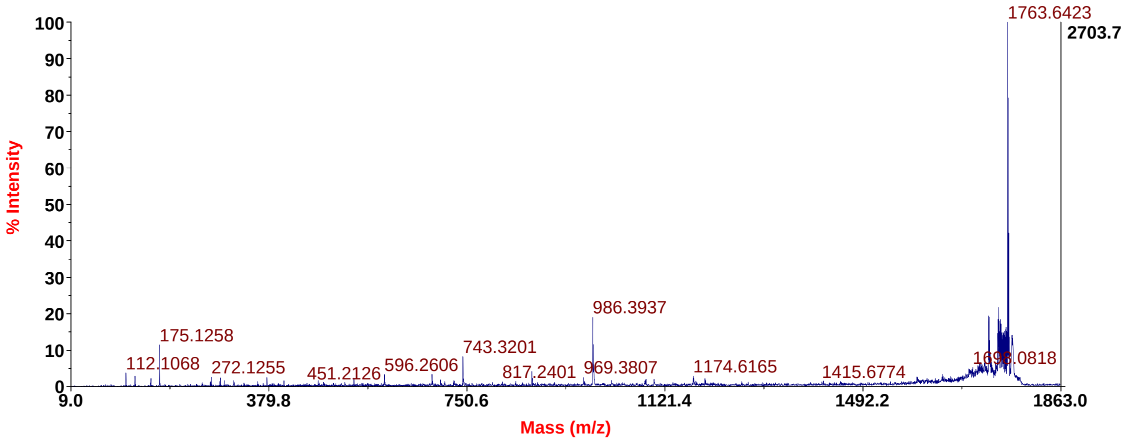


C:\...\F1_MSMS_1763.8232_18.t2d
Acquired:

**4700 MS/MS Precursor 1727.89 Spec #1 MC[BP = 1727.8, 7715]**

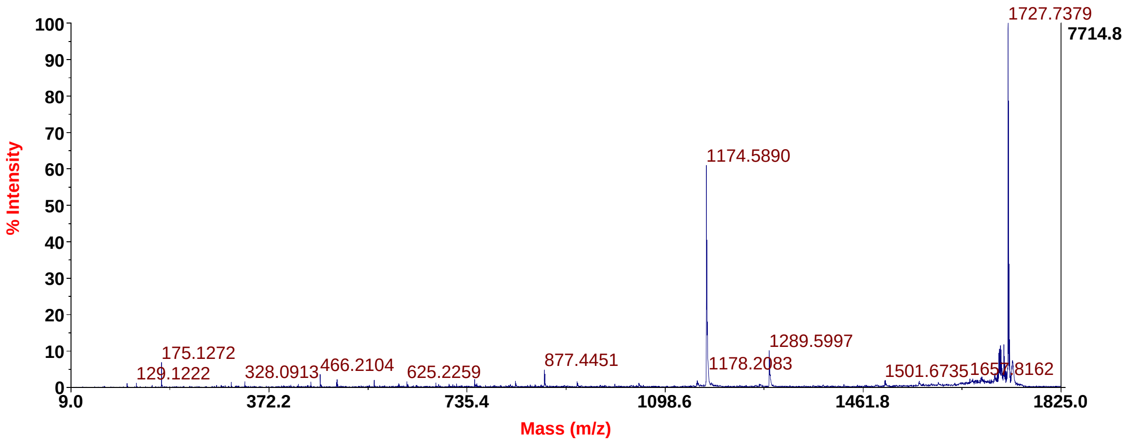


C:\...\F1_MSMS_1727.8885_13.t2d
Acquired:

# F1_MSMS_2

4700 MS/MS Precursor 1373.75 Spec #1 MC[BP = 1373.6, 16757]

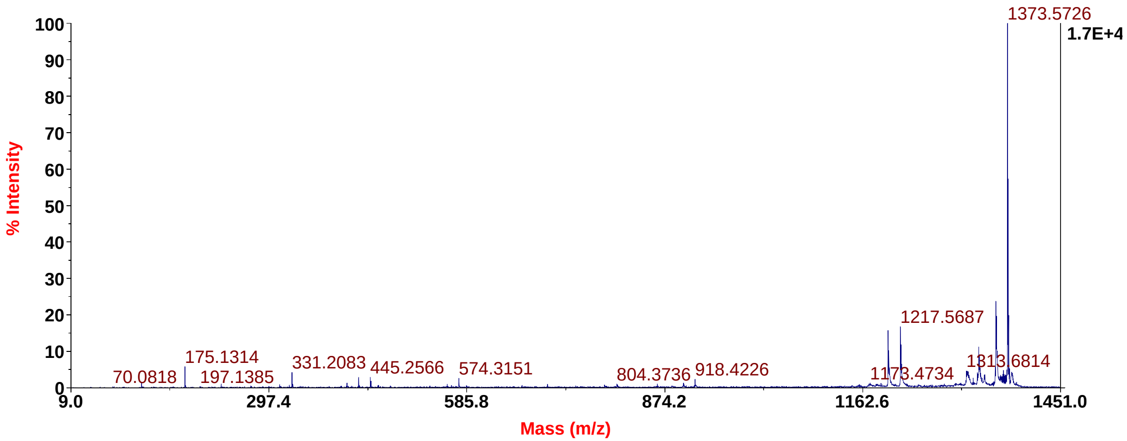


C:\...\F4_MSMS_1373.7477_15.t2d
Acquired:

4700 MS/MS Precursor 1211.59 Spec #1 MC[BP = 1211.5, 14531]

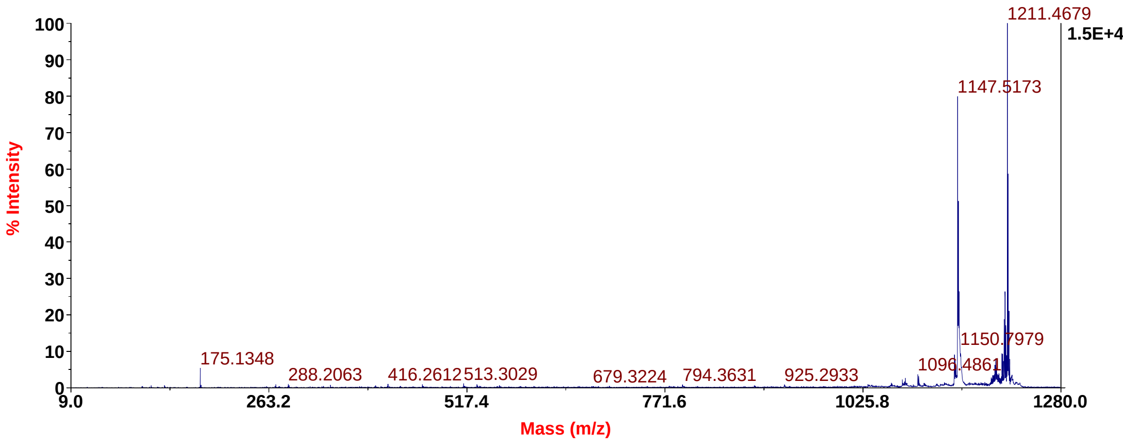


C:\...\F4_MSMS_1211.5892_18.t2d
Acquired:

4700 MS/MS Precursor 1195.59 Spec #1 MC[BP = 1195.5, 26960]

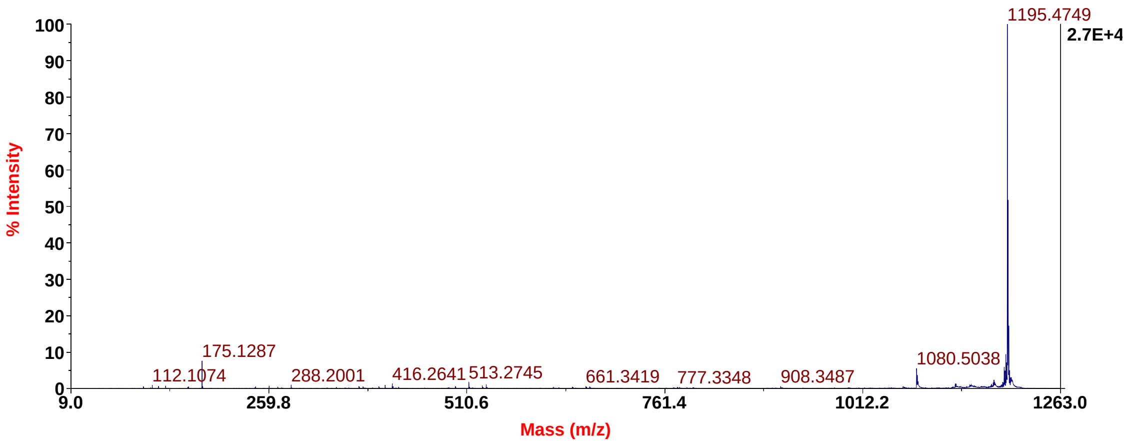


C:\...\F4_MSMS_1195.5948_16.t2d
Acquired:

4700 MS/MS Precursor 1125.61 Spec #1 MC[BP = 1125.5, 25173]

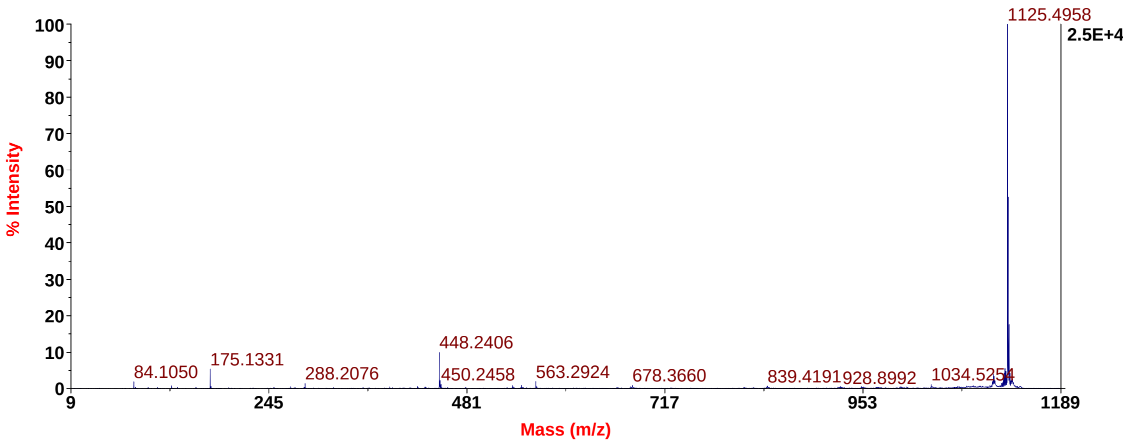


C:\...\F4_MSMS_1125.6052_17.t2d
Acquired:

4700 MS/MS Precursor 997.494 Spec #1 MC[BP = 997.4, 35563]

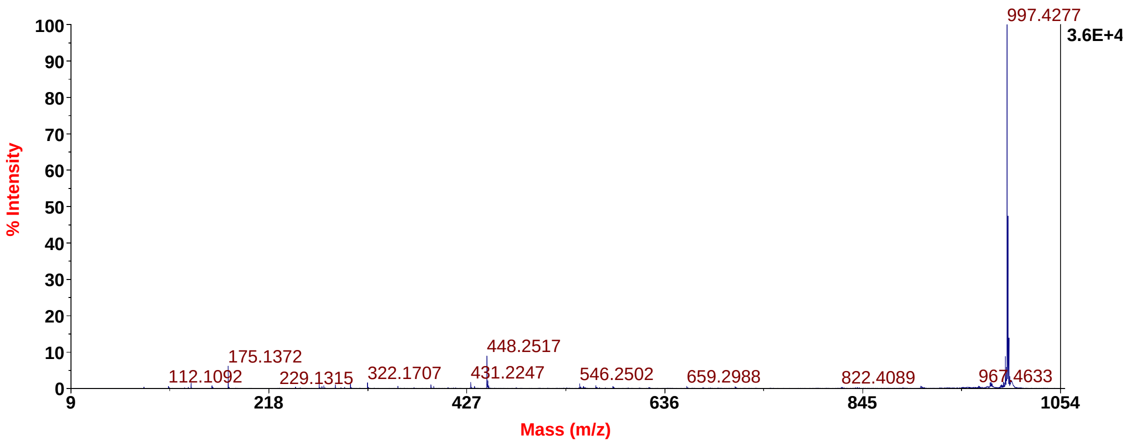


C:\...\F4_MSMS_997.4941_14.t2d
Acquired:

F4_MSMS_1

**4700 MS/MS Precursor 3176.91 Spec #1 MC[BP = 668.2, 2846]**

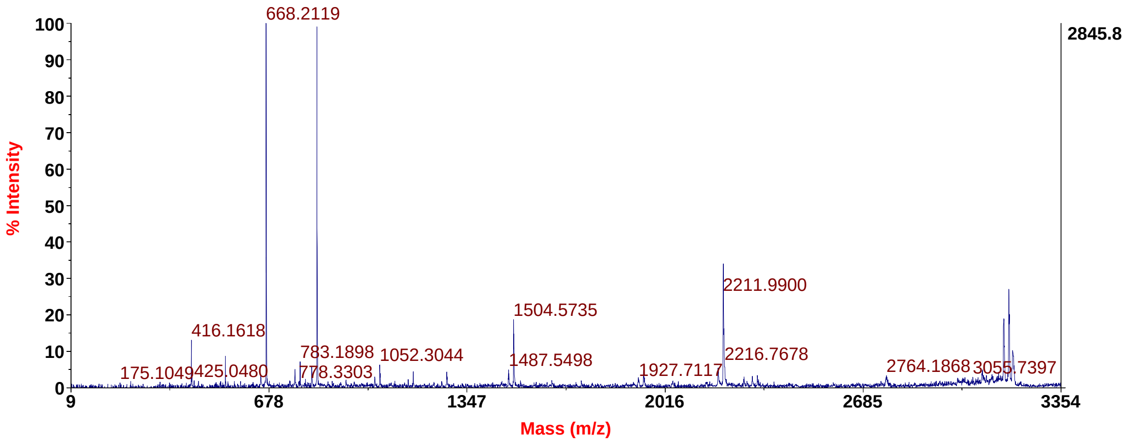


C:\...\F4_MSMS_3176.9102_22.t2d
Acquired:

**4700 MS/MS Precursor 2203.33 Spec #1 MC[BP = 2192.9, 4578]**

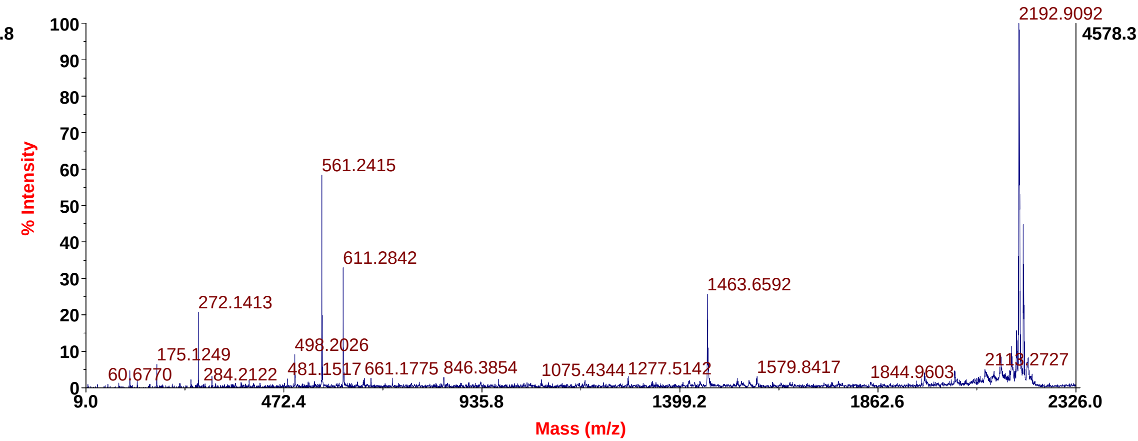


C:\...\F4_MSMS_2203.3330_20.t2d
Acquired:

**4700 MS/MS Precursor 2042.21 Spec #1 MC[BP = 2041.8, 12808]**

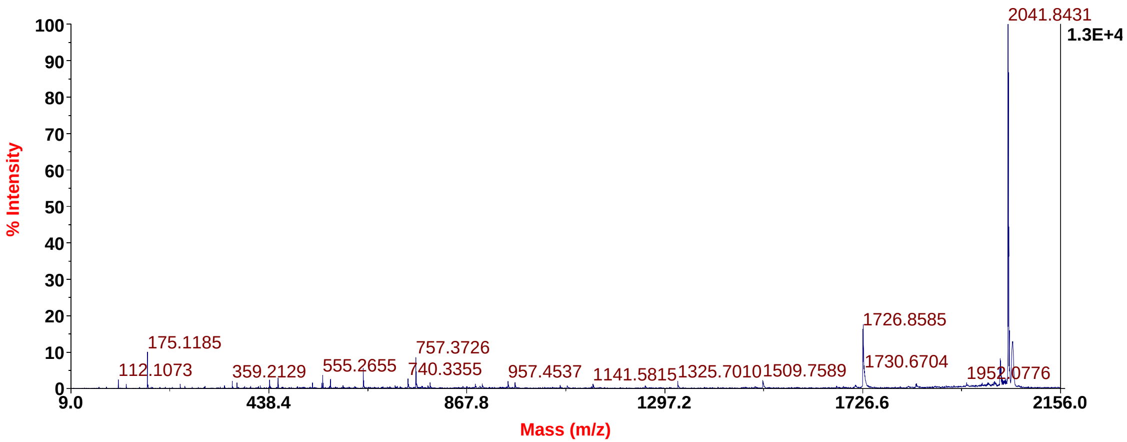


C:\...\F4_MSMS_2042.2073_13.t2d
Acquired:

**4700 MS/MS Precursor 1882 Spec #1 MC[BP = 1881.7, 3299]**

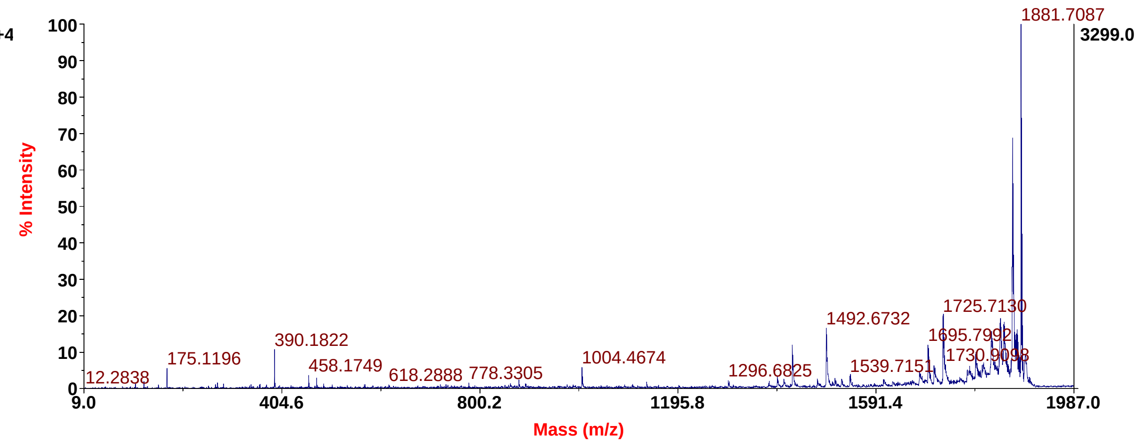


C:\...\F4_MSMS_1882.0017_21.t2d
Acquired:

**4700 MS/MS Precursor 1556.88 Spec #1 MC[BP = 1556.7, 7095]**

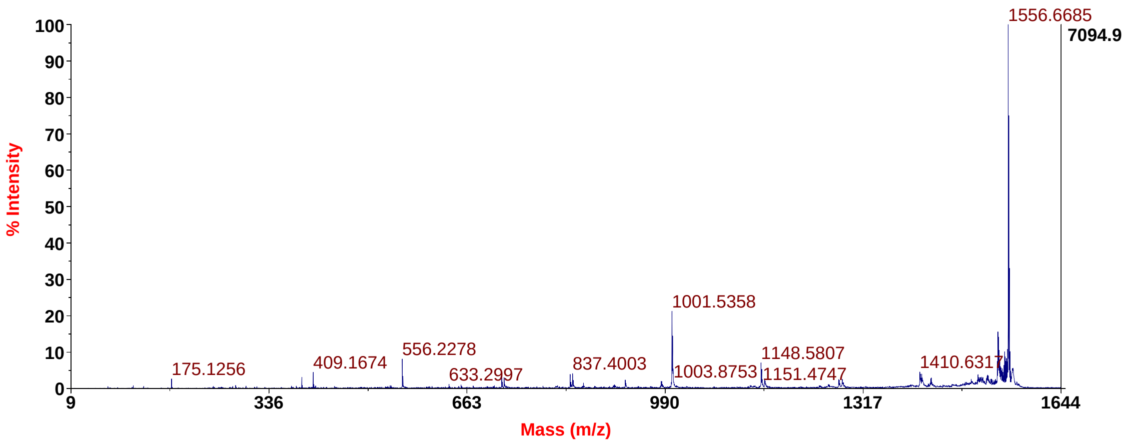


C:\...\F4_MSMS_1556.8794_19.t2d
Acquired:

# F4_MSMS_2

4700 MS/MS Precursor 1550.55 Spec #1 MC[BP = 1550.6, 8791]

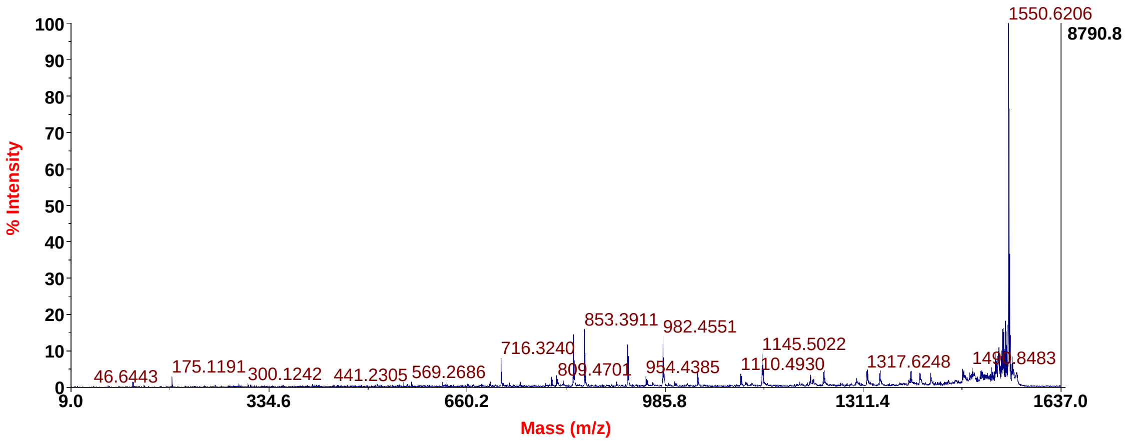


C:\...\F5_MSMS_1550.5463_17.t2d
Acquired:

4700 MS/MS Precursor 1529.5 Spec #1 MC[BP = 1522.7, 7839]

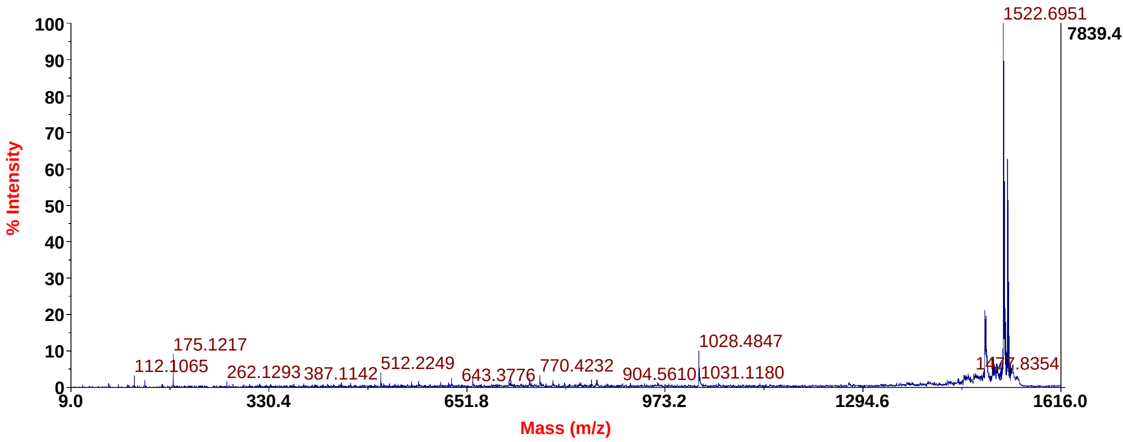


C:\...\F5_MSMS_1529.5024_21.t2d
Acquired:

4700 MS/MS Precursor 1193.32 Spec #1 MC[BP = 1193.6, 68904]

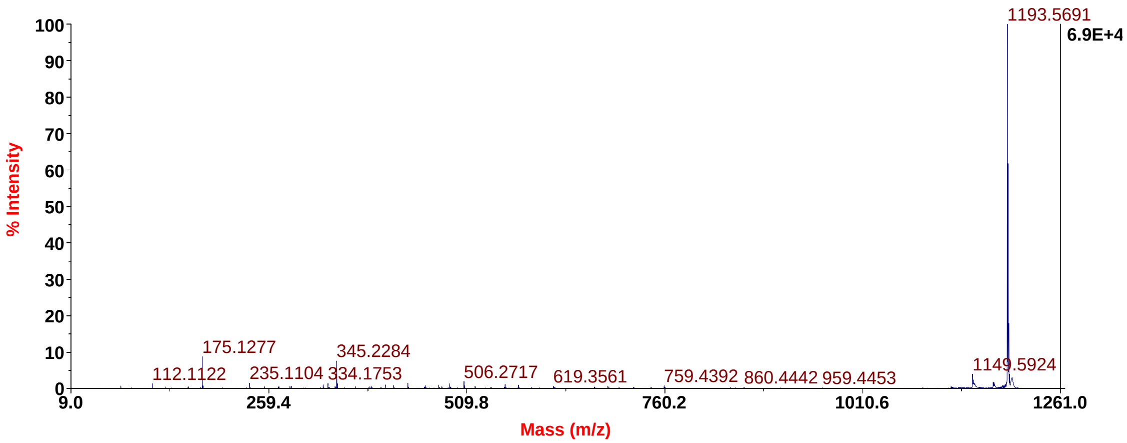


C:\...\F5_MSMS_1193.3182_14.t2d
Acquired:

4700 MS/MS Precursor 1141.18 Spec #1 MC[BP = 1141.5, 10994]

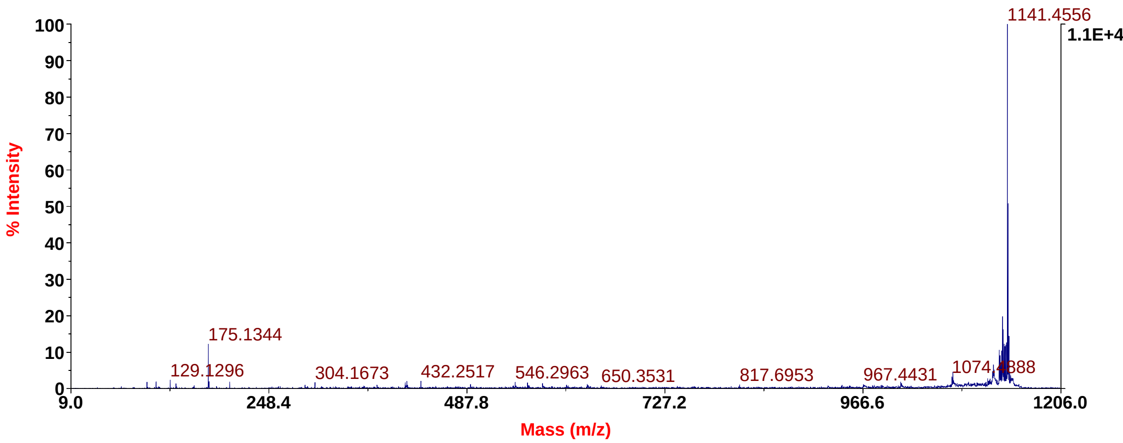


C:\...\F5_MSMS_1141.1798_20.t2d
Acquired:

4700 MS/MS Precursor 1044.08 Spec #1 MC[BP = 1044.4, 6279]

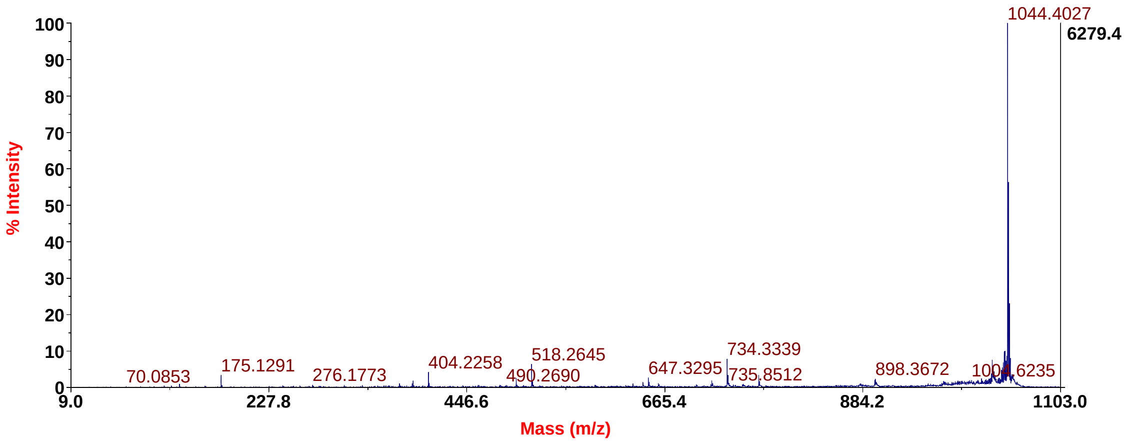


C:\...\F5_MSMS_1044.0767_19.t2d
Acquired:

# F5_MSMS_1

**4700 MS/MS Precursor 2042.06 Spec #1 MC[BP = 2041.8, 2954]**

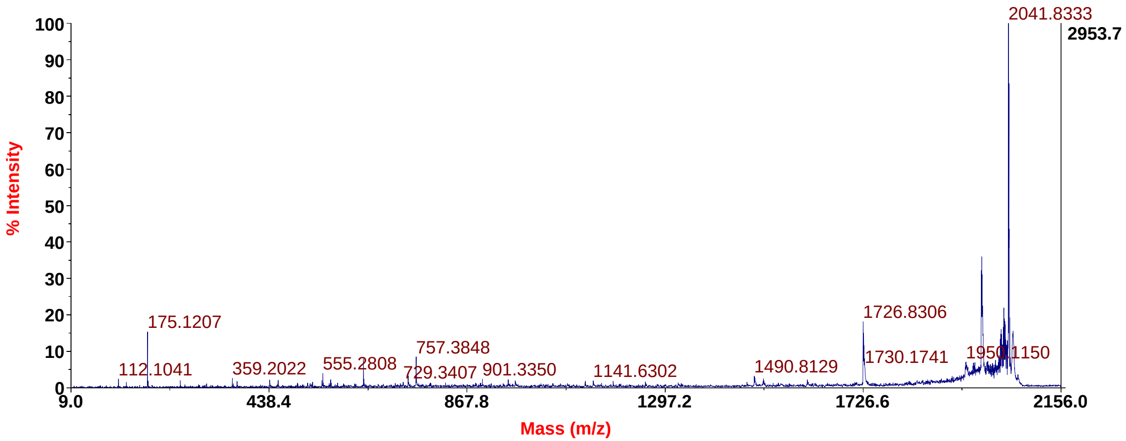


C:\...\F5_MSMS_2042.0649_22.t2d
Acquired:

**4700 MS/MS Precursor 1982.96 Spec #1 MC[BP = 1982.7, 12512]**

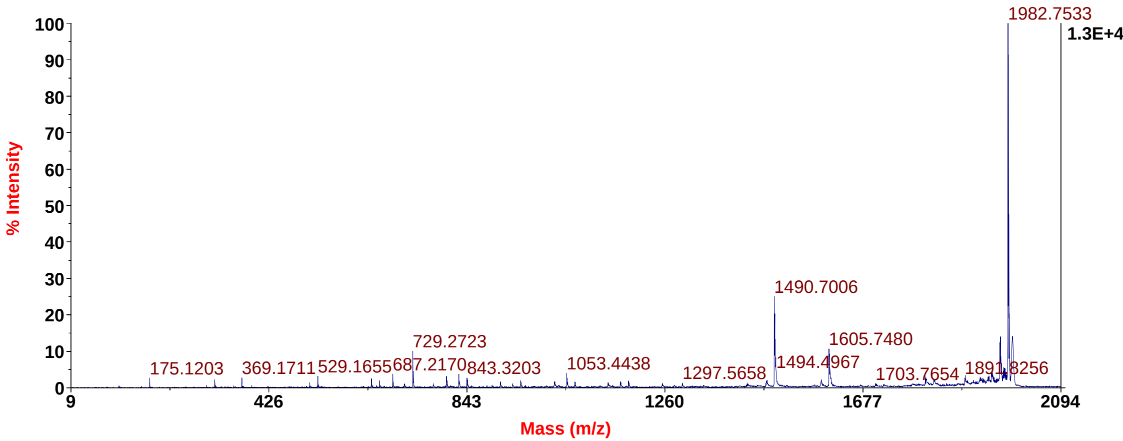


C:\...\F5_MSMS_1982.9603_16.t2d
Acquired:

**4700 MS/MS Precursor 1719.81 Spec #1 MC[BP = 1711.8, 82199]**

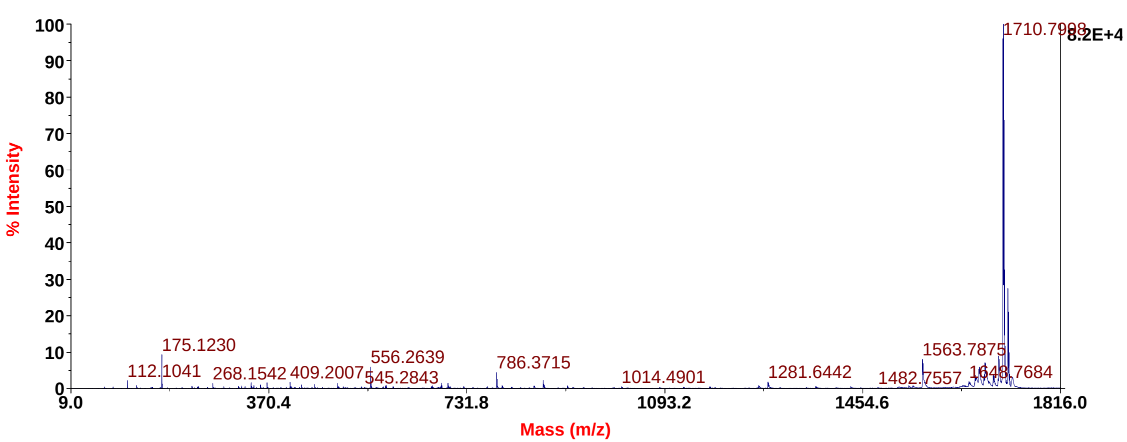


C:\...\F5_MSMS_1719.8083_15.t2d
Acquired:

**4700 MS/MS Precursor 1710.74 Spec #1 MC[BP = 1710.7, 71855]**

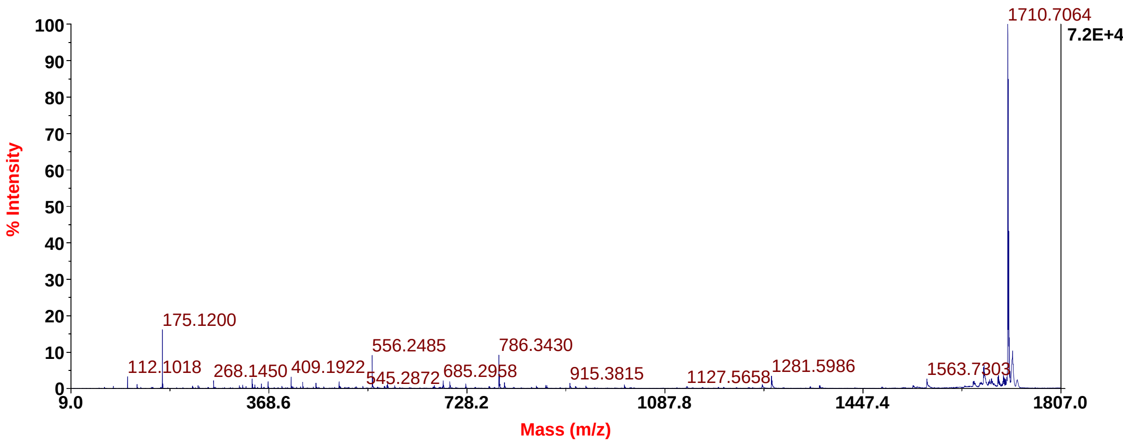


C:\...\F5_MSMS_1710.7411_13.t2d
Acquired:

**4700 MS/MS Precursor 1580.62 Spec #1 MC[BP = 1580.7, 4428]**

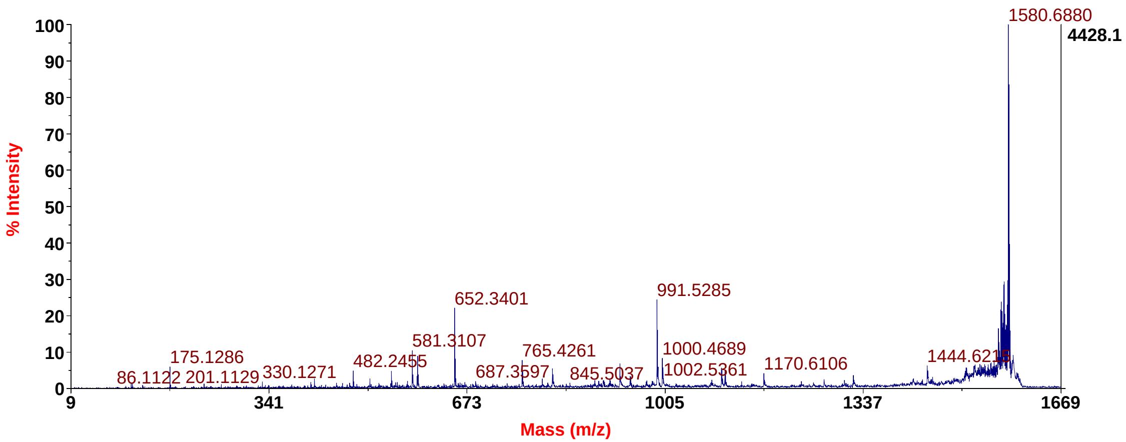


C:\...\F5_MSMS_1580.6240_18.t2d
Acquired:

# F5_MSMS_2

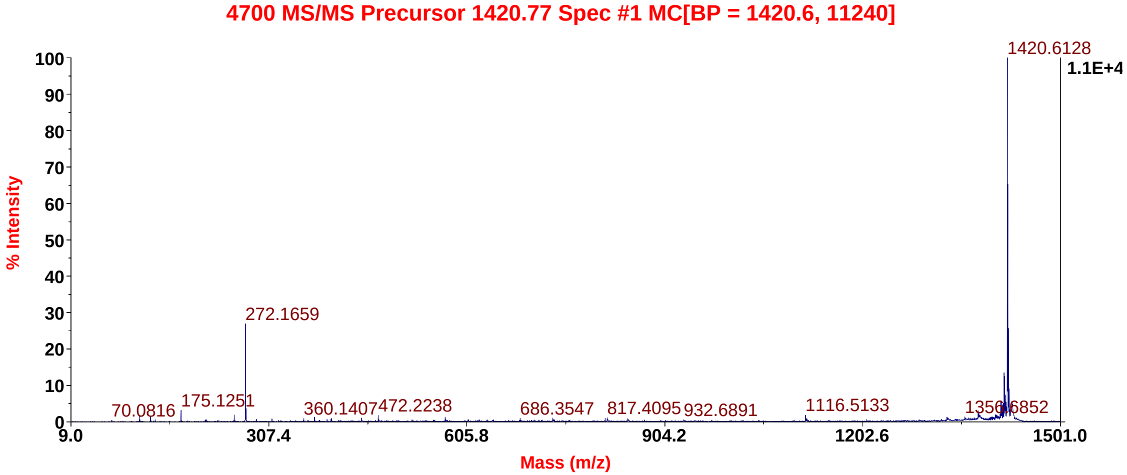


C:\...\F8_MSMS_1420.7706_15.t2d
Acquired:

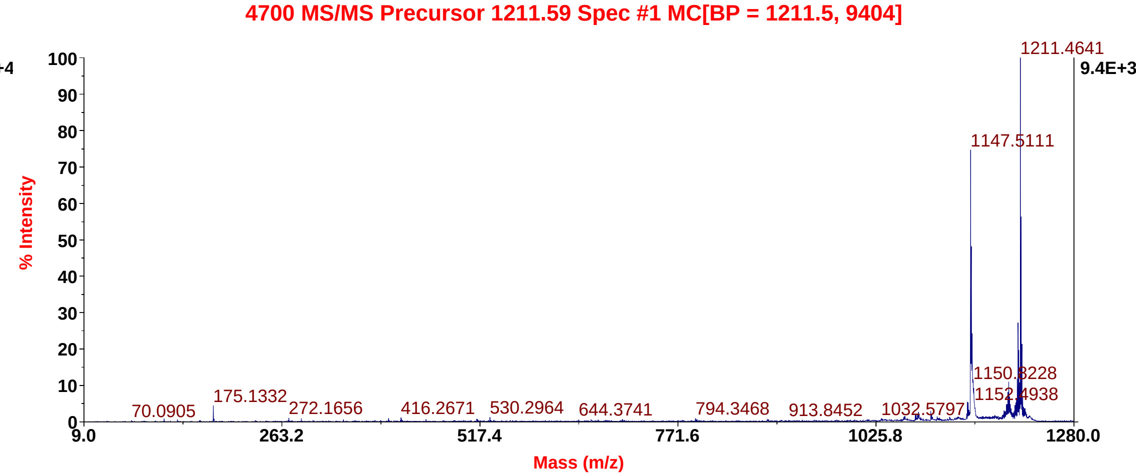


C:\...\F8_MSMS_1211.5906_20.t2d
Acquired:

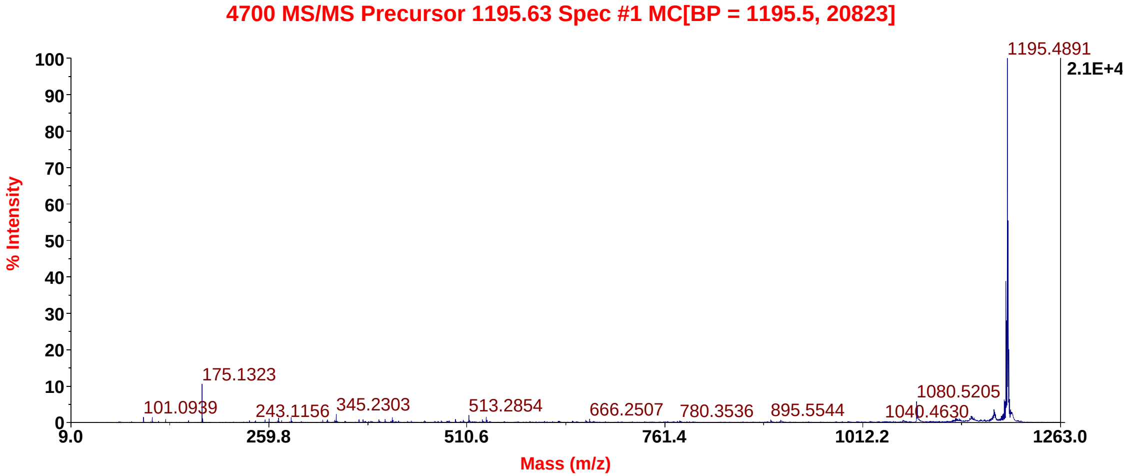


C:\...\F8_MSMS_1195.6337_16.t2d
Acquired:

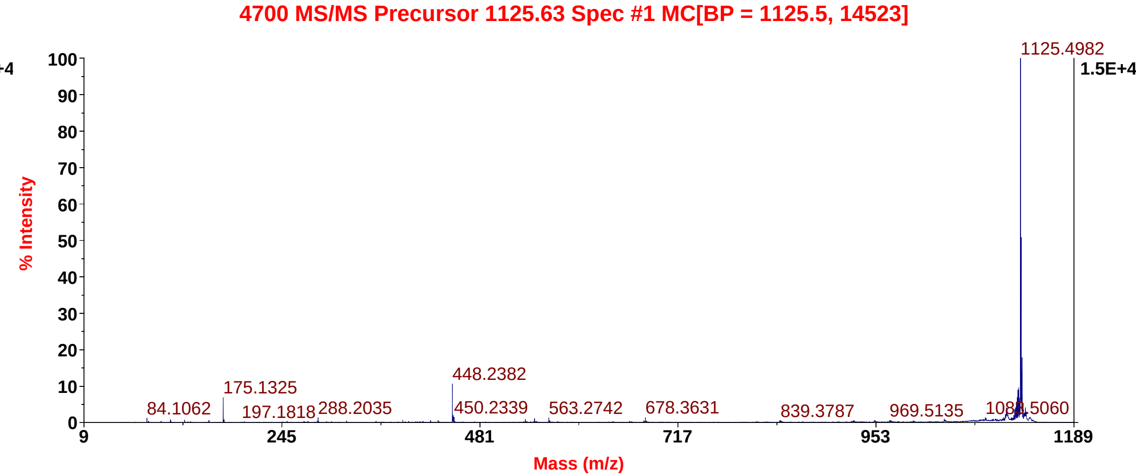


C:\...\F8_MSMS_1125.6273_18.t2d
Acquired:

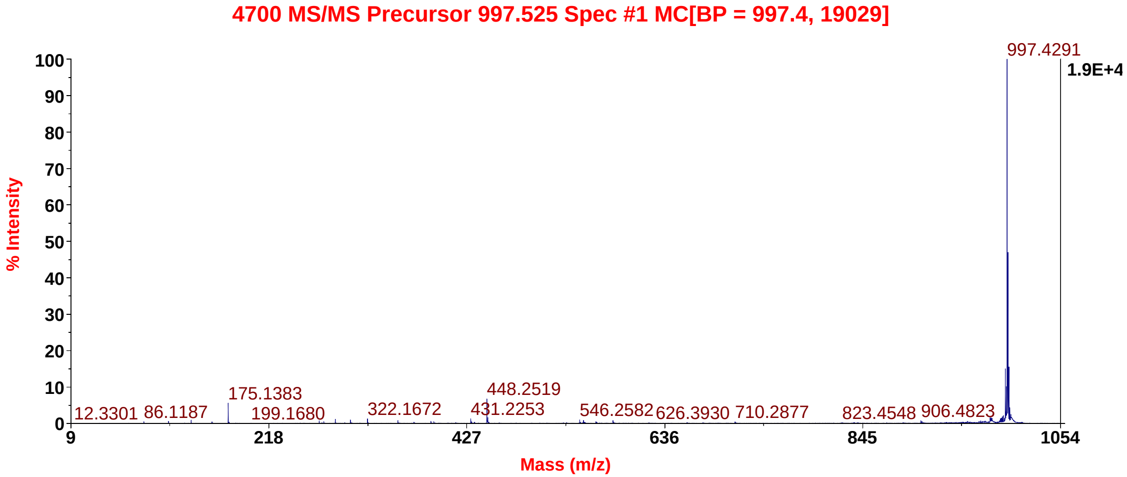


C:\...\F8_MSMS_997.5250_13.t2d
Acquired:

F8_MSMS_1

4700 MS/MS Precursor 2549.32 Spec #1 MC[BP = 2550.0, 2062]

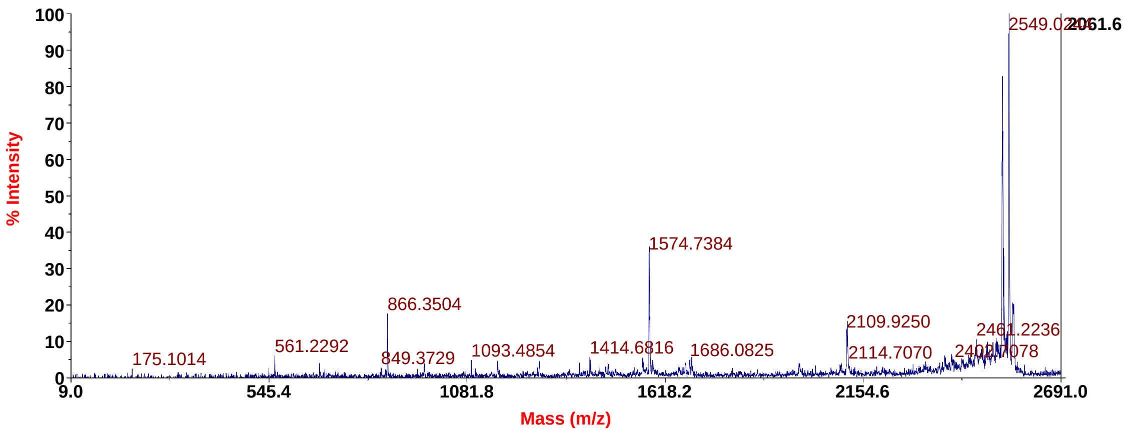


C:\...\F8_MSMS_2549.3208_21.t2d
Acquired:

4700 MS/MS Precursor 2203.17 Spec #1 MC[BP = 2202.9, 2161]

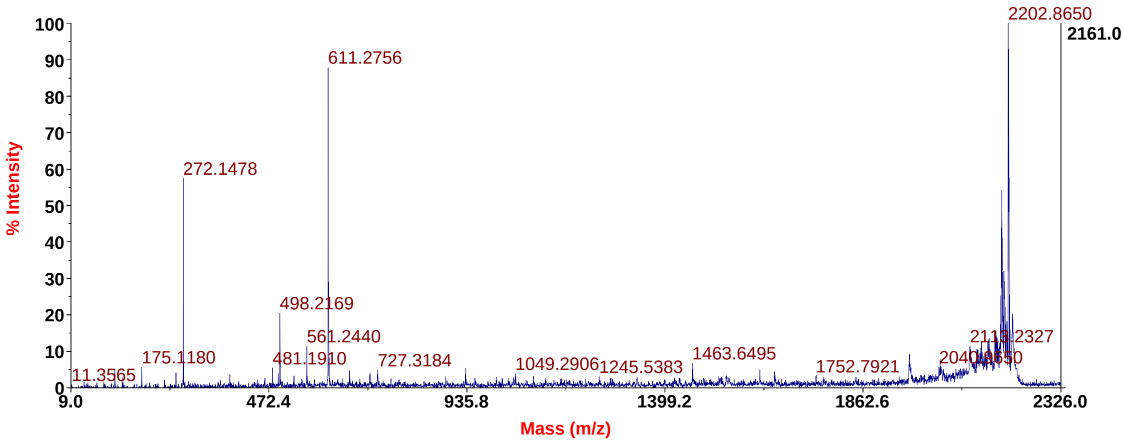


C:\...\F8_MSMS_2203.1709_14.t2d
Acquired:

4700 MS/MS Precursor 1772.92 Spec #1 MC[BP = 1772.7, 6885]

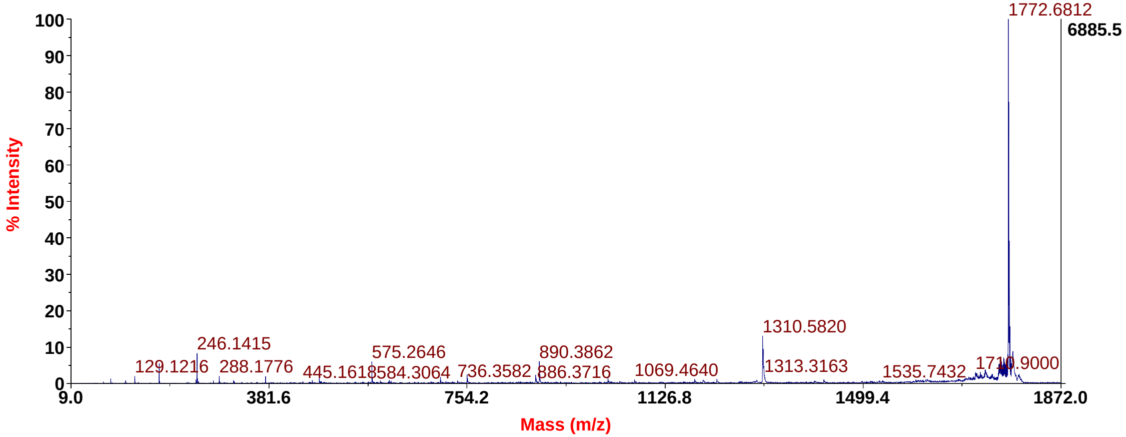


C:\...\F8_MSMS_1772.9197_17.t2d
Acquired:

4700 MS/MS Precursor 1710.95 Spec #1 MC[BP = 1710.7, 5115]

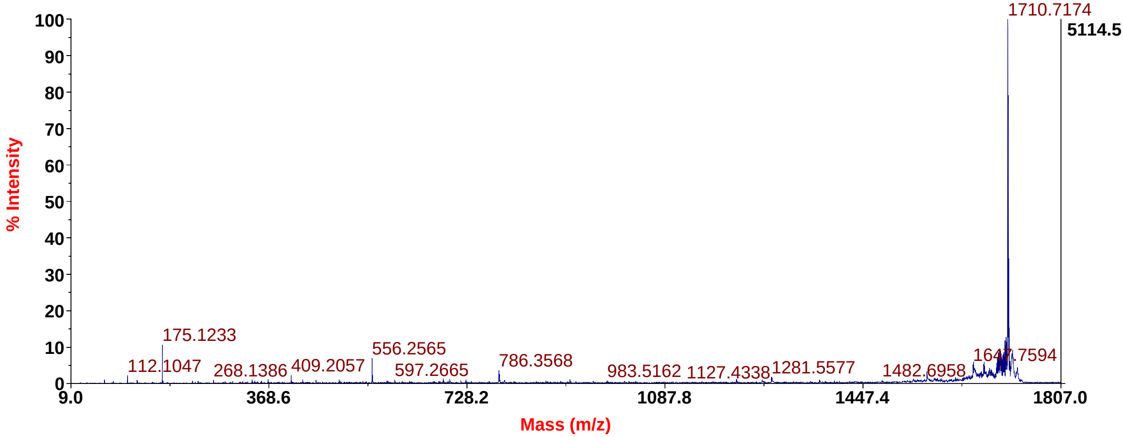


C:\...\F8_MSMS_1710.9480_19.t2d
Acquired:

4700 MS/MS Precursor 1576.89 Spec #1 MC[BP = 1576.7, 5816]

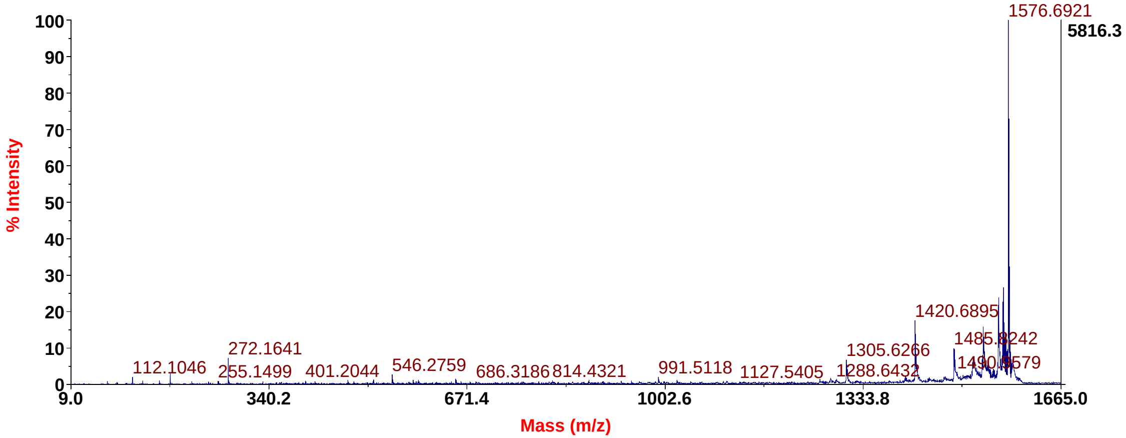


C:\...\F8_MSMS_1576.8851_22.t2d
Acquired:

F8_MSMS_2

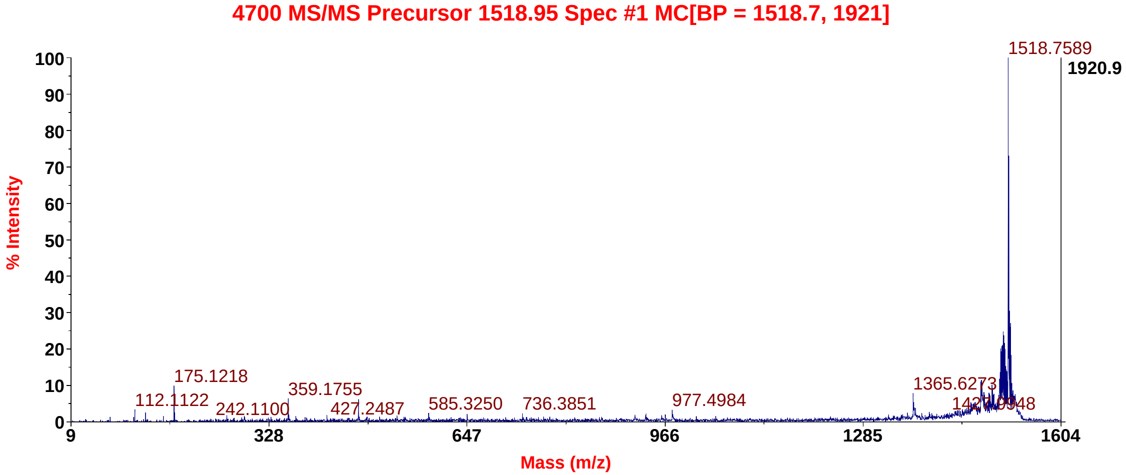


C:\...\F9_MSMS_1518.9531_22.t2d
Acquired:

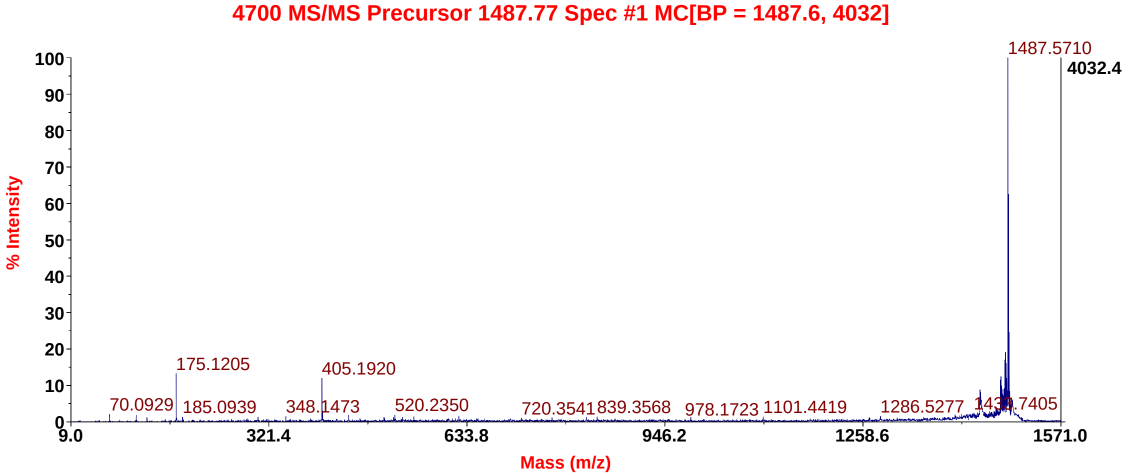


C:\...\F9_MSMS_1487.7661_14.t2d
Acquired:

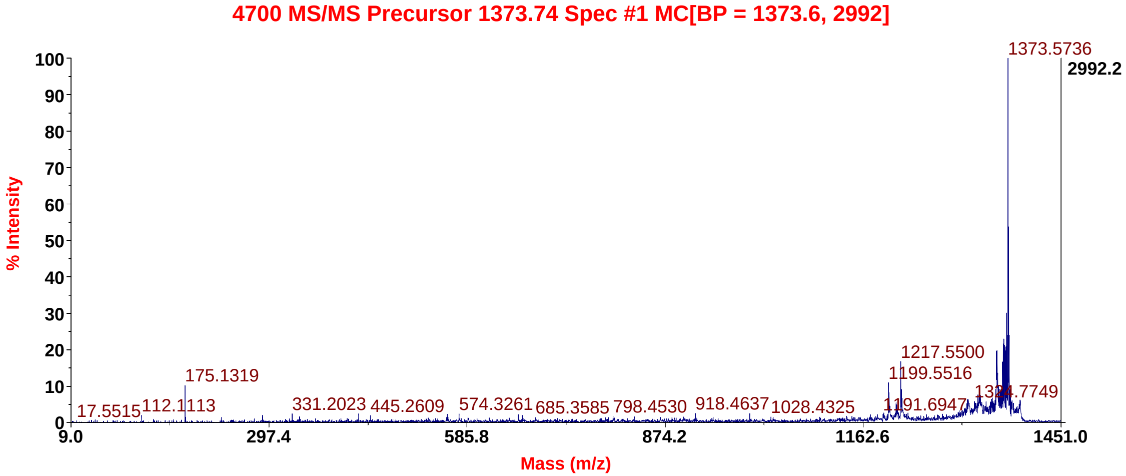


C:\...\F9_MSMS_1373.7427_21.t2d
Acquired:

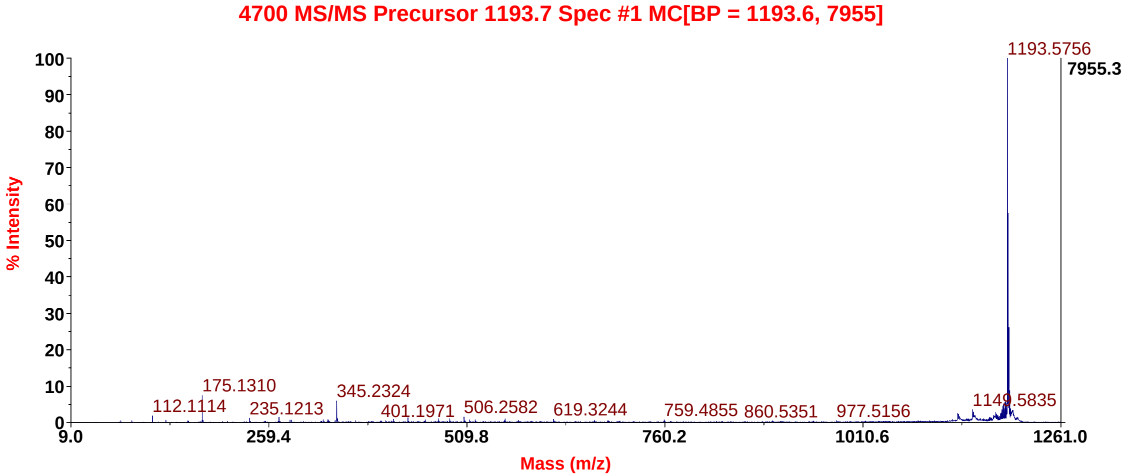


C:\...\F9_MSMS_1193.7023_16.t2d
Acquired:

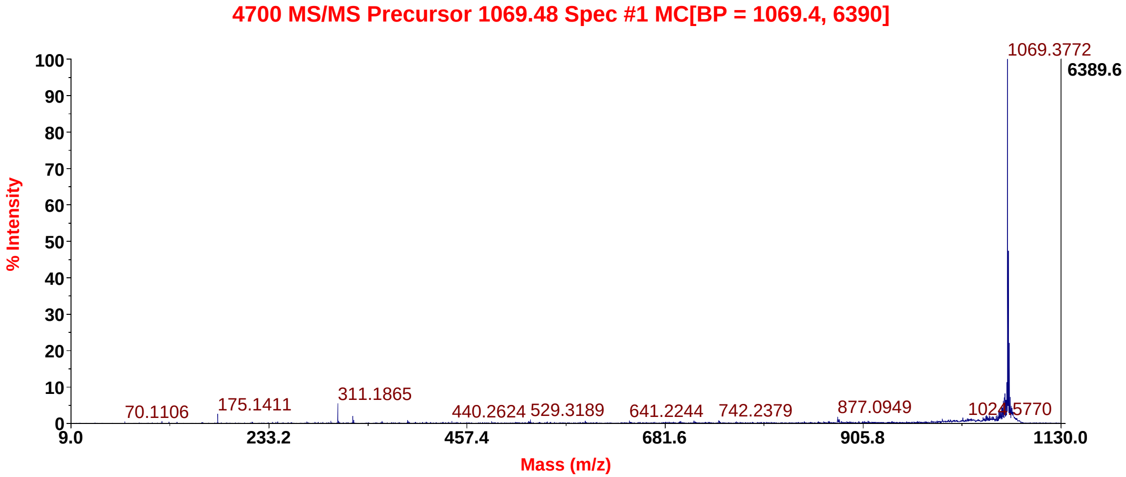


C:\...\F9_MSMS_1069.4824_20.t2d
Acquired:

F9_MSMS_1

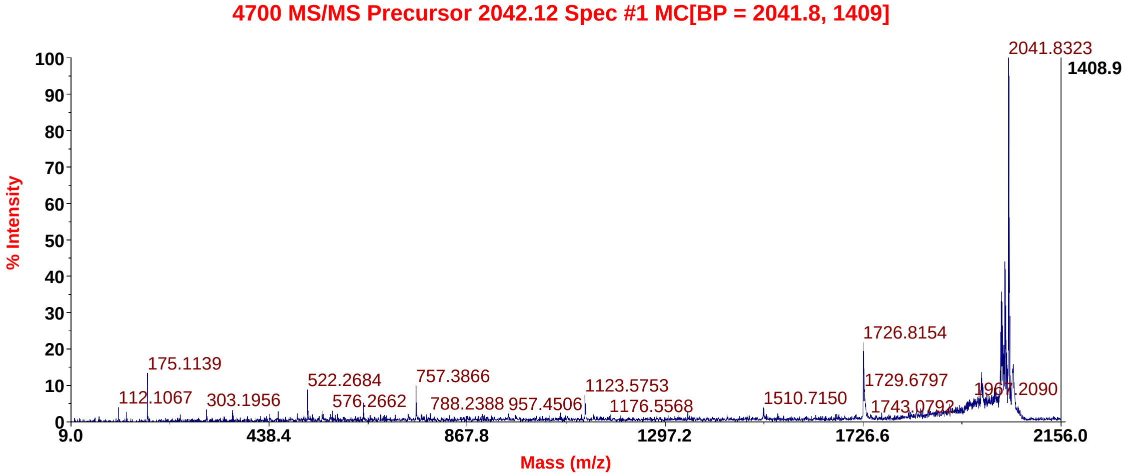


C:\...\F9_MSMS_2042.1218_17.t2d
Acquired:

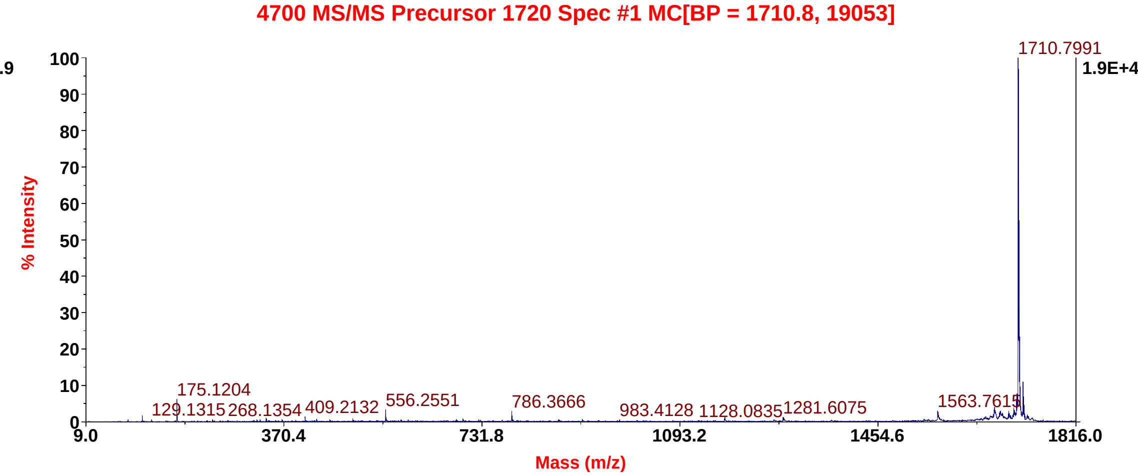


C:\...\F9_MSMS_1720.0018_18.t2d
Acquired:

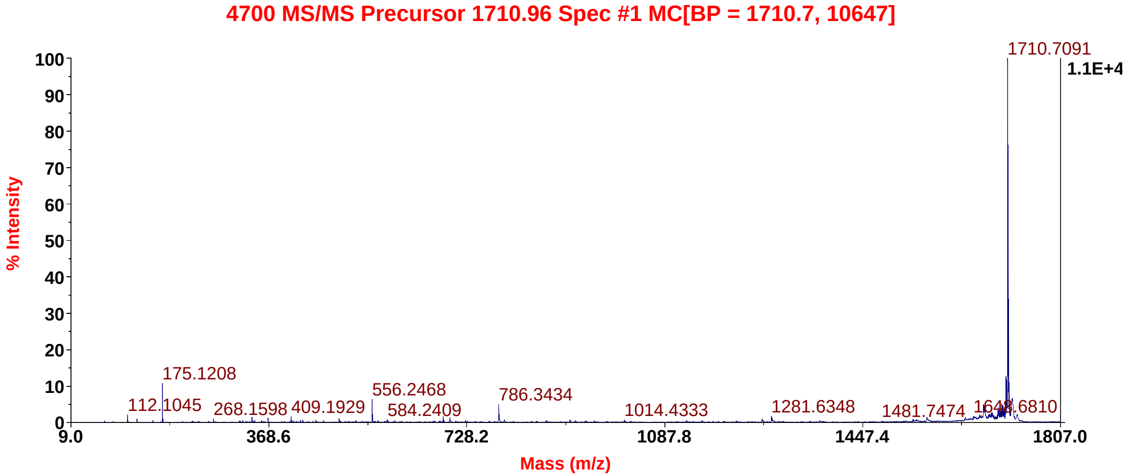


C:\...\F9_MSMS_1710.9609_13.t2d
Acquired:

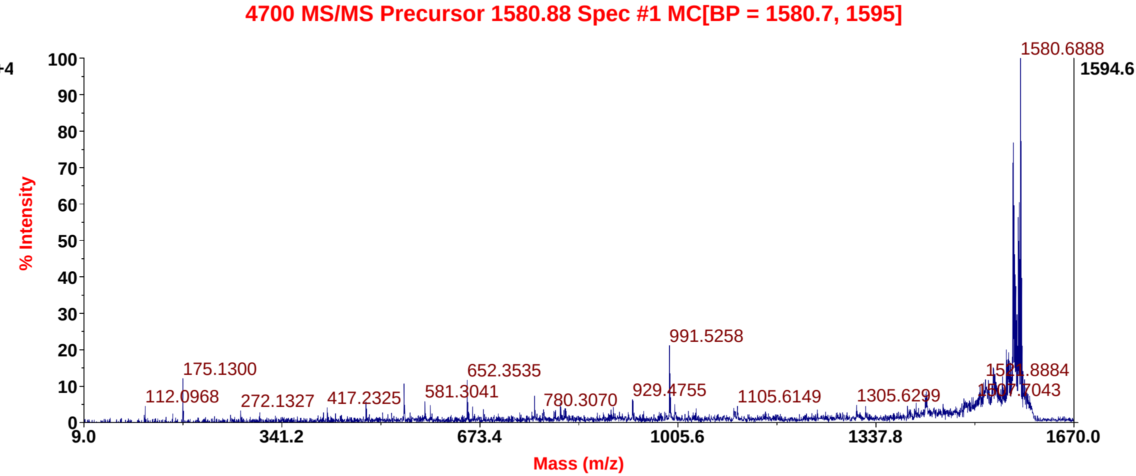


C:\...\F9_MSMS_1580.8771_19.t2d
Acquired:

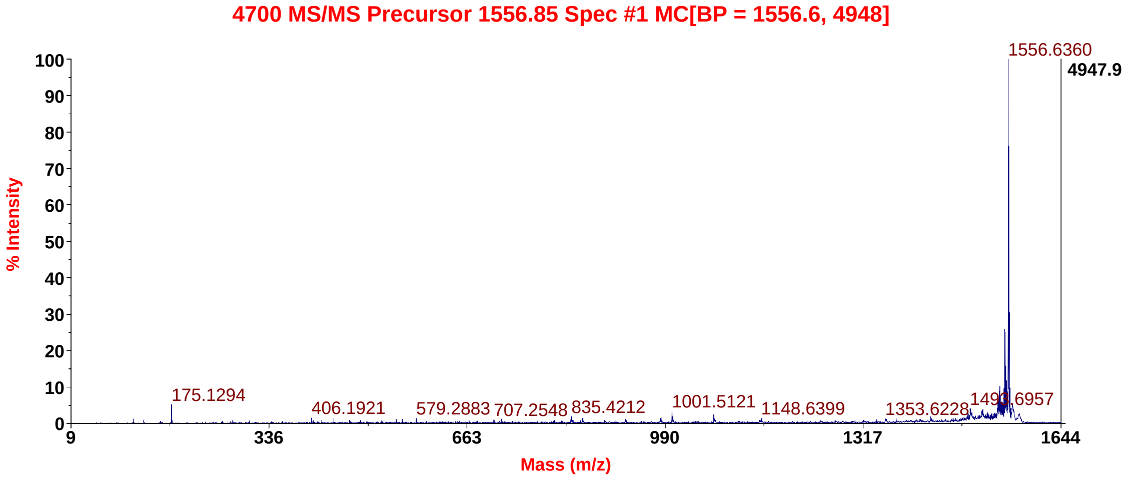


C:\...\F9_MSMS_1556.8470_15.t2d
Acquired:

# F9_MSMS_2

4700 MS/MS Precursor 1410.75 Spec #1 MC[BP = 1410.6, 10826]

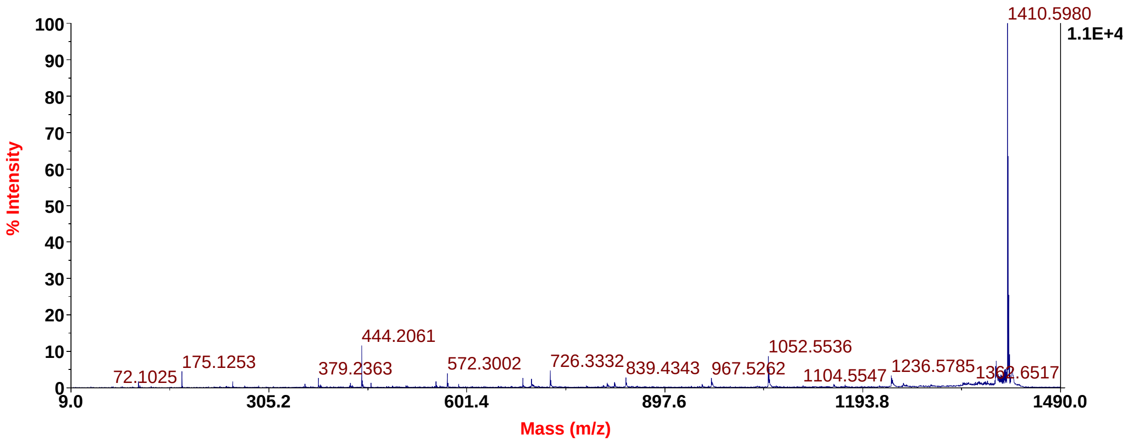


C:\...\F19_MSMS_1410.7474_20.t2d
Acquired:

4700 MS/MS Precursor 1295.67 Spec #1 MC[BP = 1295.6, 21711]

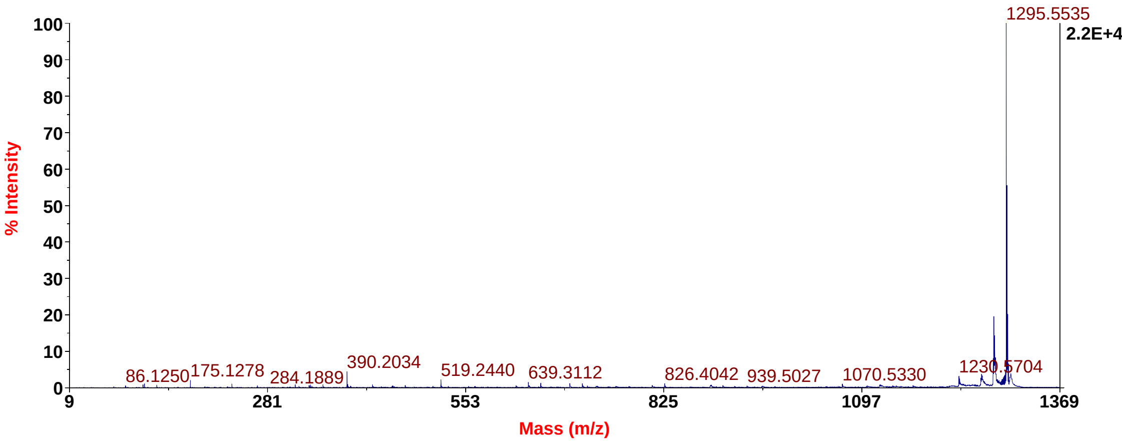


C:\...\F19_MSMS_1295.6746_16.t2d
Acquired:

4700 MS/MS Precursor 1278.65 Spec #1 MC[BP = 1278.5, 17116]

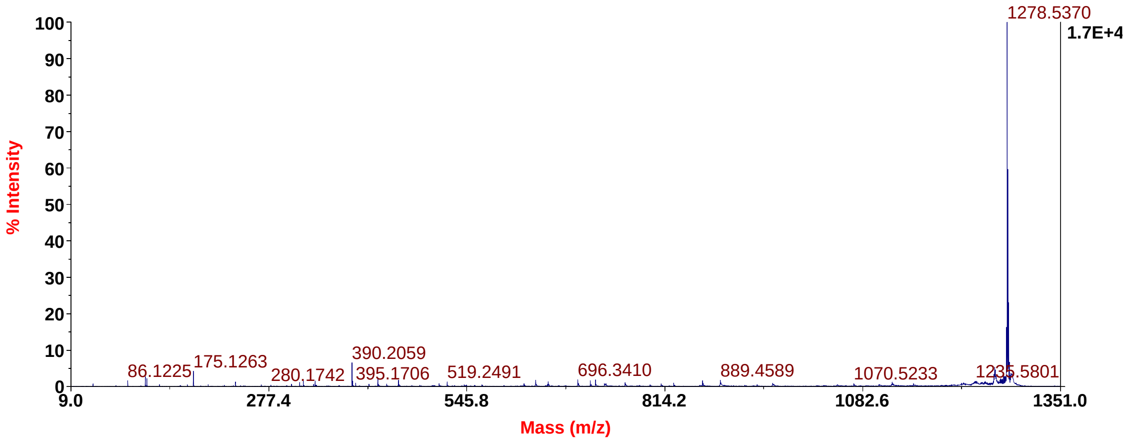


C:\...\F19_MSMS_1278.6504_19.t2d
Acquired:

4700 MS/MS Precursor 1241.71 Spec #1 MC[BP = 1241.6, 6227]

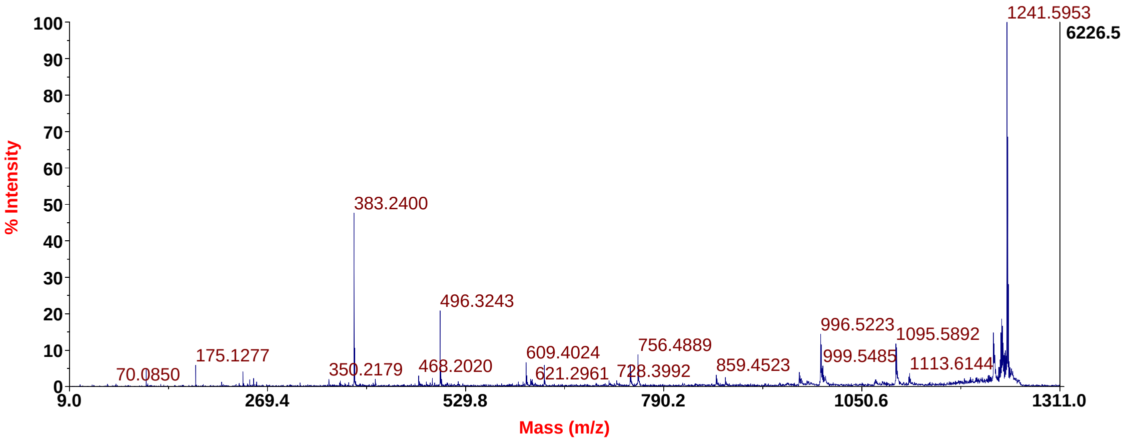


C:\...\F19_MSMS_1241.7062_17.t2d
Acquired:

4700 MS/MS Precursor 923.547 Spec #1 MC[BP = 923.5, 26938]

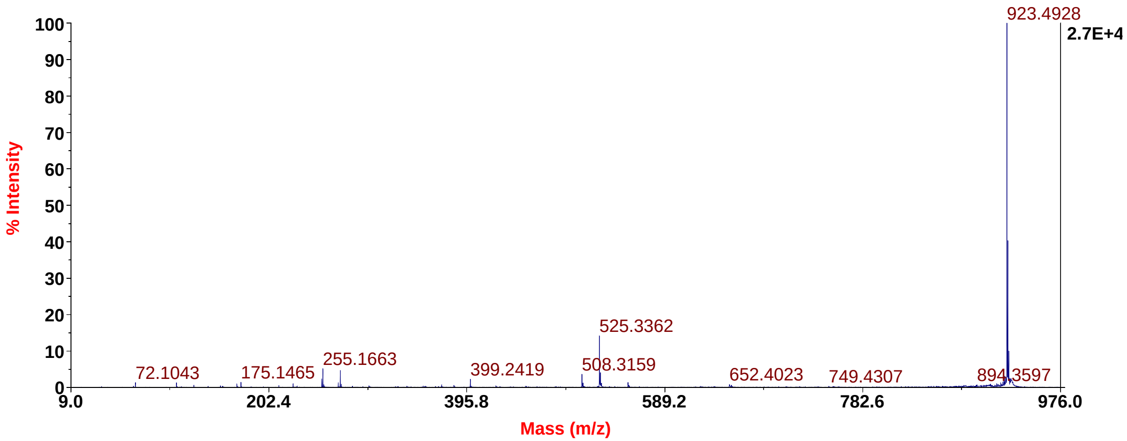


C:\...\F19_MSMS_923.5469_18.t2d
Acquired:

F19_MSMS_1

4700 MS/MS Precursor 2725.32 Spec #1 MC[BP = 619.2, 9937]

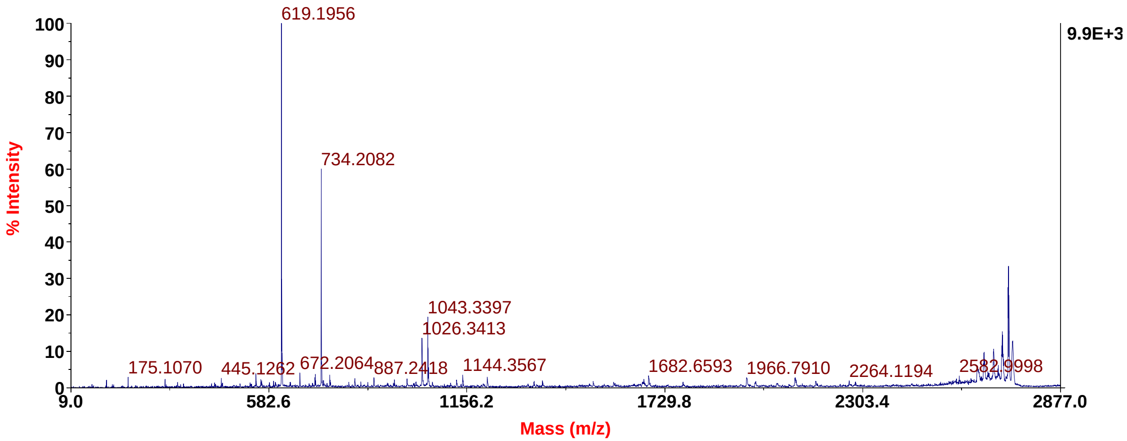


C:\...\F19_MSMS_2725.3242_14.t2d
Acquired:

4700 MS/MS Precursor 2653.43 Spec #1 MC[BP = 2654.1, 6361]

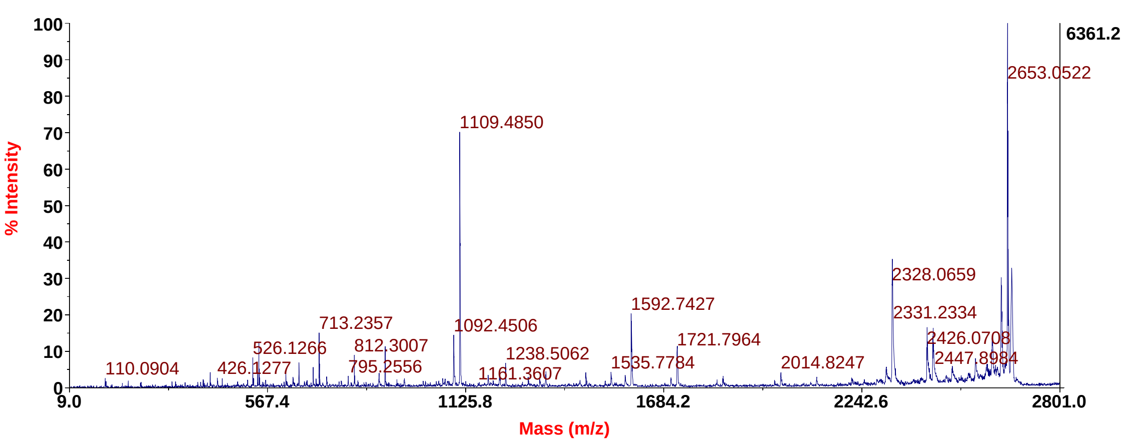


C:\...\F19_MSMS_2653.4299_13.t2d
Acquired:

4700 MS/MS Precursor 2051.09 Spec #1 MC[BP = 2050.8, 1811]

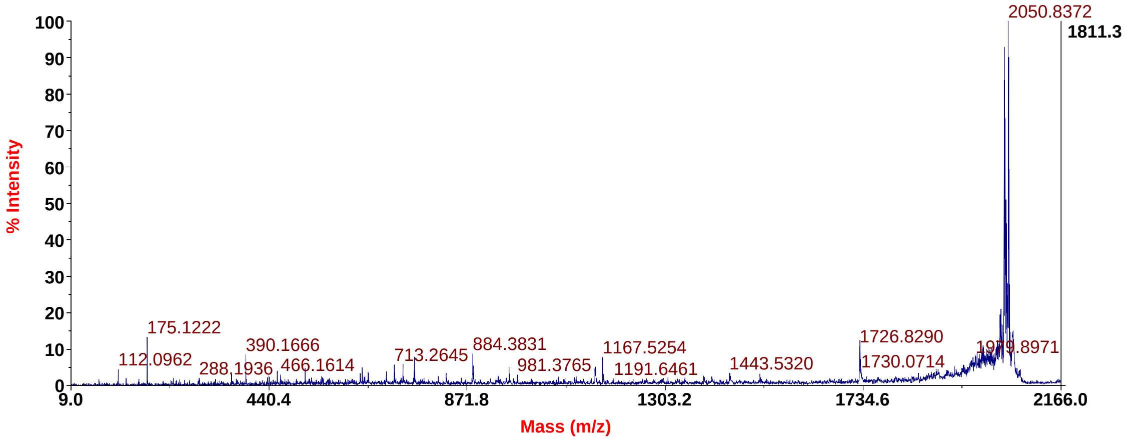


C:\...\F19_MSMS_2051.0906_22.t2d
Acquired:

4700 MS/MS Precursor 1649.84 Spec #1 MC[BP = 1649.7, 18699]

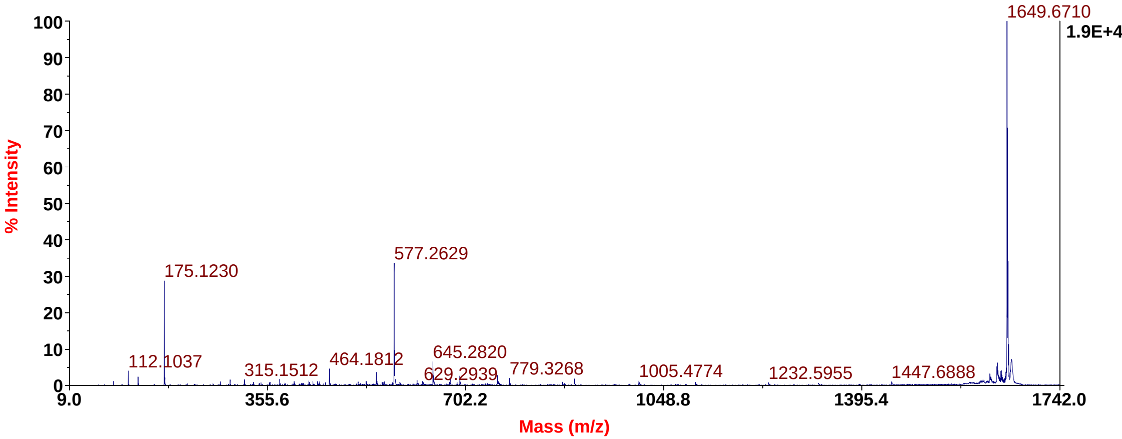


C:\...\F19_MSMS_1649.8361_15.t2d
Acquired:

4700 MS/MS Precursor 1440.82 Spec #1 MC[BP = 1440.7, 3442]

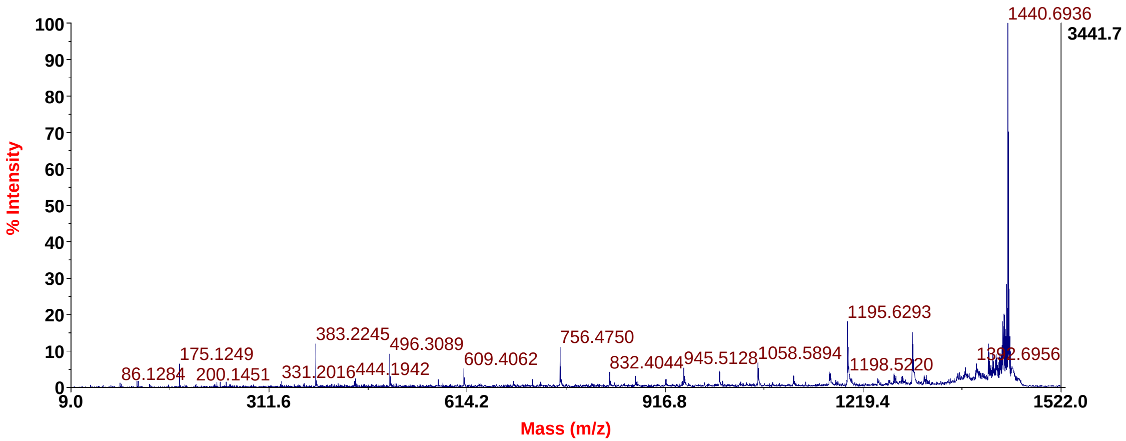


C:\...\F19_MSMS_1440.8157_21.t2d
Acquired:

F19_MSMS_2

4700 MS/MS Precursor 1556.85 Spec #1 MC[BP = 1556.6, 13850]

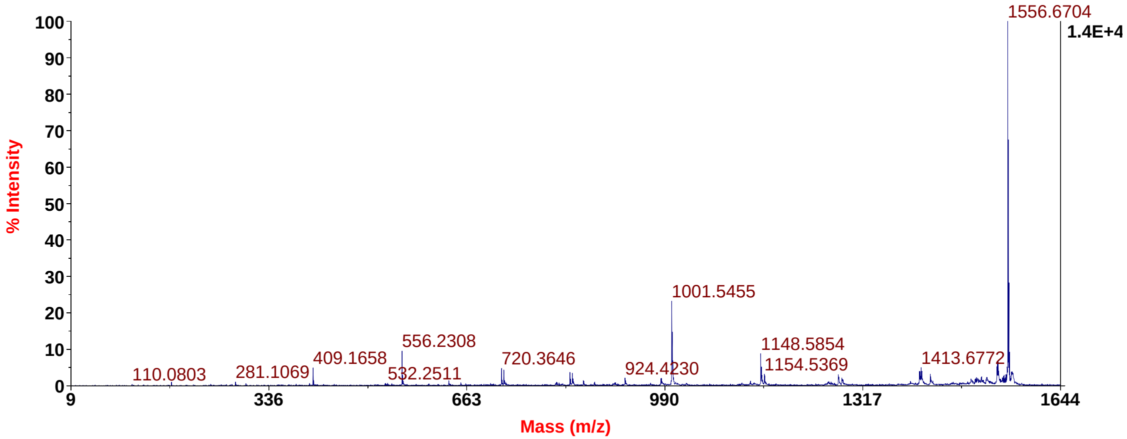


C:\...\F21_MSMS_1556.8529_15.t2d
Acquired:

4700 MS/MS Precursor 1506.78 Spec #1 MC[BP = 1502.7, 14749]

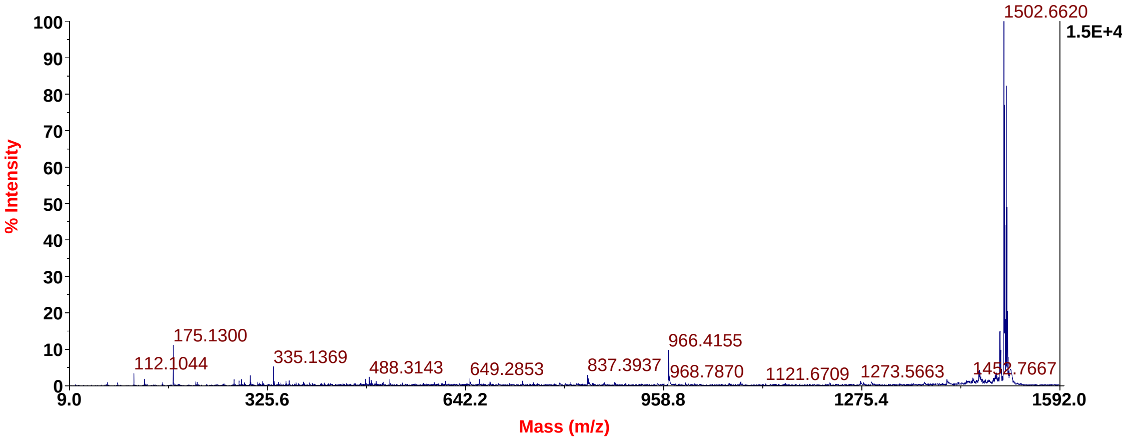


C:\...\F21_MSMS_1506.7789_16.t2d
Acquired:

4700 MS/MS Precursor 1428.74 Spec #1 MC[BP = 1428.6, 4620]

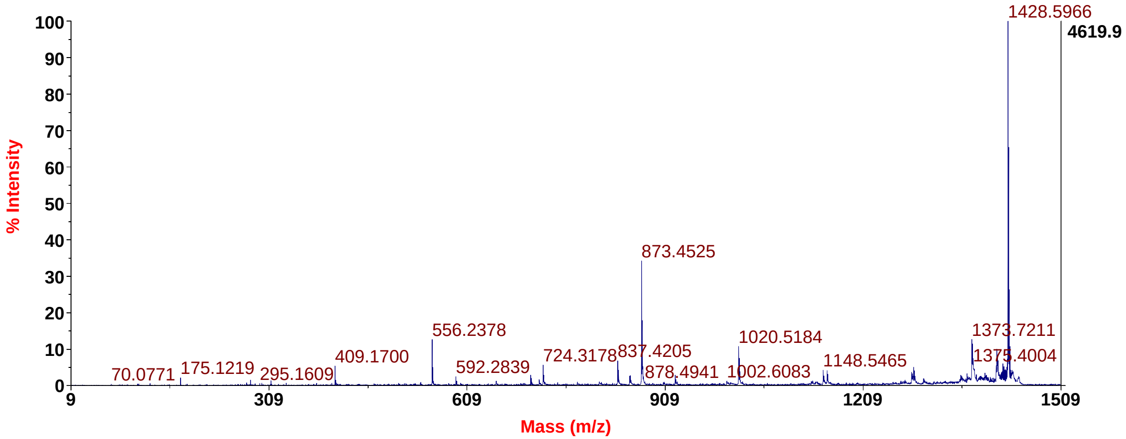


C:\...\F21_MSMS_1428.7402_19.t2d
Acquired:

4700 MS/MS Precursor 1373.7 Spec #1 MC[BP = 1373.6, 31255]

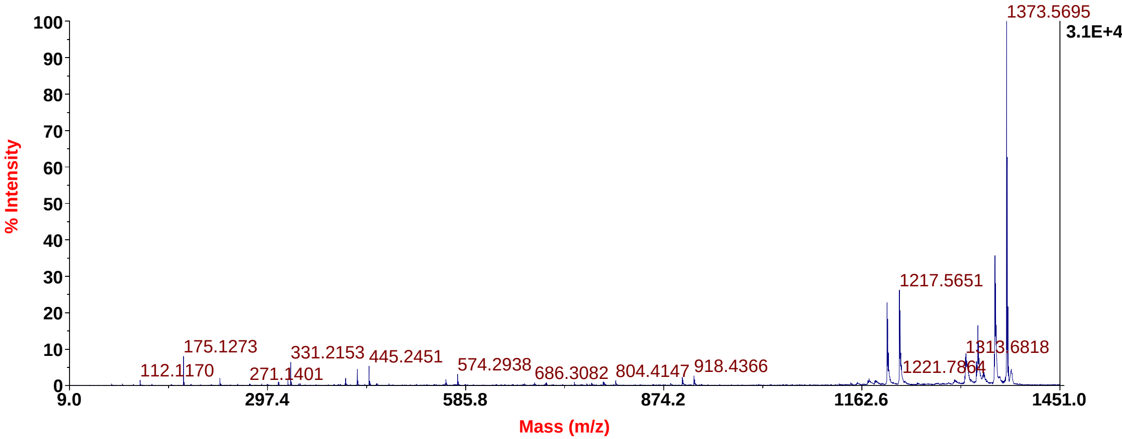


C:\...\F21_MSMS_1373.7042_13.t2d
Acquired:

4700 MS/MS Precursor 1208.63 Spec #1 MC[BP = 1208.5, 3079]

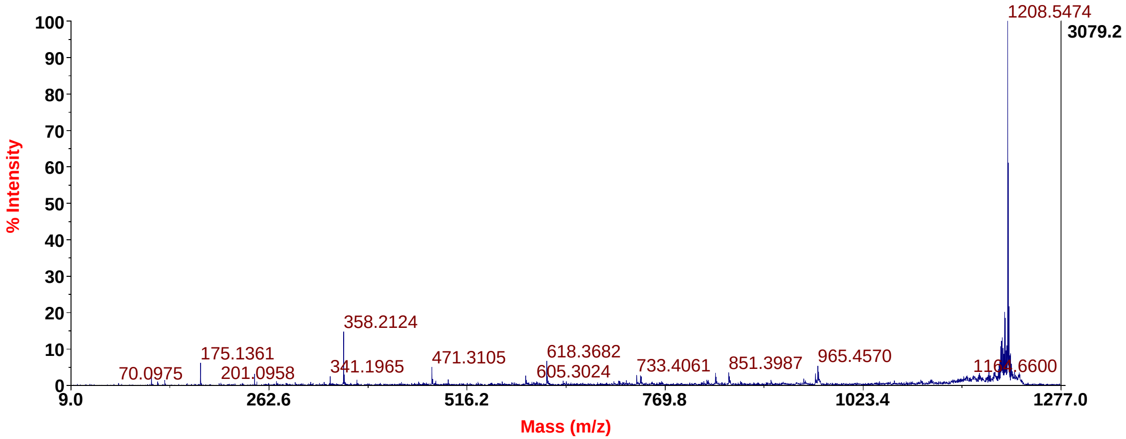


C:\...\F21_MSMS_1208.6283_22.t2d
Acquired:

F21_MSMS_1

4700 MS/MS Precursor 3176.83 Spec #1 MC[BP = 840.3, 6230]

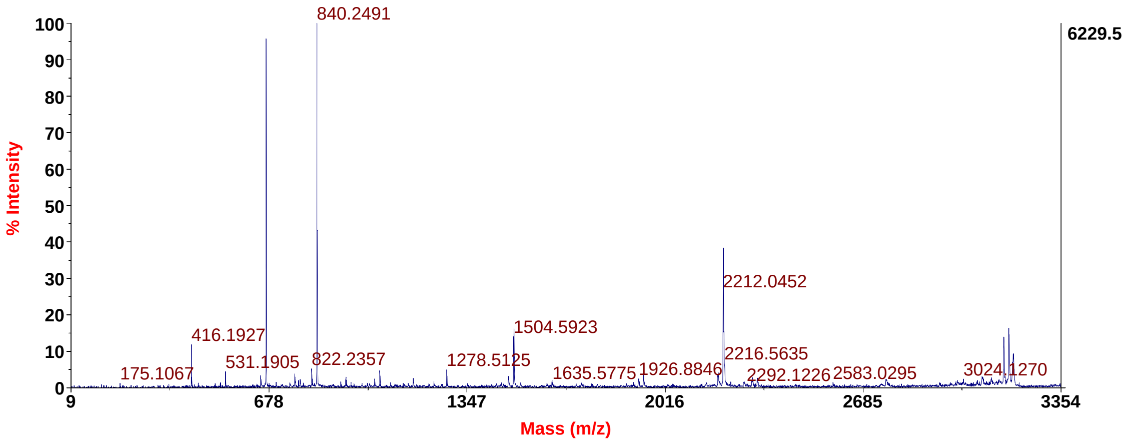


C:\...\F21_MSMS_3176.8335_18.t2d
Acquired:

4700 MS/MS Precursor 2193.17 Spec #1 MC[BP = 561.2, 6401]

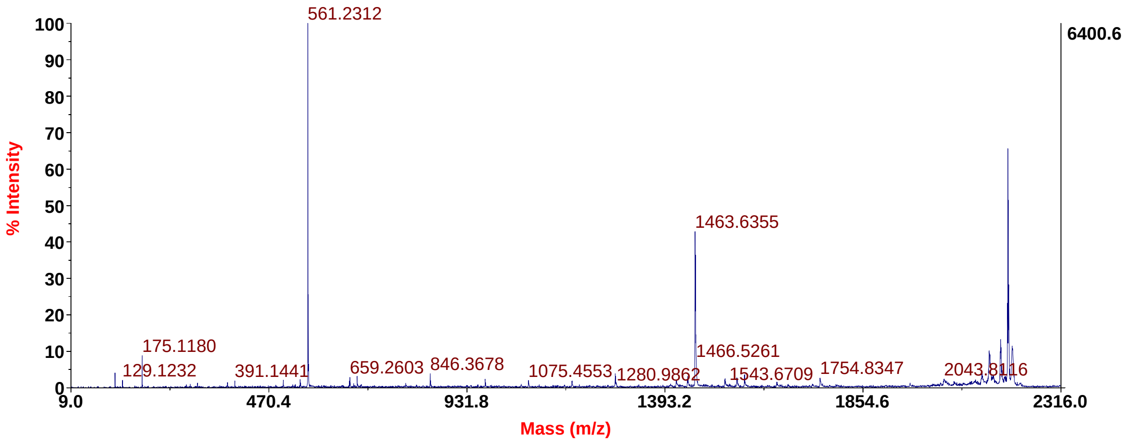


C:\...\F21_MSMS_2193.1746_17.t2d
Acquired:

4700 MS/MS Precursor 2042.17 Spec #1 MC[BP = 2041.8, 11031]

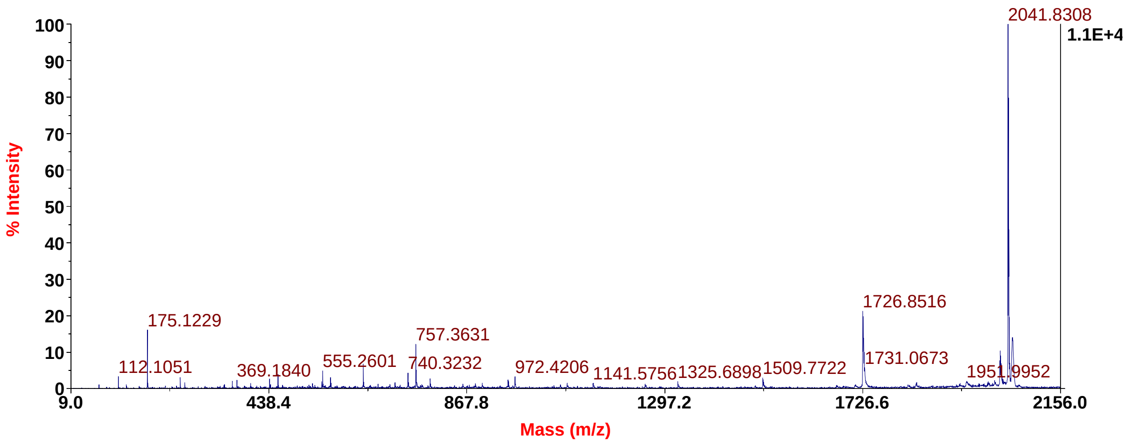


C:\...\F21_MSMS_2042.1663_14.t2d
Acquired:

4700 MS/MS Precursor 1650.91 Spec #1 MC[BP = 1650.7, 4125]

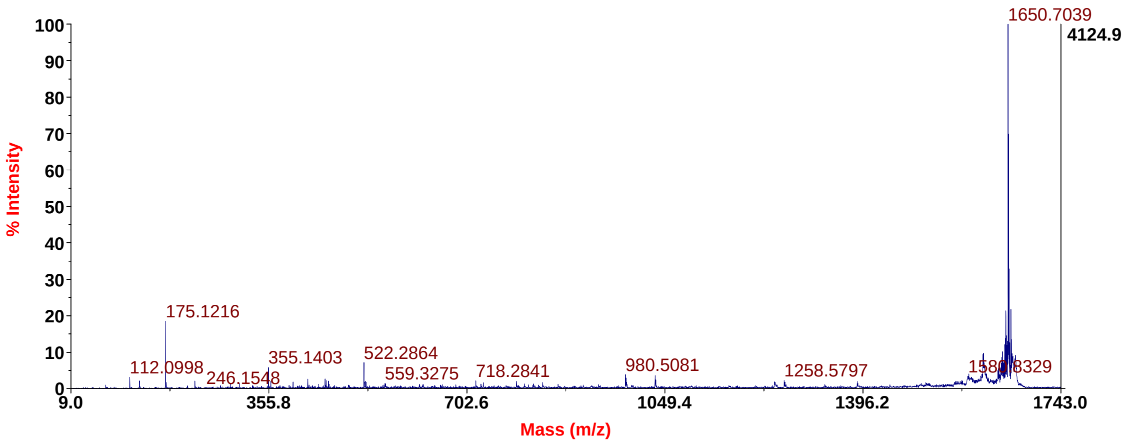


C:\...\F21_MSMS_1650.9136_21.t2d
Acquired:

4700 MS/MS Precursor 1580.87 Spec #1 MC[BP = 1580.7, 3582]

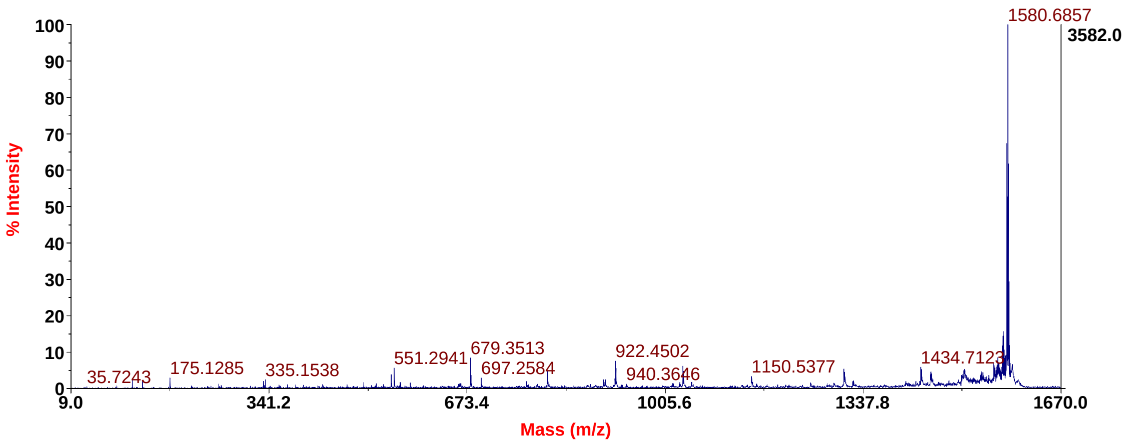


C:\...\F21_MSMS_1580.8727_20.t2d
Acquired:

F21_MSMS_2

4700 MS/MS Precursor 1650.87 Spec #1 MC[BP = 1650.7, 22049]

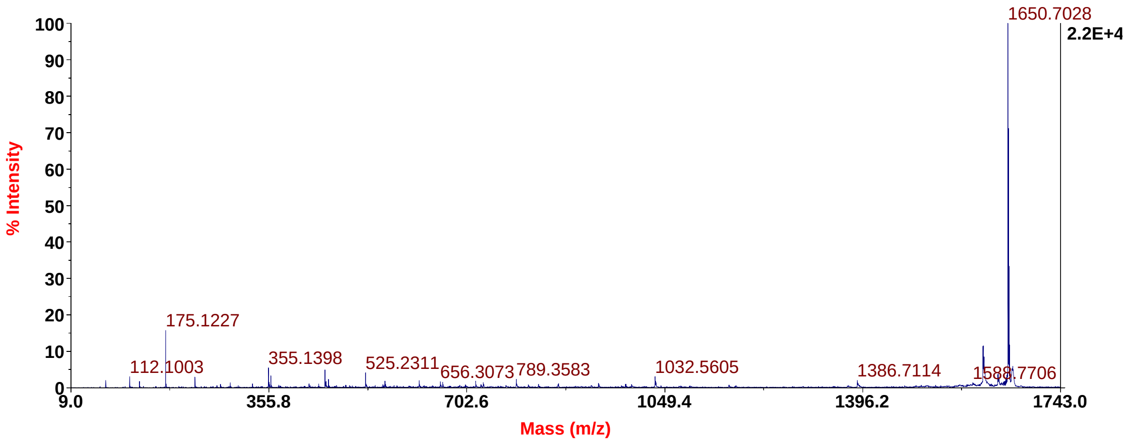


C:\...\F23_MSMS_1650.8668_15.t2d
Acquired:

4700 MS/MS Precursor 1556.81 Spec #1 MC[BP = 1556.7, 7054]

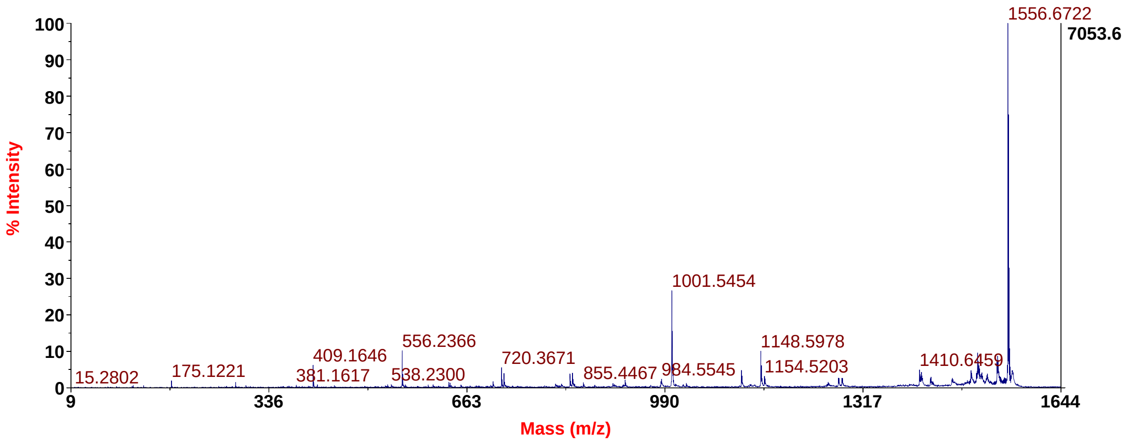


C:\...\F23_MSMS_1556.8064_20.t2d
Acquired:

4700 MS/MS Precursor 1506.73 Spec #1 MC[BP = 1506.6, 15826]

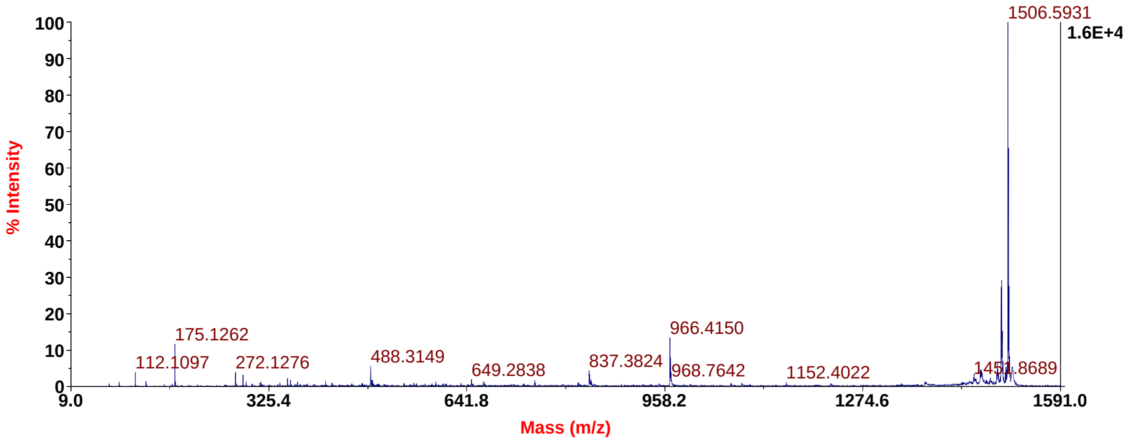


C:\...\F23_MSMS_1506.7313_19.t2d
Acquired:

4700 MS/MS Precursor 1428.7 Spec #1 MC[BP = 1428.6, 8697]

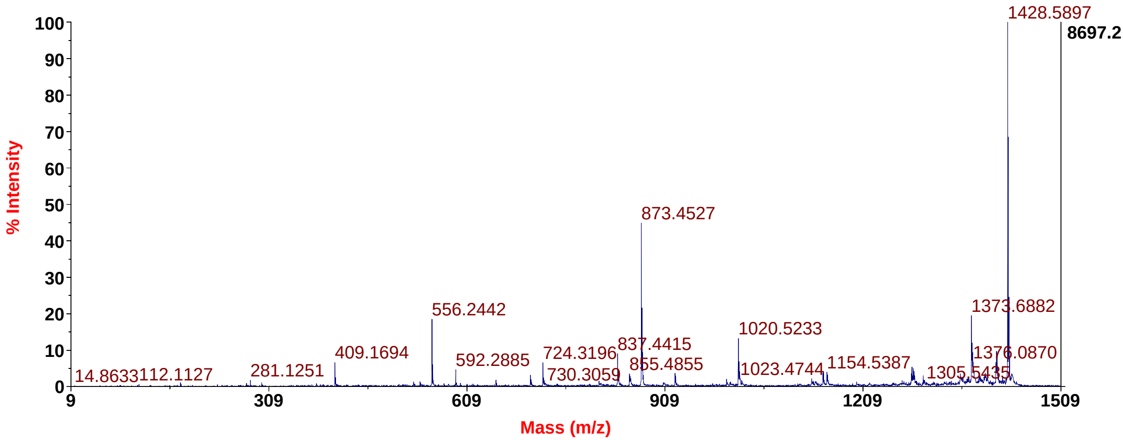


C:\...\F23_MSMS_1428.6981_17.t2d
Acquired:

4700 MS/MS Precursor 1373.67 Spec #1 MC[BP = 1373.6, 24809]

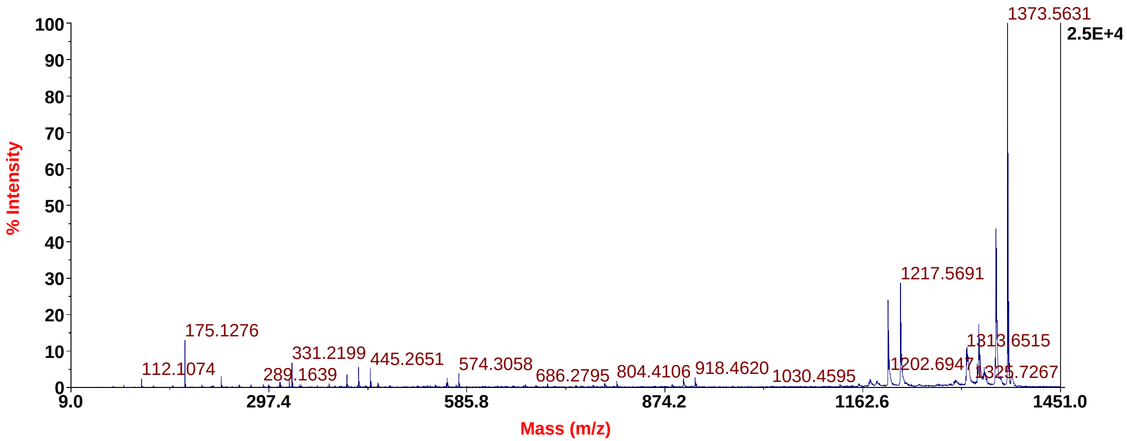


C:\...\F23_MSMS_1373.6707_16.t2d
Acquired:

F23_MSMS_1

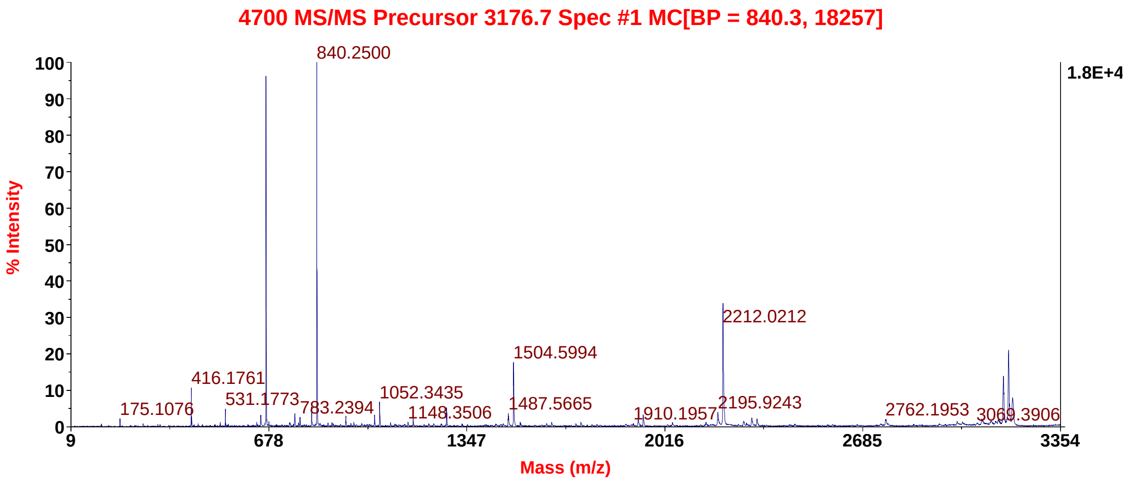


C:\...\F23_MSMS_3176.7026_14.t2d
Acquired:

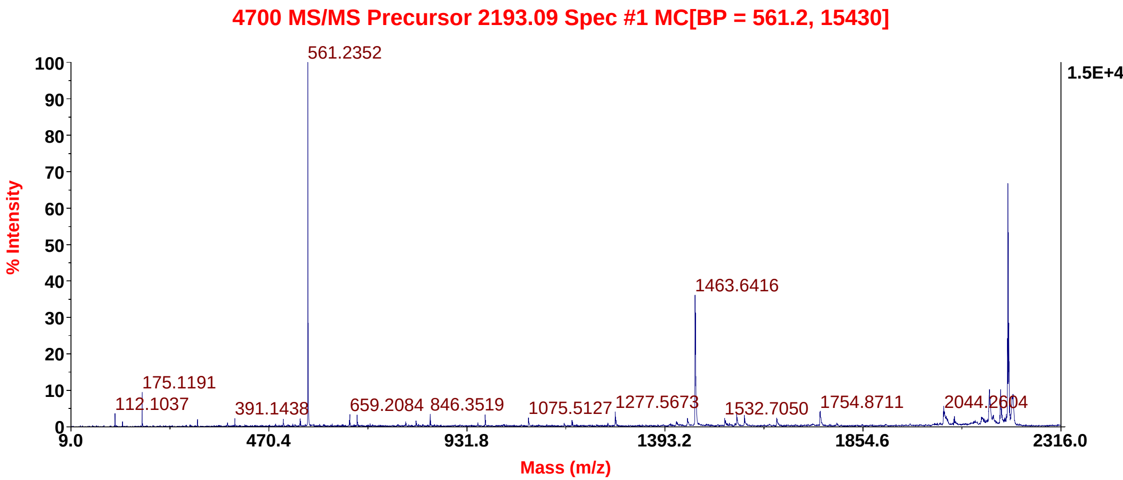


C:\...\F23_MSMS_2193.0864_22.t2d
Acquired:

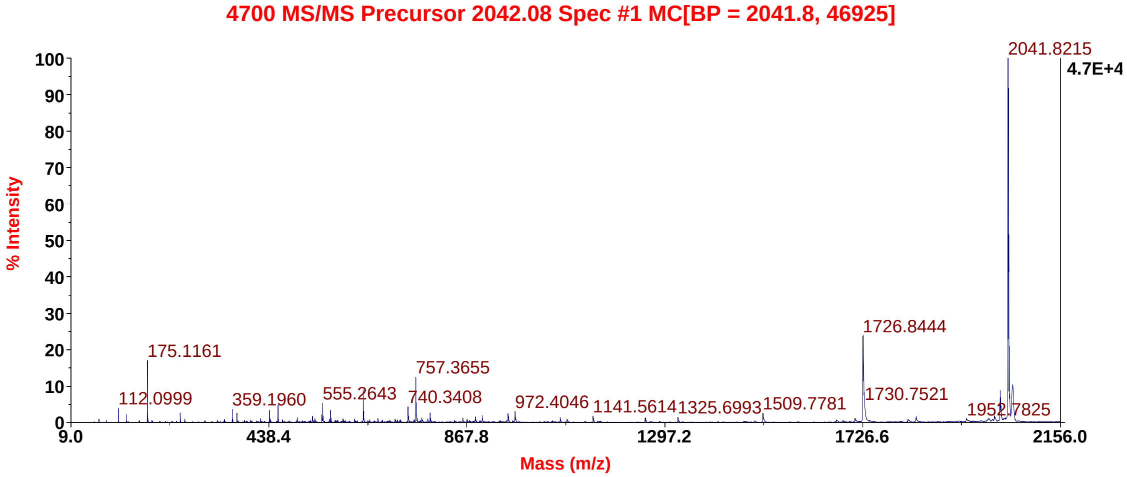


C:\...\F23_MSMS_2042.0836_13.t2d
Acquired:

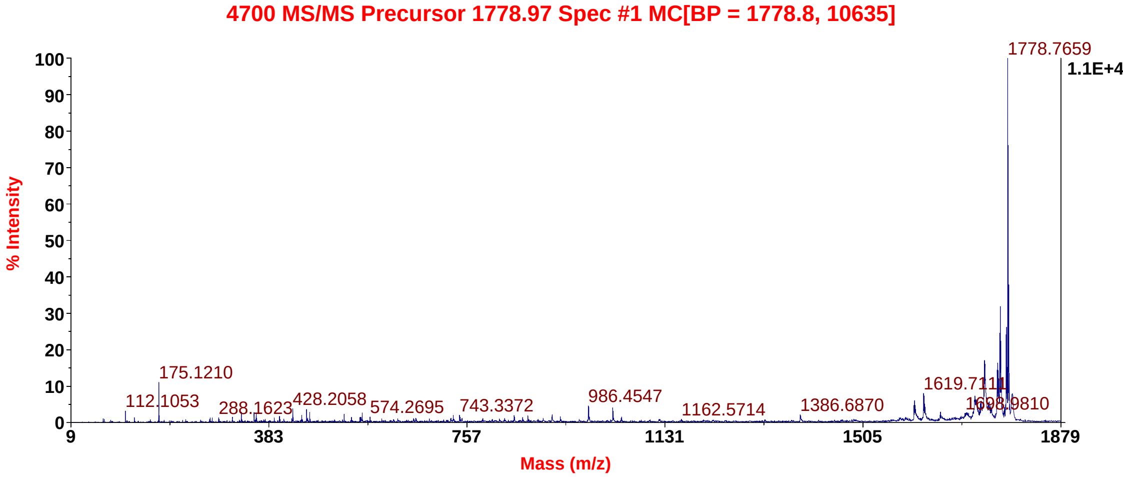


C:\...\F23_MSMS_1778.9694_18.t2d
Acquired:

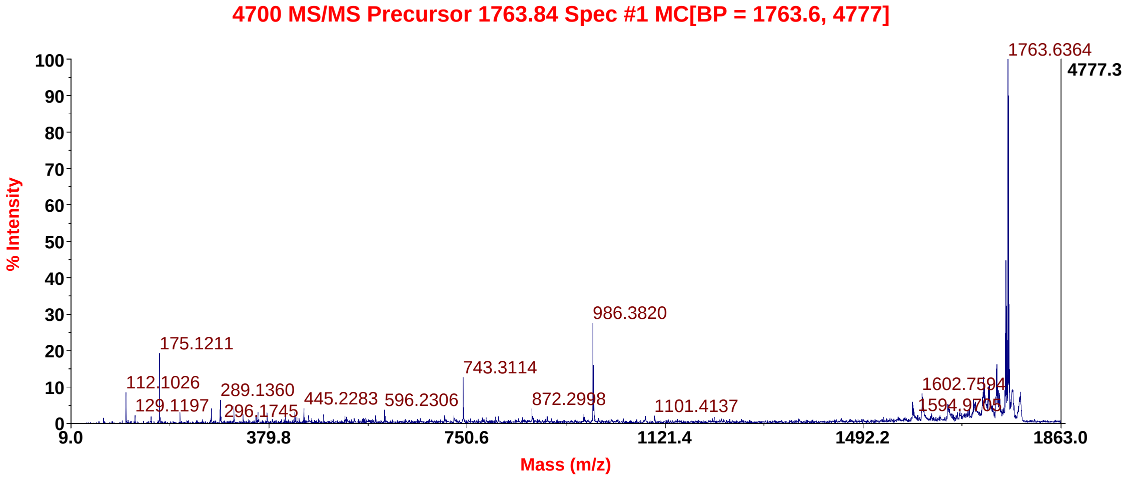


C:\...\F23_MSMS_1763.8428_21.t2d
Acquired:

F23_MSMS_2

4700 MS/MS Precursor 1556.84 Spec #1 MC[BP = 1556.6, 1104]

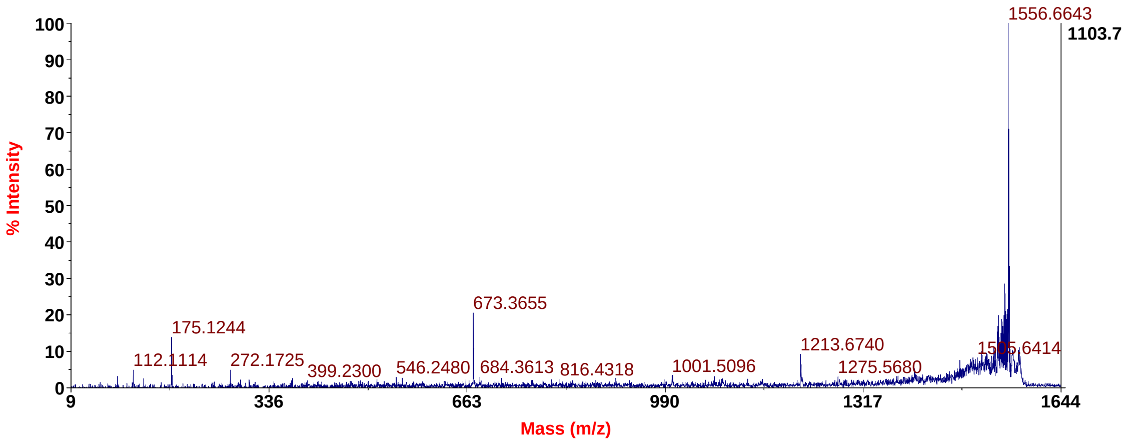


C:\...\G10_MSMS_1556.8444_22.t2d
Acquired:

4700 MS/MS Precursor 1420.77 Spec #1 MC[BP = 1420.6, 9746]

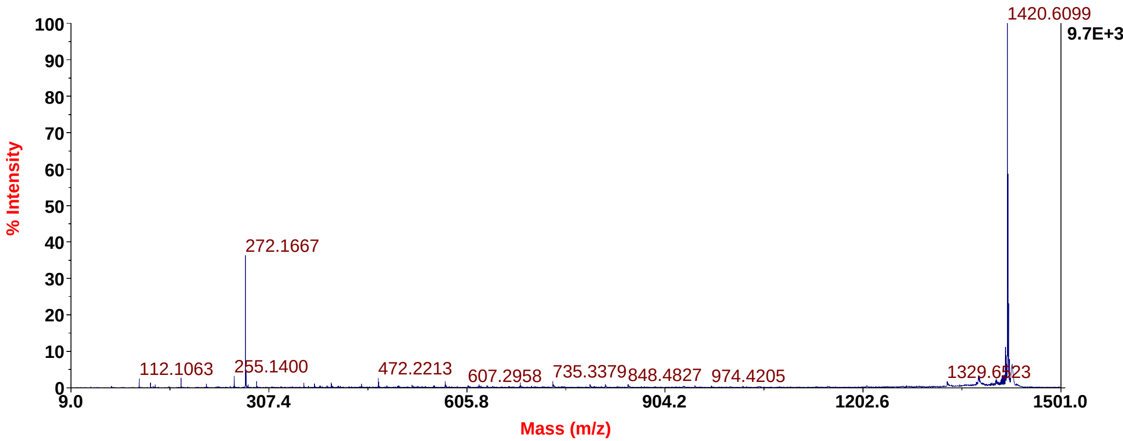


C:\...\G10_MSMS_1420.7726_13.t2d
Acquired:

4700 MS/MS Precursor 1236.57 Spec #1 MC[BP = 1236.4, 3245]

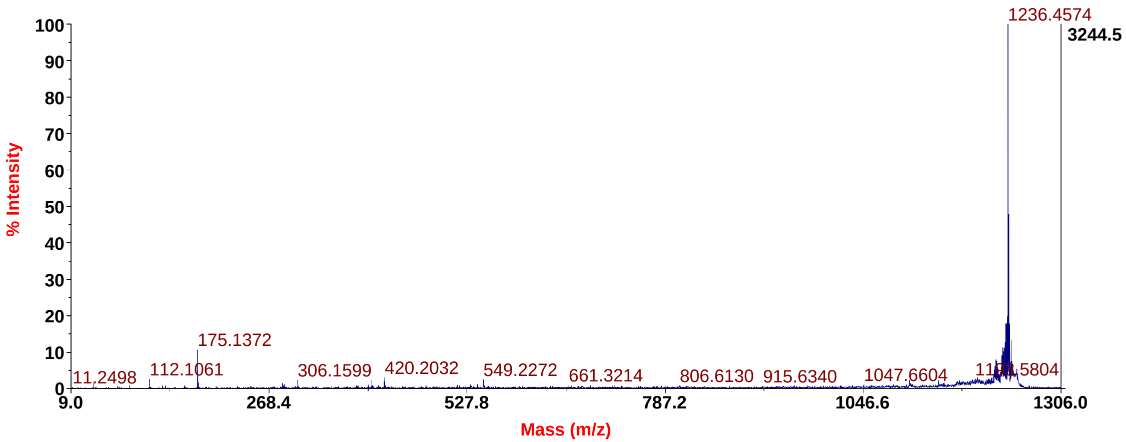


C:\...\G10_MSMS_1236.5706_20.t2d
Acquired:

4700 MS/MS Precursor 1069.47 Spec #1 MC[BP = 1069.4, 5204]

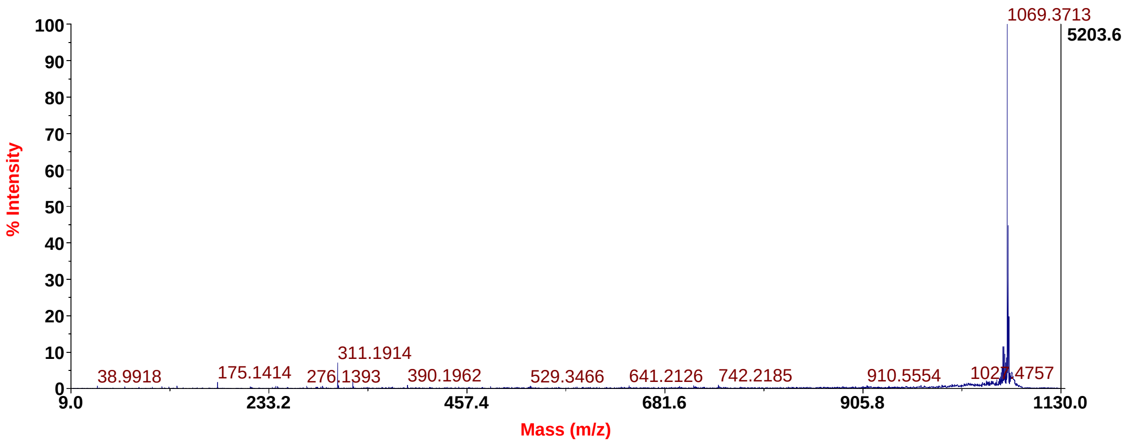


C:\...\G10_MSMS_1069.4663_16.t2d
Acquired:

4700 MS/MS Precursor 870.548 Spec #1 MC[BP = 870.5, 10217]

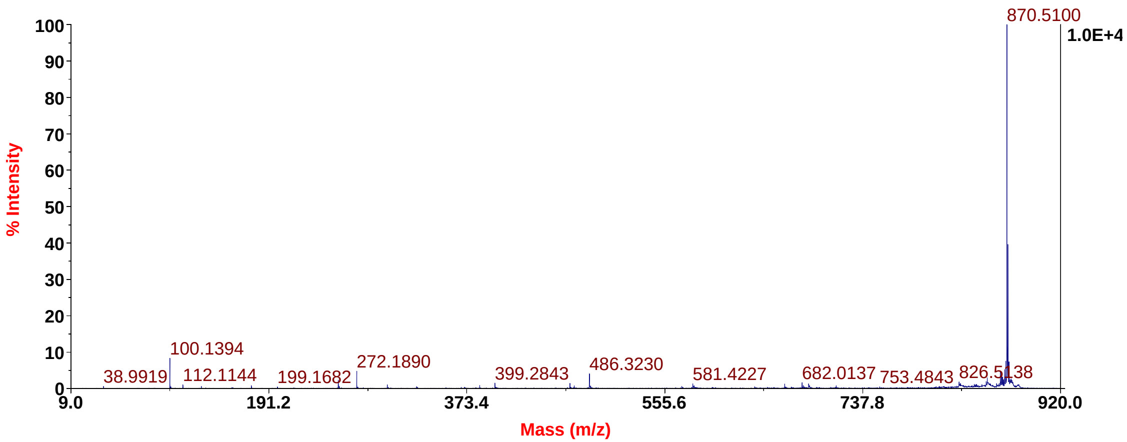


C:\...\G10_MSMS_870.5482_19.t2d
Acquired:

G10_MSMS_1

4700 MS/MS Precursor 2173.17 Spec #1 MC[BP = 2156.0, 1307]

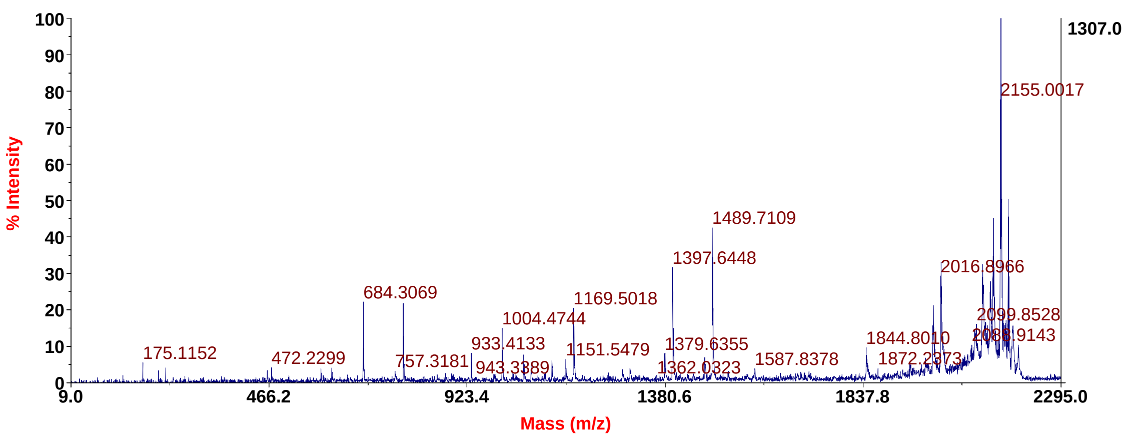


C:\...\G10_MSMS_2173.1741_17.t2d
Acquired:

4700 MS/MS Precursor 2014.05 Spec #1 MC[BP = 648.3, 760]

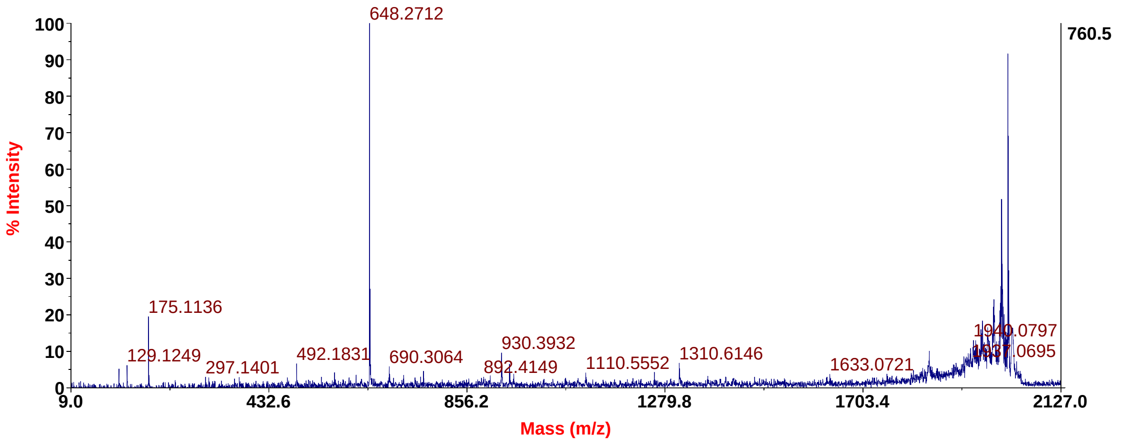


C:\...\G10_MSMS_2014.0463_18.t2d
Acquired:

4700 MS/MS Precursor 1783.99 Spec #1 MC[BP = 1773.8, 4617]

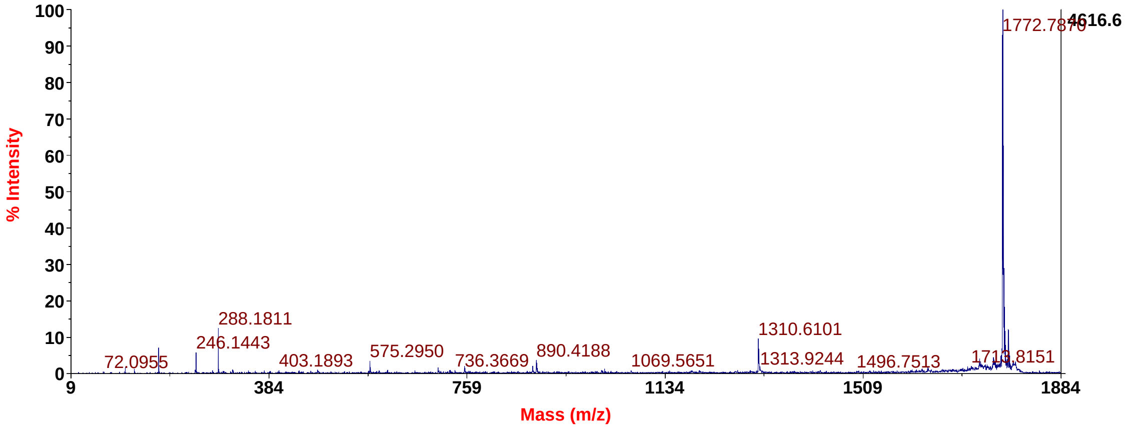


C:\...\G10_MSMS_1783.9897_21.t2d
Acquired:

4700 MS/MS Precursor 1772.92 Spec #1 MC[BP = 1772.7, 3539]

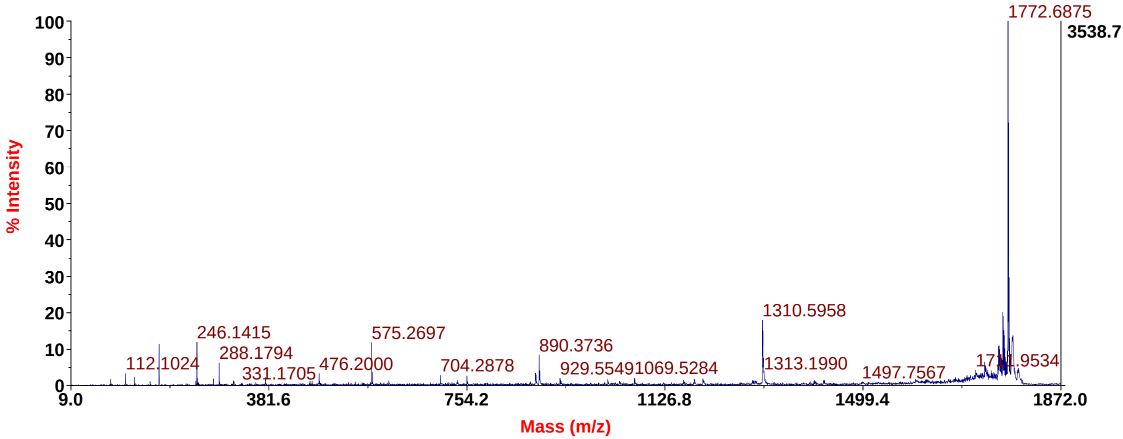


C:\...\G10_MSMS_1772.9185_14.t2d
Acquired:

4700 MS/MS Precursor 1576.86 Spec #1 MC[BP = 1576.7, 2573]

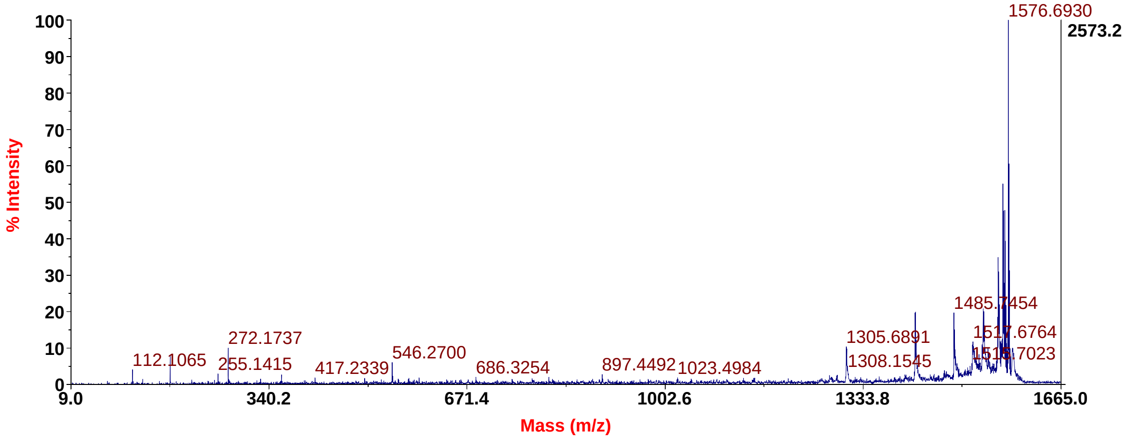


C:\...\G10_MSMS_1576.8636_15.t2d
Acquired:

G10_MSMS_2

Supplementary material 3：Peptides for MS/MS identification

| Spot No. | Accession No. | Protein Name | | | | | | Protein PI | Protein MW | Protein Score | Protein Score / C. I. % | Total Ion Score | Total Ion / C. I. % | Peptide number |
|---|---|---|---|---|---|---|---|---|---|---|---|---|---|---|
| 597 | gi\|29335504 | keratin 18 | | | | | | 5.07 | 41247.4 | 499 | 100 | 322 | 100 | 23 |
| | Peptide Information | | | | | | | | | | | | | |
| | Calc. Mass | Obsrv. Mass | ± da | ± ppm | Start Seq. | End Seq. | Sequence | | | | Ion Score / C. I. % | Modification | | Result Type |
| | 807.3995 | 807.4735 | 0.074 | 92 | 91 | 97 | LAADDFR | | | | | | | Mascot |
| | 951.4993 | 951.5565 | 0.0572 | 60 | 347 | 355 | VVTSSTETK | | | | | | | Mascot |
| | 996.4996 | 996.5766 | 0.077 | 77 | 100 | 107 | YESELSIR | | | | | | | Mascot |
| | 1020.5472 | 1020.6179 | 0.0707 | 69 | 314 | 322 | RLLDGGDFK | | | | | | | Mascot |
| | 1041.6051 | 1041.6664 | 0.0613 | 59 | 82 | 90 | LVLQIDNAR | | | | | | | Mascot |
| | 1041.6051 | 1041.6664 | 0.0613 | 59 | 82 | 90 | LVLQIDNAR | | | 77 | 99.999 | | | Mascot |
| | 1059.543 | 1059.6146 | 0.0716 | 68 | 120 | 128 | VIDDTNLNR | | | | | | | Mascot |
| | 1065.5575 | 1065.6189 | 0.0614 | 58 | 305 | 313 | LEAEIATYR | | | | | | | Mascot |
| | 1092.4924 | 1092.568 | 0.0756 | 69 | 14 | 22 | MAMQNLNDR | | | | | | | Mascot |
| | 1116.6049 | 1116.6627 | 0.0578 | 52 | 61 | 69 | FQPIVDELR | | | | | | | Mascot |
| | 1116.6049 | 1116.6627 | 0.0578 | 52 | 61 | 69 | FQPIVDELR | | | 65 | 99.981 | | | Mascot |
| | 1122.5538 | 1122.6207 | 0.0669 | 60 | 72 | 81 | IFDATTNNAR | | | | | | | Mascot |
| | 1122.5538 | 1122.6207 | 0.0669 | 60 | 72 | 81 | IFDATTNNAR | | | 36 | 85.027 | | | Mascot |
| | 1187.6743 | 1187.6842 | 0.0099 | 8 | 247 | 257 | NLKGSLEGTLR | | | | | | | Mascot |
| | 1201.5709 | 1201.6477 | 0.0768 | 64 | 51 | 60 | RGPDVHDYSR | | | 57 | 99.891 | | | Mascot |
| | 1201.6423 | 1201.6477 | 0.0054 | 4 | 323 | 332 | LQDALEEQKK | | | | | | | Mascot |
| | 1221.6586 | 1221.718 | 0.0594 | 49 | 305 | 314 | LEAEIATYRR | | | | | | | Mascot |
| | 1223.6631 | 1223.7274 | 0.0643 | 53 | 98 | 107 | VKYESELSIR | | | | | | | Mascot |
| | 1223.6631 | 1223.7274 | 0.0643 | 53 | 98 | 107 | VKYESELSIR | | | 60 | 99.936 | | | Mascot |
| | 1244.6998 | 1244.7698 | 0.07 | 56 | 61 | 70 | FQPIVDELRK | | | | | | | Mascot |
| | 1244.6998 | 1244.7698 | 0.07 | 56 | 61 | 70 | FQPIVDELRK | | | 29 | 27.336 | | | Mascot |
| | 1250.6488 | 1250.7146 | 0.0658 | 53 | 71 | 81 | KIFDATTNNAR | | | | | | | Mascot |
| | 1272.5637 | 1272.6333 | 0.0696 | 55 | 148 | 157 | NHDNEVMELR | | | | | Oxidation (M)[7] | | Mascot |
| | 1291.6927 | 1291.735 | 0.0423 | 33 | 335 | 346 | VMTVTQTLVDGK | | | | | | | Mascot |
| | 1324.693 | 1324.7616 | 0.0686 | 52 | 303 | 313 | MKLEAEIATYR | | | | | | | Mascot |
| | 1340.6879 | 1340.7439 | 0.056 | 42 | 303 | 313 | MKLEAEIATYR | | | | | Oxidation (M)[1] | | Mascot |
| | 1384.6638 | 1384.7479 | 0.0841 | 61 | 147 | 157 | KNHDNEVMELR | | | | | | | Mascot |
| | 1480.7061 | 1480.823 | 0.1169 | 79 | 250 | 262 | GSLEGTLRDTEMR | | | | | Oxidation (M)[12] | | Mascot |
| | 1684.8501 | 1684.8691 | 0.019 | 11 | 158 | 173 | NQISQSGVQVDVDAPK | | | | | | | Mascot |
| **Spot No.** | **Accession No.** | **Protein Name** | | | | | | **Protein PI** | **Protein MW** | **Protein Score** | **Protein Score / C. I. %** | **Total Ion Score** | **Total Ion / C. I. %** | **Peptide number** |
| 591 | gi\|30410758 | keratin, type I | | | | | | 5.53 | 48573.1 | 424 | 100 | 287 | 100 | 20 |
| | Peptide Information | | | | | | | | | | | | | |
| | Calc. Mass | Obsrv. Mass | ± da | ± ppm | Start Seq. | End Seq. | Sequence | | | | Ion Score / C. I. % | Modification | | Result Type |
| | 807.3995 | 807.4024 | 0.0029 | 4 | 163 | 169 | LAADDFR | | | | | | | Mascot |

| | | | | | | | | | | | | | | |
|---|---|---|---|---|---|---|---|---|---|---|---|---|---|---|
| | 951.4894 | 951.4828 | -0.0066 | -7 | 26 | 33 | TTNVPTYR | | | | | | | Mascot |
| | 996.4996 | 996.488 | -0.0116 | -12 | 172 | 179 | YESELSIR | | | | | | | Mascot |
| | 1026.5215 | 1026.5236 | 0.0021 | 2 | 48 | 57 | ISSASYSGVR | | | | | | | Mascot |
| | 1041.6051 | 1041.6071 | 0.002 | 2 | 154 | 162 | LVLQIDNAR | | | | | | | Mascot |
| | 1041.6051 | 1041.6071 | 0.002 | 2 | 154 | 162 | LVLQIDNAR | | | 66 | 99.983 | | | |
| | 1059.543 | 1059.538 | -0.005 | -5 | 192 | 200 | VIDDTNLNR | | | | | | | Mascot |
| | 1065.5575 | 1065.5347 | -0.0228 | -21 | 377 | 385 | LEAEIATYR | | | | | | | Mascot |
| | 1107.5906 | 1107.5165 | -0.0741 | -67 | 25 | 33 | RTTNVPTYR | | | | | | | Mascot |
| | 1116.6049 | 1116.6068 | 0.0019 | 2 | 133 | 141 | FQPIVDELR | | | | | | | Mascot |
| | 1116.6049 | 1116.6068 | 0.0019 | 2 | 133 | 141 | FQPIVDELR | | | 46 | 98.46 | | | Mascot |
| | 1122.5538 | 1122.5529 | -0.0009 | -1 | 144 | 153 | IFDATTNNAR | | | | | | | Mascot |
| | 1201.5709 | 1201.6033 | 0.0324 | 27 | 123 | 132 | RGPDVHDYSR | | | | | | | Mascot |
| | 1223.6631 | 1223.6694 | 0.0063 | 5 | 170 | 179 | VKYESELSIR | | | | | | | Mascot |
| | 1223.6631 | 1223.6694 | 0.0063 | 5 | 170 | 179 | VKYESELSIR | | | 56 | 99.823 | | | Mascot |
| | 1244.6998 | 1244.697 | -0.0028 | -2 | 133 | 142 | FQPIVDELRK | | | | | | | Mascot |
| | 1250.6488 | 1250.6443 | -0.0045 | -4 | 143 | 153 | KIFDATTNNAR | | | | | | | Mascot |
| | 1256.5688 | 1256.6512 | 0.0824 | 66 | 220 | 229 | NHDNEVMELR | | | | | | | Mascot |
| | 1256.5688 | 1256.6512 | 0.0824 | 66 | 220 | 229 | NHDNEVMELR | | | 29 | 26.953 | | | Mascot |
| | 1265.6233 | 1265.6366 | 0.0133 | 11 | 34 | 47 | AASIYGGAGGQGTR | | | | | | | Mascot |
| | 1265.6233 | 1265.6366 | 0.0133 | 11 | 34 | 47 | AASIYGGAGGQGTR | | | 90 | 100 | | | Mascot |
| | 1291.6927 | 1291.6851 | -0.0076 | -6 | 407 | 418 | VMTVTQTLVDGK | | | | | | | Mascot |
| | 1324.693 | 1324.6937 | 0.0007 | 1 | 375 | 385 | MKLEAEIATYR | | | | | | | Mascot |
| | 1684.8501 | 1684.8528 | 0.0027 | 2 | 230 | 245 | NQISQSGVQVDVDAPK | | | | | | | Mascot |
| | 2210.1584 | 2210.1094 | -0.049 | -22 | 407 | 427 | VMTVTQTLVDGKVVSSSTETK | | | | | | | Mascot |
| **Spot No.** | **Accession No.** | **Protein Name** | | | | | | **Protein PI** | **Protein MW** | **Protein Score** | **Protein Score / C. I. %** | **Total Ion Score** | **Total Ion / C. I. %** | **Peptide number** |
| 1094 | gi\|22651065 | keratin-like | | | | | | 4.85 | 42534.4 | 224 | 100 | 157 | 100 | 13 |
| | **Peptide Information** | | | | | | | | | | | | | |
| | **Calc. Mass** | **Obsrv. Mass** | **± da** | **± ppm** | **Start Seq.** | **End Seq.** | **Sequence** | | | | **Ion Score / C. I. %** | **Modification** | | **Result Type** |
| | 976.488 | 976.4646 | -0.0234 | -24 | 309 | 317 | GEMAVRDAK | | | | | | | Mascot |
| | 1002.5466 | 1002.5302 | -0.0164 | -16 | 284 | 292 | LQSEIDAVK | | | | | | | |
| | 1017.5258 | 1017.5685 | 0.0427 | 42 | 332 | 339 | QDMARQIR | | | | | | | |
| | 1063.5419 | 1063.5529 | 0.011 | 10 | 193 | 200 | QIFEEEIR | | | | | | | Mascot |
| | 1079.5116 | 1079.5267 | 0.0151 | 14 | 232 | 240 | AQYEDIANR | | | | | | | Mascot |
| | 1186.6427 | 1186.6385 | -0.0042 | -4 | 320 | 329 | VKDLEDALQR | | | | | | | Mascot |
| | 1343.7278 | 1343.7421 | 0.0143 | 11 | 284 | 295 | LQSEIDAVKGQR | | | | | | | Mascot |
| | 1345.6781 | 1345.701 | 0.0229 | 17 | 220 | 231 | NLDMDAIVAEVR | | | | | | | Mascot |
| | 1345.6781 | 1345.701 | 0.0229 | 17 | 220 | 231 | NLDMDAIVAEVR | | | 47 | 98.77 | | | Mascot |
| | 1361.673 | 1361.6859 | 0.0129 | 9 | 220 | 231 | NLDMDAIVAEVR | | | | | Oxidation | | Mascot |
| | 1381.6669 | 1381.6456 | -0.0213 | -15 | 154 | 164 | TECENEFVLIK | | | | | Carbamidom | | Mascot |
| | 1449.7584 | 1449.7772 | 0.0188 | 13 | 181 | 192 | LESLTDEINFLR | | | | | | | Mascot |
| | 1449.7584 | 1449.7772 | 0.0188 | 13 | 181 | 192 | LESLTDEINFLR | | | 68 | 99.992 | | | Mascot |
| | 1486.7133 | 1486.7386 | 0.0253 | 17 | 296 | 308 | ANLENQIAEAEER | | | | | | | Mascot |
| | 1486.7133 | 1486.7386 | 0.0253 | 17 | 296 | 308 | ANLENQIAEAEER | | | 42 | 96.59 | | | Mascot |

| | | | | | | | | | | | | | | |
|---|---|---|---|---|---|---|---|---|---|---|---|---|---|---|
| | 1509.7618 | 1509.7504 | -0.0114 | -8 | 154 | 165 | TECENEFVLIKK | | | | | Carbamidom | | Mascot |
| | 1889.9967 | 1890.0021 | 0.0054 | 3 | 193 | 207 | QIFEEEIRELQSQIK | | | | | | | Mascot |
| **Spot No.** | **Accession No.** | **Protein Name** | | | | | | **Protein PI** | **Protein MW** | **Protein Score** | **Protein Score / C. I. %** | **Total Ion Score** | **Total Ion / C. I. %** | **Peptide number** |
| 616 | gi\|29335504 | keratin 18 | | | | | | 5.07 | 41247.4 | 62 | 86.998 | 37 | 88.586 | 8 |
| | **Peptide Information** | | | | | | | | | | | | | |
| | **Calc. Mass** | **Obsrv. Mass** | **± da** | **± ppm** | **Start Seq.** | **End Seq.** | **Sequence** | | | | **Ion Score / C. I. %** | **Modification** | | **Result Type** |
| | 807.3995 | 807.416 | 0.0165 | 20 | 91 | 97 | LAADDFR | | | | | | | Mascot |
| | 1041.6051 | 1041.594 | -0.0111 | -11 | 82 | 90 | LVLQIDNAR | | | | | | | Mascot |
| | 1041.6051 | 1041.594 | -0.0111 | -11 | 82 | 90 | LVLQIDNAR | | | 37 | 88.586 | | | Mascot |
| | 1116.6049 | 1116.5851 | -0.0198 | -18 | 61 | 69 | FQPIVDELR | | | | | | | Mascot |
| | 1122.5538 | 1122.5667 | 0.0129 | 11 | 72 | 81 | IFDATTNNAR | | | | | | | Mascot |
| | 1201.6423 | 1201.6194 | -0.0229 | -19 | 323 | 332 | LQDALEEQKK | | | | | | | Mascot |
| | 1223.6631 | 1223.6648 | 0.0017 | 1 | 98 | 107 | VKYESELSIR | | | | | | | Mascot |
| | 1256.5688 | 1256.6448 | 0.076 | 60 | 148 | 157 | NHDNEVMELR | | | | | | | Mascot |
| | 1286.7063 | 1286.644 | -0.0623 | -48 | 108 | 119 | QGVEADITGLRK | | | | | | | Mascot |
| **Spot No.** | **Accession No.** | **Protein Name** | | | | | | **Protein PI** | **Protein MW** | **Protein Score** | **Protein Score / C. I. %** | **Total Ion Score** | **Total Ion / C. I. %** | **Peptide number** |
| 549 | gi\|18760808 | malic | | | | | | 6.9 | 65141.4 | 151 | 100 | 135 | 100 | 5 |
| | **Peptide Information** | | | | | | | | | | | | | |
| | **Calc. Mass** | **Obsrv. Mass** | **± da** | **± ppm** | **Start Seq.** | **End Seq.** | **Sequence** | | | | **Ion Score / C. I. %** | **Modification** | | **Result Type** |
| | 945.5 | 945.4929 | -0.0071 | -8 | 150 | 158 | AVVVTDGER | | | | | | | Mascot |
| | 1531.7686 | 1531.6827 | -0.0859 | -56 | 527 | 539 | HKMATLHPEPQDK | | | | | | | Mascot |
| | 1531.7686 | 1531.6827 | -0.0859 | -56 | 527 | 539 | HKMATLHPEPQDK | | | | | | | Mascot |
| | 1788.7528 | 1788.762 | 0.0092 | 5 | 416 | 430 | AECTAEQCYTLTEGR | | | | | Carbamidom | | Mascot |
| | 1788.7528 | 1788.762 | 0.0092 | 5 | 416 | 430 | AECTAEQCYTLTEGR | | | 88 | 100 | Carbamidom | | Mascot |
| | 1832.9178 | 1832.9258 | 0.008 | 4 | 431 | 448 | GIFASGSPFDPVTLPDGR | | | | | | | Mascot |
| | 1832.9178 | 1832.9258 | 0.008 | 4 | 431 | 448 | GIFASGSPFDPVTLPDGR | | | 47 | 98.885 | | | Mascot |
| | 1961.0128 | 1961.009 | -0.0038 | -2 | 431 | 449 | GIFASGSPFDPVTLPDGR | | | | | | | Mascot |
| **Spot No.** | **Accession No.** | **Protein Name** | | | | | | **Protein PI** | **Protein MW** | **Protein Score** | **Protein Score / C. I. %** | **Total Ion Score** | **Total Ion / C. I. %** | **Peptide number** |
| 631 | gi\|52218932 | aldehyde | | | | | | 6.61 | 54061.2 | 304 | 100 | 249 | 100 | 10 |
| | **Peptide Information** | | | | | | | | | | | | | |
| | **Calc. Mass** | **Obsrv. Mass** | **± da** | **± ppm** | **Start Seq.** | **End Seq.** | **Sequence** | | | | **Ion Score / C. I. %** | **Modification** | | **Result Type** |
| | 900.5149 | 900.5208 | 0.0059 | 7 | 76 | 83 | LADLIEAR | | | | | | | Mascot |
| | 1050.4891 | 1050.5127 | 0.0236 | 22 | 56 | 64 | EAFPDWSAK | | | | | | | Mascot |
| | 1050.4891 | 1050.5127 | 0.0236 | 22 | 56 | 64 | EAFPDWSAK | | | 34 | 75.256 | | | Mascot |
| | 1268.6885 | 1268.7125 | 0.024 | 19 | 7 | 17 | YLVLENYIGGK | | | | | | | Mascot |
| | 1354.8053 | 1354.7256 | -0.0797 | -59 | 72 | 83 | VLNKLADLIEAR | | | | | | | Mascot |

| | | | | | | | | | | | | | | |
|---|---|---|---|---|---|---|---|---|---|---|---|---|---|---|
| | 1671.8185 | 1671.9368 | 0.1183 | 71 | 316 | 332 | TGVPSDPSNDNGALISK | | | | | | | Mascot |
| | 1745.7582 | 1745.8405 | 0.0823 | 47 | 278 | 292 | SSFSNQGEICLCTSR | | | | | Carbamidom | | Mascot |
| | 1745.7582 | 1745.8405 | 0.0823 | 47 | 278 | 292 | SSFSNQGEICLCTSR | | | 104 | 100 | Carbamidom | | Mascot |
| | 1786.9011 | 1787.0121 | 0.111 | 62 | 472 | 486 | DSYHFFTEVKSVTVK | | | | | | | Mascot |
| | 1985.9928 | 1986.0833 | 0.0905 | 46 | 314 | 332 | WKTGVPSDPSNDNGALISK | | | | | | | Mascot |
| | 2383.2253 | 2383.4028 | 0.1775 | 74 | 214 | 237 | AGDALVSHPDVPLISFTGSTATAR | | | | | | | Mascot |
| | 2383.2253 | 2383.4028 | 0.1775 | 74 | 214 | 237 | AGDALVSHPDVPLISFTGSTATAR | | | 111 | 100 | | | Mascot |
| | 3314.5081 | 3314.6953 | 0.1872 | 56 | 383 | 411 | DSSALMQEEIFGPVTCVTPFDEEEEV | | | | | Carbamidom | | Mascot |
| **Spot No.** | **Accession No.** | **Protein Name** | | | | | | **Protein PI** | **Protein MW** | **Protein Score** | **Protein Score / C. I. %** | **Total Ion Score** | **Total Ion / C. I. %** | **Peptide number** |
| 647 | gi\|47551317 | beta-enolase | | | | | | 6.25 | 47841.4 | 257 | 100 | 207 | 100 | 9 |
| | **Peptide Information** | | | | | | | | | | | | | |
| | **Calc. Mass** | **Obsrv. Mass** | **± da** | **± ppm** | **Start Seq.** | **End Seq.** | **Sequence** | | | | **Ion Score / C. I. %** | **Modification** | | **Result Type** |
| | 1072.531 | 1072.4968 | -0.0342 | -32 | 254 | 262 | SGKYDLDFK | | | | | | | Mascot |
| | 1231.6793 | 1231.6703 | -0.009 | -7 | 121 | 132 | AGAAEKGVPLYR | | | | | | | Mascot |
| | 1373.7059 | 1373.6915 | -0.0144 | -10 | 270 | 281 | HITGEQLGDLYK | | | | | | | Mascot |
| | 1373.7059 | 1373.6915 | -0.0144 | -10 | 270 | 281 | HITGEQLGDLYK | | | 92 | 100 | | | Mascot |
| | 1541.7642 | 1541.7504 | -0.0138 | -9 | 359 | 372 | LAQSNGWGVMVSHR | | | | | | | Mascot |
| | 1633.8214 | 1633.7933 | -0.0281 | -17 | 344 | 358 | VNQIGSVTESIQACK | | | | | Carbamidom | | Mascot |
| | 1649.8494 | 1649.8269 | -0.0225 | -14 | 16 | 30 | GNPTVEVDLYTTKGR | | | | | | | Mascot |
| | 1764.924 | 1764.922 | -0.002 | -1 | 33 | 50 | AAVPSGASTGVHEALELR | | | | | | | Mascot |
| | 1764.924 | 1764.922 | -0.002 | -1 | 33 | 50 | AAVPSGASTGVHEALELR | | | 115 | 100 | | | Mascot |
| | 2068.0935 | 2068.0906 | -0.0029 | -1 | 31 | 50 | FRAAVPSGASTGVHEALELR | | | | | | | Mascot |
| | 2105.0762 | 2105.0334 | -0.0428 | -20 | 307 | 326 | FTGSVDIQVVGDDLTVTNPK | | | | | | | Mascot |
| **Spot No.** | **Accession No.** | **Protein Name** | | | | | | **Protein PI** | **Protein MW** | **Protein Score** | **Protein Score / C. I. %** | **Total Ion Score** | **Total Ion / C. I. %** | **Peptide number** |
| 722 | gi\|77567762 | Eno3 protein | | | | | | 6.07 | 47118.3 | 261 | 100 | 203 | 100 | 10 |
| | **Peptide Information** | | | | | | | | | | | | | |
| | **Calc. Mass** | **Obsrv. Mass** | **± da** | **± ppm** | **Start Seq.** | **End Seq.** | **Sequence** | | | | **Ion Score / C. I. %** | **Modification** | | **Result Type** |
| | 800.3825 | 800.434 | 0.0515 | 64 | 247 | 252 | YDLDFK | | | | | | | Mascot |
| | 800.3825 | 800.434 | 0.0515 | 64 | 247 | 252 | YDLDFK | | | | | | | Mascot |
| | 814.4053 | 814.4361 | 0.0308 | 38 | 253 | 259 | SPDDPKR | | | | | | | Mascot |
| | 824.4083 | 824.451 | 0.0427 | 52 | 397 | 402 | YNQLMR | | | | | | | Mascot |
| | 824.4083 | 824.451 | 0.0427 | 52 | 397 | 402 | YNQLMR | | | 11 | 0 | | | Mascot |
| | 840.4032 | 840.4464 | 0.0432 | 51 | 397 | 402 | YNQLMR | | | 8 | 0 | Oxidation | | Mascot |
| | 840.4363 | 840.4464 | 0.0101 | 12 | 413 | 419 | FAGKDFR | | | | | | | Mascot |
| | 894.5043 | 894.4995 | -0.0048 | -5 | 99 | 106 | YLGKGTQK | | | | | | | Mascot |
| | 1018.6077 | 1018.5942 | -0.0135 | -13 | 39 | 47 | LALRMSISK | | | | | | | Mascot |
| | 1373.7059 | 1373.7434 | 0.0375 | 27 | 260 | 271 | HITGEQLGDLYK | | | | | | | Mascot |
| | 1373.7059 | 1373.7434 | 0.0375 | 27 | 260 | 271 | HITGEQLGDLYK | | | 78 | 99.999 | | | Mascot |
| | 1764.924 | 1764.9702 | 0.0462 | 26 | 75 | 92 | AAVPSGASTGVHEALELR | | | | | | | Mascot |
| | 1764.924 | 1764.9702 | 0.0462 | 26 | 75 | 92 | AAVPSGASTGVHEALELR | | | 114 | 100 | | | Mascot |

| | | | | | | | | | | | | | | |
|---|---|---|---|---|---|---|---|---|---|---|---|---|---|---|
| | 2105.0762 | 2105.114 | 0.0378 | 18 | 297 | 316 | FTGSVDIQVVGDDLTVTNPK | | | | | | | Mascot |
| | 2261.1772 | 2261.2847 | 0.1075 | 48 | 297 | 317 | FTGSVDIQVVGDDLTVTNPKR | | | | | | | Mascot |
| **Spot No.** | **Accession No.** | **Protein Name** | | | | | | **Protein PI** | **Protein MW** | **Protein Score** | **Protein Score / C. I. %** | **Total Ion Score** | **Total Ion / C. I. %** | **Peptide number** |
| 715 | gi\|47551317 | beta-enolase | | | | | | 6.25 | 47841.4 | 425 | 100 | 293 | 100 | 17 |
| | **Peptide Information** | | | | | | | | | | | | | |
| | **Calc. Mass** | **Obsrv. Mass** | **± da** | **± ppm** | **Start Seq.** | **End Seq.** | **Sequence** | | | | **Ion Score / C. I. %** | **Modification** | | **Result Type** |
| | 824.4083 | 824.4151 | 0.0068 | 8 | 407 | 412 | YNQLMR | | | | | | | Mascot |
| | 824.4083 | 824.4151 | 0.0068 | 8 | 407 | 412 | YNQLMR | | | 23 | 0 | | | Mascot |
| | 840.4363 | 840.435 | -0.0013 | -2 | 423 | 429 | FAGKDFR | | | | | | | Mascot |
| | 1032.5255 | 1032.5514 | 0.0259 | 25 | 327 | 334 | RIQQACEK | | | | | Carbamidom | | Mascot |
| | 1033.4844 | 1033.5127 | 0.0283 | 27 | 395 | 403 | TGAPCRSER | | | | | Carbamidom | | Mascot |
| | 1072.531 | 1072.5143 | -0.0167 | -16 | 254 | 262 | SGKYDLDFK | | | | | | | Mascot |
| | 1136.6245 | 1136.6095 | -0.015 | -13 | 404 | 412 | LAKYNQLMR | | | | | | | Mascot |
| | 1231.6793 | 1231.6917 | 0.0124 | 10 | 121 | 132 | AGAAEKGVPLYR | | | | | | | Mascot |
| | 1296.614 | 1296.6875 | 0.0735 | 57 | 93 | 103 | FMLELDGTENK | | | | | | | Mascot |
| | 1373.7059 | 1373.7271 | 0.0212 | 15 | 270 | 281 | HITGEQLGDLYK | | | | | | | Mascot |
| | 1373.7059 | 1373.7271 | 0.0212 | 15 | 270 | 281 | HITGEQLGDLYK | | | 76 | 99.998 | | | Mascot |
| | 1541.7642 | 1541.7979 | 0.0337 | 22 | 359 | 372 | LAQSNGWGVMVSHR | | | | | | | Mascot |
| | 1556.803 | 1556.7948 | -0.0082 | -5 | 240 | 253 | IIIGMDVAASEFFK | | | | | Oxidation | | Mascot |
| | 1633.8214 | 1633.8584 | 0.037 | 23 | 344 | 358 | VNQIGSVTESIQACK | | | | | Carbamidom | | Mascot |
| | 1633.8214 | 1633.8584 | 0.037 | 23 | 344 | 358 | VNQIGSVTESIQACK | | | 57 | 99.875 | Carbamidom | | Mascot |
| | 1764.924 | 1764.9777 | 0.0537 | 30 | 33 | 50 | AAVPSGASTGVHEALELR | | | | | | | Mascot |
| | 1764.924 | 1764.9777 | 0.0537 | 30 | 33 | 50 | AAVPSGASTGVHEALELR | | | 118 | 100 | | | Mascot |
| | 2068.0935 | 2068.1602 | 0.0667 | 32 | 31 | 50 | FRAAVPSGASTGVHEALELR | | | | | | | Mascot |
| | 2068.0935 | 2068.1602 | 0.0667 | 32 | 31 | 50 | FRAAVPSGASTGVHEALELR | | | 19 | 0 | | | Mascot |
| | 2105.0762 | 2105.1055 | 0.0293 | 14 | 307 | 326 | FTGSVDIQVVGDDLTVTNPK | | | | | | | Mascot |
| | 2261.1772 | 2261.2593 | 0.0821 | 36 | 307 | 327 | FTGSVDIQVVGDDLTVTNPKR | | | | | | | Mascot |
| | 2743.342 | 2743.3774 | 0.0354 | 13 | 203 | 228 | DATNVGDEGGFAPNILENNEALELLK | | | | | | | Mascot |
| **Spot No.** | **Accession No.** | **Protein Name** | | | | | | **Protein PI** | **Protein MW** | **Protein Score** | **Protein Score / C. I. %** | **Total Ion Score** | **Total Ion / C. I. %** | **Peptide number** |
| 724 | gi\|77567762 | Eno3 protein | | | | | | 5.47 | 47118.3 | 254 | 100 | 185 | 100 | 11 |
| | **Peptide Information** | | | | | | | | | | | | | |
| | **Calc. Mass** | **Obsrv. Mass** | **± da** | **± ppm** | **Start Seq.** | **End Seq.** | **Sequence** | | | | **Ion Score / C. I. %** | **Modification** | | **Result Type** |
| | 824.4083 | 824.4182 | 0.0099 | 12 | 397 | 402 | YNQLMR | | | | | | | Mascot |
| | 1032.5255 | 1032.5294 | 0.0039 | 4 | 317 | 324 | RIQQACEK | | | | | Carbamidom | | Mascot |
| | 1033.4844 | 1033.5177 | 0.0333 | 32 | 385 | 393 | TGAPCRSER | | | | | Carbamidom | | Mascot |
| | 1131.5892 | 1131.5745 | -0.0147 | -13 | 403 | 412 | IEEELGDKAK | | | | | | | Mascot |
| | 1136.6245 | 1136.6012 | -0.0233 | -20 | 394 | 402 | LAKYNQLMR | | | | | | | Mascot |
| | 1231.6793 | 1231.6847 | 0.0054 | 4 | 111 | 122 | AGAAEKGVPLYR | | | | | | | Mascot |
| | 1373.7059 | 1373.7177 | 0.0118 | 9 | 260 | 271 | HITGEQLGDLYK | | | | | | | Mascot |
| | 1373.7059 | 1373.7177 | 0.0118 | 9 | 260 | 271 | HITGEQLGDLYK | | | 84 | 100 | | | Mascot |

| | | | | | | | | | | | | | | |
|---|---|---|---|---|---|---|---|---|---|---|---|---|---|---|
| | 1764.924 | 1764.9413 | 0.0173 | 10 | 75 | 92 | AAVPSGASTGVHEALELR | | | | | | | Mascot |
| | 1764.924 | 1764.9413 | 0.0173 | 10 | 75 | 92 | AAVPSGASTGVHEALELR | | | 101 | 100 | | | Mascot |
| | 2105.0762 | 2105.0449 | -0.0313 | -15 | 297 | 316 | FTGSVDIQVVGDDLTVTNPK | | | | | | | Mascot |
| | 2261.1772 | 2261.1729 | -0.0043 | -2 | 297 | 317 | FTGSVDIQVVGDDLTVTNPKR | | | | | | | Mascot |
| | 2743.342 | 2743.2673 | -0.0747 | -27 | 193 | 218 | DATNVGDEGGFAPNILENNEALELLK | | | | | | | Mascot |
| **Spot No.** | **Accession No.** | **Protein Name** | | | | | | **Protein PI** | **Protein MW** | **Protein Score** | **Protein Score / C. I. %** | **Total Ion Score** | **Total Ion / C. I. %** | **Peptide number** |
| 720 | gi\|47551317 | beta-enolase | | | | | | 6.25 | 47841.4 | 358 | 100 | 246 | 100 | 15 |
| | **Peptide Information** | | | | | | | | | | | | | |
| | **Calc. Mass** | **Obsrv. Mass** | **± da** | **± ppm** | **Start Seq.** | **End Seq.** | **Sequence** | | | | **Ion Score / C. I. %** | **Modification** | | **Result Type** |
| | 800.3825 | 800.386 | 0.0035 | 4 | 257 | 262 | YDLDFK | | | | | | | Mascot |
| | 814.4053 | 814.4082 | 0.0029 | 4 | 263 | 269 | SPDDPKR | | | | | | | Mascot |
| | 824.4083 | 824.4144 | 0.0061 | 7 | 407 | 412 | YNQLMR | | | | | | | Mascot |
| | 824.4083 | 824.4144 | 0.0061 | 7 | 407 | 412 | YNQLMR | | | | | | | Mascot |
| | 840.4032 | 840.4014 | -0.0018 | -2 | 407 | 412 | YNQLMR | | | | | Oxidation | | Mascot |
| | 876.4244 | 876.4503 | 0.0259 | 30 | 328 | 334 | IQQACEK | | | | | Carbamidom | | Mascot |
| | 1033.4844 | 1033.5186 | 0.0342 | 33 | 395 | 403 | TGAPCRSER | | | | | Carbamidom | | Mascot |
| | 1136.6245 | 1136.6324 | 0.0079 | 7 | 404 | 412 | LAKYNQLMR | | | | | | | Mascot |
| | 1231.6793 | 1231.6902 | 0.0109 | 9 | 121 | 132 | AGAAEKGVPLYR | | | | | | | Mascot |
| | 1296.614 | 1296.6901 | 0.0761 | 59 | 93 | 103 | FMLELDGTENK | | | | | | | Mascot |
| | 1373.7059 | 1373.7015 | -0.0044 | -3 | 270 | 281 | HITGEQLGDLYK | | | | | | | Mascot |
| | 1373.7059 | 1373.7015 | -0.0044 | -3 | 270 | 281 | HITGEQLGDLYK | | | 47 | 99.102 | | | Mascot |
| | 1633.8214 | 1633.8386 | 0.0172 | 11 | 344 | 358 | VNQIGSVTESIQACK | | | | | Carbamidom | | Mascot |
| | 1764.924 | 1764.921 | -0.003 | -2 | 33 | 50 | AAVPSGASTGVHEALELR | | | | | | | Mascot |
| | 1764.924 | 1764.921 | -0.003 | -2 | 33 | 50 | AAVPSGASTGVHEALELR | | | 98 | 100 | | | Mascot |
| | 2105.0762 | 2105.0444 | -0.0318 | -15 | 307 | 326 | FTGSVDIQVVGDDLTVTNPK | | | | | | | Mascot |
| | 2105.0762 | 2105.0444 | -0.0318 | -15 | 307 | 326 | FTGSVDIQVVGDDLTVTNPK | | | 66 | 99.987 | | | Mascot |
| | 2261.1772 | 2261.1531 | -0.0241 | -11 | 307 | 327 | FTGSVDIQVVGDDLTVTNPKR | | | | | | | Mascot |
| | 2261.1772 | 2261.1531 | -0.0241 | -11 | 307 | 327 | FTGSVDIQVVGDDLTVTNPKR | | | 35 | 85.666 | | | Mascot |
| | 2353.1592 | 2353.1262 | -0.033 | -14 | 373 | 394 | SGETEDTFIADLVVGLCTGUIK | | | | | Carbamidom | | Mascot |
| | 2743.342 | 2743.2751 | -0.0669 | -24 | 203 | 228 | DATNVGDEGGFAPNILENNEALELLK | | | | | | | Mascot |
| **Spot No.** | **Accession No.** | **Protein Name** | | | | | | **Protein PI** | **Protein MW** | **Protein Score** | **Protein Score / C. I. %** | **Total Ion Score** | **Total Ion / C. I. %** | **Peptide number** |
| 781 | gi\|41388972 | Pgk1 protein | | | | | | 6.47 | 45146.1 | 254 | 100 | 211 | 100 | 9 |
| | **Peptide Information** | | | | | | | | | | | | | |
| | **Calc. Mass** | **Obsrv. Mass** | **± da** | **± ppm** | **Start Seq.** | **End Seq.** | **Sequence** | | | | **Ion Score / C. I. %** | **Modification** | | **Result Type** |
| | 892.4886 | 892.4644 | -0.0242 | -27 | 323 | 330 | LYAEAVAR | | | | | | | Mascot |
| | 892.4886 | 892.4644 | -0.0242 | -27 | 323 | 330 | LYAEAVAR | | | 34 | 73.522 | | | Mascot |
| | 949.4811 | 949.436 | -0.0451 | -47 | 23 | 30 | VDFNVPMK | | | | | | | Mascot |
| | 1219.6569 | 1219.6316 | -0.0253 | -21 | 76 | 86 | YSLEPVAAELK | | | | | | | Mascot |
| | 1634.7922 | 1634.8364 | 0.0442 | 27 | 157 | 171 | LGDVYVNDAFGTAHR | | | | | | | Mascot |
| | 1634.7922 | 1634.8364 | 0.0442 | 27 | 157 | 171 | LGDVYVNDAFGTAHR | | | 90 | 100 | | | Mascot |

| | | | | | | | | | | | | | | |
|---|---|---|---|---|---|---|---|---|---|---|---|---|---|---|
| | 1683.7942 | 1683.8536 | 0.0594 | 35 | 42 | 56 | AAVPSIQHCLDNCAK | | | | | Carbamidom | | Mascot |
| | 1727.886 | 1727.9292 | 0.0432 | 25 | 231 | 246 | VNEMIIGGGMAFTFLK | | | | | | | Mascot |
| | 1740.9127 | 1740.939 | 0.0263 | 15 | 389 | 406 | VSHVSTGGGASLELLEGK | | | | | | | Mascot |
| | 1744.9844 | 1744.8889 | -0.0955 | -55 | 76 | 91 | YSLEPVAAELKNLLGK | | | | | | | Mascot |
| | 1795.9371 | 1796.0029 | 0.0658 | 37 | 107 | 123 | ACADPPAGSVILLENLR | | | | | Carbamidom | | Mascot |
| | 1795.9371 | 1796.0029 | 0.0658 | 37 | 107 | 123 | ACADPPAGSVILLENLR | | | 87 | 100 | Carbamidom | | Mascot |
| **Spot No.** | **Accession No.** | **Protein Name** | | | | | | **Protein PI** | **Protein MW** | **Protein Score** | **Protein Score / C. I. %** | **Total Ion Score** | **Total Ion / C. I. %** | **Peptide number** |
| 2044 | gi\|50540008 | L-lactate | | | | | | 6.39 | 36857.4 | 222 | 100 | 160 | 100 | 10 |
| | **Peptide Information** | | | | | | | | | | | | | |
| | **Calc. Mass** | **Obsrv. Mass** | **± da** | **± ppm** | **Start Seq.** | **End Seq.** | **Sequence** | | | | **Ion Score / C. I. %** | **Modification** | | **Result Type** |
| | 1085.556 | 1085.5697 | 0.0137 | 13 | 171 | 178 | FRYLMAER | | | | | | | Mascot |
| | 1101.551 | 1101.5686 | 0.0176 | 16 | 171 | 178 | FRYLMAER | | | | | Oxidation | | Mascot |
| | 1150.6104 | 1150.6163 | 0.0059 | 5 | 235 | 244 | VVDSAYEVIR | | | | | | | Mascot |
| | 1150.6104 | 1150.6163 | 0.0059 | 5 | 235 | 244 | VVDSAYEVIR | | | 70 | 99.995 | | | Mascot |
| | 1189.6172 | 1189.6152 | -0.002 | -2 | 159 | 170 | VIGSGTNLDSAR | | | | | | | Mascot |
| | 1189.6172 | 1189.6152 | -0.002 | -2 | 159 | 170 | VIGSGTNLDSAR | | | 31 | 56.053 | | | Mascot |
| | 1243.6277 | 1243.6459 | 0.0182 | 15 | 215 | 225 | LNPDIGKDTDR | | | | | | | Mascot |
| | 1256.6205 | 1256.6276 | 0.0071 | 6 | 320 | 329 | SADMLWHIQR | | | | | | | Mascot |
| | 1256.6205 | 1256.6276 | 0.0071 | 6 | 320 | 329 | SADMLWHIQR | | | 58 | 99.916 | | | Mascot |
| | 1272.6154 | 1272.6235 | 0.0081 | 6 | 320 | 329 | SADMLWHIQR | | | | | Oxidation | | Mascot |
| | 1278.7052 | 1278.682 | -0.0232 | -18 | 234 | 244 | KVVDSAYEVIR | | | | | | | Mascot |
| | 1482.7772 | 1482.7573 | -0.0199 | -13 | 157 | 170 | HRVIGSGTNLDSAR | | | | | | | Mascot |
| | 1492.7867 | 1492.774 | -0.0127 | -9 | 159 | 172 | VIGSGTNLDSARFR | | | | | | | Mascot |
| | 1556.814 | 1556.8376 | 0.0236 | 15 | 101 | 113 | QQEGESRLNLVQR | | | | | | | Mascot |
| | 1727.9399 | 1727.911 | -0.0289 | -17 | 92 | 107 | IVVVTAGVRQQEGESR | | | | | | | Mascot |
| | 1727.9399 | 1727.911 | -0.0289 | -17 | 92 | 107 | IVVVTAGVRQQEGESR | | | | | | | Mascot |
| **Spot No.** | **Accession No.** | **Protein Name** | | | | | | **Protein PI** | **Protein MW** | **Protein Score** | **Protein Score / C. I. %** | **Total Ion Score** | **Total Ion / C. I. %** | **Peptide number** |
| 1432 | gi\|21351421 | Sorbitol | | | | | | 5.61 | 38674.9 | 248 | 100 | 226 | 100 | 5 |
| | **Peptide Information** | | | | | | | | | | | | | |
| | **Calc. Mass** | **Obsrv. Mass** | **± da** | **± ppm** | **Start Seq.** | **End Seq.** | **Sequence** | | | | **Ion Score / C. I. %** | **Modification** | | **Result Type** |
| | 923.5309 | 923.5469 | 0.016 | 17 | 88 | 96 | VAVEPGVPR | | | | | | | Mascot |
| | 923.5309 | 923.5469 | 0.016 | 17 | 88 | 96 | VAVEPGVPR | | | 46 | 98.36 | | | Mascot |
| | 1026.4462 | 1026.4734 | 0.0272 | 26 | 131 | 138 | HSANFCYK | | | | | Carbamidom | | Mascot |
| | 1071.543 | 1071.5082 | -0.0348 | -32 | 223 | 232 | GDGPEELAKR | | | | | | | Mascot |
| | 1788.8118 | 1788.9075 | 0.0957 | 53 | 297 | 311 | YCNTWPMAIAMLASK | | | | | Carbamidom | | Mascot |
| | 2653.3291 | 2653.4299 | 0.1008 | 38 | 139 | 162 | LPDNVTYEEGALIEPLSVGIHACR | | | | | Carbamidom | | Mascot |
| | 2653.3291 | 2653.4299 | 0.1008 | 38 | 139 | 162 | LPDNVTYEEGALIEPLSVGIHACR | | | 181 | 100 | Carbamidom | | Mascot |

| Spot No. | Accession No. | Protein Name | | | | | | Protein PI | Protein MW | Protein Score | Protein Score / C. I. % | Total Ion Score | Total Ion / C. I. % | Peptide number |
|---|---|---|---|---|---|---|---|---|---|---|---|---|---|---|
| 1823 | gi\|29820122 | glyceraldehy | | | | | | 6.74 | 35504.2 | 327 | 100 | 275 | 100 | 8 |
| | Peptide Information | | | | | | | | | | | | | |
| | Calc. Mass | Obsrv. Mass | ± da | ± ppm | Start Seq. | End Seq. | Sequence | | | | Ion Score / C. I. % | Modification | | Result Type |
| | 802.4318 | 802.4451 | 0.0133 | 17 | 190 | 195 | LWRDGR | | | | | | | Mascot |
| | 1495.8479 | 1495.8385 | -0.0094 | -6 | 230 | 243 | VPTPNVSVVDLTVR | | | | | | | Mascot |
| | 1495.8479 | 1495.8385 | -0.0094 | -6 | 230 | 243 | VPTPNVSVVDLTVR | | | 63 | 99.972 | | | Mascot |
| | 1710.9497 | 1710.8623 | -0.0874 | -51 | 196 | 214 | GAGQNIIPASTGAAKAVGK | | | | | | | Mascot |
| | 1763.8024 | 1763.8232 | 0.0208 | 12 | 305 | 318 | LVTWYDNEFGYSNR | | | | | | | Mascot |
| | 1763.8024 | 1763.8232 | 0.0208 | 12 | 305 | 318 | LVTWYDNEFGYSNR | | | 57 | 99.889 | | | Mascot |
| | 1805.8884 | 1805.855 | -0.0334 | -18 | 141 | 157 | VVSNASCTTNCLAPLAK | | | | | Carbamidom | | Mascot |
| | 1861.9443 | 1861.9357 | -0.0086 | -5 | 287 | 304 | SSIFDAGAGIALNDHFVK | | | | | | | Mascot |
| | 1861.9443 | 1861.9357 | -0.0086 | -5 | 287 | 304 | SSIFDAGAGIALNDHFVK | | | 94 | 100 | | | Mascot |
| | 2246.0032 | 2246.0007 | -0.0025 | -1 | 267 | 286 | GILGYTEDQVVSTDFNGDCR | | | | | Carbamidom | | Mascot |
| | 2246.0032 | 2246.0007 | -0.0025 | -1 | 267 | 286 | GILGYTEDQVVSTDFNGDCR | | | 60 | 99.944 | Carbamidom | | Mascot |
| | 2987.3511 | 2987.2834 | -0.0677 | -23 | 259 | 286 | AAADGPMKGILGYTEDQVVSTDFNG | | | | | Carbamidom | | Mascot |
| Spot No. | Accession No. | Protein Name | | | | | | Protein PI | Protein MW | Protein Score | Protein Score / C. I. % | Total Ion Score | Total Ion / C. I. % | Peptide number |
| 1546 | gi\|46849373 | fructose- | | | | | | 5.21 | 36637.2 | 249 | 100 | 232 | 100 | 4 |
| | Peptide Information | | | | | | | | | | | | | |
| | Calc. Mass | Obsrv. Mass | ± da | ± ppm | Start Seq. | End Seq. | Sequence | | | | Ion Score / C. I. % | Modification | | Result Type |
| | 1217.5757 | 1217.6454 | 0.0697 | 57 | 14 | 23 | INVENTEENR | | | | | | | Mascot |
| | 1373.6768 | 1373.7477 | 0.0709 | 52 | 14 | 24 | INVENTEENRR | | | | | | | Mascot |
| | 1373.6768 | 1373.7477 | 0.0709 | 52 | 14 | 24 | INVENTEENRR | | | 60 | 99.944 | | | Mascot |
| | 1655.8976 | 1655.9983 | 0.1007 | 61 | 272 | 288 | ALQASALAAWGGKAANR | | | | | | | Mascot |
| | 2042.0336 | 2042.2073 | 0.1737 | 85 | 121 | 140 | ISDGCPSALAIAENANVLAR | | | | | Carbamidom | | Mascot |
| | 2042.0336 | 2042.2073 | 0.1737 | 85 | 121 | 140 | ISDGCPSALAIAENANVLAR | | | 172 | 100 | Carbamidom | | Mascot |
| Spot No. | Accession No. | Protein Name | | | | | | Protein PI | Protein MW | Protein Score | Protein Score / C. I. % | Total Ion Score | Total Ion / C. I. % | Peptide number |
| 1600 | gi\|77380115 | fructose 1,6- | | | | | | 6.03 | 4355.1 | 92 | 99.986 | 81 | 100 | 1 |
| | Peptide Information | | | | | | | | | | | | | |
| | Calc. Mass | Obsrv. Mass | ± da | ± ppm | Start Seq. | End Seq. | Sequence | | | | Ion Score / C. I. % | Modification | | Result Type |
| | 2042.0336 | 2042.0649 | 0.0313 | 15 | 12 | 31 | ISDGCPSALAIAENANVLAR | | | | | Carbamidom | | Mascot |
| | 2042.0336 | 2042.0649 | 0.0313 | 15 | 12 | 31 | ISDGCPSALAIAENANVLAR | | | 81 | 100 | Carbamidom | | Mascot |
| Spot No. | Accession No. | Protein Name | | | | | | Protein PI | Protein MW | Protein Score | Protein Score / C. I. % | Total Ion Score | Total Ion / C. I. % | Peptide number |

| | | | | | | | | | | | | | | |
|---|---|---|---|---|---|---|---|---|---|---|---|---|---|---|
| 1245 | gi\|34851444 | PREDICTED | | | | | | 6.21 | 39885.2 | 428 | 100 | 351 | 100 | 11 |
| | Peptide Information | | | | | | | | | | | | | |
| | Calc. Mass | Obsrv. Mass | ± da | ± ppm | Start Seq. | End Seq. | Sequence | | | | Ion Score / C. I. % | Modification | | Result Type |
| | 812.3243 | 812.3148 | -0.0095 | -12 | 141 | 147 | DGCDFAK | | | | | Carbamidom | | Mascot |
| | 899.3927 | 899.3861 | -0.0066 | -7 | 202 | 208 | CQYATEK | | | | | Carbamidom | | Mascot |
| | 1154.5048 | 1154.5134 | 0.0086 | 7 | 141 | 149 | DGCDFAKWR | | | | | Carbamidom | | Mascot |
| | 1217.5757 | 1217.6104 | 0.0347 | 28 | 47 | 56 | INVENTEENR | | | | | | | Mascot |
| | 1373.6768 | 1373.7042 | 0.0274 | 20 | 47 | 57 | INVENTEENRR | | | | | | | Mascot |
| | 1373.6768 | 1373.7042 | 0.0274 | 20 | 47 | 57 | INVENTEENRR | | | 49 | 99.278 | | | Mascot |
| | 1536.7687 | 1536.7935 | 0.0248 | 16 | 29 | 43 | GILAADESTGTMAKR | | | | | Oxidation | | Mascot |
| | 1650.8521 | 1650.9136 | 0.0615 | 37 | 244 | 258 | YTPQEVAMATVTALR | | | | | | | Mascot |
| | 1650.8521 | 1650.9136 | 0.0615 | 37 | 244 | 258 | YTPQEVAMATVTALR | | | 54 | 99.744 | | | Mascot |
| | 1778.9469 | 1779.0304 | 0.0835 | 47 | 243 | 258 | KYTPQEVAMATVTALR | | | | | | | Mascot |
| | 1794.9419 | 1794.9639 | 0.022 | 12 | 243 | 258 | KYTPQEVAMATVTALR | | | | | Oxidation | | Mascot |
| | 1806.9531 | 1807.04 | 0.0869 | 48 | 244 | 259 | YTPQEVAMATVTALRR | | | | | | | Mascot |
| | 1822.948 | 1823.0336 | 0.0856 | 47 | 244 | 259 | YTPQEVAMATVTALRR | | | | | Oxidation | | Mascot |
| | 2042.0336 | 2042.1663 | 0.1327 | 65 | 154 | 173 | ISDGCPSALAIAENANVLAR | | | | | Carbamidom | | Mascot |
| | 2042.0336 | 2042.1663 | 0.1327 | 65 | 154 | 173 | ISDGCPSALAIAENANVLAR | | | 156 | 100 | Carbamidom | | Mascot |
| | 3176.5681 | 3176.8335 | 0.2654 | 84 | 174 | 201 | YASICQQNGLVPIVEPEILPDGDHDL | | | | | Carbamidom | | Mascot |
| | 3176.5681 | 3176.8335 | 0.2654 | 84 | 174 | 201 | YASICQQNGLVPIVEPEILPDGDHDL | | | 93 | 100 | Carbamidom | | Mascot |
| Spot No. | Accession No. | Protein Name | | | | | | Protein PI | Protein MW | Protein Score | Protein Score / C. I. % | Total Ion Score | Total Ion / C. I. % | Peptide number |
| 1240 | gi\|34851444 | PREDICTED | | | | | | 6.42 | 39885.2 | 595 | 100 | 537 | 100 | 9 |
| | Peptide Information | | | | | | | | | | | | | |
| | Calc. Mass | Obsrv. Mass | ± da | ± ppm | Start Seq. | End Seq. | Sequence | | | | Ion Score / C. I. % | Modification | | Result Type |
| | 812.3243 | 812.2935 | -0.0308 | -38 | 141 | 147 | DGCDFAK | | | | | Carbamidom | | Mascot |
| | 899.3927 | 899.3693 | -0.0234 | -26 | 202 | 208 | CQYATEK | | | | | Carbamidom | | Mascot |
| | 1217.5757 | 1217.5636 | -0.0121 | -10 | 47 | 56 | INVENTEENR | | | | | | | Mascot |
| | 1373.6768 | 1373.6707 | -0.0061 | -4 | 47 | 57 | INVENTEENRR | | | | | | | Mascot |
| | 1373.6768 | 1373.6707 | -0.0061 | -4 | 47 | 57 | INVENTEENRR | | | 54 | 99.762 | | | Mascot |
| | 1650.8521 | 1650.8668 | 0.0147 | 9 | 244 | 258 | YTPQEVAMATVTALR | | | | | | | Mascot |
| | 1650.8521 | 1650.8668 | 0.0147 | 9 | 244 | 258 | YTPQEVAMATVTALR | | | 118 | 100 | | | Mascot |
| | 1666.8469 | 1666.8524 | 0.0055 | 3 | 244 | 258 | YTPQEVAMATVTALR | | | | | Oxidation | | Mascot |
| | 1778.9469 | 1778.9694 | 0.0225 | 13 | 243 | 258 | KYTPQEVAMATVTALR | | | | | | | Mascot |
| | 1778.9469 | 1778.9694 | 0.0225 | 13 | 243 | 258 | KYTPQEVAMATVTALR | | | 69 | 99.993 | | | Mascot |
| | 1794.9419 | 1794.9181 | -0.0238 | -13 | 243 | 258 | KYTPQEVAMATVTALR | | | | | Oxidation | | Mascot |
| | 1806.9531 | 1806.9663 | 0.0132 | 7 | 244 | 259 | YTPQEVAMATVTALRR | | | | | | | Mascot |
| | 1822.948 | 1822.9426 | -0.0054 | -3 | 244 | 259 | YTPQEVAMATVTALRR | | | | | Oxidation | | Mascot |
| | 2042.0336 | 2042.0836 | 0.05 | 24 | 154 | 173 | ISDGCPSALAIAENANVLAR | | | | | Carbamidom | | Mascot |
| | 2042.0336 | 2042.0836 | 0.05 | 24 | 154 | 173 | ISDGCPSALAIAENANVLAR | | | 168 | 100 | Carbamidom | | Mascot |
| | 3176.5681 | 3176.7026 | 0.1345 | 42 | 174 | 201 | YASICQQNGLVPIVEPEILPDGDHDL | | | | | Carbamidom | | Mascot |
| | 3176.5681 | 3176.7026 | 0.1345 | 42 | 174 | 201 | YASICQQNGLVPIVEPEILPDGDHDL | | | 128 | 100 | Carbamidom | | Mascot |

| Spot No. | Accession No. | Protein Name | | | | | | Protein PI | Protein MW | Protein Score | Protein Score / C. I. % | Total Ion Score | Total Ion / C. I. % | Peptide number |
|---|---|---|---|---|---|---|---|---|---|---|---|---|---|---|
| 1636 | gi\|15767918 | HSP70 | | | | | | 5.13 | 69952.1 | 105 | 100 | 65 | 99.982 | 10 |
| | Peptide Information | | | | | | | | | | | | | |
| | Calc. Mass | Obsrv. Mass | ± da | ± ppm | Start Seq. | End Seq. | Sequence | | | | Ion Score / C. I. % | Modification | | Result Type |
| | 1217.6307 | 1217.6881 | 0.0574 | 47 | 166 | 177 | DAGTIAGLNVMR | | | | | | | Mascot |
| | 1228.6281 | 1228.7017 | 0.0736 | 60 | 28 | 38 | VEIIANDQGNR | | | | | | | Mascot |
| | 1240.6241 | 1240.686 | 0.0619 | 50 | 133 | 143 | MKETAEAYLGK | | | | | | | Mascot |
| | 1313.6195 | 1313.7288 | 0.1093 | 83 | 307 | 316 | FEELNMDLFR | | | | | | | Mascot |
| | 1487.7013 | 1487.7661 | 0.0648 | 44 | 39 | 51 | TTPSYVAFTDTER | | | | | | | Mascot |
| | 1487.7013 | 1487.7661 | 0.0648 | 44 | 39 | 51 | TTPSYVAFTDTER | | | 65 | 99.982 | | | Mascot |
| | 1536.7979 | 1536.885 | 0.0871 | 57 | 119 | 132 | TFAPEEISAMVLTK | | | | | | | Mascot |
| | 1567.6805 | 1567.7728 | 0.0923 | 59 | 521 | 533 | MVNDAERFADEDR | | | | | | | Mascot |
| | 1576.8806 | 1576.8988 | 0.0182 | 12 | 104 | 118 | SKPHIQVDIGGGQIK | | | | | | | Mascot |
| | 1787.881 | 1788.0402 | 0.1592 | 89 | 539 | 553 | IDSRNELESYAYSLK | | | | | | | Mascot |
| | 1827.9231 | 1827.9902 | 0.0671 | 37 | 119 | 134 | TFAPEEISAMVLTKMK | | | | | Oxidation | | Mascot |
| Spot No. | Accession No. | Protein Name | | | | | | Protein PI | Protein MW | Protein Score | Protein Score / C. I. % | Total Ion Score | Total Ion / C. I. % | Peptide number |
| 1793 | gi\|31942994 | apolipoprotei | | | | | | 5.26 | 12145.3 | 310 | 100 | 262 | 100 | 6 |
| | Peptide Information | | | | | | | | | | | | | |
| | Calc. Mass | Obsrv. Mass | ± da | ± ppm | Start Seq. | End Seq. | Sequence | | | | Ion Score / C. I. % | Modification | | Result Type |
| | 888.4785 | 888.467 | -0.0115 | -13 | 42 | 48 | DIEDLRK | | | | | | | Mascot |
| | 913.5717 | 913.5345 | -0.0372 | -41 | 92 | 99 | TKLEPVVK | | | | | | | Mascot |
| | 947.4727 | 947.4717 | -0.001 | -1 | 85 | 91 | QRMEEVR | | | | | | | Mascot |
| | 963.4676 | 963.456 | -0.0116 | -12 | 85 | 91 | QRMEEVR | | | | | Oxidation | | Mascot |
| | 1300.6678 | 1300.6538 | -0.014 | -11 | 49 | 58 | QLEPMREELR | | | | | | | Mascot |
| | 1300.6678 | 1300.6538 | -0.014 | -11 | 49 | 58 | QLEPMREELR | | | 40 | 93.607 | | | Mascot |
| | 1316.6627 | 1316.6406 | -0.0221 | -17 | 49 | 58 | QLEPMREELR | | | | | Oxidation | | Mascot |
| | 1866.9385 | 1866.9338 | -0.0047 | -3 | 1 | 16 | VFLGQSIDNLHGYFEK | | | | | | | Mascot |
| | 1866.9385 | 1866.9338 | -0.0047 | -3 | 1 | 16 | VFLGQSIDNLHGYFEK | | | 91 | 100 | | | Mascot |
| | 2179.1394 | 2179.156 | 0.0166 | 8 | 17 | 36 | AFEFITPVATQVLDATATQR | | | | | | | Mascot |
| | 2179.1394 | 2179.156 | 0.0166 | 8 | 17 | 36 | AFEFITPVATQVLDATATQR | | | 132 | 100 | | | Mascot |
| Spot No. | Accession No. | Protein Name | | | | | | Protein PI | Protein MW | Protein Score | Protein Score / C. I. % | Total Ion Score | Total Ion / C. I. % | Peptide number |
| 1795 | gi\|11290111 | glutathione | | | | | | 5.31 | 26301.7 | 269 | 100 | 217 | 100 | 9 |
| | Peptide Information | | | | | | | | | | | | | |
| | Calc. Mass | Obsrv. Mass | ± da | ± ppm | Start Seq. | End Seq. | Sequence | | | | Ion Score / C. I. % | Modification | | Result Type |
| | 936.4938 | 936.4884 | -0.0054 | -6 | 5 | 11 | LAYWDIR | | | | | | | Mascot |
| | 936.4938 | 936.4884 | -0.0054 | -6 | 5 | 11 | LAYWDIR | | | 48 | 98.965 | | | Mascot |
| | 1017.551 | 1017.5524 | 0.0014 | 1 | 70 | 78 | VVQSNAIMR | | | | | | | Mascot |

| Spot No. | Accession No. | Protein Name | | | | | | Protein PI | Protein MW | Protein Score | Protein Score / C. I. % | Total Ion Score | Total Ion / C. I. % | Peptide number |
|---|---|---|---|---|---|---|---|---|---|---|---|---|---|---|
| | 1017.551 | 1017.5524 | 0.0014 | 1 | 70 | 78 | VVQSNAIMR | | | 31 | 45.707 | | | Mascot |
| | 1037.5878 | 1037.5729 | -0.0149 | -14 | 19 | 27 | LLLEYTGTK | | | | | | | Mascot |
| | 1084.5059 | 1084.5121 | 0.0062 | 6 | 136 | 144 | QFSDFLGDR | | | | | | | Mascot |
| | 1212.6008 | 1212.6158 | 0.015 | 12 | 136 | 145 | QFSDFLGDRK | | | | | | | Mascot |
| | 1212.6008 | 1212.6158 | 0.015 | 12 | 136 | 145 | QFSDFLGDRK | | | 66 | 99.981 | | | Mascot |
| | 1365.7347 | 1365.7112 | -0.0235 | -17 | 1 | 11 | MAVKLAYWDIR | | | | | | | Mascot |
| | 1450.6996 | 1450.7412 | 0.0416 | 29 | 97 | 108 | VDILENQAMDFR | | | | | | | Mascot |
| | 1450.6996 | 1450.7412 | 0.0416 | 29 | 97 | 108 | VDILENQAMDFR | | | 72 | 99.995 | | | Mascot |
| | 1466.6945 | 1466.7526 | 0.0581 | 40 | 97 | 108 | VDILENQAMDFR | | | | | Oxidation | | Mascot |
| | 1531.7712 | 1531.7367 | -0.0345 | -23 | 187 | 198 | FESLEKIVEYMK | | | | | Oxidation | | Mascot |
| | 1586.8312 | 1586.886 | 0.0548 | 35 | 19 | 31 | LLLEYTGTKYEEK | | | | | | | Mascot |
| **Spot No.** | **Accession No.** | **Protein Name** | | | | | | **Protein PI** | **Protein MW** | **Protein Score** | **Protein Score / C. I. %** | **Total Ion Score** | **Total Ion / C. I. %** | **Peptide number** |
| 1889 | gi\|16940397 | glutathione | | | | | | 6.84 | 21922.2 | 237 | 100 | 175 | 100 | 8 |
| | **Peptide Information** | | | | | | | | | | | | | |
| | **Calc. Mass** | **Obsrv. Mass** | **± da** | **± ppm** | **Start Seq.** | **End Seq.** | **Sequence** | | | | **Ion Score / C. I. %** | **Modification** | | **Result Type** |
| | 843.4247 | 843.4305 | 0.0058 | 7 | 8 | 14 | FYDLSAK | | | | | | | Mascot |
| | 1152.532 | 1152.5438 | 0.0118 | 10 | 149 | 157 | NDISWNFEK | | | | | | | Mascot |
| | 1219.6357 | 1219.6398 | 0.0041 | 3 | 158 | 168 | FLIGPDGEPFK | | | | | | | Mascot |
| | 1263.6481 | 1263.6606 | 0.0125 | 10 | 89 | 99 | YVRPGNGFEPK | | | | | | | Mascot |
| | 1375.7369 | 1375.7471 | 0.0102 | 7 | 158 | 169 | FLIGPDGEPFKR | | | | | | | Mascot |
| | 1375.7369 | 1375.7471 | 0.0102 | 7 | 158 | 169 | FLIGPDGEPFKR | | | 90 | 100 | | | Mascot |
| | 1393.6166 | 1393.6389 | 0.0223 | 16 | 46 | 56 | DYTQMNELHSR | | | | | | | Mascot |
| | 1393.6166 | 1393.6389 | 0.0223 | 16 | 46 | 56 | DYTQMNELHSR | | | 85 | 100 | | | Mascot |
| | 1406.7889 | 1406.7888 | -0.0001 | 0 | 15 | 27 | LLSGDLLNFSSLK | | | | | | | Mascot |
| | 1409.6115 | 1409.6488 | 0.0373 | 26 | 46 | 56 | DYTQMNELHSR | | | | | Oxidation | | Mascot |
| | 1409.6115 | 1409.6488 | 0.0373 | 26 | 46 | 56 | DYTQMNELHSR | | | 20 | 0 | Oxidation | | Mascot |
| | 1591.9054 | 1591.86 | -0.0454 | -29 | 15 | 29 | LLSGDLLNFSSLKGK | | | | | | | Mascot |
| **Spot No.** | **Accession No.** | **Protein Name** | | | | | | **Protein PI** | **Protein MW** | **Protein Score** | **Protein Score / C. I. %** | **Total Ion Score** | **Total Ion / C. I. %** | **Peptide number** |
| 700 | gi\|60279651 | betaine-- | | | | | | 6.61 | 44641.3 | 339 | 100 | 272 | 100 | 11 |
| | **Peptide Information** | | | | | | | | | | | | | |
| | **Calc. Mass** | **Obsrv. Mass** | **± da** | **± ppm** | **Start Seq.** | **End Seq.** | **Sequence** | | | | **Ion Score / C. I. %** | **Modification** | | **Result Type** |
| | 896.4261 | 896.4385 | 0.0124 | 14 | 344 | 349 | RDYWEK | | | | | | | Mascot |
| | 907.4995 | 907.4943 | -0.0052 | -6 | 282 | 289 | EAYKAGIR | | | | | | | Mascot |
| | 976.5098 | 976.5145 | 0.0047 | 5 | 314 | 322 | GFLPEASQK | | | | | | | Mascot |
| | 990.5982 | 990.6112 | 0.013 | 13 | 139 | 146 | QLDVFIKK | | | | | | | Mascot |
| | 1098.6167 | 1098.6073 | -0.0094 | -9 | 55 | 62 | QLHREFLR | | | | | | | Mascot |
| | 1111.4989 | 1111.5206 | 0.0217 | 20 | 274 | 281 | WEMQQYAR | | | | | | | Mascot |
| | 1111.4989 | 1111.5206 | 0.0217 | 20 | 274 | 281 | WEMQQYAR | | | 57 | 99.871 | | | Mascot |
| | 1127.4939 | 1127.5114 | 0.0175 | 16 | 274 | 281 | WEMQQYAR | | | | | Oxidation | | Mascot |
| | 1594.8159 | 1594.8397 | 0.0238 | 15 | 270 | 281 | ILTRWEMQQYAR | | | | | | | Mascot |

| | | | | | | | | | | | | | | |
|---|---|---|---|---|---|---|---|---|---|---|---|---|---|---|
| | 1610.8108 | 1610.8231 | 0.0123 | 8 | 270 | 281 | ILTRWEMQQYAR | | | | | Oxidation | | Mascot |
| | 1788.8665 | 1788.8934 | 0.0269 | 15 | 38 | 54 | AGPWTPEAAAEHPEAVR | | | | | | | Mascot |
| | 1788.8665 | 1788.8934 | 0.0269 | 15 | 38 | 54 | AGPWTPEAAAEHPEAVR | | | 99 | 100 | | | Mascot |
| | 2091.1233 | 2090.9751 | -0.1482 | -71 | 14 | 33 | LNAGEVVIGDGGFVFALEKR | | | | | | | Mascot |
| | 2394.103 | 2394.1802 | 0.0772 | 32 | 63 | 83 | AGSNVMQTFTFYASDDKLENR | | | | | | | Mascot |
| | 2394.103 | 2394.1802 | 0.0772 | 32 | 63 | 83 | AGSNVMQTFTFYASDDKLENR | | | 116 | 100 | | | Mascot |
| | 2410.0979 | 2410.1355 | 0.0376 | 16 | 63 | 83 | AGSNVMQTFTFYASDDKLENR | | | | | Oxidation | | Mascot |
| | 2452.166 | 2452.144 | -0.022 | -9 | 104 | 127 | EVANEGDALVAGGVSQTPSYLSCK | | | | | Carbamidom | | Mascot |
| **Spot No.** | **Accession No.** | **Protein Name** | | | | | | **Protein PI** | **Protein MW** | **Protein Score** | **Protein Score / C. I. %** | **Total Ion Score** | **Total Ion / C. I. %** | **Peptide number** |
| 1199 | gi\|47085773 | aspartate | | | | | | 6.53 | 46238.3 | 197 | 100 | 173 | 100 | 7 |
| | **Peptide Information** | | | | | | | | | | | | | |
| | **Calc. Mass** | **Obsrv. Mass** | **± da** | **± ppm** | **Start Seq.** | **End Seq.** | **Sequence** | | | | **Ion Score / C. I. %** | **Modification** | | **Result Type** |
| | 805.4566 | 805.4532 | -0.0034 | -4 | 112 | 118 | IGAEFLR | | | | | | | Mascot |
| | 948.5261 | 948.5455 | 0.0194 | 20 | 31 | 39 | VNLGVGAYR | | | | | | | Mascot |
| | 961.5577 | 961.5517 | -0.006 | -6 | 112 | 119 | IGAEFLRR | | | | | | | Mascot |
| | 1012.4846 | 1012.5068 | 0.0222 | 22 | 257 | 264 | NFGLYNER | | | | | | | Mascot |
| | 1012.4846 | 1012.5068 | 0.0222 | 22 | 257 | 264 | NFGLYNER | | | 58 | 99.922 | | | Mascot |
| | 1254.5862 | 1254.6389 | 0.0527 | 42 | 119 | 128 | RWYNGTDNTK | | | | | | | Mascot |
| | 1313.6597 | 1313.676 | 0.0163 | 12 | 291 | 302 | ITWSNPPSQGAR | | | | | | | Mascot |
| | 1313.6597 | 1313.676 | 0.0163 | 12 | 291 | 302 | ITWSNPPSQGAR | | | 63 | 99.972 | | | Mascot |
| | 1598.7996 | 1598.8199 | 0.0203 | 13 | 40 | 52 | TDECQPWVLPVVR | | | | | Carbamidom | | Mascot |
| | 1598.7996 | 1598.8199 | 0.0203 | 13 | 40 | 52 | TDECQPWVLPVVR | | | 51 | 99.589 | Carbamidom | | Mascot |
| **Spot No.** | **Accession No.** | **Protein Name** | | | | | | **Protein PI** | **Protein MW** | **Protein Score** | **Protein Score / C. I. %** | **Total Ion Score** | **Total Ion / C. I. %** | **Peptide number** |
| 1591 | gi\|41386743 | eukaryotic | | | | | | 6.28 | 96463.2 | 218 | 100 | 169 | 100 | 14 |
| | **Peptide Information** | | | | | | | | | | | | | |
| | **Calc. Mass** | **Obsrv. Mass** | **± da** | **± ppm** | **Start Seq.** | **End Seq.** | **Sequence** | | | | **Ion Score / C. I. %** | **Modification** | | **Result Type** |
| | 814.5145 | 814.5267 | 0.0122 | 15 | 520 | 526 | LVEGLKR | | | | | | | Mascot |
| | 887.4581 | 887.4653 | 0.0072 | 8 | 276 | 284 | TANGPDGKK | | | | | | | Mascot |
| | 969.5476 | 969.6081 | 0.0605 | 62 | 717 | 726 | GGGQIIPTAR | | | | | | | Mascot |
| | 1125.6488 | 1125.6273 | -0.0215 | -19 | 717 | 727 | GGGQIIPTARR | | | | | | | Mascot |
| | 1125.6488 | 1125.6273 | -0.0215 | -19 | 717 | 727 | GGGQIIPTARR | | | | | | | Mascot |
| | 1323.6475 | 1323.6974 | 0.0499 | 38 | 21 | 32 | NMSVIAHVDHGK | | | | | Oxidation | | Mascot |
| | 1420.7253 | 1420.7706 | 0.0453 | 32 | 728 | 739 | VLYACQLTAEPR | | | | | Carbamidom | | Mascot |
| | 1420.7253 | 1420.7706 | 0.0453 | 32 | 728 | 739 | VLYACQLTAEPR | | | 57 | 99.875 | Carbamidom | | Mascot |
| | 1567.7784 | 1567.7758 | -0.0026 | -2 | 560 | 572 | DLEEDHACIPLKK | | | | | Carbamidom | | Mascot |
| | 1576.8264 | 1576.8851 | 0.0587 | 37 | 727 | 739 | RVLYACQLTAEPR | | | | | Carbamidom | | Mascot |
| | 1576.8264 | 1576.8851 | 0.0587 | 37 | 727 | 739 | RVLYACQLTAEPR | | | 25 | 0 | Carbamidom | | Mascot |
| | 1629.8846 | 1629.8556 | -0.029 | -18 | 843 | 857 | GLKEGIPALDNFLDK | | | | | | | Mascot |
| | 1649.868 | 1649.8624 | -0.0056 | -3 | 2 | 15 | VNFTVDQIRAIMDK | | | | | | | Mascot |
| | 1757.9764 | 1757.9446 | -0.0318 | -18 | 145 | 159 | QAIAERIKPVLMMNK | | | | | Oxidation | | Mascot |

| | | | | | | | | | | | | | | |
|---|---|---|---|---|---|---|---|---|---|---|---|---|---|---|
| | 1772.849 | 1772.9197 | 0.0707 | 40 | 634 | 647 | YLADKYEWEVTEAR | | | | | | | Mascot |
| | 1772.849 | 1772.9197 | 0.0707 | 40 | 634 | 647 | YLADKYEWEVTEAR | | | 88 | 100 | | | Mascot |
| | 1777.9126 | 1777.9698 | 0.0572 | 32 | 17 | 32 | SNIRNMSVIAHVDHGK | | | | | | | Mascot |
| | 1982.9424 | 1983.1217 | 0.1793 | 90 | 482 | 498 | TGTITTFDQAHNMRVMK | | | | | Oxidation | | Mascot |
| **Spot No.** | **Accession No.** | **Protein Name** | | | | | | **Protein PI** | **Protein MW** | **Protein Score** | **Protein Score / C. I. %** | **Total Ion Score** | **Total Ion / C. I. %** | **Peptide number** |
| 1595 | gi\|41386743 | eukaryotic | | | | | | 6.28 | 96463.2 | 230 | 100 | 188 | 100 | 12 |
| | **Peptide Information** | | | | | | | | | | | | | |
| | **Calc. Mass** | **Obsrv. Mass** | **± da** | **± ppm** | **Start Seq.** | **End Seq.** | **Sequence** | | | | **Ion Score / C. I. %** | **Modification** | | **Result Type** |
| | 969.5476 | 969.5629 | 0.0153 | 16 | 717 | 726 | GGGQIIPTAR | | | | | | | Mascot |
| | 1086.5288 | 1086.5269 | -0.0019 | -2 | 227 | 235 | QFAEMYVAK | | | | | | | Mascot |
| | 1420.7253 | 1420.7726 | 0.0473 | 33 | 728 | 739 | VLYACQLTAEPR | | | | | Carbamidom | | Mascot |
| | 1420.7253 | 1420.7726 | 0.0473 | 33 | 728 | 739 | VLYACQLTAEPR | | | 80 | 100 | Carbamidom | | Mascot |
| | 1487.858 | 1487.7438 | -0.1142 | -77 | 499 | 512 | FSVSPVVRVAVEAK | | | | | | | Mascot |
| | 1567.7784 | 1567.7322 | -0.0462 | -29 | 560 | 572 | DLEEDHACIPLKK | | | | | Carbamidom | | Mascot |
| | 1576.8264 | 1576.8636 | 0.0372 | 24 | 727 | 739 | RVLYACQLTAEPR | | | | | Carbamidom | | Mascot |
| | 1576.8264 | 1576.8636 | 0.0372 | 24 | 727 | 739 | RVLYACQLTAEPR | | | 21 | 0 | Carbamidom | | Mascot |
| | 1629.8846 | 1629.9265 | 0.0419 | 26 | 843 | 857 | GLKEGIPALDNFLDK | | | | | | | Mascot |
| | 1649.868 | 1649.8584 | -0.0096 | -6 | 2 | 15 | VNFTVDQIRAIMDK | | | | | | | Mascot |
| | 1757.9764 | 1757.9105 | -0.0659 | -37 | 145 | 159 | QAIAERIKPVLMMNK | | | | | Oxidation | | Mascot |
| | 1772.849 | 1772.9185 | 0.0695 | 39 | 634 | 647 | YLADKYEWEVTEAR | | | | | | | Mascot |
| | 1772.849 | 1772.9185 | 0.0695 | 39 | 634 | 647 | YLADKYEWEVTEAR | | | 87 | 100 | | | Mascot |
| | 1966.9474 | 1967.0856 | 0.1382 | 70 | 482 | 498 | TGTITTFDQAHNMRVMK | | | | | Oxidation | | Mascot |
| | 2235.0938 | 2235.2009 | 0.1071 | 48 | 648 | 667 | KIWCFGPDGTGPNMLVDVTK | | | | | Carbamidom | | Mascot |

**Supplementary material 4**

**The gene sequences of the antioxidant metabolism related proteins that subjected to mRNA expression analysis are shown in below.**

**1. *β-actin***

**>gi|33772046|gb|AY170122.2| beta-actin gene, complete cds [*Megalobrama amblycephala*]**

CTGTTGTTGTTTAGAACTGCCATTGAAGGCCATGTGCATTACTCCCAAGTTACACATTAA
AAAGTCACTGTAGCTTGCCAGCATCATGTCTGCTCAAGCACAGCCTTATCTATTATTCAA
ACTGTGGCAACATCTGAAATATGCAAGAAATGTTTTTAAAGGGCTAGTTCACCCAAAA
ATGAAAATTATGTCATTAATTACACACCCTCATGTCGTTCTACACCTGTAAGACCTTCGT
TCATCTTCAGAACACAAATTAAGATATTTTTGATGAAATCCGATGGCTCCGTGAGGCCT
GCATTGACAGCAAAGCCTTTCAAACTCTCAAGGTCCATAAAGGTACTAAAACATATTCA
AAACAGTTCATGCGAGTACAGTGGTTCTACCTTACGAGAATACTTTCTGTGCACAAAAA
AACCCAAAATAATGACTTTTAACAAGATCTAGTGATGGCCGATTTCAAATCACTGCTTC
TTGAAGCTACGGAGCTTTTCGAATCAAATCAGTGATCGGAGCGCCAAAGTCACGTGCTT
TCAGCAGTCTAATACGCGATCCGAATCACTGATTCGACTCAAAAGATACGTAAAGCTCC
GAAGCTTCAAGAAGCAGCGATTTGAAATCGGCCATCACTGGATATTGTTGAAAAGTCCT
TTTTGGGGTTTTTTGGCACACAAAAAATATTCCATTGGCTTTATAATATTAAGATAGAAC
CACTGAACTCACATGAACTGATTTAAATATGTTTTTAGTACCTTTACGGATCTTGAGAGA
GGAAGTACCGTTGTGGTCTATGGAGACCTCACTGAGCCATCGGATTTCATCAAAAATAT
CTCAATTTGTTTTTCTGAAGATGAACGAAGGTCTTACAGGTGTGAAACCACATGAGGGT
GAGTAATAAATGACATAATTTTCATTTTTGGGTGAACTAACCCTTTAATGAAGATAACC
ATGAATATCTTCACCGTTATTTTTTTGACAAAGGAAACAATGTTTTGAAGATTTATCATT
ATTATAAATCTCTCCAAACAGTAAGTCAAAGTTCATATGTCTGTATGACGATACTTCAAT
AATGTGTAGTATGTTTTTGAATGAGATACTAGACTTGCCTATGAAACATCTACAACAAG
ATATTTAATTGGTCAAGACCAAGACTTGAATGCTGACATTTGGGCTAGGCCCACTTACA
GCCATCAACAACTACTATCCCTTTATCATCACCACCAAGTAACATGCACACACCTATGC
GTGTAAAAATATTACTCCTGTAAATGAGTGATTCATAATTACAGGAATGCAAACCCCAT
TCATTCCAAGTTTCATTACAATGTCTTTGACAACATTACCTAATGAGCTTAGACCAGTTA
GTTGCTCTTACAGGAAACGGGTCATTTAACTTCAGTTATCATGACTTCAATGAAAAATTA
CCCCTATTTTGCATAGAGAAGTACTTGGTAGATTCCTACACAGAAATATTATGGTATTAT
TGTGAATACAATAAAAGGTAAATGACCTACAGAGCTGCTGCTGTGTTAAATTGTAAACA
CAACACAGGACCAAGGAGGTGTCCACCACTACGACCAATACTGGCACTTGTAACCTTTC
ACAGACTTTTTAAAAGGTTGAAGAGTCTTATTGGTGCTATCGCAACTAAATACGTAATT
ACGAAACACAATGTATTAAAATTCGCAAATAACAGGGTAAAATTTGATCAAAATCACC
GAGGCCTTGTTCTTCAACTAGTCTAGCTTCCCCTTCTTTCACTCTCAAGTTGCAAGAAAG
CAAGTGTAGCAATGTGCACGCGACAGCCGGGTGTGTGACGCTGGACCAATCAGAGCGC
AGAGCTCCGAAAGTTTACCTTTTATGGCTAGAGCCGGGCATATGCCGTCATATAAAAGA
GCTCGCCCAGCCTTTCAACCTCACTTTGAGCTCCTCCACACGCAGCTAGTGCGGAATATC

ATCAGCTTGTAACCCATTCTCTTAAGTCGACAAATCCCCCAAACCTAAGGTGAGTTTATT
TTTCAGCTTTTATTGCATTTATTAGTTCATTAATATTAATTTAAACATGTAACAATGATGT
ATAAATAAAACTGGATGAAGAAATTAGACTAAGTTACCGGTCTTTTCACTTTTAAGTATT
AACGCCTGCTTAAACTGTTGCTATTCATTAATTTGTAGTTATTTTTATGTAAATTTTTGCT
ATATTATCACAACTTTATATACTGTGCTGGACATGTCAGGTGGAAATGATGGTATCCGTT
GTAGACACGACATTGAATGGGCCGGTGTAAAATAAGAGTTCGCCTTTCTTAACATTTAA
GATGTGCTCAGATTAACCGTGTTTTAACGGCTATAAACTTGACTCGACAGTTTCAAGGAT
GTATATTTGTGAGGCATGTTACACACTTGCTGGATAGCCGGCATGGGAAGTTCTTTGTGC
AGGCAGTGCTGCAGAAGGGTGTGACCTACTTTAGCTAACCGGCTAACTTAACCAGCGTT
CACCTTCTGTAAACCTGGTGAATCTAACTCACTTATTCGATCACTAAACCATCAAAGATG
AGTTTTTCGTTCACTTTCAGCACATAAAACATTTTTAGTGAATACTGGCGCTTTATAATG
GCGCATGCAGCCTTTCAGTCTTCCCTTAAAACTGACGATTAGTCAAGTAACAGTTATTTC
ATTCACTATTAAAGCGTTTCGAAATATAAGGTGTGGTCAATTTTAATAACTGGCGTAAA
GCGTTCCGGATGCATTTGAACTCCTATAATTGTGTCGAACAGTAATTTTTCTTAAAACTA
ATTGATAAAGACTAGAATATAAAAGTGAATGGGCTGACTGTGATCTCTCACAAGTGCTT
TTGCACAAGTCTGCTCTTATGGAGTCATTTGAAGTGACTGCAGATCTGTGACGCAGTAAT
GTTGGGCAGACACCCGTCGAAATTCGGTTGCGTAATTGATACCAGGCGAGGATGAAAG
AGGATGTAAAACTTCATTCGTGTAGAATTTTTAGGGAGTGGCCCTGGCGTGATGAATGT
CGAAATCTGTTCCTTTTTACTGAACCATACGACTCTGGCTGAGTGCCACCCCGCCGGCAG
CCGCAAAGCGGCTCAATCCATTGCCTTTTATGGTAATAATGAGAGAATGCAGAGGGACT
TCCTTTGTCTGGCATATCTGAGGCGCGCATTGTCACTCTAGCACCCACTAGCGGTCAGAC
TGTAGAATGCAGCATGAAACAGGAAGTTGACTCCACATGGTCACATGCTCGCTGACACT
TTCTTACATGGCAGCAGTGCACTTCAAAAACACACTTCACTCTGTTTTACAGTTCAGCCA
TGGATGATGAAATTGCCGCACTGGTTGTTGACAACGGATCCGGTATGTGCAAAGCTGGA
TTCGCTGGAGATGATGCTCCCCGTGCTGTCTTCCCATCCATTGTGGGTCGCCCCAGACAT
CAGGTGAGAGATGGAGGATAAACGATTTTGGGCTGACTGATTTTAATATGAATTTTCAT
GCTTTTATTCTCTAGTTCCTGAACATTTTACAAAAATTAACATGCTTTTCTTCGTTACAGG
GCGTCATGGTCGGTATGGGACAGAAGGACAGCTATGTTGGTGACGAGGCTCAGAGCAA
GAGGGGTATCCTGACCCTGAAGTACCCCATCGAGCACGGTATTGTCACCAACTGGGACG
ATATGGAGAAGATCTGGCATCACACCTTCTACAACGAGCTGCGTGTTGCCCCAGAGGAG
CACCCCGTCCTGCTCACAGAGGCCCCCCTGAACCCCAAAGCCAACAGGGAAAAGATGA
CACAGGTGGGTTTTTGGCTAGCAAATGGTGCTTTGAAGTCTCTTGTCTGTCCTGTTACCT
CATTTAAGTTCTCCTCTTCATTCGTTCACTTCCTCCAGGCTTTGTTTCCTCTGAGCTCCTG
AGTTTCTCATCTTTTGCTGGAAGCAGCAGGTTATCTATACTTTTGCCTGCCTGTTTTGCAG
TCTCCTCTGCACTCTGATTCTTTGTGCACTTTTGTTTCTTTACTCTAGATTTTCAACTAACC
CCTGCATGGGTGTGTGGATGATGTGCTGTAACTTTTTGAGCATTGGTTAACTTCTCCTCT
CTCGTTACAGATCATGTTCGAGACCTTCAACACCCCTGCCATGTACGTTGCCATCCAGGC
TGTGCTGTCCCTGTATGCCTCTGGTCGTACCACTGGTATCGTGATGGACTCTGGTGATGG
TGTCACCCACACTGTGCCCATCTACGAGGGTTATGCCCTGCCCCATGCCATCCTCCGTCT
GGACTTGGCTGGCCGTGACCTGACTGACTACCTCATGAAGATCCTCACCGAGAGAGGCT
ACAGCTTCACCACCACAGCTGAGAGGGAAATTGTCCGTGACATCAAGGAGAAGCTCTG
CTATGTGGCTCTTGACTTCGAGCAGGAGATGGGCACCGCTGCTTCCTCCTCCTCCCTGGA

GAAGAGCTATGAGCTGCCTGACGGACAGGTCATCACCATTGGCAATGAGAGGTTCAGG
TGCCCAGAGGCTCTGTTCCAGCCATCCTTCTTGGGTAGGTTTCCTGACAAACGTTACCTG
GTGTGTGTACTCTAGATTTGATTTGAAGGAGAATTGAAGAACCAAGGTTAACCTTTTTTT
CTCTTGCTCTGCAGGTATGGAGTCTTGCGGTATCCATGAGACCACCTTCAACTCCATCAT
GAAGTGTGATGTCGACATCCGTAAGGACCTGTATGCCAACACTGTATTGTCTGGTGGTA
CCACCATGTACCCTGGCATTGCTGACAGGATGCAGAAGGAGATCACATCCCTGGCCCCT
AGCACAATGAAAATCAAGGTGAGCTGTGGTCTGAACTTTGACCCTTAACACTCATATCA
GTCTGTAACTGAATGCATATGCAACTCGTGCATTGTGCTAATCATTTGTTTCTCCACAGA
TCATCGCCCCACCTGAGCGTAAATACTCTGTCTGGATCGGAGGTTCCATCCTGGCCTCCC
TGTCCACCTTCCAGCAGATGTGGATTAGCAAGCAGGAGTACGACGAGTCTGGACCATCC
ATCGTCCACCGCAAATGCTTCTAAACGGACTGCTACCACTTCACGCCGACTCAAACTGC
GCAGAGAAAAACTTCAAACGACAACATTGGCATGGCTTTTGTTATTTTTGGCGCTTGACT
CAGGATCTAAAAACTGGAACGGTGAAGGTGACGGCAATGTTTTTTGGCAAATAAGCATC
CCCGAAGTTCTACAATGCATCTGAGGACTCAATGGTTTTTGTTTTGTTTCTTTAGTCATTC
CAAATGTTTGTTAAATGCATTGTTCCGAAACTTATTTGCCTCTATGAAGGCTGCCCAGTA
ATTGGGAGCATACTTAACATTGTAGTATTGTATGTAAATTATGTAACAAAACAACGTCT
GGGTTTTGTACTTTTCAGCCTTAAAATCTTGGGTTATTTTTTTTCTTTTTTTTTTTTTTTT
TTTTTTTTTTTTCTTTGTTCCAAAAAACTAAGCTTTACCATTCAAGATGTAAAGGTATCCA
TTCTCCCCCTGGGCATATTGTAAAAGCTGTGTGGAACGTGGCGGTGCCAGACATTTGGT
GGGGCCAACCTGTACACTGACTAATTCAATTCCAATAAAAGTGCACATGTGTAAGACAT
CATATTCCTGTGTGACCTCCTGTGTTGGTGCTGAATGAACTTGAGTAGAAGTATATTATA
TTTAATAAAACTAGTCTTTGATCTTCTTCGTGTTGTAATTACACTTTATTGGAGTATGCTC
TTCAGTTCCAGAGCACTTGGGTGAAAGAAGATAAATGGGTTTTCTTGACCTGATCAAAC
AGGGTTATCAGGGTGTTGTAGGTGGCTTTAACAAACTCAAATCTTTCAATTTGAGCTTAA
AATGGTGTACATGGCAAATAACTTGCTCTTAAATGCTTTCAAATGCTCAAACTAACACA
AATTGGGCTTTTATCAAGCTTATTTACATGTTTCCTTCATAAATGTAGCTTCAAACTGCTT
TTAATTGGCCTTCACTGTTTACATGGGATAAAACCACACCAAAGCTTTGAGAACACCCT
TTAAAAATGGAGGTCAATGTCAGTTTTATGCTTGTGCATATAAACTCCAATATCAAGAC
ATTTAATGCCAGCGTTACTGTGGGAAATTTTAAGCATATGGGGAAAGTAGCCAAGTTTT
AATCAACAGGTGAAGACTATAATTTTTACGGAGCTAAGTTAAATTAAGTTTATTAATCT
GTTCATTGCGTCTTTAGTGAGCTGTTGTGAGTGAATACAAATTTATATTTGTGCCTTTAG
ACTGAAGTTTGAACGTTATGAGAGAGCATTATGCCGCCTCATGGTCAGATGGTGTTATA
TTTTAATTTGCACTTCACGTAACTGTACAGCAGATGGCGCTACATACACATGAAACGAA
ATTCTTGATGCTTTGATTTGAGGAAACATGAACACAGACTCATAGTAACTCAATCAAGG
TGCAAGAGACTGTGTTTTCTCTTGGGAAGAGTACAAAAAATTCAGTAAGTGACATGAAA
TAGAATAACAAAACGTGGCACATATCCAATTTGTGCTAACAATATACACTAATAACTGG
CTGCTGTTTTTAGGTTGATCCTGGTGAGCTTGAACTTTGGTTATTGATGTAGTAGATACT
TATAAAGATGGGTGCCCTAGTTGCATATGATTTACTGCAAGCTGCTTCTCGTCTGACAGC
ACTTGATGAAAAATGATCAAGAGATTTTCCTGCTGTGTACATCTGTGCATGTGTAGAGT
AGCCTATAGTACAGATGTACTTTATTAACAG

**2.** ***ALDOB***

**>gi|42734425|ref|NP_919348.3| fructose-bisphosphate aldolase B [*Danio rerio*]**

AGAATAGATGAAGTGATCTGGCCCCGAATCATTAAAATCCATCTGCGTCAGTGAGGAGA
GGCCAGATCACTTCATCTATTCTTCATTCATAGTGCCACCAACCTACAGAGCAATCAAC
ATGACACACCAGTTTCCAGCTCTCTCTACGGAGCAGAAGAAGGAGCTTTCCACAATTGC
CCAGCGTATTGTGGCAACTGGAAAAGGCATCTTGGCTGCAGATGAGTCCACAGGCACCA
TGGCAAATCGCTTTCAGAAGATAAATGTGGAGAACACCGAAGAGAACCGTCGTAGCTTT
CGTGACCTCCTCTTCTCTGTAGACAATTCTATCTCTGAAAGTATTGGCGGTGTCATTTTTT
TCCACGAAACACTGTACCAGAAATCAGACAAAGGAGTTCTGTTTCCAAAAGTCGTCAAG
GACAAGGGCATCGTAGTTGGTATCAAGGTGGACAAAGGCACAGCGGGCCTGGGCGGTA
CAAATGGAGAGACAACCACACAAGGATTGGATGGTCTTTCTGAACGCTGTGCCCAGTAC
AAAAAGGACGGTTGTGACTTTGCCAAGTGGCGTTGTGTACTTAAGATCTCTGACGGCTG
CCCCTCTGCTCTTGCAATTGCTGAAAACGCTAACGTTCTTGCCAGATATGCCAGCATTTG
CCAACAGAATGGCTTGGTTCCCATTGTAGAGCCTGAGATCCTCCCAGACGGAGATCATG
ATCTGCAACGGTGCCAGTATGCCACTGAGAAGGTCCTGGCGGCTGTGTACAAAGCTCTC
TCTGACCACCATGTGTATCTGGAGGGAACTCTGCTCAAACCAAACATGGTCACTGCTGG
ACACTCCTGCACCAAGAAGTACACCCCCCAGGAGGTCGCCATGGCAACAGTTACTGCTC
TCAGACGCACTGTGCCAGCTGCTGTACCTGGCATCTGCTTCCTCTCTGGTGGTCAAAGTG
AGGAGGAGGCCTCTCTGAATCTGAATGCCATGAACCAGCTTCCCCTGCACAGACCCTGG
AAGCTGAGCTTCTCTTATGGCCGTGCTCTCCAGGCCTCGGCTCTTGCTGCATGGAAGGG
ACAAGCAGCGAACAAGAAGGCTGCACAGGATGCCTTTGTCACACGTGCCAAGATCAAT
AGTCTTGCATCACAAGGCGAATACAAACCCTCAGGCCAGGCTGACCAAGCATCTAAAC
AGTCCCTCTTTACTGCCAGCTATGTCTACTAAATCCAACAACAAACAACAGCAACTTGTT
CTGGACAATACAAAATTTACATGCAAACATACCAAAAGATATTGAAGAAGATCTGACA
AATAGGGGTTGCACAGACTAGTCGACTTTAATGCTCTCCCATGACGCTTTAAGCTTGAC
GTCGACTTGTCGCTGATCATGGGGCGCAACAGGAACAGGATCTTTGAAGTCCGCTTTTA
CACTCTGTTTCAAACAGTTAAAATGACGAATAAATGCATACAACATGTTTGATTATACC
ATCTGGAGCTTATTGTACATCTAAGTTAATCACCGTTCTAAATGCACTGCTGCACTGTAA
TGCACACAGACAGACAGCTCGCACATCACGCGCAGATCGTACACACAGAAAGTAGCGC
AGATATGTATTGTCAACACTGTTCTGTGATTTAGCAATAAAATAGCTTAGAAACTGAAA
TGTTCTAGCATATATTTCTTAGTAACTAAAACATACATTCATATAAATGTCATTTTTAAT
GCGCTACTTTATCTGTATATGATTTAGAAGGAGCAGAAACCTGTGAGAAATATAACAAC
CCAAAGACAAAAGAACGGATAAAAATGATTATGTAGGTGCAATAGCCTTGTAGGCAGC
GTTGTGTCATATGCCATAACTGTACACAAGGCAACCCACGTTCGAGTCAGATCCATCCA
TCTACTCGAGGTTCAGGCTTATTTGTTAATTAATGACATCATCAGCGACTAGTTGACGTC
GACTTAAAAACATCTGAAGTTGTTTAACCCCTACTGACAACACATTAATGTGTACACTT
GTTAATTGTACTAAAAGGAATGTTCTACCAAGATGACTCAAACAGACCCTGGATTAGTT
TCATTTTTAATTCATGGCACTAACTAATGCTTTTCATTTTGAATAAATCATACATTTTATT
TGAGTTGATTTCTTCTGTAAACATTAAGGTGGGAGTTGTTTAGTCAACCATTTTGTTGTG
CTCTACTTTACTAAGAACCAACATGTAAATTGACATGTTTAAATGTTTAAAACCTAAAA
AGTAATGTGCCTTTGCATCTGATGTCAACTGGTCTCTCTGACTTCCACAGCAGACTGAAA

AAAAGATGCTGAAGGGTCAGTTTTCAATAAGGTTGAAGAGCTTCAAAATGTCATGAAGG
TTTTTCTCTCTTGCTTCATCTGCTGCTGTCCTTTCCCAAACATCAGCAATCTGATAGGGCA
GAGACAAATTATACATGTTACAGGTTGAGAGTTATGGCTTATTCTCTCTTAGGTTCATTT
CGACCCGAAATTCATTTTCATTTTTAAAAATTTAGTTTTCATCGGGGTGGTCCAAGACCT
TAGGGCAGTTGTCACATTTGAACTTGAAATACACACACACACACACACACACA

**3. *GAPDH***

**>gi|169403946|ref|NM_001115114.1| glyceraldehyde-3-phosphate dehydrogenase (gapdh), mRNA [*Danio rerio*]**

ACTCACACCAAGTGTCAGGACGAACAGAGGCTTCTCACAAACGAGGACACAACCAAAT
CAGGCATAATGGTTAAAGTTGGTATTAACGGATTCGGTCGCATTGGCCGTCTGGTGACC
CGTGCTGCTTTCTTGACCAAGAAAGTGGAGATCGTGGCCATCAATGACCCATTCATTGA
CCTTGATTACATGGTTTACATGTTCCAGTACGACTCCACCCATGGAAAGTACAAGGGTG
AGGTTAAGGCAGAAGGCGGCAAACTGGTCATTGATGGTCATGCAATCACAGTCTATAGC
GAGAGGGACCCAGCCAACATTAAGTGGGGTGATGCAGGTGCTACTTATGTTGTGGAGTC
TACTGGTGTCTTCACTACTATTGAGAAGGCTTCTGCTCACATTAAGGGTGGTGCAAAGA
GAGTCATCATCTCTGCCCCAAGTGCAGATGCCCCCATGTTTGTCATGGGTGTCAACCATG
AGAAATATGACAACTCTCTCACAGTTGTAAGCAATGCCTCCTGCACCACCAACTGCCTG
GCTCCTTTGGCAAAGGTCATCAATGATAACTTTGTCATCGTTGAAGGTCTTATGAGCACT
GTTCATGCCATCACAGCAACACAGAAGACCGTTGATGGGCCCTCTGGGAAGCTGTGGAG
GGATGGCCGTGGTGCCAGTCAGAACATCATCCCAGCCTCCACTGGGGCTGCCAAGGCTG
TAGGCAAAGTAATTCCTGAGCTCAATGGCAAGCTTACTGGTATGGCCTTCCGTGTCCCC
ACCCCCAATGTCTCTGTTGTGGATCTGACAGTCCGTCTTGAGAAACCTGCCAAGTATGAT
GAGATCAAGAAAGTCGTCAAGGCTGCAGCTGATGGGCCCATGAAAGGAATTCTGGGAT
ACACGGAGCACCAGGTTGTGTCCACTGACTTCAATGGGGATTGCCGTTCATCCATCTTTG
ACGCTGGTGCTGGTATTGCTCTCAACGATCACTTTGTCAAGCTGGTCACATGGTATGACA
ATGAGTTCGGTTACAGCAACCGTGTATGTGACCTGATGGCACACATGGCCTCCAAGGAG
TAGATGTGACCCCTTTGCTGTTTCTTTTTTTTGATACGCGACCATTCTCCCATCTGGTTGA
ATGTTTGCACCACGTGCCTGGAAGGAAATTACATGCTTAAATTGAAGACCAATATTATT
TTTATATACTCTGTTCTGTTTCGTGTGTGAGGTTAAAAATAAATGTTGACTTCAAAGGCT
TTTCTGTCTGTTAACAACTTGCGATGGAATAAAAGTCCTCTGTTTGTGAGAAATGAAAA
AAAAAAAAAAAAAAAAAAAAAAAAA

**4. *GPx1***

**>gi|226425217|gb|ACO53608.1| glutathione peroxidase [*Hypophthalmichthys nobilis*]**

AGGCACGAGGGGGAATATTATCTCACGTGACAGAATGAGAAAAATGTGGAAGTGAAAA
TGTGAGGCTAAGGTTTGTCATATGAACCGCTTCTGTTTTTTTATCAGTTTTGTAAATTGT
GAAATAAGGAACAATAGGATTTTTTTTTTAAATTTTTATTTTCAGTTAACAGTTGAAAGA
CAAAACCTGAGCCGTGCAATTCATGGATAAAACACAACTATTATAATAAACCTTTACAG
CTACATCAGAAACTTCCCTCAGAAAGGTTTTAGGAAGCATCTTCATTAAGTAAAATGTC
TAAAAAACAGTCGCACTTTAATCATGTCTGCACAGTCGGCTTATATCAAACAACAATCT
TATGGCTTCACGTGATATGCAGTGGACAGACTATCTAGCGTGGCCAAACACGAGAAGGC
CGCCTGCCAGGTTTACTTCGTCCTCTTGAGAAGCTCTTTGATATCTGCTTCAATGTCGCT
GGTGAGGAACCTTCTGCTGTACCGCTTGAACGGTTCTCCGTCCGGGCCGATGAGGAACT
TCTCAAAGTTCCAGGCGATGTCATTCCGGTTCACGGGACTCCAGATGATGAGTTTGGGA
TCACCCATCAGGGACACAGCGTCGTCACTGGGCTGAGGCAGCTTCTCTTTCAGGAACAC
AAACAGAGGGTGGGCGTTCTCACCGTTCACTTCTAGCTTCTCCAGAAGCTGGAATTTGG
GCTCGAAGCCATTTCCAGGACGGACGTACTTCAGAGACTTCAGGATTTCATCATTCTTGC
AGTTCTCCTGATGTCCGAACTGGTTGCAGGGAGCGCCCAGAATAACCAGCCCCTGATCA
GCATATCGACTGTGGAGCTCGTTCATCTGAGTGTAATCCCTGACTGTTGTGCCTCAGAGC
GACGCCACATTTTCAATAAGCACAACTTTCCCTTTGAGAGACGAAAAATTCAGGAGGTC
CCCTGACAAAAGCTTGGCGGACAGATCATAAAACTTCTTCATGGTCCCTGTCATGCTCG
AACACGCAGTTTACTCTGTAGTTAGAAGCGCAAAATGCTTTTTGTTTTATCGACAGACCT
TGAGTTGAACCTTGAGTCGAAGGGGAGGGAACTGCGTAAATGCAGTTCAAGGAAGTGT
GCTCTCGTGCCAGGGCAGCAGGAGGTAAACACTGTTAAACACCGCCATCTTTTGTCAAA
CGACGATATTCTAACCCCTGCATAAT

**Reverse Complement**

ATTATGCAGGGGTTAGAATATCGTCGTTTGACAAAAGATGGCGGTGTTTAACAGTGTTT
ACCTCCTGCTGCCCTGGCACGAGAGCACACTTCCTTGAACTGCATTTACGCAGTTCCCTC
CCCTTCGACTCAAGGTTCAACTCAAGGTCTGTCGATAAAACAAAAAGCATTTTGCGCTT
CTAACTACAGAGTAAACTGCGTGTTCGAGCATGACAGGGACCATGAAGAAGTTTTATGA
TCTGTCCGCCAAGCTTTTGTCAGGGGACCTCCTGAATTTTTCGTCTCTCAAAGGGAAAGT
TGTGCTTATTGAAAATGTGGCGTCGCTCTGAGGCACAACAGTCAGGGATTACACTCAGA
TGAACGAGCTCCACAGTCGATATGCTGATCAGGGGCTGGTTATTCTGGGCGCTCCCTGC
AACCAGTTCGGACATCAGGAGAACTGCAAGAATGATGAAATCCTGAAGTCTCTGAAGT
ACGTCCGTCCTGGAAATGGCTTCGAGCCCAAATTCCAGCTTCTGGAGAAGCTAGAAGTG
AACGGTGAGAACGCCCACCCTCTGTTTGTGTTCCTGAAAGAGAAGCTGCCTCAGCCCAG
TGACGACGCTGTGTCCCTGATGGGTGATCCCAAACTCATCATCTGGAGTCCCGTGAACC
GGAATGACATCGCCTGGAACTTTGAGAAGTTCCTCATCGGCCCGGACGGAGAACCGTTC
AAGCGGTACAGCAGAAGGTTCCTCACCAGCGACATTGAAGCAGATATCAAAGAGCTTC
TCAAGAGGACGAAGTAAACCTGGCAGGCGGCCTTCTCGTGTTTGGCCACGCTAGATAGT
CTGTCCACTGCATATCACGTGAAGCCATAAGATTGTTGTTTGATATAAGCCGACTGTGC
AGACATGATTAAAGTGCGACTGTTTTTTAGACATTTTACTTAATGAAGATGCTTCCTAAA

ACCTTTCTGAGGGAAGTTTCTGATGTAGCTGTAAAGGTTTATTATAATAGTTGTGTTTTA
TCCATGAATTGCACGGCTCAGGTTTTGTCTTTCAACTGTTAACTGAAAATAAAAATTTAA
AAAAAAAATCCTATTGTTCCTTATTTCACAATTTACAAAACTGATAAAAAAAAACAGAAG
CGGTTCATATGACAAACCTTAGCCTCACATTTTCACTTCCACATTTTTCTCATTCTGTCAC
GTGAGATAATATTCCCCCTCGTGCCT

**5. *GSTm***

**>gi|220679252|emb|CAX14388.1| glutathione S-transferase M [*Danio rerio*]**

CAGGCGTTTCGTCGTAATTACAGTAGCATGATGAACTGTAACCACACCCCAGCTAAAGC
ATGTTAAATTTTAAACACCCCTGACTATCCGTGACCTTTTTTTATTTATTTTATTTGTTTA
GGTATAAAGACATTATGAATCAGTACCAAACTTTGCAAACTGACTAGTCCACCAAAGAT
GTGACTTACAAGCTATACATAAGAAATAGTACAGATTTGACATTTTTAGACATGACTAA
CCACACTGAGTGCAATCTTCTAATTTTCTGTGATGACTGAAAGACAAGTTACAACAAAA
ATTTGAGATCAGATAATCAATTTAATGTCAACCAGTTATGCAAAATTATTAGTTTCTTAC
CCCTCGTATATATCCCAGTATGCCAATTTCTTTGCCAGCTTGCAAATTGCAACAAGCACT
TTGTTGCAATCTGAGGTGTGGGCAAGCTCAGGTGTAAGAGCAGTGCGACATGACACTTT
GGGGCGTGTCTAGACGGGGGCATCTCAGCAGGTGCAGCTGTAAACATTCACCCTCATGT
GCTCCAAACTCGTAAGACTTTCTTCTGTGGACAGGGTCGTTCTTGGTGCGGTGCAACTGG
TGCGACCGCAGGCTAACAGATCAATGATTCATCTTTCACAGCAATGATATGTGCATACT
AAAATGCATTAAGTTTGTAAATTTAATTGAACAAAGCAGTGCTCTGATTTATTGGGTCA
AAATAAGGTACTGCAATTTAAGCTTTGTTTACAATTCAAATGTAGGATCACAATCCAAA
TGGTGAAATCTATAACAATGAAATGCTTCTTTTCTTTTCAAGGCATAAAATAGACAAAA
ATCAAAGTGCAACACTCACCTAAACACTTTAATCTAAACATAAGTGCTTTTCAACTACA
TTATATCTGTAGACAACAACAGAGATTACTGCTGTTTTGTAATACTTTTATAATCGATAT
GTGTCAGCTGGATCACTGGGATGTAAATTTAACTAATTATCCTTAAAGTGCCTGTTCATC
AAAAGGTTTAAAATAGCTGTCCCAAGAAACTATCACCACATGTACTCTTCAACTGCACT
GACGTTATATTCAAAGCAGTTGATGGTGCACCTCATGATGCTTGATCAAGACGGTCTTGT
CATTTTCACTCCTTCTTGTTTCCCCATTTGGCCATCTTGCCGTTCACAGGTGTTTTCATGA
ACTTGCTTGACTTCATGTATTCTGCAATCTTCTCAAGACTCTCAAAGCGCTCCAGGAAAC
ATCTAAGGTTTTTGTAGTTATCCAAGCATGCTGGTTCATACATACGATGCTGATCCAACA
ACTCGTACATGATGAAATCCACAAATGTGATCTTGTCCCCAGCAAACCACTTCCTGTCA
CCAAGGAAGTCAGAGAACTGCTTTAGAGTTCCTGGCAGATTCTCATCGTAACATGATTT
GTTTGTGTCAAACTCTCTATAGCAGAGCTGAACAAAACCATTGCGGAAGTCCATCGCCT
GATTCTCCAAGATGTCAACTCTCACCTGCTCTTCTTCAGTTTCCCCACAGAGGTTGTTTTT
GCGGGCGATGTATCTCATTATGGCATTGCTTTGGACTACCTTGGTGTCACCATCCACTAG
GTAGGGCAAATTAGGAAAGTCCAGCCCAAGTTTGAATTTCTCATTTAACCAACAGCTTC
TGTCATAGTCGGGAGCGTCACCACAAGTATAGAACTTATCCTCATACTTAGTACCAGTG
TATTCCAACAGCAGACGGATTGGTTGAGCAAGCCCGCGTATATCCCAATATGCCAATTT
CATTGCCATCTTGCAACAGGCTTCAAAATGAAGTGCAGCCAGGAGCAGCGGAATGGAG
GAGGGGGTGTGGGGGCGCACGTGGTTCTGTGCGGTTGGGCAAAGAGCTTAGAACCCAA
GAG

**Reverse Complement**

CTCTTGGGTTCTAAGCTCTTTGCCCAACCGCACAGAACCACGTGCGCCCCCACACCCCCT
CCTCCATTCCGCTGCTCCTGGCTGCACTTCATTTTGAAGCCTGTTGCAAGATGGCAATGA
AATTGGCATATTGGGATATACGCGGGCTTGCTCAACCAATCCGTCTGCTGTTGGAATAC
ACTGGTACTAAGTATGAGGATAAGTTCTATACTTGTGGTGACGCTCCCGACTATGACAG
AAGCTGTTGGTTAAATGAGAAATTCAAACTTGGGCTGGACTTTCCTAATTTGCCCTACCT

AGTGGATGGTGACACCAAGGTAGTCCAAAGCAATGCCATAATGAGATACATCGCCCGC
AAAAACAACCTCTGTGGGGAAACTGAAGAAGAGCAGGTGAGAGTTGACATCTTGGAGA
ATCAGGCGATGGACTTCCGCAATGGTTTTGTTCAGCTCTGCTATAGAGAGTTTGACACA
AACAAATCATGTTACGATGAGAATCTGCCAGGAACTCTAAAGCAGTTCTCTGACTTCCT
TGGTGACAGGAAGTGGTTTGCTGGGGACAAGATCACATTTGTGGATTTCATCATGTACG
AGTTGTTGGATCAGCATCGTATGTATGAACCAGCATGCTTGGATAACTACAAAAACCTT
AGATGTTTCCTGGAGCGCTTTGAGAGTCTTGAGAAGATTGCAGAATACATGAAGTCAAG
CAAGTTCATGAAAACACCTGTGAACGGCAAGATGGCCAAATGGGGAAACAAGAAGGAG
TGAAAATGACAAGACCGTCTTGATCAAGCATCATGAGGTGCACCATCAACTGCTTTGAA
TATAACGTCAGTGCAGTTGAAGAGTACATGTGGTGATAGTTTCTTGGGACAGCTATTTTA
AACCTTTTGATGAACAGGCACTTTAAGGATAATTAGTTAAATTTACATCCCAGTGATCCA
GCTGACACATATCGATTATAAAAGTATTACAAAACAGCAGTAATCTCTGTTGTTGTCTAC
AGATATAATGTAGTTGAAAAGCACTTATGTTTAGATTAAAGTGTTTAGGTGAGTGTTGC
ACTTTGATTTTTGTCTATTTTATGCCTTGAAAAGAAAAGAAGCATTTCATTGTTATAGAT
TTCACCATTTGGATTGTGATCCTACATTTGAATTGTAAACAAAGCTTAAATTGCAGTACC
TTATTTTGACCCAATAAATCAGAGCACTGCTTTGTTCAATTAAATTTACAAACTTAATGC
ATTTTAGTATGCACATATCATTGCTGTGAAAGATGAATCATTGATCTGTTAGCCTGCGGT
CGCACCAGTTGCACCGCACCAAGAACGACCCTGTCCACAGAAGAAAGTCTTACGAGTTT
GGAGCACATGAGGGTGAATGTTTACAGCTGCACCTGCTGAGATGCCCCCGTCTAGACAC
GCCCCAAAGTGTCATGTCGCACTGCTCTTACACCTGAGCTTGCCCACACCTCAGATTGCA
ACAAAGTGCTTGTTGCAATTTGCAAGCTGGCAAAGAAATTGGCATACTGGGATATATAC
GAGGGGTAAGAAACTAATAATTTTGCATAACTGGTTGACATTAAATTGATTATCTGATC
TCAAATTTTTGTTGTAACTTGTCTTTCAGTCATCACAGAAAATTAGAAGATTGCACTCAG
TGTGGTTAGTCATGTCTAAAAATGTCAAATCTGTACTATTTCTTATGTATAGCTTGTAAG
TCACATCTTTGGTGGACTAGTCAGTTTGCAAAGTTTGGTACTGATTCATAATGTCTTTAT
ACCTAAACAAATAAAATAAATAAAAAAAGGTCACGGATAGTCAGGGGTGTTTAAAATT
TAACATGCTTTAGCTGGGGTGTGGTTACAGTTCATCATGCTACTGTAATTACGACGAAAC
GCCTG

**6. *HSP70***

**>gi|238690471|gb|EU884290.2| heat shock protein 70 (hsp70) mRNA, complete cds [*Megalobrama amblycephala*]**

GGAAAATGTACTTCGGGATAGTCAGGAACAGTGCAAAGAGCGAGACAGCGAAGAGTAC
TATCTAGATAACGAGCCACAATACGCCTGTCGGAAAACATCTAACTCACAAAAGGTAG
AAATTAAAAGAAATCAAACATGTCATCAGCAAAAGGAGTCGCTATTGGCATTGACCTG
GGCACCACCTACTCCTGTGTGGGGGTGTTTCAGCATGGAAAGGTGGAGATCATCGCCAA
TGACCAGGGGAACAGAACAACACCCAGCTATGTTGCCTTCACAGACACAGAGAGGCTC
ATTGGAGATGCAGCTAAAAACCAGGTGGCCATGAACCCCAACAACACTGTGTTTGATGC
CAAGAGGCTGATTGGCAGGAAGTTTGATGACCCAGTTGTGCAGTCTGACATGAAGCACT
GGTCCTTCAAAGTGGTCAGTGATGGAGGAAAACCAAAGGTTCAAGTCGAATACAAAGG
AGAAAACAAGACATTTAATCCTGAAGAAATTTCCTCAATGGTCCTGGTGAAGATGAAGG
AGATTGCTGAAGCTTATCTGGGGCAGAAGGTGACAAACGCAGTTATCACAGTTCCTGCC
TATTTCAATGACTCCCAGAGGCAAGCCACTAAAGACGCCGGAGTAATCGCTGGGCTCAA
CGTCCTCAGAATCATCAACGAGCCCACAGCTGCAGCCATCGCCTACGGCCTTGACAAAG
GCAAAGCAGCAGAACGCAACGTCCTGATCTTTGACCTGGGTGGAGGCACCTTTGACGTG
TCCATCCTGACCATTGAAGACGGCATCTTTGAGGTGAAGGCCACAGCCGGAGACACCCA
TCTGGGTGGCGAGGACTTTGACAACCGCATGGTGAATCACTTTGTAGAAGAATTCAAGA
GGAAGCACAAGAAGGACATCAGTCAGAACAAGAGGGCACTGAGGAGGCTGCGGACAG
CGTGTGAGCGAGCCAAGAGAACCCTCTCCTCCAGCTCTCAGGCCAGCCTTGAGATCGAC
TCGCTGTACGAGGGCATCGACTTCTACACGTCCATCACCAGAGCGCGCTTTGAAGAGAT
GTGCTCAGACCTCTTCAGGGGAACACTGGAGCCTGTGGAGAAAGCCCTGAGAGACGCC
AAGATGGACAAGTCTCAGATCCATGACATCGTTCTGGTTGGTGGATCAACAAGAATCCC
AAAGATCCAGAAGCTTCTGCAGGATTTCTTCAACGGCAGGGACTTGAACAAGAGCATCA
ACCCAGATGAGGCAGTGGCTTACGGTGCAGCGGTGCAAGCCGCCATCCTCATGGGTGAC
ACATCTGGAAATGTCCAGGACCTGCTGCTGCTGGATGTGGCTCCTCTGTCCCTGGGTATT
GAAACCGCCGGTGGAGTCATGACGGCCCTCATCAAACGCAACACCACCATCCCCACCA
AACAGACCCAGACCTTCACCACCTACTCTGACAACCAGCCCGGTGTCCTGATCCAGGTG
TACGAGGGAGAGAGGGCCATGACTAAAGACAACAACCTGCTGGGTAAATTTGAGCTGA
CAGGAATTCCACCTGCACCCCGTGGAGTCCCGCAGATTGAAGTGACCTTTGACATCGAC
GCCAACGGAATCCTAAATGTGTCCGCGGTGGACAAAAGCACTGGAAAAGAGAACAAGA
TCACCATCACCAATGACAAGGGCAGACTGAGCAAAGAGGACATTGAGAGAATGGTGCA
GGAAGCAGATCAGTACAAAGCTGAAGATGATCTGCAAAGAGAGAAGATTGCTGCCAAA
AACTCCCTGGAGTCTTACGCCTTCAACATGAAGAACAGTGTGGAAGATGAGAACCTGAA
AGGCAAGATCAGCGAAGATGACAAGAAGAAAGTTATTGAAAAATGTAACGACACCATC
AGCTGGCTAGAGAACAACCAGCTGGCTGATAAGGAGGAGTATGAACATCAGCTGAAGG
AGCTGGAGAAAGTCTGCAACCCAATCATCACTAAGCTTTATCAGGGAGGGATGCCAGCT
GGAGGCTGTGGAGCTCAGGCACGTGGAGGATCAGGGGCCGCTTCCCAGGGACCAACTA
TTGAAGAGGTGGATTAAAGCACCTTATGAACTCAATGCTGCAGGGACTGATTTCAATCT
TTTCTCTTTGGTTCTTCTATTTTTTTTCAGACCTCATTACAATATGATTCCTCTTTTTTACC
CCCTGATGCTCACACACTCCATCTGCTCTTGAAAACATTTTGCTACTAAAAAGAAAAA

**7. *Pgk1***

**>gi|41388972|gb|AAH65888.1 Pgk1 protein [*Danio rerio*]**

AAAAAAAAAAAAAAAAAAAAGACTCCAGGTCAAAAAAACAGCTGATATATTTTATTATT
ATCATTCTTTTTCCAAGACAGCTCACCTGGGTCTAACATTAGCCATTATAATAATTCCAG
TGCGGCACATCTATCTCATAAGCTGCAGGTCTAGGATGACAAGTGCAATATGGGGAAGA
AAGTGTCTACATTATTACAAGTCCTCAACATTTGTGGGGAGGCAACAAATACCAAATAT
AAATTCTAGAATTACAACACACAGCGGTTTACACTGAATGCACACATTCAGACTACAAC
GGGACACACATAAACACTTAAGCAACAATCATAACAGGAACCGTCATTACTGCTGATGT
CAACCTTTTGCTCATAACGAATGGAAATATCTTGAATGAGAAACTCTTTTGGTAATTTGA
TAGAAGAAAAACAGATGGATGTGTATGAGTGAACAGGGCTGTGTTTCCCAAAAGCATT
GTAAGCCTAAGTAGATCGTAGAACCATTGCTAGAACCATTGCAACCAATAGTCTCTATG
ATCAACTTAGCTCACAATGCTTTTGGGAAACTCAGCCCAGGTCAGGAAGTCTAGCACAG
CATACTGTTAGCTGTCCATAGAGAGTGGAGTAGTTGTCTTTTGCATCCACAATAGGTTAT
AGAATGCTGAGTGAATAAGGATGACAGCTTCTTGGGAGACGAGCACCCAGCCTCTGGCT
GAAGAGCCTCAGAGCGCTTAAACATTGCTGAGGGCATCAACACCAGGCAACACTTTACC
CTCCAGCAGCTCCAGACTGGCTCCACCTCCTGTGCTGACATGACTGACCTTGTCTTCTGT
GTCCCACTTGGCACAGCAGGTTGCTGTGTCTCCCCCACCAATAATGGTGATGCAGCCAT
TTTTGGTCGCTTCCACAACTTTATCCATCAAGTTCTTGGTTCCACGAGAAAAGTTGTCCC
ATTCGAACACACCAACAGGTCCGTTCCACACAATCTGCTTGGCTCTGGCTACAGCCTCC
GCATAGAGCTTTGAGCTCTCAGGACCACAGTCCAGACCCATCCAGCCAGCTGGAATACC
ATCTGCTACTGATGCGGTCCCTGTTGCCGCCTTCTCGTCAAACTTGTCTGCAGTGATGAA
GTCAACAGGAAGTGAGATCTTAACGCCATTCTTCTCTGCTTTAGCCATGAGGTCTTTCAC
AATTTTGGCACCCTCTTCATCATACAAGGAAGTGCCGATCTCCATGTTCTTGAGAACCTT
GAGGAAGGTAAAGGCCATTCCTCCACCAATGATCATCTCATTCACCTTGTCCAACATGT
TGTTGATCAGCTGAATCTTATCTTTGACCTTGGCCCCCCCAAGAATGGCCAGGAAAGGC
CTCTGTGGTTTCTCGAGGGCCATGGCAAAGTAGTCCAGCTCCTTCTTCATTAGAAAACCA
GCTGCCTTCTGAGGGAGATTCACTCCAACCATGGAGCTGTGAGCTCTGTGCGCAGTTCC
AAAGGCATCATTGACGTAAACATCACCCAGCTTGGACAATGAGGCTCTGAAAGAATCG
ATTTCCGCTTGACTTGCTTTGGTCTTGTTCCCCGATGCATCTTTGCCCTTGCCCTCCTCAG
CCAGATGGAAACGCAGGTTCTCCAGCAGAATGACAGAACCTGCAGGTGGGTCTGCACA
GGCTTTCTCCACATCTGGACCCACACAGTCCTTCAGGAACTGAACATCTTTTCCCAGCAG
GCTCTTGAGTTCTGCAGCCACAGGCTCCAGAGAGTACTTGTCTGGCATGGGGACTCCAT
CAGGACGGCCCAAGTGGCTCATCAGGACAACAGCTTTGGCCCCATTATCTAAGCAGTGC
TGAATTGATGGGACTGCAGCCTTGATTCTCTGATTATTTGTTATTGTCTTGTCTTTCATAG
GGACATTGAAGTCAACCCTCATGATCACGCGTTTTCCTTTCACATCCACCTTGTCCAAAG
TGAGTTTGTTTGACAGAGACATTTTGGCTGTAGATTTGCAGAAAAACTCCTATACTGGTG
TGGTGCAGTGCAGCTGGGTATGTTCCCGAATGAGAGTGACCTTAGCTTGATGCTGATGC
ACATAGTGTGGG

**Reverse Complement**

CCCACACTATGTGCATCAGCATCAAGCTAAGGTCACTCTCATTCGGGAACATACCCAGC
TGCACTGCACCACACCAGTATAGGAGTTTTCTGCAAATCTACAGCCAAAATGTCTCTGT
CAAACAAACTCACTTTGGACAAGGTGGATGTGAAAGGAAAACGCGTGATCATGAGGGT
TGACTTCAATGTCCCTATGAAAGACAAGACAATAACAAATAATCAGAGAATCAAGGCT
GCAGTCCCATCAATTCAGCACTGCTTAGATAATGGGGCCAAAGCTGTTGTCCTGATGAG
CCACTTGGGCCGTCCTGATGGAGTCCCCATGCCAGACAAGTACTCTCTGGAGCCTGTGG
CTGCAGAACTCAAGAGCCTGCTGGGAAAAGATGTTCAGTTCCTGAAGGACTGTGTGGGT
CCAGATGTGGAGAAAGCCTGTGCAGACCCACCTGCAGGTTCTGTCATTCTGCTGGAGAA
CCTGCGTTTCCATCTGGCTGAGGAGGGCAAGGGCAAAGATGCATCGGGGAACAAGACC
AAAGCAAGTCAAGCGGAAATCGATTCTTTCAGAGCCTCATTGTCCAAGCTGGGTGATGT
TTACGTCAATGATGCCTTTGGAACTGCGCACAGAGCTCACAGCTCCATGGTTGGAGTGA
ATCTCCCTCAGAAGGCAGCTGGTTTTCTAATGAAGAAGGAGCTGGACTACTTTGCCATG
GCCCTCGAGAAACCACAGAGGCCTTTCCTGGCCATTCTTGGGGGGGCCAAGGTCAAAGA
TAAGATTCAGCTGATCAACAACATGTTGGACAAGGTGAATGAGATGATCATTGGTGGAG
GAATGGCCTTTACCTTCCTCAAGGTTCTCAAGAACATGGAGATCGGCACTTCCTTGTATG
ATGAAGAGGGTGCCAAAATTGTGAAAGACCTCATGGCTAAAGCAGAGAAGAATGGCGT
TAAGATCTCACTTCCTGTTGACTTCATCACTGCAGACAAGTTTGACGAGAAGGCGGCAA
CAGGGACCGCATCAGTAGCAGATGGTATTCCAGCTGGCTGGATGGGTCTGGACTGTGGT
CCTGAGAGCTCAAAGCTCTATGCGGAGGCTGTAGCCAGAGCCAAGCAGATTGTGTGGA
ACGGACCTGTTGGTGTGTTCGAATGGGACAACTTTTCTCGTGGAACCAAGAACTTGATG
GATAAAGTTGTGGAAGCGACCAAAAATGGCTGCATCACCATTATTGGTGGGGGAGACA
CAGCAACCTGCTGTGCCAAGTGGGACACAGAAGACAAGGTCAGTCATGTCAGCACAGG
AGGTGGAGCCAGTCTGGAGCTGCTGGAGGGTAAAGTGTTGCCTGGTGTTGATGCCCTCA
GCAATGTTTAAGCGCTCTGAGGCTCTTCAGCCAGAGGCTGGGTGCTCGTCTCCCAAGAA
GCTGTCATCCTTATTCACTCAGCATTCTATAACCTATTGTGGATGCAAAAGACAACTACT
CCACTCTCTATGGACAGCTAACAGTATGCTGTGCTAGACTTCCTGACCTGGGCTGAGTTT
CCCAAAAGCATTGTGAGCTAAGTTGATCATAGAGACTATTGGTTGCAATGGTTCTAGCA
ATGGTTCTACGATCTACTTAGGCTTACAATGCTTTTGGGAAACACAGCCCTGTTCACTCA
TACACATCCATCTGTTTTTCTTCTATCAAATTACCAAAAGAGTTTCTCATTCAAGATATTT
CCATTCGTTATGAGCAAAAGGTTGACATCAGCAGTAATGACGGTTCCTGTTATGATTGTT
GCTTAAGTGTTTATGTGTGTCCCGTTGTAGTCTGAATGTGTGCATTCAGTGTAAACCGCT
GTGTGTTGTAATTCTAGAATTTATATTTGGTATTTGTTGCCTCCCCACAAATGTTGAGGA
CTTGTAATAATGTAGACACTTTCTTCCCCATATTGCACTTGTCATCCTAGACCTGCAGCT
TATGAGATAGATGTGCCGCACTGGAATTATTATAATGGCTAATGTTAGACCCAGGTGAG
CTGTCTTGGAAAAAGAATGATAATAATAAAATATATCAGCTGTTTTTTGACCTGGAGTC
TTTTTTTTTTTTTTTTTT

**8.** ***Aldh8a1***

**>gi|52218932|ref|NP_001004540.1| aldehyde dehydrogenase family 8 member A1 [*Danio rerio*]**

GTCTCTAAGCTATGAAACACCAACTTGCAAGCAATAAAATTTAACACTGGTTAAAGGAA
TATGACCATTTAAGACCACTAAAATGATTAACCCACATTCGATCACCAAAAACACTATG
AAGCATGTTTTCATGAACTTTATTCCCAGTCACATTATGTCTGATTGCTTCCGCTATATA
ATATACACAGATTTGAAATAACACATGAATCACAGAGTGATTCACTATCCCTGCATATA
TTGCTTCAGTGTTTGATGGTGATGGCTTTAACCTCTGTGAAGAAGTGGTATGAGTCTTTT
CCCCCCTCTCGACCAATGCCAGAGTGTTTCATGCCACCAAATGGAAGATTTAAATCTCT
GACCAGCCAGCAGTTGGTCCAAACCAACCCGGCCTGCAGTTTCCTAGCAACCCTGTGTA
CGCGCCCCACATCTCGGGTCCACACTGTGGCGGATAAGCCGTAGCGTACGCCGTTGGCC
CGTGATATCACTTCCTCCTCTTCATCGAAGGGTGTCACACAGGTTACAGGACCGAAAAT
CTCTTCCTGCATCAATGCGGAGGAGTCTTTTACTCCAGAGATGACGGTGGGCATCATGA
AATAGCCACCCGCATTATGCTGTGGAAGGGTCAGTTTGTCCACACCCTCCCCACAATGC
ACCTGCGCACCTTCTGTCAGCGCCAAAGCAACATAACTTTTAACCTTCTGTAGATGCTCT
TTGCTGATGAGCGCTCCGTTGTCGTTGGAGGGGTCAGAGGGCACGCCAGTTTTCCACAG
GCGAGCCGCCTCCACAAAACGAGTGAGGAATTCTGGGTAAATGCTCCGCTCTACGAAG
ATTCTGCTGGTACAGAGACAGATCTCTCCCTGATTGGAGAAGCTGGATCGTACTGTAGT
GCTGATGCATTGCTCCATGTCAGCATCAGCAAATATAATAGCCGGGTTTTTCCCGCCAA
GCTCCAATGAAAGCTTCTTACAATATGGCGCACTGCGTTCTGTTATCAGTCTTGCTGTAG
CCGTGCTCCCCGTAAATGAGATCAACGGCACGTCAGGGTGAGACACCAAGGCATCCCC
GGCTCGTGGGCCCGTACCGAAGACCATGTTGACCACACCGGGTGGAAAGCCAGCTTCCT
CCAATAGCTGACACATCATCCAGGCAGTGACTGAGGTCATTTCACTAGGTTTTGCAACC
ACAGTGTTGCCTGTAGCAACAGCCGGAGTGATCTTCCAGGTCAGGAGATACAGTGGCAG
GTTCCATGGGCTTATAAGACCAGCCACTCCTACAGGACAGCGTACGGTGTAGTTGAGAC
AGCCCATGTGATCCATCTGACTGCAGTCATTAGTGTGATGCAGGACAGATGAAGCAAAA
AAACGGAAGTTGTACGCTGATCGTGGAATGTCCACATTTCGGGCAAAAGTTATAGTCTT
CCCCTGGTCTTTAGACTCAGCCTGGGCAAACTCCTCCAGTCGTGCCTCTATCAGGTCTGC
CAGTTTATTCAGTACTTTGGATCTCTCTGCTGGGCTCTTAGCTGACCAATCAGGAAAGGC
CTCTTTGGCTGCCTTCACTGCAGCATCCACCTCCTCAGGTCCACTGTCTGGAACTTTGCA
ATAAACCTTTCCAGTTGATGGGTTGAAAGAGTCTATTAGTTTGGAACATGGTACAAACT
TCCCACCGATATAGTTCTCCAGCACTAGATATTTAATATCTTTTGACATTGCAGAAAAAT
GTGAATAAAAATCTGCTTTGCAAACAAAAGGGTCCGAGATGTTTGAGCACCAGTCAGCA
CGCTGTTACAGATGAGCTCTCAGCGGACTCTTTTGCAATCTGTTTACTCAGGTAAATGAG
AGGTTGTATAGGAAACTAAATGGCTTCACCGGCAGGAATGTGTCGACACTGATTAATTA
GATCATGAGTTGGACATTAAAAG

**Reverse Complement**

CTTTTAATGTCCAACTCATGATCTAATTAATCAGTGTCGACACATTCCTGCCGGTGAAGC
CATTTAGTTTCCTATACAACCTCTCATTTACCTGAGTAAACAGATTGCAAAAGAGTCCGC

TGAGAGCTCATCTGTAACAGCGTGCTGACTGGTGCTCAAACATCTCGGACCCTTTTGTTT
GCAAAGCAGATTTTTATTCACATTTTTCTGCAATGTCAAAAGATATTAAATATCTAGTGC
TGGAGAACTATATCGGTGGGAAGTTTGTACCATGTTCCAAACTAATAGACTCTTTCAAC
CCATCAACTGGAAAGGTTTATTGCAAAGTTCCAGACAGTGGACCTGAGGAGGTGGATGC
TGCAGTGAAGGCAGCCAAAGAGGCCTTTCCTGATTGGTCAGCTAAGAGCCCAGCAGAG
AGATCCAAAGTACTGAATAAACTGGCAGACCTGATAGAGGCACGACTGGAGGAGTTTG
CCCAGGCTGAGTCTAAAGACCAGGGGAAGACTATAACTTTTGCCCGAAATGTGGACATT
CCACGATCAGCGTACAACTTCCGTTTTTTTGCTTCATCTGTCCTGCATCACACTAATGAC
TGCAGTCAGATGGATCACATGGGCTGTCTCAACTACACCGTACGCTGTCCTGTAGGAGT
GGCTGGTCTTATAAGCCCATGGAACCTGCCACTGTATCTCCTGACCTGGAAGATCACTC
CGGCTGTTGCTACAGGCAACACTGTGGTTGCAAAACCTAGTGAAATGACCTCAGTCACT
GCCTGGATGATGTGTCAGCTATTGGAGGAAGCTGGCTTTCCACCCGGTGTGGTCAACAT
GGTCTTCGGTACGGGCCCACGAGCCGGGGATGCCTTGGTGTCTCACCCTGACGTGCCGT
TGATCTCATTTACGGGGAGCACGGCTACAGCAAGACTGATAACAGAACGCAGTGCGCC
ATATTGTAAGAAGCTTTCATTGGAGCTTGGCGGGAAAAACCCGGCTATTATATTTGCTG
ATGCTGACATGGAGCAATGCATCAGCACTACAGTACGATCCAGCTTCTCCAATCAGGGA
GAGATCTGTCTCTGTACCAGCAGAATCTTCGTAGAGCGGAGCATTTACCCAGAATTCCT
CACTCGTTTTGTGGAGGCGGCTCGCCTGTGGAAAACTGGCGTGCCCTCTGACCCCTCCA
ACGACAACGGAGCGCTCATCAGCAAAGAGCATCTACAGAAGGTTAAAAGTTATGTTGC
TTTGGCGCTGACAGAAGGTGCGCAGGTGCATTGTGGGGAGGGTGTGGACAAACTGACC
CTTCCACAGCATAATGCGGGTGGCTATTTCATGATGCCCACCGTCATCTCTGGAGTAAA
AGACTCCTCCGCATTGATGCAGGAAGAGATTTTCGGTCCTGTAACCTGTGTGACACCCTT
CGATGAAGAGGAGGAAGTGATATCACGGGCCAACGGCGTACGCTACGGCTTATCCGCC
ACAGTGTGGACCCGAGATGTGGGGCGCGTACACAGGGTTGCTAGGAAACTGCAGGCCG
GGTTGGTTTGGACCAACTGCTGGCTGGTCAGAGATTTAAATCTTCCATTTGGTGGCATGA
AACACTCTGGCATTGGTCGAGAGGGGGGAAAAGACTCATACCACTTCTTCACAGAGGTT
AAAGCCATCACCATCAAACACTGAAGCAATATATGCAGGGATAGTGAATCACTCTGTGA
TTCATGTGTTATTTCAAATCTGTGTATATTATATAGCGGAAGCAATCAGACATAATGTGA
CTGGGAATAAAGTTCATGAAAACATGCTTCATAGTGTTTTTGGTGATCGAATGTGGGTT
AATCATTTTAGTGGTCTTAAATGGTCATATTCCTTTAACCAGTGTTAAATTTTATTGCTTG
CAAGTTGGTGTTTCATAGCTTAGAGAC

**9. *Sord***

**>gi|289063382|ref|NP_001165890.1| sorbitol dehydrogenase [*Danio rerio*]**

GATGGCCGTAAACAAGGGGCGTCAGTGCGCGTGTGAGTGAGTGAGAGCGCGCTTGATC
CGTACATGAGCCAAATAATAAGTATATGAACAGTGAGTACACGAGTTATCTGGAGTTTA
TCAGTTGTTCTATACGTGTGTAAGTGAATAAAATGGATAAAGACAACCTCTCCGTGGTC
CTGCACTCTAAAGGAGATATCAGACTGGAACAGCGCCCGATCCCGGAGCCCGGACCAA
ATGATGTTCTACTTCAGATGCACTCTGTGGGAATTTGTGGATCAGACGTGCACTATTGGC
AGAACGGCCGCATCGGGGACTATGTAGTGAAACAGCCCATGATACTGGGACATGAAGC
CTCTGGTCGTGTGGTGAAAGTGGGATCTGATGTGATGCACCTCAAACCAGGAGACAGAG
TTGCCGTTGAGCCTGGAGTTCCTCGTGAAGTAGACGAGTTCTTTAAATCTGGACACTAC
AACCTTTCTCCCAGCATATTCTTCTGTGCCACTCCTCCAGATGATGGAAACCTGTGCAGA
TACTACAAACACAGTGCTAACTTCTGCTACAAACTTCCCGATAATGTGACTTATGAGGA
GGGAGCCCTGATTGAGCCCCTGTCAGTGGGCATTCATGCCTGCAGGAGGGCAGGAGTCA
CTCTTGGAAGCTCAGTGTTTGTCTGCGGTGCAGGACCAATTGGACTAGTTTCTTTGTTGG
CGGCCAAAGCCATGGGTGCTTCGCAAGTAATAATAAGTGACTTGTCCTCTGATCGGCTT
GCCAAGGCTAAAGAGATCGGAGCAGACTTCCTGCTTCATGTGAAGAAAGAGGATGGAC
CACAGGACATAGCCAAAAAAGTGGAGGGAATGCTGGGTTGCATGCCTCAAATCAGCAT
TGAATGCACTGGAGTGCAGAGCAGCATTCAAACAGCCATCTATGCTACTCATTCAGGAG
GAGTGGTGGTGTTGGTTGGGCTTGGTACTGAGATGACCACCATACCTCTTCTGAATGCA
GCAGTCAGAGAAGTTGACATCAGAGGCGTATTCCGCTACTGTAATACCTGGCCGGTGGC
CATTGCTATGTTGGCATCTAAGAAGGTGAACGTCAAGCCACTGGTCACCCACCGTTTCC
CGCTGGAGCAGGCTGTACAGGCCTTTGAGACCACCCGTCAGGGTCTTGGGGTTAAAGTT
ATGTTAAAATGTGACAAGAATGACCAGAACCCATGAGATCAATTACTAGGCCCACATCT
GATTATTGCCTTGCTACAGTCACTTGAATACATTTGTTTAACCCAGCTGATTTTGACAAT
AACCTCACTATCTTGTCTCAGGTACAGCAGTCACAGGAATTCAGTGACTACTCTTTACAG
TAATTATTTATAATTTAATTTTCTTTGTCATTTATGAATCATGAAAAGTGTTAAAATTGTA
TGCAACATTGTCAGAGTGCAGTTTTTAATGGCATGCTAGAATAATAGCAGATCATTTTTA
CAGCTTACTTACCTTGATCTTGTTCACAAAAAATGAATCTTTATCAAACTTCAGTTGTCT
GTGTCATTATTTTAAGTACAGTTCTCATAGCCACGAGAGCACAGTGCACATTTTTAATCA
ACTTGAATGACCAACCGAGAAA
